# Visible-light-induced cross-coupling of aryl iodides with hydrazones *via* an EDA-complex†

**DOI:** 10.1039/d2sc01909d

**Published:** 2022-05-23

**Authors:** Pan Pan, Shihan Liu, Yu Lan, Huiying Zeng, Chao-Jun Li

**Affiliations:** The State Key Laboratory of Applied Organic Chemistry, College of Chemistry and Chemical Engineering, Lanzhou University 222 Tianshui Road Lanzhou 730000 P. R. China zenghy@lzu.edu.cn; School of Chemistry and Chemical Engineering, Chongqing Key Laboratory of Theoretical and Computational Chemistry, Chongqing University Chongqing 400030 China lanyu@cqu.edu.cn; College of Chemistry, Institute of Green Catalysis, Zhengzhou University Zhengzhou 450001 P. R. China; Department of Chemistry, FQRNT Centre for Green Chemistry and Catalysis, McGill University 801 Sherbrooke Street West Montreal Quebec H3A 0B8 Canada cj.li@mcgill.ca

## Abstract

A visible-light-induced, transition-metal and photosensitizer-free cross-coupling of aryl iodides with hydrazones was developed. In this strategy, hydrazones were used as alternatives to organometallic reagents, in the absence of a transition metal or an external photosensitizer, making this cross-coupling mild and green. The protocol was compatible with a variety of functionalities, including methyl, methoxy, trifluoromethyl, halogen, and heteroaromatic rings. Mechanistic investigations showed that the association of the hydrazone anion with aryl halides formed an electron donor–acceptor complex, which when excited with visible light generated an aryl radical *via* single-electron transfer.

## Abstract 2

Visible-light-induced catalyst-free cross-coupling of aryl iodides with hydrazones *via* single-electron-transfer was reported. The mechanistic investigations showed that the association of hydrazone anion with aryl iodides formed an EDA complex.

## Graphical abstract

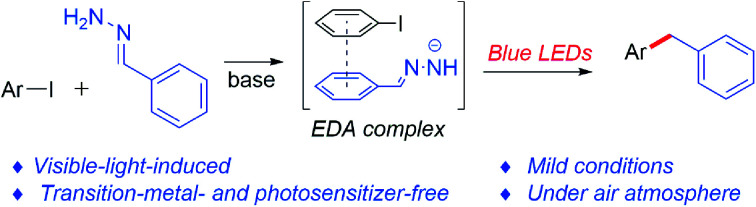

## Introduction

**Scheme 1:**
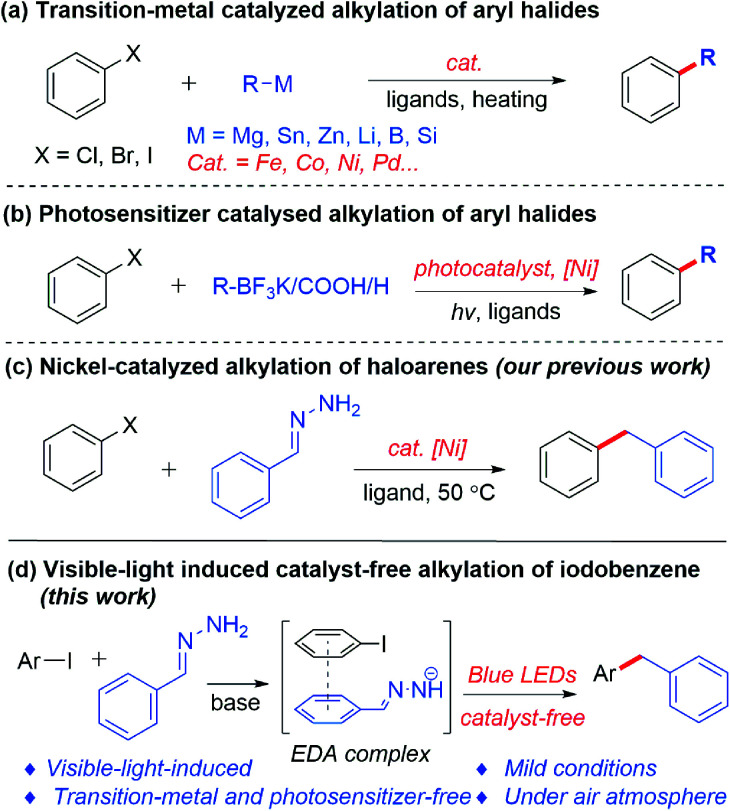
Methods for alkylation of aryl halides.

The construction of the carbon–carbon bond plays an important role in organic synthesis.^1^ In the past few decades, research interest has been primarily focused on transition-metal-catalyzed carbon–carbon bond formation, which allows the reliable, accurate and efficient synthesis of various important chemical products.^2^ Normally, transition-metal-catalyzed alkylations of aryl halides are achieved by cross-coupling between organic halides^3^ and organoboron reagents,^4^ organosilicon reagents^5^ or organometallic reagents^6^ as alkylation reagents (Scheme 1a). Such reactions have shown excellent reproducibility, efficiency and selectivity. On the other hand, visible-light-induced construction of carbon–carbon bonds has attracted increased attention in recent years. Compared with the traditional thermal reactions, photoredox-based chemistry can be carried out under milder conditions. Recently, a new strategy has emerged, by combining photoredox catalysis and transition metals, as a powerful tool in cross-coupling reactions. Using such a strategy, potassium trifluoroborates, carboxylic acids and aliphatic compounds were successfully coupled with aryl halides, respectively (Scheme 1b).^7^

In spite of the great advances of the transition-metal-catalyzed coupling reaction and visible-light-induced dual catalysis system, there are still some limitations and challenges of these coupling reactions, such as the high cost of transition metals and ligands, the need for photosensitizers, the presence of trace transition-metal residues in products, as well as the use of organometallic or metalloid reagents as nucleophiles which requires stoichiometric metals and are often sensitive to air and moisture. For the latter, our group recently developed hydrazone (derived from aldehyde/ketone) chemistry as organometallic surrogates for various classical organometallic reactions,^8^ among which the cross-coupling of aryl iodides or bromides with hydrazones led to the alkylation of aryl halides catalyzed by nickel at 50 °C (Scheme 1c).^8*m*,9^ However, a transition metal and heating were required in this transformation. To pursue greener and more sustainable synthesis, recently our group has developed a series of photoinduced reactions in the absence of a transition metal and an external photosensitizer under mild conditions.^10,15^ Recently, Hashmi’s group reported an ultraviolet light induced metal-free cross-coupling of *N*,*N*-dialkylhydrazones with perfluoroalkyl iodides to generate perfluoroalkylated *N*,*N*-dialkylhydrazones.^11^ Herein, we report a visible-light-induced transition-metal- and external photosensitizer-free cross-coupling between aryl iodides and hydrazones under mild conditions (Scheme 1d).

## Results and discussion

We started our investigation using 2-fluorobenzaldehyde hydrazone (1a) and 1-chloro-3-iodobenzene (2a) as model substrates. Fortunately, a trace amount of the desired product 3a was detected by GC-MS (Table 1, entry 1). Encouraged by this result, a series of organic and inorganic bases, such as DBU, K_2_CO_3_, LiOH, KOH and NaOH, were tested (entries 2–6). Among these bases, the highest yield (61%) was obtained with NaOH (entry 6). Different solvents were investigated (entries 6–10), with DMSO being the optimal solvent (entry 6). Adjusting the ratio of 1a and 2a to 4 : 1 gave a higher yield (66%) than others (entries 11–14). Increasing the amount of NaOH to 2.0 equiv. enhanced the yield to 69% (entry 15). To inhibit the side reaction (Wolff–Kishner–Huang reaction), we attempted to carry out the reaction at a lower temperature; however, the DMSO solvent froze in a 15 °C cold bath. Therefore, 50.0 μL DMF was added to the reaction system as a co-solvent to lower the melting point. The results indicated that adding the co-solvent did not affect the yield (entries 15 *vs.* 16). A slightly higher yield was obtained when the reaction temperature was lowered to 15 °C (entry 17). Regarding the reaction time, the results indicated that 24 h was enough to obtain a 73% yield (entries 17–19). An inert atmosphere was not crucial to this reaction, as the same yield can be afforded when the reaction was carried out under an air atmosphere (entry 20). Control experiments showed that no product was detected without the base (entry 21). A lower yield (23%) was obtained when the reaction was performed in the dark (entry 22). This result indicated that a nucleophilic substitution process was possible. To further investigate this pathway, different substrates were reacted at high temperature in the absence of light, obtaining only trace amounts of products (see Scheme S1 in the ESI†). These results indicated that light was important for this transformation.

**Table 1:** Optimization of the reaction conditions^a^

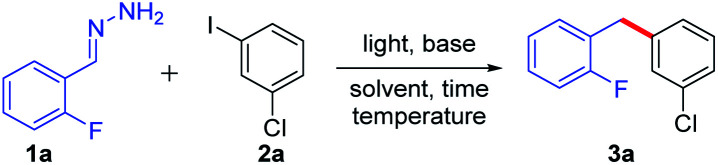				
Entry	Base	Solvent	Time/h	Yield^b^ (%)
1	DABCO	DMSO	24	Trace
2	DBU	DMSO	24	Trace
3	K_3_CO_3_	DMSO	24	4
4	LiOH	DMSO	24	23
5	KOH	DMSO	24	58
6	NaOH	DMSO	24	61
7	NaOH	CH_3_CN	24	15
8	NaOH	DMF	24	31
9	NaOH	Ethanol	24	Trace
10	NaOH	CH_2_Cl_2_	24	Trace
11^c^	NaOH	DMSO	24	66
12^d^	NaOH	DMSO	24	32
13^e^	NaOH	DMSO	24	45
14^f^	NaOH	DMSO	24	45
15^c^^,^^g^	NaOH	DMSO	24	69
16^c^^,^^g^^,^^h^	NaOH	DMSO	24	69
17^c^^,^^g^^,^^h^^,^^i^	NaOH	DMSO	24	73
18^c^^,^^g^^,^^h^^,^^i^	NaOH	DMSO	12	73
19^c^^,^^g^^,^^h^^,^^i^	NaOH	DMSO	36	73
20^c^^,^^g^^,^^h^^,^^i^^,^^j^	NaOH	DMSO	24	73
21^c^^,^^h^^,^^i^^,^^j^	—	DMSO	24	0
22^c^^,^^g^^,^^h^^,^^i^^,^^j^^,^^k^	NaOH	DMSO	24	23

a General conditions: 1a (2.0 equiv.), 2a (0.2 mmol) and base (1.5 equiv.) in solvent (1.0 mL) were irradiated with blue LEDs (425 nm, 3 W × 2) for 24 h under an argon atmosphere at 35 °C.

b Yields were determined by ^1^H NMR using nitromethane as the internal standard.

c 1a : 2a = 4 : 1.

d 1a : 2a = 1 : 1.

e 1a : 2a = 1 : 2.

f 1a : 2a = 1 : 4.

g NaOH (2.0 equiv.).

h 50.0 μL DMF was added as the co-solvent.

i 15 °C.

j Air atmosphere.

k In the dark.

With the optimized reaction conditions in hand, we examined the compatibility of diverse aryl halides. As shown in Table 2, when other halides, such as chloride and fluoride, were substituted on the aryl rings, the reaction showed good chemo-selectivity with moderate to high yields regardless of the substitution at the *ortho*-, *para*-, and *meta*-positions (3a–f). A 1.5 mmol scale reaction was also investigated, obtaining product 3a in 55% yield. The aryl iodide bearing an electron-donating group, such as methyl, *tert*-butyl, phenyl, methoxyl and trifluoromethoxyl, also reacted smoothly giving moderate to high yields (3g–n). Due to the methyl group at the *ortho*-position of aryl iodides having greater steric hindrance than a fluorine atom (3f*vs.*3g), only 15% yield of 3g was obtained. It is worth noting that tri-substituted aryl iodides also gave good to high yields (3k–l). Aryl iodides bearing an electron-withdrawing group, such as trifluoromethyl, also afforded moderate yields (3p–q). Furthermore, the reaction showed good tolerance towards heterocycles, such as morpholine (3r), quinoline (3s), thiophene (3t) and pyridine (3u). A substrate without substitution on both of the aromatic rings generated a 60% yield of coupling product 3v.

**Table 2:** The scope of iodobenzene substrates^a^

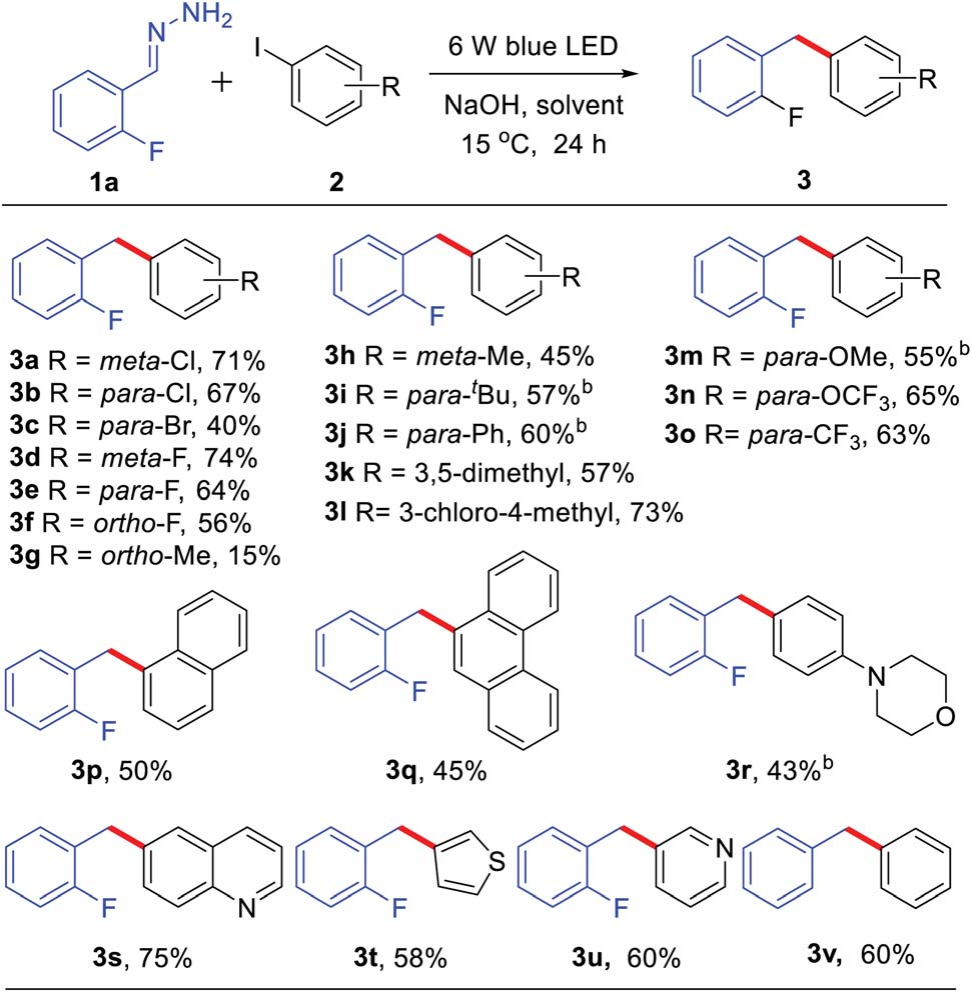

a General conditions: 1a (0.8 mmol), 2a (0.2 mmol) and NaOH (0.4 mmol) in solvent (DMSO 1.0 mL + DMF 50.0 μL) were irradiated with blue LEDs (425 nm, 3 W × 2) for 24 h under an air atmosphere at 15 °C.

b 36 h.

Subsequently, we explored the scope of hydrazone substrates. As shown in Table 3, both alkyl and aryl substituents at the *para*-, *meta*- and *ortho*-positions of hydrazones generated the corresponding products in good to high yields (3w–3ac). It was interesting to note that a sterically hindered hydrazone (bearing dimethyl at C2 and C6 positions) also afforded a 66% yield of the corresponding product 3ad. Halogen-containing (Cl and F) hydrazones also reacted smoothly (3ae–ah). Moreover, hydrazones with both strongly electron-donating groups and strongly electron-withdrawing groups gave moderate to high yields of the corresponding coupling products (3ai–am). Fused-ring and heteroaromatic ring hydrazones were also effective (3an–ao). Interestingly, alkyl hydrazone, which did not work in the nickel-catalyzed system,^9^ could also afford 39% yield of product 3ap.

**Table 3:** The scope of hydrazone substrates^a^

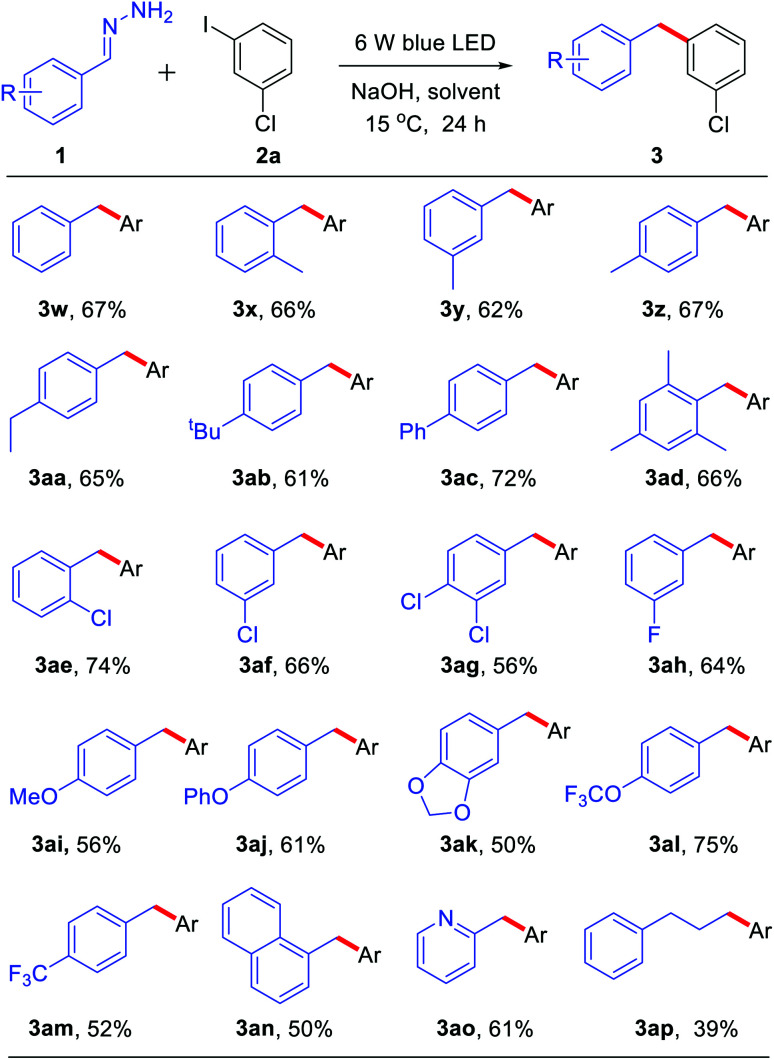

a General conditions: 1a (0.8 mmol), 2a (0.2 mmol) and NaOH (0.4 mmol) in solvent (DMSO 1.0 mL + DMF 50.0 μL) were irradiated with blue LEDs (425 nm, 3 W × 2) for 24 h under an air atmosphere at 15 °C; isolated yields are given.

**Scheme 2:**
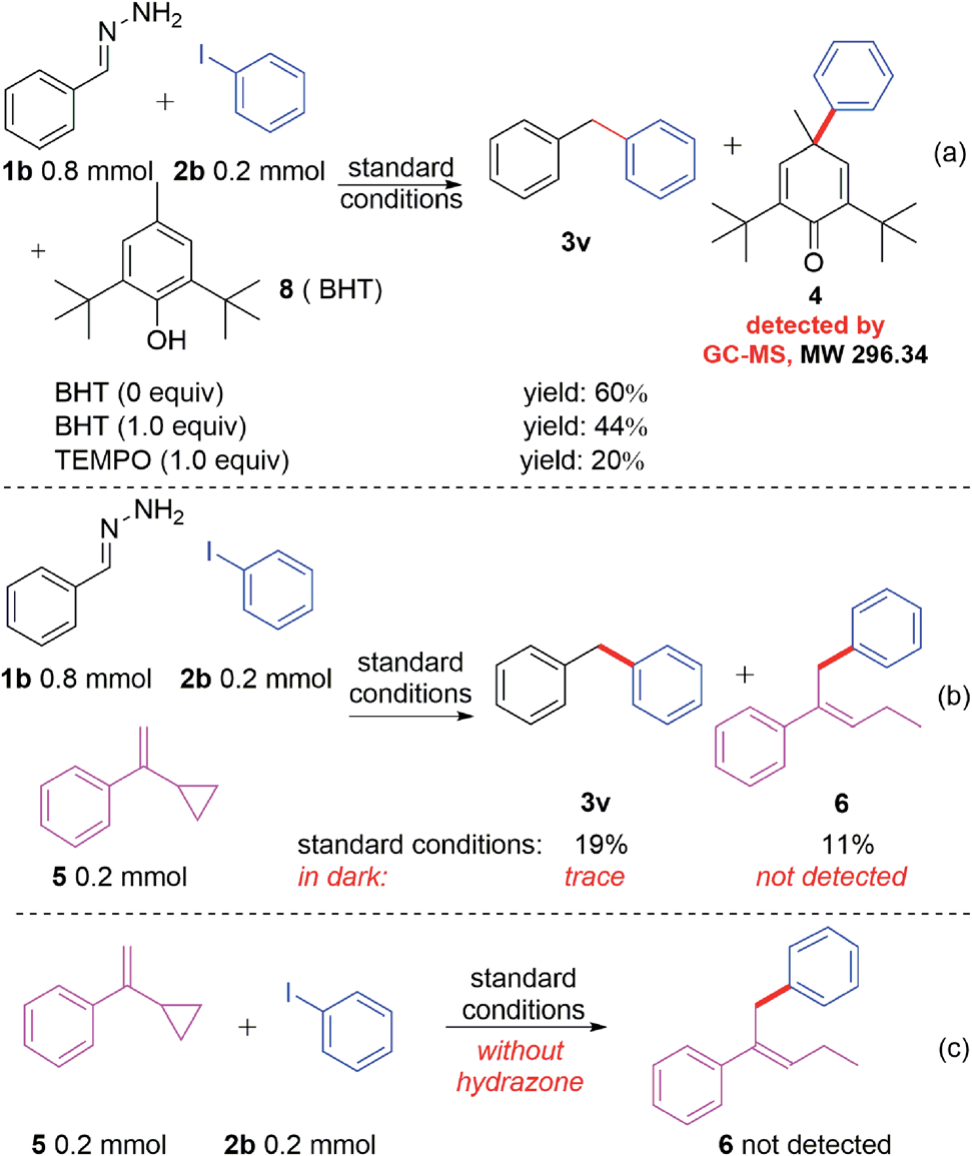
Control experiments.

**Fig. 1:**
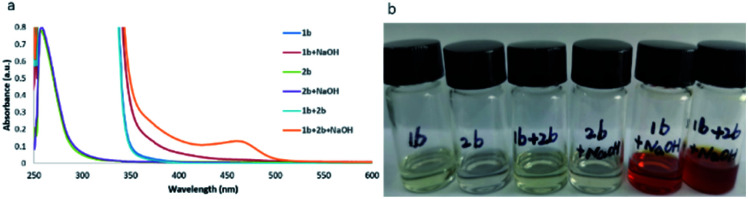
(a) UV-vis experiments. (b) Photos of DMSO solution with different components.

In spite of having achieved a broad substrate scope, a key question remained: how does the reaction occur? Neither of the reactants nor the base and solvent have visible absorption. To investigate the reaction mechanism, several control experiments were carried out. As shown in Scheme 2, when different amounts of BHT (2,6-di-*tert*-butyl-4-methylphenol) or TEMPO (2,2,6,6-tetramethylpiperidin-1-oxyl) were added to the reaction system under the standard conditions (Scheme 2a), the yields decreased accordingly. The radical trapped product 4 can be detected by GC-MS when BHT was used as the radical trapper. This information illustrated that the reaction might involve phenyl radical intermediates. To further confirm this proposal, (1-cyclopropylvinyl)benzene (5) was added as a competitive substrate, the phenyl addition and cyclopropyl ring opening product 6 was generated in 11% yield, and the yield of the desired product 3u was reduced to 19% (Scheme 2b). When the reaction was carried out in the dark, a trace amount of compound 3u can be obtained without compound 6 being detected by GC-MS (Scheme 2b). These experiments further supported the existence of the phenyl radical, which was produced by the irradiation of blue light in this reaction. However, no product 6 was detected when compounds 5 and 2b were reacted under standard reaction conditions in the absence of hydrazone 1b (Scheme 2c), which demonstrated that hydrazone 1b facilitated the generation of the phenyl radical from phenyl iodide (2b). To further understand how hydrazone 1b reacted with 2b to form the phenyl radical, we carried out a series of ultraviolet-visible experiments. When hydrazone 1b was mixed with NaOH, the colorless mixture turned brownish red (Fig. 1b), and the corresponding bathochromic shift was observed in the ultraviolet-visible spectrum (Fig. 1a, red line), compared with that of hydrazone 1b in the absence of NaOH (Fig. 1b, blue line), illustrating that hydrazone 1b might form a hydrazone anion with NaOH and lead to a red shift (but still not in the visible region) in the ultraviolet-visible spectrum. Furthermore, the mixture of 1b and 2b did not absorb in the visible light region either (Fig. 1b). However, adding NaOH powder to this mixture (1b and 2b) generated a new absorption peak at *ca.* 460 nm in UV/vis absorption spectrometry (Fig. 1b, yellow line), which was attributed to an EDA complex between the hydrazone anion and phenyl iodide (2b). The color of this mixture (1b + 2b + NaOH) also became slightly darker (Fig. 1b, right).

**Fig. 2:**
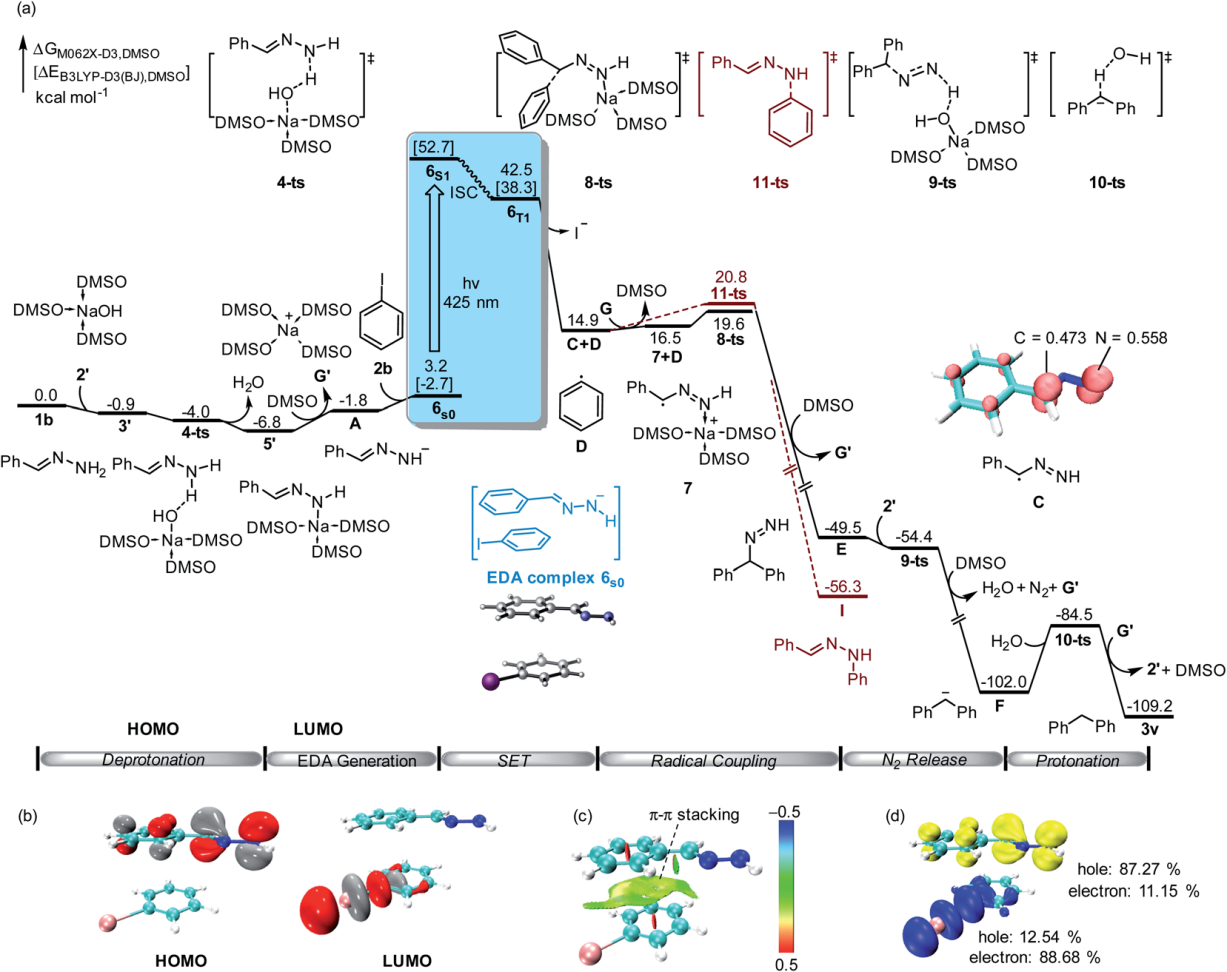
(a) Free energy profile for the alkylation of aryl halides with hydrazones. The energy values are in kcal mol^−1^ and represent the relative free energies calculated at the M06-2X-D3/6-311+G(d,p)-SDD/SMD(DMSO)//B3LYP-D3(BJ)/6-31G(d)-SDD/SMD(DMSO) level of theory in DMSO solvent. The energy values in square brackets are in kcal mol^−1^ and represent the electronic energy calculated at the B3LYP-D3(BJ)/6-31G(d)-SDD/SMD(DMSO) level of theory in DMSO solvent. The Mulliken atomic spin densities on the corresponding atoms are given (isovalue: 0.01). (b) Computed FMOs of EDA complex 6_s0_ (isovalue: 0.05). (c) Non-covalent interaction (NCI) analysis of 6_s0_ (blue: attraction; green: weak interaction; red: repulsion). (d) Hole–electron analysis of 6_S1_.

To further validate the reaction mechanism, we conducted density functional theory (DFT) calculations at the M06-2X-D3 level of theory (computational details are given in the ESI†). As shown in Fig. 2, the calculated free energy of hydrazone 1b is set to the relative zero value, which can be deprotonated by sodium hydroxide 2′ through a barrier-less process leading to the formation of benzylidenehydrazinide 5′. With the dissociation of sodium cation G′, EDA complex 6_s0_ would be formed by the combination of hydrazinide A and iodobenzene 2b through π–π interaction (Fig. 2c). A singlet diradical intermediate 6_S1_ could be found by visible light absorption. Besides, the calculated hole-electron analysis^12^ of 6_S1_ shows that 87.27% of the holes are attributed to the hydrazone fragment, while 88.68% of the electrons are located on the iodobenzene fragment (Fig. 2d). This means that electrons flow from hydrazone to iodobenzene, which is fully in agreement with TD-DFT calculations. Subsequently, it could convert into triplet state 6_T1_ through the intersystem crossing (ISC) process. TD-DFT calculation showed that the excitation of the ground-state EDA complex 6_S0_ to its excited state 6_S1_ requires the absorption of light at 443 nm (55.4 kcal mol^−1^), which is consistent with the experimental conditions (visible light enabled). After this process, the obtained hydrazone radical C and phenyl radical D underwent an internal single electron transfer. After the release of iodide, radical coupling of C with D through transition state 8-ts afforded benzhydryldiazene intermediate E with an energy barrier of only 4.7 kcal mol^−1^. Deprotonation of benzhydryldiazene E by NaOH led to further denitrogenation through a one-step barrier-less process *via* transition state 9-ts. The generated diphenylmethanide F could be protonated by water *via* transition state 10-ts to yield target product 3v.

Moreover, the DFT calculation found that the spin density of intermediate C is shared by both carbon and nitrogen atoms. Therefore, C–N bond formation through radical coupling with the phenyl radical was also considered by DFT calculation. As shown in Fig. 2 (brown lines), C–N bond formation would occur *via* transition state 11-ts, leading to the formation of the phenylhydrazine product I. The relative free energy of the transition state 11-ts is 1.2 kcal mol^−1^ higher than that of 8-ts. Therefore, diphenylmethane 3v was obtained as the major product in this experiment. In our DFT calculation, the aromatic nucleophilic substitution pathway was also considered. As shown in ESI Fig. S2,† when benzylidenehydrazinide 5′ is formed, an intermolecular aromatic nucleophilic substitution with iodobenzene would take place *via* transition state 12-ts to afford intermediate E directly. However, the calculated free energy barrier is as high as 30.0 kcal mol^−1^. Moreover, the radical substitution pathway was also considered. The DFT calculation results show that the activation free energy for the radical substitution is as high as 28.1 kcal mol^−1^ (see the ESI for details, Fig. S3†).

**Scheme 3:**
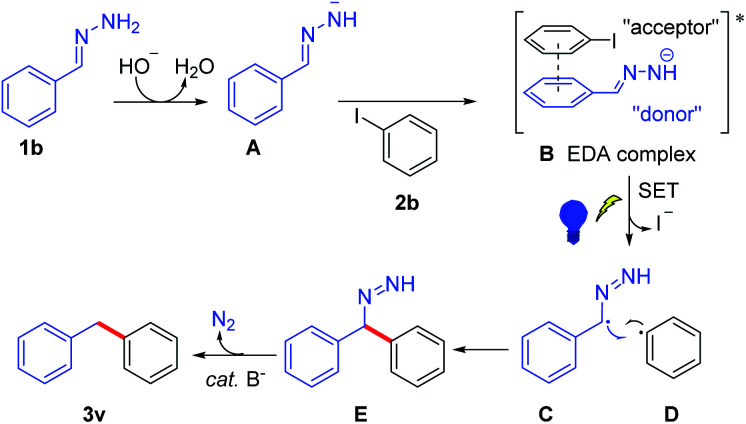
Possible mechanism.

According to the control experiments and the DFT results, a possible mechanism is proposed in Scheme 3. The major pathway was that the base abstracts a proton from hydrazone and generates hydrazone anion A, which forms EDA complex B with iodobenzene 2b. The EDA complex^13^ is irradiated with blue light, affording benzylic radical C and aryl radical D*via* single electron transfer with the release of iodide. Then, cross-coupling of C and D forms a new C–C bond and generates intermediate E. Subsequent N_2_-extrusion under base condition yields 3v,^14^ which abstracts a proton from H_2_O or solvent affording the product 3v (the source of the hydrogen atom has been studied by several deuterium labelling experiments, see Scheme S2 in the ESI†).

## Conclusions

In summary, we have established a visible-light-induced cross-coupling of aryl iodides with hydrazones as alkylation reagents *via* intermolecular single electron transfer of an EDA complex. This reaction can proceed smoothly in the absence of transition metals, ligands and photosensitizers, as well as have good tolerance to moisture, air and various functional groups. Mechanistic investigations and DFT calculations revealed the involvement of a single-electron-transfer process within the EDA complex. This method provides a greener and efficient alternative strategy to the well-established transition-metal-catalyzed cross-coupling between aryl halides with organometallic reagents.

## Data availability

All experimental and computational data is available in the ESI.†

## Author contributions

P. P. performed the experiments, S. L. performed DFT calculations. H. Z. and C.-J. L. supervised the project, Y. L. supervised the DFT calculations. All the authors analyzed the data, discussed results and contributed to the manuscript.

## Conflicts of interest

There are no conflicts to declare.

## Supplementary Material

SC-013-D2SC01909D-s001
