# Supplementary material for: Visible-light-induced cross-coupling of aryl iodides with hydrazones via an EDA-complex
Source: Chem Sci. 2022 May 23;13(24):7165–71. doi: 10.1039/d2sc01909d (PMC9214885; doi:10.1039/d2sc01909d)

Electronic Supplementary Material (ESI) for Chemical Science.
This journal is © The Royal Society of Chemistry 2022

Visible-light-induced cross-coupling of aryl iodides with hydrazones via EDA complex

Pan Pan[a], Sihan Liu[b], Yu Lan[*,b,c], Huiying Zeng[*,a] and Chao-Jun Li[*,d]

[a] The State Key Laboratory of Applied Organic Chemistry, and College of Chemistry and Chemical Engineering, Lanzhou University, 222 Tianshui Road, Lanzhou, Gansu 730000, China

[b] School of Chemistry and Chemical Engineering, Chongqing Key Laboratory of Theoretical and Computational Chemistry, Chongqing University, Chongqing 400030, China

[c] College of Chemistry and Institute of Green Catalysis, Zhengzhou University, Zhengzhou 450001, P. R. China

[d] Department of Chemistry, and FQRNT Centre for Green Chemistry and Catalysis, McGill University, 801 Sherbrooke Street West, Montreal, Quebec H3A 0B8, Canada

*Corresponding Authors: lanyu@cqu.edu.cn, zenghy@lzu.edu.cn and cj.li@mcgill.ca

Table of Contents

1. **General information S2**
2. **Optimization of reaction conditions S2**
3. **General procedure for C-C cross-coupling S4**
4. **Deuterium labeling experiment S5**
5. **Scale up experiment S6**
6. **The promotion of light on the reaction S6**
7. **Computational details S7**
9. **Characterization data S20**
9. **References S39**
10. **Copies of ^{1}H NMR, ^{13}C NMR and ^{19}F NMR S40**

1. General information

All reagents were purchased from commercial sources and used without further purification unless otherwise stated. ^{1}H, ^{19}F and ^{13}C NMR spectra were recorded at room temperature on Varian Mercury plus 300 MHz, Bruker AV400 MHz and Agilent INOVA 600 MHz with TMS as an internal standard and $CDCl_3$ (unless otherwise stated) as solvent. All reactions were carried out in argon atmosphere unless otherwise stated. Silica gel (300-400 mesh) was used for flash column chromatograph, eluting (unless otherwise stated) with an ethyl acetate/ petroleum ether (v/v =1/250) mixture. GC-MS analyses were made by Thermo Scientific Trace 1300 by means of EI. HRMS analyses were made at Lanzhou University, and Technical Institute of Physics and Chemistry & University of Chinese Academy of Sciences by means of ESI and EI. Melting points were measured on micro melting point apparatus and uncorrected. All solvents were purified and dried by standard techniques.

2. Optimization of the reaction conditions

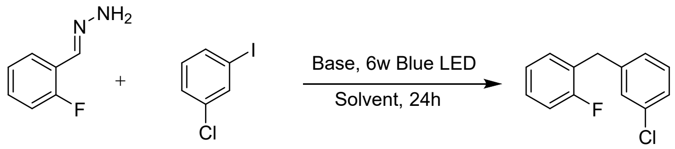

1a **2a** **3a**

2.1) Effects of bases^{a}

Entry	Conditions	Yield (%)^{b}
1	DABCO	trace
2	DBU	trace
3	K_3CO_3	4
4	LiOH	23
5	KOH	58
6	NaOH	61

^{a} General conditions: **1a** (0.4 mmol), **2a** (0.2 mmol) and base (1.5 equiv) in solvent (DMSO 1.0 mL) irradiated by blue LEDs (425 nm, 3W^{x}2) for 24 h under an argon atmosphere at 35 °C. ^{b} Yields were determined by ^{1}H NMR using nitromethane as internal standard.

2.2) Effects of solvent^{a}

Entry	Conditions	Yield (%)^{b}

1	DMSO	58
2	CH_3CN	15
3	DMF	31
4	Ethanol	trace
5	CH_2Cl_2	trace

[a] General conditions: **1a** (0.4 mmol), **2a** (0.2 mmol) and NaOH (1.5 equiv) in solvent (DMSO 1.0 mL) irradiated by blue LEDs (425 nm, 3W×2) for 24 h under an argon atmosphere at 35 °C. [b] Yields were determined by ^{1}H NMR using nitromethane as internal standard.

2.3) Effects of ratio of substrates[a]

Entry	Conditions	Yield(%)[b]
1	2:1	58
2	4:1	66
3	1:1	32
4	1:2	45
5	1:4	45

[a] General conditions: NaOH (1.5 equiv) and solvent (DMSO 1.0 mL) irradiated by blue LEDs (425 nm, 3W×2) for 24 h under an argon atmosphere at 35 °C. [b] Yields were determined by ^{1}H NMR using nitromethane as internal standard.

2.4) The amount of base[a]

Entry	Conditions	Yield (%)[b]
1	1.5	66
2	2.0	69

[a] General conditions: **1a** (0.8 mmol), **2a** (0.2 mmol) and NaOH (1.5 equiv) in solvent (DMSO 1.0 mL) irradiated by blue LEDs (425 nm, 3W×2) for 24 h under an argon atmosphere at 35 °C. [b] Yields were determined by ^{1}H NMR using nitromethane as internal standard.

2.5) Effects of co-solvent[a]

Entry	Conditions	Yield (%)[b]
1	DMSO	69
2	DMSO+DMF (50 μL)	69

[a] General conditions: **1a** (0.8 mmol), **2a** (0.2 mmol) and NaOH (2.0 equiv) in solvent (1.0 mL) irradiated by blue LEDs (425 nm, 3W×2) for 24 h under an argon atmosphere at 35 °C. [b] Yields were determined by ^{1}H NMR using nitromethane as internal standard.

2.6) Effects of temperature[a]

Entry	Conditions	Yield (%)^b
1	35 °C	69
2	15 °C	73

^{a} General conditions: **1a** (0.8 mmol), **2a** (0.2 mmol) and NaOH (2.0 equiv) in solvent [DMSO (1.0 mL) + DMF (50.0 μL)] irradiated by blue LEDs (425 nm, 3W^{x}2) for 24 h under an argon atmosphere. ^{b} Yields were determined by ^{1}H NMR using nitromethane as internal standard.

2.7) Effects of reaction time^a

Entry	Conditions	Yield (%)^b
1	12 h	70
2	24 h	73
3	36 h	69

^{a} General conditions: **1a** (0.8 mmol), **2a** (0.2 mmol) and NaOH (2.0 equiv) in solvent [DMSO (1.0 mL) + DMF (50.0 μL)] irradiated by blue LEDs (425 nm, 3W^{x}2) for 24 h under an argon atmosphere at 15 °C. ^{b} Yields were determined by ^{1}H NMR using nitromethane as internal standard.

2.8) Effects of air^a

Entry	Conditions	Yield (%)^b
1	air atmosphere	73
2	argon atmosphere	73

^{a} General conditions: **1a** (0.8 mmol), **2a** (0.2 mmol) and NaOH (2.0 equiv) in solvent [DMSO (1.0 mL) + DMF (50.0 μl)] irradiated by blue LEDs (425 nm, 3W^{x}2) for 24 h at 15 °C. ^{b} Yields were determined by ^{1}H NMR using nitromethane as internal standard.

3. General procedure for C–C coupling

In the glove box, NaOH (0.4 mmol 16.0 mg) was added into a test tube (20.0 mL) charged with a magnetic stir bar. Then, the tube was moved out of the glovebox, followed by the addition of DMSO (1.0 mL), DMF (50.0 μL), hydrazone (0.8 mmol) and alkyl halide (0.2 mmol), sealed and the mixture was irradiated by 425 nm 3W^{x}2 blue LED for 24 h under an air atmosphere at 15 °C. Brine (10.0 mL) was added to the reaction system. The mixture was extracted with EA (20.0 mL × 3), and the combined organic phase was dried over Na_2SO_4, filtered and concentrated. The product diphenylmethane (**3a**) was isolated by flash chromatography on silica gel with PE/EA (250/1(v/v)).

4. The promotion of light on the reaction

23% yield of **3a** was obtained in dark indicating that nucleophilic substitution process was possible. To further investigate this pathway, different substrates were reacted at high temperature in absence of light, obtaining very low yield of products (2% yield of **3v**, trace amounts of **3m**) (Scheme S1). We increased the reaction's temperature without light, and the yield of **3a** increased at first and then decreased. The highest yield (50%) was obtained when heated at 60 ºC, which was also lower than visible-light induced condition at 15 ºC (73%). Further increasing the temperature lowered the yield because of competitive Wolff-Kishner-Huang reaction of **1a** under basic conditions. For product **3v** and **3m**, when increasing the reaction temperature in dark, the yields were much lower than visible-light induced condition at 15 ºC (60% for **3v** and 50% for **3m**, respectively). These results indicated that light was important for this transformation. On the other hand, the DFT calculations indicated that free energy barrier of an intermolecular aromatic nucleophilic substitution was as high as 30.0 kcal mol^{-1} (see Figure S2 in SI). Therefore, in the absence of light, the experimental results can only obtain low to trace yields. These controlling experiments and DFT calculations results indicated that visible light has a great promoting effect on the reaction.

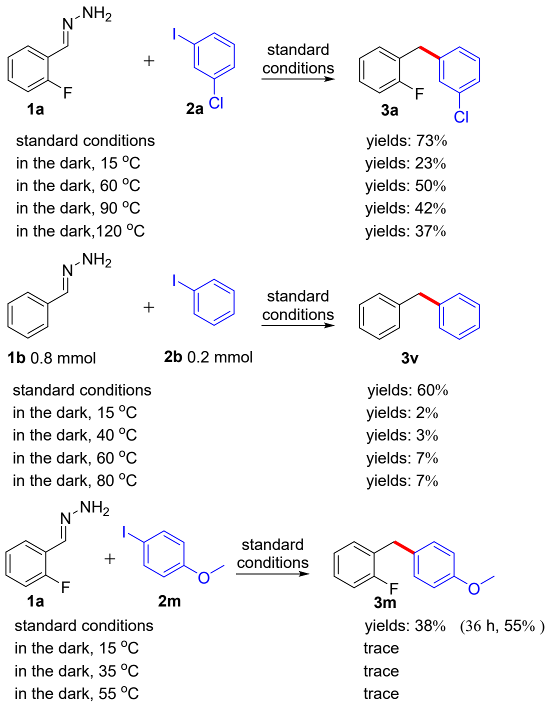

Scheme S1 Control experiments of different substrates under dark conditions.

5. Deuterium labelling experiment

We have carried out several deuterium labeling experiments, 0.4 mmol D_2O was added to the reaction system under the standard conditions (DMSO solvent), the yield was deceased to 44%. However, no deuterated product was detected. The same result was obtained using NaOD (40% in D_2O) instead of NaOH (Scheme S2a). Considering that the deuterium atom at the benzyl position may be exchanged with DMSO solvent under basic conditions leading to the above results, to further confirm this hypothesis, DMSO-*d6* (1.0 mL) was used as solvent instead of DMSO, and obtained the deuterated product **3v-*d*^{2}** (70% D) in 48% yield under standard conditions (Scheme S2b). Directly using diphenylmethane **3v** as starting material in DMSO-*d6* (1.0 mL) under basic conditions, the hydrogen atoms at the benzylic position were completely exchanged with/without light (Scheme S2c). These results can explain why no deuterated products were obtained when adding D_2O or using NaOD (due to the hydrogen/deuterium atoms at the benzyl position can easily exchange with DMSO solvent under basic condition). The similar results for *α*-trideuteration of methylarenes have been reported with DMSO-*d6* in the presence of NaOH (*Org. Chem. Front.*, **2021**, *8*, 2981).

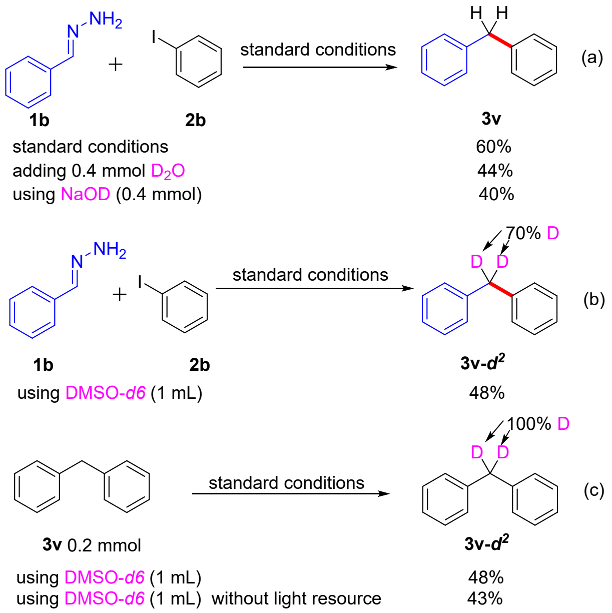

Scheme S2 Deuterium labeling experiments.

6. Scale-up experiment

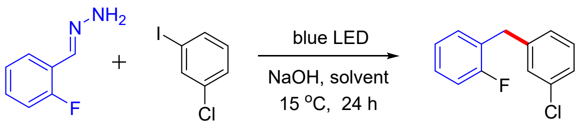

In glove box, NaOH (3.0 mmol, 120.0 mg) were added into a round bottom flask (25.0 mL) charged with a magnetic stir bar. Then, the flask was moved out of the glovebox, followed by the addition of DMSO (7.5 mL), DMF (0.35 mL), hydrazone **1a** (6.0 mmol) and aryl halide **2a** (1.5 mmol), sealed, and the mixture was irradiated by 425 nm blue LEDs (3W×4) for 24 h under an air atmosphere at 15 °C. Brine (10.0 mL) was added to the reaction system. The mixture was extracted with EA (30.0 mL × 3), and the combined organic phase was dried over Na_2SO_4, filtered and concentrated. The product **3a** (colorless liquid, 180 mg, 55% yield) was isolated by flash chromatography on silica gel with PE/EA (250/1 (v/v)).

7. Computational details

7.1 Complete reference for Gaussian 09

Frisch, M. J.; Trucks, G. W.; Schlegel, H. B.; Scuseria, G. E.; Robb, M. A.; Cheeseman, J. R.; Scalmani, G.; Barone, V.; Mennucci, B.; Petersson, G. A.; Nakatsuji, H.; Caricato, M.; Li, X.; Hratchian, H. P.; Izmaylov, A. F.; Bloino, J.; Zheng, G.; Sonnenberg, J. L.; Hada, M.; Ehara, M.; Toyota, K.; Fukuda, R.; Hasegawa, J.; Ishida, M.; Nakajima, T.; Honda, Y.; Kitao, O.; Nakai, H.; Vreven, T.; Montgomery, Jr., J. A.; Peralta, J. E.; Ogliaro, F.; Bearpark, M.; Heyd, J. J.; Brothers, E.; Kudin, K. N.; Staroverov, V. N.; Keith, T.; Kobayashi, R.; Normand, J.; Raghavachari, K.; Rendell, A.; Burant, J. C.; Iyengar, S. S.; Tomasi, J.; Cossi, M.; Rega, N.; Millam, J. M.; Klene, M.; Knox, J. E.; Cross, J. B.; Bakken, V.; Adamo, C.; Jaramillo, J.; Gomperts, R.; Stratmann, R. E.; Yazyev, O.; Austin, A. J.; Cammi, R.; Pomelli, C.; Ochterski, J. W.; Martin, R. L.; Morokuma, K.; Zakrzewski, V. G.; Voth, G. A.; Salvador, P.; Dannenberg, J. J.; Dapprich, S.; Daniels, A. D.; Farkas, O.; Foresman, J. B.; Ortiz, J. V.; Cioslowski, J.; and Fox, D. J. Gaussian 09, revision D.01; Gaussian, Inc.: Wallingford, CT, 2013.

7.2 Computational methods.

All DFT calculations were carried out using Gaussian 09 program. All geometry optimizations and frequency calculations in this paper were performed with the B3LYP

functional[1, 2] including Grimme's D3 (BJ-damping) dispersion corrections[3] in implicit dimethylsulfoxide using at 6-31G(d) basis set (SDD basis set[4] for I and Na) by using the Solvation Model Based on Density (SMD)[5] with the keyword in the Gaussian code route section "SCRF=(SMD,Solvent=dimethylsulfoxide). The vibrational frequencies were computed at the same level of theory as for the geometry optimizations to confirm whether each optimized structure is an energy minimum or a transition state and to evaluate the zero-point vibrational energy (ZPVE) and thermal corrections. The single-point energies were computed with the M062X functional[6] including Grimme's D3 dispersion corrections[7] using a higher-level basis set 6-311+G(d,p) basis set (SDD basis set for I and Na). The TDDFT/B3LYP-D3(BJ)/6-31G(d)-SDD/SMD(DMSO) method was applied to optimize the geometry of the lowest singlet excited state. The frontier molecular orbital (FMO) analyses were generated using VMD[8] and Multiwfn[9]. The 3D diagrams of molecules were generated using CYLView [10].

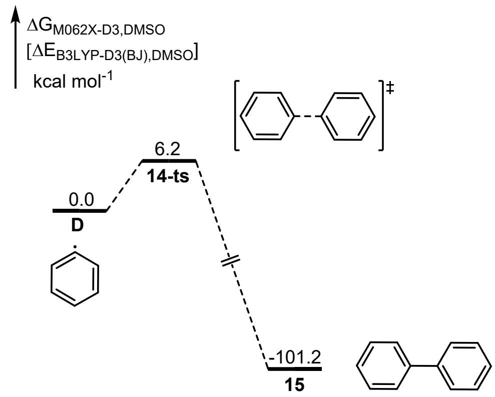

Figure S1. Free energy profile for the homo-coupling of phenyl radical **D**. The energy values are in kcal mol^{-1} and represent the relative free energies calculated at M062X-D3/6-311+G(d,p)-SDD/SMD(DMSO)//B3LYP-D3(BJ)/6-31G(d)-SDD/SMD(DMSO) level of theory in DMSO solvent.

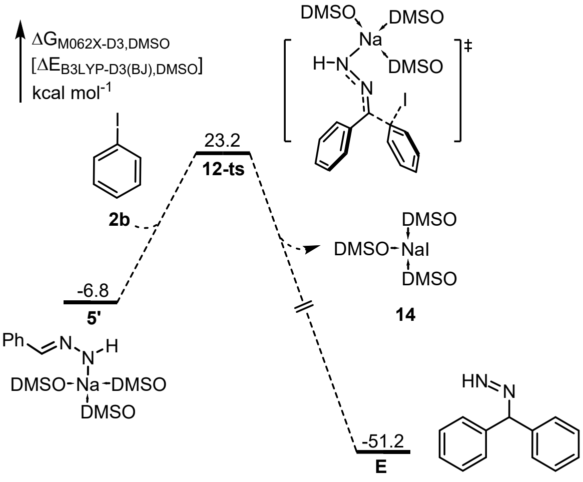

Figure S2. Free energy profile for the nucleophilic substitution of benzylidenehydrazinide **5'** with iodobenzene **2b**. The energy values are in kcal mol^{-1} and represent the relative free energies calculated at M062X-D3/6-311+G(d,p)-SDD/SMD(DMSO)//B3LYP-D3(BJ)/6-31G(d)-SDD/SMD(DMSO) level of theory in DMSO solvent.

7.3 DFT calculations for the radical substitution (SRN)

As shown in Figure S3, when hydrazone radical **C** is formed, the calculated activation free energy for the direct radical substitution between hydrazone radical and iodobenzene **2b** via **16-ts** is 31.4 kcal mol^{-1}, leading to the iodine radical and benzhydryldiazene intermediate **E**. Moreover, the calculated activation free energy for the radical substitution between **7** and iodobenzene **2b** through transition state **17-ts** is as high as 28.1 kcal mol^{-1}. The activation energies of these two pathways are much higher than the corresponding radical coupling pathway via transition states **8-ts**. Therefore, the radical coupling of **C** with **D** is a favorable pathway due to the low energy barriers.

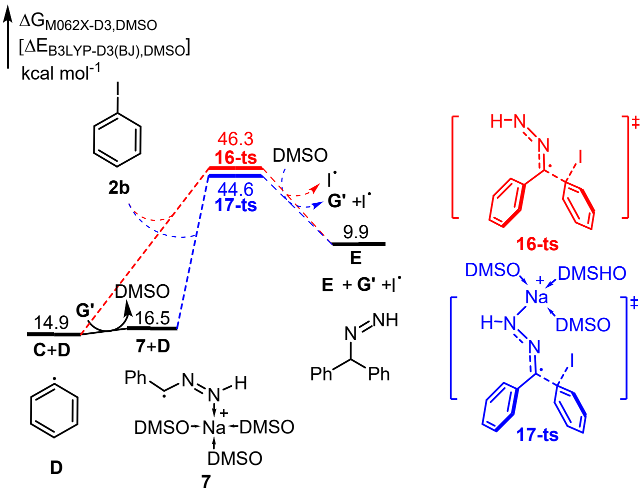

Figure S3. Free energy profiles for the radical substitution of hydrazone radical **C** with iodobenzene **2b**. The energy values are in kcal mol^{-1} and represent the relative free energies calculated at M062X-D3/6-311+G(d,p)-SDD/SMD(DMSO)//B3LYP-D3(BJ)/6-31G(d)-SDD/SMD(DMSO) level of theory in DMSO solvent.

7.4 Computational Methods Thermal correction to Gibbs free energy, thermal correction to enthalpy, electronic energies and Gibbs free energies (in Hartree) of structures calculated at the M062X-D3/6-311+G(d,p)-SDD/SMD(DMSO)//B3LYP-D3(BJ)/6-31G(d)-SDD/SMD(DMSO) level of theory.

Geometry	Thermal Correction to Free Energy	Thermal Correction to Enthalpy	Electronic Energy	Gibbs Free Energy	Imaginary
**1b**	0.107356	0.148421	-380.973558	-380.866202	
**2'**	0.201158	0.281080	-1897.670502	-1897.469344	
**3'**	0.330408	0.431418	-2278.667380	-2278.336972	
**4-ts**	0.325604	0.426613	-2278.667549	-3439.125812	977.12*i*
**5'**	0.308207	0.404252	-2202.228786	-2201.920579	
**DMSO**	0.051181	0.086338	-553.163715	-553.112534	
**A**	0.093210	0.133651	-380.466042	-380.372832	
**G'**	0.268002	0.356072	-2374.920338	-2374.652336	
**6_{S0}**	0.171217	0.232830	-623.447594	-623.276377	
**6_{T1}**	0.165063	0.229806	-623.378845	-623.213782	
**2b**	0.058570	0.097170	-242.970170	-242.9116	
**I^-**	-0.016848	0.002360	-11.552529	-11.569377	
**C**	0.092795	0.134204	-380.321994	-380.229199	
**D**	0.059711	0.093067	-231.518774	-231.459063	
**7**	0.307309	0.405147	-2202.073839	-2201.76653	
**8-ts**	0.388530	0.498859	-2433.609169	-2433.220639	58.24*i*
**E**	0.179849	0.233718	-611.970957	-611.791108	
**9-ts**	0.398173	0.511766	-2509.666477	-2509.268304	898.28*i*
**H_2O**	0.003229	0.024684	-76.429043	-76.425814	
**N_2**	-0.012839	0.008914	-109.515412	-109.528251	
**F**	0.159156	0.207072	-502.009480	-501.850324	
**10-ts**	0.176103	0.229589	-578.424297	-578.248194	799.43*i*

**3v**	0.172626	0.221967	-502.530658	-502.358032	
**11-ts**	0.170310	0.228163	-611.849302	-611.678992	55.31*i*
**I^-**	0.180266	0.234024	-611.982206	-611.80194	
**12-ts**	0.388358	0.502151	-2445.172618	-2444.78426	340.12*i*
**13**	0.187618	0.270773	-1833.299513	-1833.111895	
**14-ts**	0.130306	0.186079	-463.038477	-462.908171	21.81*i*
**15**	0.148688	0.192330	-463.228156	-463.079468	
**16-ts**	0.389922	0.502888	-623.263605	-623.090748	599.48*i*
**17-ts**	0.172857	0.232233	-2445.023281	-2444.633359	605.71*i*
**$I^{\cdot}$**	-0.017503	0.002360	-11.340252	-11.357755	

7.5 B3LYP-D3(BJ) geometries for all the optimized compounds and transition states.

1b

C	-2.78196200	-0.26500400	0.00981100
C	-1.87785800	-1.33436700	0.00089700
C	-0.50688900	-1.09725600	-0.00889800
C	-0.01056500	0.21992500	-0.00867000
C	-0.92543600	1.28605400	-0.00032700
C	-2.29982900	1.04536800	0.00890300
H	-3.85166800	-0.45440000	0.01656200
H	-2.24669100	-2.35657300	0.00057100
H	0.19463900	-1.92555100	-0.01694400
H	-0.55203000	2.30718300	-0.00096200
H	-2.99343400	1.88178100	0.01508300
C	1.42506500	0.51234400	-0.01295800
N	2.30798700	-0.42195900	0.00397000
H	1.71722600	1.56986600	-0.03023400
N	3.62890800	-0.05879300	-0.08423900
H	3.82947200	0.88515600	0.25599600
H	4.20906200	-0.74458900	0.38925600

1a

C	3.39665500	-1.67333400	0.69753600
C	2.20312100	-0.94818600	0.65722700
C	2.15847400	0.30994500	0.03379000
C	3.33411000	0.83381200	-0.53094600
C	4.51965000	0.10201200	-0.50579000
C	4.55319600	-1.15487200	0.10920800
H	3.42370400	-2.64564600	1.19532800
H	1.31110600	-1.35293800	1.13732400
C	0.93091900	1.17538500	0.02443800
H	5.42412900	0.51352300	-0.96047200
H	3.29688600	1.82430600	-0.98785000
O	1.05444200	2.39217200	0.05561500
C	-0.42575900	0.55585400	-0.02010000
C	-1.51459300	1.32552500	0.42047800
C	-0.68664600	-0.72399800	-0.54976300
C	-2.81823900	0.84005800	0.37373300
H	-1.31655500	2.32615900	0.80881600
C	-1.98315500	-1.21562400	-0.61774600
H	0.12967700	-1.33207000	-0.94132600
C	-3.06201200	-0.44283500	-0.14984500
H	-3.63539400	1.46088800	0.73973100
H	-2.19459400	-2.19959400	-1.04042800
O	-4.28367800	-1.00372200	-0.24771700
C	-5.41969600	-0.26806300	0.16515200
H	-5.53012500	0.66826400	-0.40894700
H	-6.29125000	-0.90783800	-0.02737900
H	-5.38395500	-0.02981300	1.24258100
H	5.48411700	-1.72690800	0.13520000

2’

O	-0.93738200	-0.83710900	-1.88180700
H	-0.82308200	-1.28230400	-2.73372200
Na	-0.09422800	0.28600500	-0.27548800
S	0.59192300	-2.78395400	0.76913200
O	0.66673800	-1.30678300	1.22336300
C	-1.11183000	-3.06038100	0.19533000
H	-1.19894400	-4.10004200	-0.13911400
H	-1.77389800	-2.89519300	1.05022700
H	-1.29045700	-2.33833900	-0.62830100
C	1.36288700	-2.84661800	-0.87847500
H	2.41699100	-2.57974200	-0.76854000
H	1.27988900	-3.86643900	-1.26815000
H	0.81462200	-2.12930500	-1.50711000
S	-3.13745100	1.54301800	-0.19108500
O	-1.85126200	1.40309500	0.64751000
C	-3.62345800	-0.12795500	-0.69391100
H	-4.54841100	-0.06004900	-1.27717200
H	-2.77701700	-0.50800100	-1.30253500
H	-3.77665100	-0.74721700	0.19671500
C	-4.47347600	1.86088900	0.99967000

H	-4.29678400	2.84027100	1.45245100
H	-5.42899300	1.86786300	0.46638000
H	-4.46451000	1.08131000	1.76748600
S	2.90479900	1.71192700	0.39786400
O	1.69278400	1.64862600	-0.54896900
C	3.58361300	0.03112800	0.49383200
H	4.49040600	0.04815900	1.10661200
H	2.81506400	-0.59041300	0.96134600
H	3.80672700	-0.32882400	-0.51527500
C	4.25318200	2.45018800	-0.56783700
H	3.98800000	3.49046100	-0.77364400
H	5.17544400	2.41370300	0.01998300
H	4.36724300	1.89606100	-1.50427700

3’

C	-4.92680200	-1.48439700	-0.47832100
C	-5.19342800	-0.22496200	0.07314500
C	-4.31832400	0.83732900	-0.13728200
C	-3.15051400	0.66460000	-0.90637100
C	-2.90084900	-0.60069100	-1.46674200
C	-3.77729000	-1.66400700	-1.25170000
H	-5.60923200	-2.31245200	-0.30793800
H	-6.08817500	-0.07323100	0.67156300
H	-4.52519000	1.81110100	0.29565000
H	-1.98906100	-0.75392300	-2.03504700
H	-3.55787000	-2.63704900	-1.68392600
C	-2.17900200	1.73789300	-1.11454600
N	-2.29600100	2.88591400	-0.53117800
H	-1.32729500	1.51862000	-1.76715300
N	-1.35414700	3.83502100	-0.80121400
H	-1.38014300	4.54323200	-0.07291800
O	1.03677900	2.67149200	-1.12456800
H	0.90751300	2.26807000	-2.00004800
Na	0.71714000	1.01576400	0.29380100
H	-0.36683500	3.43912700	-0.97536400
S	4.11366100	0.93031500	0.38722000
O	2.82514000	0.18550100	0.79090900
C	5.36418500	-0.35754200	0.10812900
H	6.27977700	0.11245400	-0.26376300
H	5.56196500	-0.84486200	1.06656400
H	4.98150500	-1.08353700	-0.61542800
C	3.90326200	1.45799700	-1.33474000
H	2.99445200	2.08513600	-1.34826400
H	4.79032600	2.02445200	-1.63732800
H	3.77230100	0.58003300	-1.97515700
S	-0.84678600	-0.29256600	2.99708500
O	-0.41478200	0.99064700	2.26409500
C	-1.92923400	-1.21720900	1.86632500
H	-2.19373700	-2.16802000	2.34012500
H	-2.83041100	-0.62015300	1.71367000
H	-1.40476900	-1.37872500	0.92109000
C	0.56352700	-1.43999300	2.93152300
H	1.37745500	-1.00028100	3.51274500
H	0.26183300	-2.39274100	3.37788300
H	0.86727700	-1.57495600	1.89142700
S	1.35677300	-1.64732600	-1.45978800
O	0.23525000	-1.01970600	-0.61090700
C	2.24643200	-2.81182700	-0.38936300
H	2.99970600	-3.33326000	-0.98827800
H	2.72635500	-2.20721900	0.38187600
H	1.54159600	-3.52409400	0.04983900
C	0.52382300	-2.87347100	-2.50912500
H	-0.09042100	-2.33039000	-3.23192000
H	1.27649800	-3.46719700	-3.03662300
H	-0.10510400	-3.51264200	-1.88247000

4-ts

C	-5.00196800	-1.38591800	-0.50103100
C	-5.17881300	-0.15140700	0.13650100
C	-4.26236900	0.88047400	-0.04741500
C	-3.13313300	0.70968400	-0.87843900
C	-2.97401600	-0.53560800	-1.52062200
C	-3.89250700	-1.56698000	-1.33228800
H	-5.71783500	-2.18974300	-0.35323200
H	-6.03933100	0.00630100	0.78247700
H	-4.40709900	1.83370700	0.45177000
H	-2.09754800	-0.69703300	-2.14095500
H	-3.73835000	-2.51958300	-1.83379000
C	-2.13477400	1.75085900	-1.07715400
N	-2.18721400	2.88803600	-0.43964200
H	-1.32109500	1.53614800	-1.77996300
N	-1.23206000	3.80034900	-0.68504400
H	-1.33778100	4.55297100	-0.00605200
O	1.02057900	2.75464700	-0.94889200
H	1.02257000	2.39574400	-1.85246500
Na	0.63947500	0.98662000	0.37579100
H	-0.05177500	3.32547200	-0.86721400
S	4.09817000	0.86132800	0.51454700
O	2.75411900	0.16890300	0.81052800
C	5.27414700	-0.47259500	0.14128300
H	6.23253800	-0.02907100	-0.14569900
H	5.40000100	-1.06864400	1.04874500
H	4.87845600	-1.09177600	-0.66846500
C	3.98160300	1.54844000	-1.16083100
H	3.17077100	2.28477400	-1.13815700
H	4.93551900	2.02078500	-1.41596400
H	3.74329100	0.74803800	-1.86748200
S	-0.86841800	-0.51222400	2.98616400
O	-0.46120300	0.82766100	2.34522200
C	-1.91695300	-1.38619400	1.78556900
H	-2.14372300	-2.37938000	2.18640300
H	-2.84040200	-0.81392100	1.67525100
H	-1.38742400	-1.45912000	0.83171000
C	0.56981500	-1.61940000	2.86022500
H	1.37036800	-1.19519600	3.47071100
H	0.28846000	-2.60325600	3.24852900
H	0.88361400	-1.68737200	1.81654300
S	1.39565700	-1.48637700	-1.57595100
O	0.22517100	-0.95444400	-0.72766700
C	2.22406600	-2.76755400	-0.59267000
H	2.99380800	-3.24177400	-1.20938300
H	2.67954400	-2.25650500	0.25654200
H	1.48874900	-3.50574700	-0.25926200
C	0.63284300	-2.58088500	-2.80757700
H	0.04841800	-1.95592100	-3.48768000
H	1.42094700	-3.09619700	-3.36525500
H	-0.01749600	-3.30047900	-2.30156500

H_2O

O	0.00000000	0.00000000	0.12075400
H	0.00000000	-0.76055800	-0.48301800
H	0.00000000	0.76055800	-0.48301800

5’

C	-6.09785000	0.27716900	-0.52095200
C	-5.60416200	-0.07751600	0.74124400
C	-4.34250100	0.33609000	1.15723900

C	-3.51661000	1.12578700	0.31990000
C	-4.03393900	1.47741100	-0.94882000
C	-5.29790600	1.06053400	-1.35983200
H	-7.08273200	-0.05035100	-0.84203400
H	-6.21175300	-0.68523100	1.40841800
H	-3.97329900	0.05319800	2.13799900
H	-3.42176000	2.08117100	-1.61603100
H	-5.66146000	1.34810000	-2.34387300
C	-2.18755600	1.55625300	0.70001200
N	-1.65064100	1.23806200	1.85812800
H	-1.61477500	2.14967000	-0.02807000
N	-0.39756700	1.61350100	2.10797200
H	-0.19798000	1.27738600	3.05400600
Na	0.93658300	0.85112900	0.34303100
S	-1.41143000	-1.39244100	-0.87118900
O	-0.02659300	-0.71760200	-0.94119500
C	-1.18646700	-3.02091900	-1.64791700
H	-2.12018500	-3.58509500	-1.56065100
H	-0.95550100	-2.85278300	-2.70301000
H	-0.36610200	-3.55520700	-1.16051800
C	-1.62381200	-1.98334500	0.83289100
H	-1.69033400	-1.10205200	1.47421400
H	-2.55073900	-2.56148600	0.89084800
H	-0.76478800	-2.60033600	1.11151300
S	3.54554600	2.59108600	-0.91127600
O	2.01583800	2.42118900	-0.86859700
C	4.13379800	2.48511900	0.80728600
H	5.22709100	2.53641800	0.81023000
H	3.72435100	3.34124700	1.34989400
H	3.79346400	1.54414100	1.24978600
C	4.23005800	1.00077400	-1.46620200
H	3.84855600	0.81215700	-2.47320400
H	5.32200800	1.07371500	-1.49074000
H	3.90141000	0.22426600	-0.77157500
S	2.34960400	-2.09330700	0.69852800
O	2.59919800	-0.59037000	0.95119500
C	2.70597600	-2.41126500	-1.05405100
H	2.60529800	-3.48585900	-1.23711300
H	1.95590100	-1.86215900	-1.62576100
H	3.71716500	-2.07581400	-1.30001100
C	3.80026300	-2.93446500	1.39385600
H	3.77922800	-2.78496900	2.47653300
H	3.73836700	-4.00319000	1.16714700
H	4.71080100	-2.50121500	0.96982400

DMSO

S	-0.00002900	0.23406700	-0.43778100
O	-0.00041200	1.51867100	0.38034700
C	1.35591300	-0.81673300	0.18212800
H	1.31940900	-1.78747500	-0.32194900
H	2.29826100	-0.31545100	-0.05543700
H	1.26133600	-0.94092900	1.26550200
C	-1.35556500	-0.81735200	0.18208200
H	-2.29826000	-0.31741900	-0.05687600
H	-1.31755600	-1.78881200	-0.32048800
H	-1.26152500	-0.93984900	1.26569700

G’

Na	-0.00019800	0.00026600	-0.77568700
S	-2.43882400	-1.69966700	0.63990800
O	-1.01391900	-1.82812700	0.06022700
C	-3.58121900	-1.78076900	-0.76546400
H	-4.60772600	-1.79924900	-0.38644600
H	-3.40949400	-0.87766300	-1.35412400
H	-3.37041600	-2.67761500	-1.35615600
C	-2.81750600	-3.32348800	1.35462100
H	-2.13939400	-3.47898400	2.19771300
H	-3.85300100	-3.32276100	1.70798400
H	-2.66599800	-4.09998900	0.59919000
S	2.43944000	1.69989500	0.64023000
O	1.01416800	1.82750100	0.06132600
C	3.58102400	1.78059900	-0.76586600
H	4.60770800	1.80011200	-0.38738000
H	3.40961800	0.87687200	-1.35366200
H	3.36926800	2.67680100	-1.35719500
C	2.81800000	3.32425500	1.35372100
H	2.14037400	3.48001900	2.19715800
H	3.85371100	3.32405800	1.70646800
H	2.66582100	4.10031400	0.59797300
S	2.47885900	-2.30325800	-0.59666300
O	1.95530900	-1.00890200	-1.25488300
C	4.26263000	-2.04898800	-0.36793300
H	4.67578100	-2.91198000	0.16285400
H	4.71713400	-1.97109300	-1.35883000
H	4.43101700	-1.13039200	0.20094000
C	2.00633000	-2.23577400	1.15545500
H	0.91402800	-2.26765700	1.18868200
H	2.42665800	-3.10812300	1.66570100
H	2.38101100	-1.31096300	1.60410400
S	-2.47930500	2.30316600	-0.59667500
O	-1.95525600	1.00914800	-1.25516300
C	-4.26291900	2.04817500	-0.36779100
H	-4.67629700	2.91103700	0.16304100
H	-4.71756900	1.97015900	-1.35861600
H	-4.43099000	1.12953700	0.20109900
C	-2.00661100	2.23575800	1.15539900
H	-0.91432000	2.26773300	1.18862500
H	-2.38126300	1.31100800	1.60417700
H	-2.42698200	3.10815800	1.66554000

A

C	2.72859900	0.31587000	0.00001100
C	1.78104300	1.35044600	0.00000300
C	0.41834300	1.07661700	-0.00000200
C	-0.07120400	-0.25864600	0.00000100
C	0.90550700	-1.28984900	0.00000900
C	2.26909600	-1.00667800	0.00001400
H	3.79262500	0.53568700	0.00001500
H	2.11393000	2.38677000	0.00000100
H	-0.30219900	1.88925400	-0.00000800
H	0.57200500	-2.32619900	0.00001100
H	2.98292900	-1.82819400	0.00002000
C	-1.47322600	-0.57824300	-0.00000400
N	-2.40827000	0.36572200	-0.00001200
H	-1.76614800	-1.63683600	-0.00000200
N	-3.66833600	-0.01457100	-0.00001600
H	-4.20585300	0.86435700	-0.00002200

2b

C	-3.36286100	-0.00000200	-0.00000200
C	-2.66318500	-1.20831200	-0.00000200
C	-1.26518100	-1.21790100	-0.00000100
C	-0.58753500	0.00000500	0.00000000
C	-1.26518400	1.21790200	-0.00000100
C	-2.66319300	1.20830700	-0.00000100
H	-4.44889200	-0.00000800	-0.00000300
H	-3.20046200	-2.15245800	-0.00000200
H	-0.72279600	-2.15682500	-0.00000100

H	-0.72281100	2.15683300	0.00000000
H	-3.20046700	2.15245600	-0.00000200
I	1.56864600	0.00000000	0.00000100

6_{S0}

C	0.51743500	-2.90575500	0.01688500
C	0.50408500	-2.02139300	-1.07195600
C	1.48668200	-1.05101000	-1.21986000
C	2.54509300	-0.91475400	-0.28004500
C	2.54632800	-1.82904400	0.80667900
C	1.55565200	-2.79688900	0.95001500
H	-0.25566600	-3.66087400	0.12909100
H	-0.29174700	-2.08672500	-1.81072900
H	1.45417900	-0.36647700	-2.06199100
H	3.33897500	-1.75765200	1.54941100
H	1.59053300	-3.47224800	1.80273100
C	3.56141100	0.09569700	-0.38730700
N	3.55094400	0.99896800	-1.36179500
H	4.34615200	0.12791600	0.38045000
N	4.50357100	1.90722400	-1.36989900
C	1.20962400	1.65139900	1.88282300
C	0.79172900	2.28657700	0.71158200
C	-0.36496800	1.86303400	0.05136000
C	-1.08586800	0.79583400	0.58520700
C	-0.68234100	0.13961600	1.74526000
C	0.47641800	0.57926700	2.39369300
H	2.11411400	1.98059700	2.38533500
H	1.37209000	3.10558200	0.29656500
H	-0.68781200	2.35277900	-0.86046800
H	-1.24612100	-0.69894500	2.13720600
H	0.80448000	0.07200800	3.29621500
I	-2.86018800	0.11729100	-0.43456400
H	4.30148900	2.48881100	-2.19609100

6_{T1}

C	-0.11645000	2.90904800	0.30024800
C	-0.33329900	2.30797300	-0.95538000
C	-1.39613400	1.44707300	-1.16514400
C	-2.31514800	1.13314200	-0.09568300
C	-2.08061100	1.76807900	1.17062700
C	-1.01562100	2.62431100	1.36027100
H	0.71962200	3.58545300	0.45248900
H	0.35094500	2.52055600	-1.77472800
H	-1.55479200	0.98370400	-2.13349200
H	-2.75642000	1.54741900	1.99511900
H	-0.86051700	3.08295000	2.33474000
C	-3.35264800	0.20400700	-0.26680300
N	-3.59932000	-0.45846000	-1.44040600
H	-4.00124300	-0.00875100	0.59120900
N	-4.54583500	-1.44306300	-1.20912700
C	-1.43683500	-2.03443000	1.40419700
C	-1.07374800	-2.27499400	0.07712600
C	0.11111900	-1.74660900	-0.43891000
C	0.92372100	-0.98267800	0.40084300
C	0.58245400	-0.72896500	1.72634300
C	-0.60919300	-1.26562100	2.22526900
H	-2.36698700	-2.43802000	1.79264500
H	-1.72797500	-2.85061300	-0.57024000
H	0.38944300	-1.92472900	-1.47143600
H	1.21963300	-0.12270600	2.36018400
H	-0.88850500	-1.06969200	3.25659800
I	2.75269900	-0.15706700	-0.38861700
H	-4.72579500	-1.83237900	-2.14767900

C

C	2.69763800	0.29536000	0.00001100
C	1.77674500	1.35291500	0.00000300
C	0.41341000	1.09447200	-0.00000200
C	-0.05897200	-0.24054500	0.00000100
C	0.88059200	-1.29739500	0.00000900
C	2.24448600	-1.02874700	0.00001300
H	3.76349100	0.50421400	0.00001400
H	2.13173700	2.37937100	0.00000100
H	-0.30511600	1.90681800	-0.00000700
H	0.52258400	-2.32321500	0.00001100
H	2.95770000	-1.84778100	0.00001900
C	-1.45859900	-0.55185700	-0.00000400
N	-2.39274300	0.40901300	-0.00001200
H	-1.77102400	-1.59905700	-0.00000100
N	-3.61395000	-0.05744100	-0.00001700
H	-4.22431300	0.77342300	-0.00002300

D

C	0.00000000	-1.32474800	0.00000200
C	-1.21522400	-0.63280500	0.00000100
C	-1.22789200	0.77250200	-0.00000100
C	0.00000100	1.39914100	-0.00000200
C	1.22789100	0.77250200	-0.00000100
C	1.21522300	-0.63280700	0.00000000
H	-0.00000100	-2.41106500	0.00000300
H	-2.15571000	-1.17823600	0.00000300
H	-2.16537700	1.32241100	-0.00000100
H	2.16537900	1.32240800	-0.00000200
H	2.15571100	-1.17823400	0.00000100

7

C	-5.74526300	-1.51653600	-0.27101400
C	-5.60865000	-0.31790300	-0.98637900
C	-4.35014000	0.14445200	-1.34230500
C	-3.19490900	-0.59067400	-0.98067000
C	-3.34748300	-1.80171500	-0.26638600
C	-4.61230200	-2.25726300	0.08460300
H	-6.73368000	-1.87146800	0.00572900
H	-6.49163100	0.25089400	-1.26226900
H	-4.23560800	1.07018600	-1.89529200
H	-2.46359300	-2.37220400	-0.00034500
H	-4.72060200	-3.18779100	0.63357400
C	-1.87225300	-0.13795800	-1.29748000
N	-1.67316300	0.96534300	-2.02723700
H	-1.01427900	-0.70922500	-0.93205500
N	-0.41957100	1.31507600	-2.15163400
H	-0.40275800	2.11188900	-2.79931700
Na	1.42056500	0.80375300	-0.70572900
S	0.88829200	-1.20765600	1.93297900
O	0.94517500	-1.09712100	0.39164100
C	1.77567300	-2.73701200	2.33781200
H	2.82975000	-2.58221200	2.09403600
H	1.66986500	-2.93575700	3.40851700
H	1.36454600	-3.55993400	1.74635900
C	-0.78330100	-1.78116400	2.34116500
H	-0.83649000	-1.95142900	3.42101100
H	-1.48600700	-0.99580200	2.05528200
H	-0.99622900	-2.70389000	1.79544200
S	4.28677000	-0.72549900	-1.60598700
O	3.30240400	0.43471600	-1.85119400
C	4.62478800	-0.74196000	0.17958000
H	5.25127300	-1.60633200	0.41957100

H	5.15886000	0.18037200	0.42298200
H	3.67903700	-0.78658700	0.72356600
C	3.31614200	-2.26207400	-1.67153200
H	2.93904600	-2.36446500	-2.69285900
H	3.97500900	-3.10401600	-1.43724000
H	2.48747000	-2.19985900	-0.95905600
S	0.30588000	3.18609900	1.53359900
O	1.44096200	2.37241500	0.88738000
C	-0.53020400	4.08058300	0.19039100
H	-1.36385300	4.64984500	0.61284200
H	0.19895600	4.76595400	-0.24914200
H	-0.88876200	3.37172600	-0.56053800
C	-1.03948200	2.01998900	1.90209100
H	-0.69748100	1.36418000	2.70590400
H	-1.91292100	2.58553300	2.24157300
H	-1.28609300	1.44441600	1.00550700

8-ts

C	-5.40725600	1.41117100	0.98389300
C	-5.21446900	1.61110800	-0.38988500
C	-3.93298200	1.61308700	-0.92380700
C	-2.81342800	1.41273300	-0.08328600
C	-3.02107100	1.21147600	1.29935200
C	-4.30701900	1.21269500	1.82573400
H	-6.41256700	1.40855800	1.39481700
H	-6.07067800	1.76211300	-1.04064500
H	-3.77621300	1.76061400	-1.98663600
H	-2.16365400	1.04838900	1.94420400
H	-4.45714000	1.05509300	2.88961800
C	-1.47572800	1.34525300	-0.59994300
N	-1.21980800	1.58078100	-1.89554900
H	-0.65622700	1.12016400	0.08557500
N	-0.00119600	1.31946900	-2.27134100
H	0.08065900	1.63085000	-3.24630000
C	-3.85875700	-2.74254600	-0.61989800
C	-3.41191200	-2.29740400	-1.86824600
C	-2.16982800	-1.65065100	-1.98362300
C	-1.45086800	-1.49563100	-0.81915800
C	-1.83357200	-1.91420700	0.43588400
C	-3.07766400	-2.55901500	0.52590500
H	-4.82423500	-3.23373300	-0.53886700
H	-4.02691900	-2.44071400	-2.75313900
H	-1.81991800	-1.27238000	-2.94006100
H	-1.21654000	-1.73396300	1.30944000
H	-3.43415000	-2.90647000	1.49247100
Na	1.57513700	0.00052100	-1.01838500
S	1.65967500	0.14952700	2.41526100
O	0.98298200	-0.35531100	1.12201200
C	1.83059600	-1.31201000	3.47578900
H	2.56085400	-1.97782400	3.00921600
H	2.19525400	-0.99429700	4.45729600
H	0.86158000	-1.81123500	3.56524200
C	0.35930200	0.98674700	3.36330900
H	0.76989900	1.28171700	4.33392900
H	0.06227700	1.87666500	2.80364800
H	-0.49141500	0.31204400	3.49276600
S	2.70448200	-3.14932300	-1.01766000
O	2.16358900	-1.98827400	-1.87569800
C	3.91805300	-2.41879600	0.12264400
H	4.27478400	-3.18956300	0.81218900
H	4.75196000	-2.04691200	-0.47823000
H	3.45255500	-1.59624000	0.67063100
C	1.42768300	-3.52850900	0.21879200
H	0.57666600	-3.96185000	-0.31178300
H	1.83040800	-4.25920000	0.92731000
H	1.13572000	-2.60498200	0.72489000
S	3.32375000	2.92846900	-0.49654200
O	3.30141400	1.40052400	-0.66930900
C	2.63954900	3.64481500	-2.02129200
H	2.62781200	4.73464900	-1.92345300
H	3.30007200	3.35567100	-2.84303300
H	1.63058300	3.25833100	-2.19015100
C	1.93814200	3.37164300	0.59669300
H	2.18079400	3.01824300	1.60130500
H	1.83983500	4.46158500	0.61031400
H	1.01544400	2.91113900	0.23178800

E

C	3.71006500	-1.04761600	0.31450100
C	3.20817200	-0.01107100	1.10320900
C	2.01625800	0.62681400	0.75009800
C	1.31562200	0.23381400	-0.39622300
C	1.82704900	-0.80395100	-1.18560200
C	3.01512300	-1.44320300	-0.83236300
H	4.63660700	-1.54378000	0.58956300
H	3.74249600	0.30400200	1.99528200
H	1.63201800	1.43315700	1.36701400
H	1.28580900	-1.11351200	-2.07612600
H	3.40069800	-2.24742200	-1.45297700
C	-0.01181700	0.87118500	-0.77309400
N	0.03289000	2.30618000	-0.36559600
H	-0.05431700	0.92759400	-1.86794300
N	-0.89534800	2.66995000	0.37660700
H	-0.73634000	3.67513800	0.56845900
C	-3.48277100	-1.32556400	0.58442500
C	-3.42525400	-0.82649500	-0.71819900
C	-2.29656500	-0.12484100	-1.14801400
C	-1.21267600	0.08118400	-0.28747300
C	-1.27753600	-0.42470100	1.01725200
C	-2.40532900	-1.12075900	1.45156600
H	-4.35864500	-1.87282200	0.92190100
H	-4.25572700	-0.98439300	-1.40089100
H	-2.25396500	0.26466000	-2.16228300
H	-0.44101000	-0.27762900	1.69375400
H	-2.44247400	-1.50669400	2.46668100

9-ts

C	-4.42148900	-4.22770200	0.20181800
C	-3.23572100	-4.15792200	0.93796300
C	-2.47208100	-2.99012300	0.92237400
C	-2.87583600	-1.86849100	0.18196100
C	-4.06174400	-1.95589700	-0.56151700
C	-4.82951100	-3.12188100	-0.54850900
H	-5.02113000	-5.13383200	0.21324800
H	-2.90749700	-5.01107200	1.52612200
H	-1.54929700	-2.93895900	1.49635700
H	-4.39728600	-1.10250700	-1.14281400
H	-5.74979100	-3.16639900	-1.12535600
C	-1.96772400	-0.67452400	0.12560900
N	-0.94809700	-0.70181100	-1.05092600
N	-0.98797700	-1.62177500	-1.86761800
H	0.09188300	-1.33187300	-2.59490400
C	-3.88552400	3.20051200	0.04398900
C	-3.73242700	2.47103300	-1.13723400
C	-3.11538900	1.21911200	-1.11412100
C	-2.65147000	0.67071400	0.09026200
C	-2.80854200	1.41272100	1.26820500
C	-3.42102300	2.66690900	1.24909000

H	-4.36110400	4.17737800	0.02604400
H	-4.08683900	2.87998300	-2.07986400
H	-2.98057900	0.66658100	-2.03912000
H	-2.44132400	1.00303400	2.20595800
H	-3.53261800	3.22804500	2.17322100
O	1.15831700	-0.96355800	-3.00911800
H	0.97059400	-0.38438900	-3.76544200
H	-1.29676400	-0.69415800	0.98949900
Na	1.31179600	0.26513400	-1.06662100
S	4.53088500	-0.84256100	-1.39675500
O	3.61108900	0.21874800	-0.76428200
C	5.65131100	-1.38424200	-0.07126900
H	6.26722000	-2.20849000	-0.44333500
H	6.28905500	-0.53685400	0.19374600
H	5.06514700	-1.70289800	0.79514200
C	3.55875200	-2.36877400	-1.53973000
H	2.73254500	-2.13625000	-2.22298200
H	4.19858400	-3.15534600	-1.95280500
H	3.18219800	-2.65893800	-0.55396400
S	-0.13083700	3.16225000	-0.29421500
O	0.94341100	2.52159500	-1.19435500
C	0.39092400	2.85222200	1.41954500
H	-0.31312500	3.33911800	2.10022900
H	0.38561700	1.76961300	1.57045100
H	1.40267300	3.24428500	1.56401000
C	0.21517800	4.94760300	-0.30710500
H	-0.00089200	5.32096200	-1.31160700
H	-0.44109400	5.44013700	0.41700500
H	1.26659900	5.12059600	-0.05801200
S	2.34635700	-0.86538200	1.95592800
O	1.11698300	-0.37014200	1.17422200
C	3.33306000	0.59327500	2.39778500
H	4.16466800	0.27484700	3.03433800
H	3.71164400	0.99846900	1.45776800
H	2.70714500	1.32233800	2.91983200
C	1.72628200	-1.25041900	3.61919900
H	1.04142600	-2.09735800	3.52516300
H	2.56615200	-1.52340900	4.26529900
H	1.19590100	-0.38082900	4.01845200

F

C	4.01907700	-0.56738100	0.01845200
C	2.93250900	-1.42625500	0.22214700
C	1.61909000	-0.96253200	0.22268400
C	1.29944700	0.41914800	0.00995300
C	2.44065800	1.27329500	-0.16571200
C	3.74310300	0.79858800	-0.16465400
H	5.03922900	-0.94068100	0.01203900
H	3.11010200	-2.48684100	0.39555300
H	0.82901900	-1.67032200	0.44130300
H	2.26364700	2.33802900	-0.31227000
H	4.56222800	1.50120400	-0.31112400
C	0.00000000	1.00104900	-0.00001300
H	-0.00000300	2.09253000	-0.00000200
C	-4.01908200	-0.56737600	-0.01843500
C	-3.74309300	0.79859800	0.16467000
C	-2.44065100	1.27329300	0.16571100
C	-1.29943600	0.41913700	-0.00996600
C	-1.61909600	-0.96253500	-0.22269700
C	-2.93252400	-1.42624900	-0.22214200
H	-5.03923900	-0.94066100	-0.01201400
H	-4.56221500	1.50121600	0.31114900
H	-2.26362500	2.33802500	0.31226500
H	-0.82904300	-1.67034200	-0.44133300
H	-3.11011400	-2.48683600	-0.39555100

N_2

N	0.00000000	0.00000000	0.55239400
N	0.00000000	0.00000000	-0.55239400

10-ts

C	3.71008200	-0.96800300	0.65158200
C	3.68348300	-0.33772900	-0.59823500
C	2.49702000	0.19328500	-1.09951600
C	1.28295400	0.12530400	-0.37693900
C	1.33355100	-0.51804600	0.88052400
C	2.52085200	-1.05179800	1.38245900
H	4.63355500	-1.38613500	1.04297600
H	4.59414700	-0.26565200	-1.18945700
H	2.49762900	0.67529500	-2.07576800
H	0.42339300	-0.60203500	1.46844900
H	2.51542500	-1.54190400	2.35405200
C	0.06080800	0.78478600	-0.89650800
H	0.12161300	0.84726000	-1.99225700
C	-3.69039000	-0.97798700	0.37097400
C	-3.05056500	-1.45407700	-0.77758200
C	-1.84043700	-0.89158700	-1.19097700
C	-1.24490800	0.16386000	-0.47858500
C	-1.91246000	0.64453500	0.66323200
C	-3.11538600	0.07566000	1.08791100
H	-4.62914800	-1.41840100	0.69755800
H	-3.49215100	-2.26814000	-1.34802500
H	-1.34411700	-1.27695700	-2.07986900
H	-1.46851900	1.50509100	1.16245500
H	-3.61430600	0.46239700	1.97415600
H	0.04822300	1.97480900	-0.38224800
H	-0.95558300	3.42508400	-0.01438600
O	-0.09222300	3.14251100	0.33404100

3v

C	3.56371500	-0.10755000	-0.93562400
C	2.70725200	-1.21308700	-0.91586100
C	1.56452000	-1.19959600	-0.11657300
C	1.25886500	-0.08602100	0.67941000
C	2.12227100	1.01510700	0.65256300
C	3.26776600	1.00655400	-0.14874500
H	4.45427600	-0.11714900	-1.55808900
H	2.93086700	-2.08585000	-1.52375300
H	0.89779000	-2.05872100	-0.10961700
H	1.89740800	1.88376500	1.26650900
H	3.92767500	1.87009300	-0.15594500
C	-0.00205600	-0.07133000	1.52297800
H	0.04059700	0.77835500	2.21420200
C	-3.56320700	0.19574300	-0.92910400
C	-3.29311500	-0.97091300	-0.21236000
C	-2.14769300	-1.05515100	0.58496500
C	-1.25895400	0.02180100	0.67778800
C	-1.53808200	1.18836500	-0.04911600
C	-2.68047100	1.27736100	-0.84392600
H	-4.45330200	0.26441100	-1.54847900
H	-3.97293300	-1.81687900	-0.27115200
H	-1.94283300	-1.96500200	1.14393400
H	-0.85129700	2.02950400	0.00879800
H	-2.88328300	2.19025500	-1.39783000
H	-0.04984800	-0.98048800	2.13304800

11-ts

C	3.70075000	-0.86595000	0.19015100
C	3.39832900	0.33755300	0.84256700
C	2.23141200	1.02493500	0.53803000
C	1.34093000	0.51762400	-0.43698200
C	1.65665100	-0.69658900	-1.08668600
C	2.82606000	-1.38021400	-0.77359100
H	4.61370200	-1.40095800	0.43529900
H	4.07818500	0.73296400	1.59174800
H	1.98493200	1.95214800	1.04393200
H	0.96814100	-1.09355900	-1.82730400
H	3.05879100	-2.31434300	-1.27635000
C	0.10989300	1.17851000	-0.76535200
N	-0.29163000	2.27836000	-0.12302400
H	-0.53814100	0.73465400	-1.52358600
N	-1.46749000	2.70523700	-0.51826000
H	-1.69259900	3.49723600	0.10161200
C	-1.70364500	-2.12035900	0.18841400
C	-1.35419300	-1.36323400	1.31113100
C	-1.91076200	-0.08816500	1.50369400
C	-2.79956600	0.35145500	0.54631500
C	-3.18877600	-0.35247500	-0.57214500
C	-2.61663600	-1.62463900	-0.74747400
H	-1.25968400	-3.10077200	0.04089200
H	-0.64311500	-1.75383900	2.03475700
H	-1.62760300	0.51922600	2.35895400
H	-3.88769100	0.04923600	-1.30079000
H	-2.88375500	-2.21787900	-1.61862700

I^-

C	-4.83266200	-0.47271300	-0.24421300
C	-3.93645000	-1.53874600	-0.34503800
C	-2.56529200	-1.31465100	-0.21866200
C	-2.06634000	-0.02095100	0.00957600
C	-2.97872500	1.04636500	0.11113700
C	-4.34580500	0.82024400	-0.01580200
H	-5.90096300	-0.64496800	-0.34046600
H	-4.30397900	-2.54609400	-0.52064900
H	-1.86893000	-2.14603000	-0.29672800
H	-2.60213400	2.04827300	0.29123100
H	-5.03847500	1.65373300	0.06512900
C	-0.61758900	0.16422500	0.13466100
N	-0.11934200	1.34047400	0.29870400
H	-0.00921500	-0.73788400	0.06581400
N	1.22459100	1.54485000	0.45023200
H	1.45300700	2.49769700	0.19233700
C	4.40202400	-1.14436800	-0.25218800
C	3.38187300	-1.49043700	0.63776300
C	2.32007000	-0.61804800	0.87649600
C	2.25762800	0.60951100	0.19771100
C	3.28481300	0.95865300	-0.69162800
C	4.35390500	0.08956800	-0.90589900
H	5.22909500	-1.82571100	-0.42867300
H	3.41873400	-2.43912100	1.16590600
H	1.56022700	-0.87529100	1.60623200
H	3.23671400	1.91203900	-1.21146600
H	5.14448200	0.37417800	-1.59469600

12-ts

C	-4.88888700	2.49176000	0.24278900
C	-4.89665300	1.80023800	-0.97455900
C	-3.76476800	1.11674000	-1.40959200
C	-2.58670000	1.10315100	-0.63197100
C	-2.59528700	1.80134300	0.59422300
C	-3.72843100	2.48985600	1.02202100
H	-5.77435200	3.02549000	0.57658600
H	-5.79452000	1.79348100	-1.58760600
H	-3.77755200	0.58183400	-2.35305700
H	-1.69959900	1.79902100	1.20815800
H	-3.70643400	3.02368600	1.96873400
C	-1.41082800	0.34743300	-1.01433900
N	-1.26777300	-0.09780100	-2.27880700
H	-0.53332100	0.43929000	-0.37533100
N	-0.07159300	-0.44929800	-2.63193200
H	-0.15441200	-0.85610000	-3.57046500
C	-4.75422200	-2.07797400	0.84219900
C	-4.36390200	-2.44384900	-0.45140400
C	-3.04525500	-2.26610900	-0.87917200
C	-2.13294700	-1.68427200	-0.00231600
C	-2.48472700	-1.33887000	1.30005300
C	-3.80842600	-1.52744300	1.71084300
H	-5.78032400	-2.22262300	1.16727600
H	-5.08888100	-2.87028900	-1.14113600
H	-2.75299900	-2.53465400	-1.88902000
H	-1.75494300	-0.90805700	1.97714700
H	-4.09232400	-1.23984800	2.72068300
I	0.16372600	-2.67671600	-0.09914800
Na	1.70447100	0.26248500	-1.24925400
S	0.66621900	3.13522300	-0.30495500
O	1.31270500	2.46616700	-1.52826300
C	1.92319500	4.24670100	0.38804100
H	1.47676300	4.85021200	1.18396400
H	2.71531500	3.61649300	0.79912700
H	2.32192100	4.88740000	-0.40426700
C	-0.46016800	4.40133000	-0.95172700
H	-1.25616500	3.88014000	-1.48910100
H	-0.88927900	4.95661000	-0.11214900
H	0.07953700	5.07177400	-1.62697600
S	1.04258700	0.38486100	2.15937600
O	2.00080100	0.57981000	0.96746500
C	1.80238800	-0.87999200	3.21494600
H	1.21860800	-0.96520200	4.13660000
H	1.76555200	-1.82276100	2.66512300
H	2.83609000	-0.59896700	3.43480400
C	1.31977800	1.80519100	3.25402500
H	0.91774200	2.69066400	2.75776100
H	0.78283400	1.63788800	4.19256600
H	2.39201900	1.92271600	3.43545700
S	4.93050500	-0.70876200	-1.00499600
O	3.72551600	-0.42616400	-1.92070600
C	5.14415400	0.75446700	0.05566300
H	5.97471600	0.56786800	0.74396000
H	5.39125400	1.59562000	-0.59795300
H	4.21447900	0.94540500	0.60059700
C	4.34385100	-1.84056600	0.29210800
H	4.04919200	-2.77352900	-0.19524500
H	5.16278000	-2.03201700	0.99259100
H	3.49109200	-1.38768200	0.80320100

13

Na	0.32073800	0.14281800	-0.31719600
S	-2.89803600	0.27291500	-1.24162400
O	-1.55804100	1.03052500	-1.10300200
C	-4.04212800	1.46558000	-1.98890500
H	-3.68713700	1.66801800	-3.00257200
H	-5.04247100	1.02414100	-2.02835900
H	-4.04670500	2.38523300	-1.39670300
C	-3.64123300	0.22403600	0.41438300

H	-4.63130100	-0.23584400	0.33639900
H	-2.99523700	-0.39586800	1.04139100
H	-3.71712400	1.23661300	0.81981700
S	0.57609000	2.87848100	1.60378000
O	0.92141500	1.38068300	1.45486200
C	0.82463900	3.63313400	-0.03182700
H	0.53615000	4.68751000	0.01927700
H	1.88728200	3.55468300	-0.27390700
H	0.21918400	3.10961000	-0.77810200
C	-1.23732400	2.98313200	1.62686900
H	-1.58237600	2.47571100	2.53145400
H	-1.53030300	4.03720300	1.65771000
H	-1.62632600	2.48895000	0.73251100
S	3.52006100	-0.12024800	-1.38451900
O	2.02637200	0.01063100	-1.74083900
C	3.89503600	1.22004700	-0.21574700
H	4.90707700	1.07525900	0.17506400
H	3.84668800	2.16090500	-0.77018800
H	3.15410400	1.21456400	0.58949100
C	3.66031200	-1.50107300	-0.20968600
H	3.33377700	-2.40588100	-0.72827100
H	4.70798500	-1.60375700	0.08922300
H	3.02756500	-1.31861900	0.66399800
I	-0.51570900	-2.69342800	0.63603900

14-ts

C	4.93646300	-0.02157600	-0.36946400
C	4.29337700	1.20505300	-0.17761100
C	2.97206500	1.24299700	0.30028500
C	2.37570800	0.02744400	0.55723700
C	2.95704900	-1.21078700	0.38842500
C	4.27866500	-1.22343100	-0.09055400
H	5.95795100	-0.04089800	-0.73858500
H	4.81208500	2.13514800	-0.39655100
H	2.46128400	2.18993000	0.45342200
H	2.43500000	-2.13809500	0.60887000
H	4.78563100	-2.17321000	-0.24210200
C	-4.98196100	-0.09510500	0.20151200
C	-4.23193600	-1.25805300	0.00138300
C	-2.86154800	-1.17222000	-0.29940700
C	-2.32771000	0.09579100	-0.37991900
C	-3.01454600	1.27606200	-0.19327200
C	-4.38353400	1.16497200	0.10614500
H	-6.04049500	-0.17108200	0.43358100
H	-4.70476600	-2.23409000	0.07741700
H	-2.26851300	-2.06888700	-0.45807400
H	-2.53764100	2.24956100	-0.27016000
H	-4.97309100	2.06475000	0.26362600

15

C	0.00000000	3.56208600	0.00000000
C	-0.36292100	2.85797400	1.15067700
C	-0.36328000	1.46332600	1.15058900
C	0.00000000	0.74192300	-0.00000100
C	0.36328000	1.46332700	-1.15058900
C	0.36292100	2.85797500	-1.15067600
H	0.00000000	4.64852300	0.00000100
H	-0.65419500	3.39513500	2.04925700
H	-0.66959300	0.92780400	2.04430200
H	0.66959400	0.92780500	-2.04430200
H	0.65419500	3.39513700	-2.04925600
C	0.00000000	-3.56208600	0.00000000
C	-0.36292100	-2.85797500	-1.15067600
C	-0.36328000	-1.46332700	-1.15058900
C	0.00000000	-0.74192300	-0.00000100
C	0.36328000	-1.46332600	1.15058900
C	0.36292100	-2.85797400	1.15067700
H	0.00000000	-4.64852300	0.00000100
H	-0.65419500	-3.39513700	-2.04925600
H	-0.66959400	-0.92780500	-2.04430200
H	0.66959300	-0.92780400	2.04430200
H	0.65419500	-3.39513500	2.04925700

16-ts

C	-4.53734700	-0.27083500	-0.32841000
C	-3.92101500	-0.54675500	0.89548700
C	-2.56917400	-0.88231900	0.94299400
C	-1.81239100	-0.94917900	-0.24056400
C	-2.44250000	-0.67358600	-1.46659100
C	-3.79383600	-0.33890300	-1.51044800
H	-5.59092900	-0.00793200	-0.36138500
H	-4.49492500	-0.49778300	1.81672000
H	-2.09161200	-1.09630400	1.89213800
H	-1.86347600	-0.72407900	-2.38451300
H	-4.26766100	-0.13104400	-2.46574800
C	-0.37646900	-1.25920400	-0.22819900
N	0.07468500	-2.01806100	0.86290300
H	0.07715800	-1.48976500	-1.19390300
N	1.02540800	-2.80066500	0.57745000
H	1.29443800	-3.26293000	1.46152900
C	-1.30771300	2.62049500	0.65852800
C	-0.71361200	1.89232300	1.70706100
C	0.22175700	0.91039800	1.44418800
C	0.52411000	0.55844800	0.08736200
C	0.00017700	1.38249300	-0.96248200
C	-0.93084500	2.36200700	-0.67061600
H	-2.03794700	3.39280200	0.87772700
H	-0.98218800	2.11036500	2.73690800
H	0.67772800	0.34900300	2.25157700
H	0.29053300	1.18356200	-1.98812400
H	-1.36450100	2.94251500	-1.47994100
I	2.69067700	0.04019600	-0.24934600

17-ts

C	-4.94803600	2.33440200	-0.02505100
C	-4.95344400	1.46978400	-1.12330500
C	-3.83080300	0.69808800	-1.41793000
C	-2.68250100	0.78428800	-0.61229700
C	-2.68869300	1.65029300	0.49491600
C	-3.81067600	2.42406600	0.78268700
H	-5.82466700	2.93541000	0.19930900
H	-5.83580400	1.39551700	-1.75259500
H	-3.83525600	0.03005900	-2.27140200
H	-1.80815500	1.71254400	1.12657500
H	-3.79863000	3.09440800	1.63735500
C	-1.48490700	-0.02479200	-0.87445500
N	-1.29887700	-0.43481400	-2.19941400
H	-0.59070200	0.27392000	-0.32857100
N	-0.08511200	-0.51087100	-2.54821300
H	-0.06778100	-0.87607700	-3.51096700
C	-4.42356800	-1.98848700	1.11561900
C	-4.11521200	-2.51016600	-0.15588300
C	-2.82580300	-2.45871500	-0.64762200
C	-1.80454600	-1.77831200	0.09811000
C	-2.10600200	-1.36452600	1.43927300
C	-3.40405300	-1.43223500	1.90747400
H	-5.44049600	-2.03681800	1.49113500

H	-4.89596000	-2.96943000	-0.75538600
H	-2.58980300	-2.85541000	-1.62838600
H	-1.32104100	-0.92997500	2.04731400
H	-3.63132300	-1.06015800	2.90230800
I	0.22842800	-2.71874300	-0.05291400
Na	1.67604300	0.49727600	-1.30789900
S	0.38265500	3.26429100	-0.29374800
O	0.70680800	2.48185400	-1.57911800
C	1.85969900	4.24234100	0.09813100
H	1.62941800	4.93459600	0.91354500
H	2.63113700	3.53718100	0.41617800
H	2.18292300	4.78829300	-0.79341500
C	-0.70561500	4.62349400	-0.79537100
H	-1.64528100	4.17761100	-1.13090600
H	-0.89259900	5.26591200	0.07028000
H	-0.24178000	5.19036500	-1.60779500
S	1.11226900	0.69522300	2.15569400
O	1.96601300	0.80254700	0.87498600
C	1.84991100	-0.63395200	3.14410300
H	1.33142600	-0.68011700	4.10661200
H	1.69796300	-1.56792000	2.59889400
H	2.91622800	-0.43788400	3.28667700
C	1.61182800	2.08728900	3.20461400
H	1.23888600	3.00509900	2.74560900
H	1.15967700	1.96249600	4.19304400
H	2.70295900	2.11072700	3.27503800
S	4.82083800	-0.69953300	-1.10237600
O	3.68764900	-0.14895000	-1.99053400
C	5.18045400	0.58593500	0.13099400
H	5.91986700	0.20001200	0.83982700
H	5.59711100	1.44333000	-0.40458900
H	4.25209600	0.86184300	0.64045000
C	4.05470700	-1.89443500	0.03382400
H	3.67421400	-2.72349900	-0.56800000
H	4.81655200	-2.26199600	0.72829700
H	3.24118400	-1.40638000	0.57554700

8. Characterization data

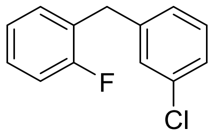

1-(3-chlorobenzyl)-2-fluorobenzene (3a)

Colorless liquid, 32.6 mg, 74% yield. **^{1}H NMR** (600 MHz, $CDCl_3$) δ 7.23 – 7.16 (m, 4H), 7.13 (t, J = 7.4 Hz, 1H), 7.11 – 7.01 (m, 3H), 3.96 (s, 2H). **^{13}C NMR** (151 MHz, $CDCl_3$) δ 161.0 (d, J_{C-F} = 245.1 Hz), 141.9, 134.3, 131.0 (d, J_{C-F} = 4.3 Hz), 129.7 128.9, 128.3 (d, J_{C-F} = 8.1 Hz), 127.2 (d, J_{C-F} = 15.9 Hz), 126.9, 126.5, 124.2 (d, J_{C-F} = 3.3 Hz), 115.5 (d, J_{C-F} = 21.9 Hz), 34.5 (d, J_{C-F} = 2.8 Hz). **^{19}F NMR** (376 MHz, $CDCl_3$) δ -117.70 (m, 1F). **IR** (KBr): 3436, 1635, 1242, 678 cm^{-1}. **MS** (EI, m/z%): 220.00 ($M^{•+}$, 45.35), 185.05 (100.00), 165.07 (46.55), 183.04 (36.65), 91.12 (17.27), 184.07 (15.88) 222.00 (13.96), 186.07 (13.91), 109.05 (13.83), 83.01 (11.19), 91.79 (9.57), 81.08 (8.97). **HRMS** (EI) calcd. for $C_{13}H_{10}Cl^{•+}$ $[M]^{•+}$: 220.0450; Found: 220.0448.

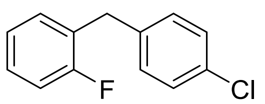

1-(4-chlorobenzyl)-2-fluorobenzene (3b)

Colorless liquid, 29.5 mg, 67% yield. **^{1}H NMR** (400 MHz, $CDCl_3$) δ 7.32 – 7.26 (m, 2H), 7.25 – .21 (m, 1H), 7.18 – 7.14 (m, 3H), 7.13 – 7.03 (m, 2H), 4.00 (s, 2H). **^{13}C NMR** (101 MHz, $CDCl_3$) δ 160.9 (d, J_{C-F} = 245.5 Hz), 138.3, 132.0, 130.9 (d, J_{C-F} = 4.5 Hz), 130.1, 128.6, 128.2 (d, J_{C-F} = 8.1 Hz), 127.5 (d, J_{C-F} = 15.8 Hz), 124.2 (d, J_{C-F} = 3.6 Hz), 115.4 (d, J_{C-F} = 21.9 Hz), 34.2 (d, J_{C-F} = 3.1 Hz). **^{19}F NMR** (282 MHz, $CDCl_3$) δ -117.74 – -117.82 (m, 1F). **IR** (KBr): 3441, 2078, 1635, 1247, 682 cm^{-1}. **MS** (EI, m/z%): 220.03 ($M^{•+}$, 51.35), 185.05 (100.00), 165.06 (45.86), 183.04 (38.84), 91.93 (17.44), 82.03 (16.25), 222.01 (15.99), 184.07 (15.70), 91.11 (15.01), 186.07 (14.19), 89.06 (11.68), 83.00 (9.85), 109.06 (9.36), 81.09 (9.34), 63.05 (7.66). **HRMS** (EI) calcd. for $C_{13}H_{16}ClF^{•+}$ $[M]^{•+}$: 220.0450; Found: 220.0447.

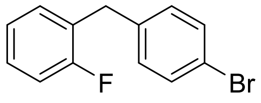

1-(4-bromobenzyl)-2-fluorobenzene (3c)

Colorless liquid, 21.2 mg, 44% yield. **^{1}H NMR** (600 MHz, $CDCl_3$) δ 7.39 (d, J = 8.0 Hz, 2H), 7.20 (q, J = 7.3 Hz, 2H), 7.12 (t, J = 7.5 Hz, 1H), 7.10 – 7.01 (m, 4H), 3.94 (s, 2H). **^{13}C NMR** (151 MHz, $CDCl_3$) δ 160.9 (d, J_{C-F} = 245.3 Hz), 138.9, 131.6, 130.9 (d, J_{C-F} = 4.2 Hz), 130.5, 128.2 (d, J_{C-F} = 8.1 Hz), 127.4 (d, J_{C-F} = 15.9 Hz), 124.2, 120.1, 115.4 (d, J_{C-F} = 22.1 Hz), 34.3. **^{19}F NMR** (282 MHz, $CDCl_3$) δ -118.12 – -118.20 (m, 1F). **IR** (KBr) :2923, 1487, 1231, 754 cm^{-1}. **MS** (EI, m/z %): 264.14 ($M^{•+}$, 20.74), 185.22 (100.00), 165.20 (98.69), 183.18 (55.18), 91.30 (35.15), 92.12 (32.09), 82.16 (28.75), 109.13 (21.37), 184.24 (20.17).

^{1}H and ^{13}C NMR data agreed with the literature[11]

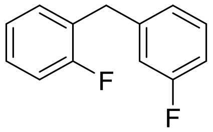

1-fluoro-2-(3-fluorobenzyl)benzene (3d)

Colorless liquid, 29.0 mg, 71% yield. **^{1}H NMR** (600 MHz, $CDCl_3$) δ 7.24 – 7.16 (m, 2H), 7.13 (td, J = 7.5, 1.7 Hz, 1H), 7.07 – 7.01 (m, 2H), 6.98 (d, J = 7.6 Hz, 1H), 6.91 – 6.85 (m, 2H), 3.97 (s, 2H). **^{13}C NMR** (151 MHz, $CDCl_3$) δ 163.0(d, J_{C-F} = 245.7 Hz), 160.9 (d, J_{C-F} = 245.6 Hz), 142.4 (d, J_{C-F} = 7.1 Hz), 131.0 (d, J_{C-F} = 4.3 Hz), 129.9 (d, J_{C-F} = 8.2 Hz), 128.3 (d, J_{C-F} = 8.0 Hz), 127.3 (d, J_{C-F} = 15.8 Hz), 124.4 (d, J_{C-F} = 1.7 Hz), 124.2 (d, J_{C-F} = 3.3 Hz), 115.6 (d, J_{C-F} = 21.3 Hz), 115.4 (d, J_{C-F} = 22.0 Hz), 113.1 (d, J_{C-F} = 21.0 Hz), 34.6. **^{19}F NMR** (376 MHz, $CDCl_3$) δ -113.41 – -113.51 (m, 1F), -117.76 – -117.80 (m, 1F). **IR** (KBr): 3446, 2077, 1635, 750 cm^{-1}. **MS** (EI, m/z%): 204.06 ($M^{•+}$, 100.00), 203.06 (81.80), 183.05 (60.94), 109.05 (43.86), 83.02 (21.25), 201.04 (21.22), 184.06 (20.95), 107.03 (14.43), 205.05 (13.51), 91.10 (10.82), 57.03 (9.89), 91.96 (9.35). **HRMS** (EI) calcd. for $C_{13}H_{10}F_2^{•+}$ $[M]^{•+}$: 204.0745; Found: 204.0745.

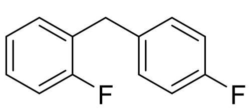

1-fluoro-2-(4-fluorobenzyl)benzene (3e)

Colorless liquid, 26.0 mg, 64% yield. **^{1}H NMR** (600 MHz, $CDCl_3$) δ 7.20 – 7.13 (m, 3H), 7.11 (td, J = 7.6, 1.7 Hz, 1H), 7.06 – 7.00 (m, 2H), 6.97 – 6.93 (m, 2H), 3.95 (s,

2H). **^{13}C NMR** (151 MHz, $CDCl_3$) δ 162.0 (d, J_{C-F} = 85.5 Hz), 160.4 (d, J_{C-F} = 86.7 Hz), 135.5 (d, J_{C-F} = 2.9 Hz), 130.9 (d, J_{C-F} = 4.4 Hz), 130.1 (d, J_{C-F} = 7.8 Hz), 128.1 (d, J_{C-F} = 8.0 Hz), 127.9 (d, J_{C-F} = 15.8 Hz), 124.1 (d, J_{C-F} = 3.3 Hz), 115.4 (d, J_{C-F} = 20.2 Hz), 115.2 (d, J_{C-F} = 21.14 Hz), 34.1 (d, J_{C-F} = 2.6 Hz). **^{19}F NMR** (376 MHz, $CDCl_3$) δ -116.86 – -117.27 (m, 1F), -117.59 – -118.06 (m, 1F). **IR** (KBr): 3044, 1509, 1229, 755 cm^{-1}. **MS** (EI, m/z%): 204.06 ($M^{\bullet+}$, 100.00), 203.05 (79.10), 183.06 (60.01), 109.06 (50.62), 83.08 (24.48), 184.08 (22.45), 201.04 (22.16), 107.08 (16.41), 205.06 (14.13), 108.07 (13.48), 57.04 (12.19), 91.12 (11.93), 92.00 (9.71), 81.09 (9.37). **HRMS** (EI) calcd. for $C_{13}H_{10}F_2^{\bullet+}$ $[M]^{\bullet+}$: 204.0745; Found: 204.0744.

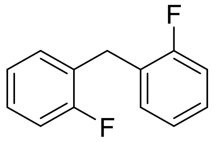

Bis(2-fluorophenyl)methane (3f)

Colorless liquid, 22.8 mg, 56% yield. **^{1}H NMR** (400 MHz, $CDCl_3$) δ 7.22 – 7.13 (m, 4H), 7.07 – 7.01 (m, 4H), 4.02 (s, 2H). **^{13}C NMR** (151 MHz, $CDCl_3$) δ 161.1 (d, J_{C-F} = 245.2 Hz), 132.0 (d, J_{C-F} = 3.02 Hz), 128.1 (d, J_{C-F} = 8.1 Hz), 126.7 (d, J_{C-F} = 15.7 Hz), 124.1, 115.3 (d, J_{C-F} = 21.8 Hz), 27.8. **^{19}F NMR** (376 MHz, $CDCl_3$) δ -117.76 – -117.82 (m, 2F). **IR** (KBr): 3438, 2078, 1635, 1234, 752 cm^{-1}. **MS** (EI, m/z%): 204.07 ($M^{\bullet+}$, 100.00), 203.05 (78.13), 183.05 (59.29), 109.06 (40.21), 184.05 (20.96), 83.04 (19.29), 201.04 (17.50), 205.06 (14.26), 107.04 (12.85), 108.07 (10.98), 91.16 (10.01), 91.98 (8.96), 57.04 (8.34), 81.07 (8.26). **HRMS** (EI) calcd. for $C_{13}H_{10}F_2^{\bullet+}$ $[M]^{\bullet+}$: 204.0745; Found: 204.0744.

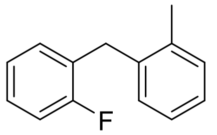

1-fluoro-2-(2-methylbenzyl)benzene (3g)

Colorless liquid, 6.0 mg, 15% yield. **^{1}H NMR** (600 MHz, $CDCl_3$) δ 7.20 – 7.13 (m, 4H), 7.08 – 7.00 (m, 3H), 6.98 – 6.92 (m, 1H), 3.99 (s, 2H), 2.26 (s, 3H). **^{13}C NMR** (151 MHz, $CDCl_3$) δ 161.0 (d, J_{C-F} = 245.1 Hz), 137.5, 136.6, 130.6 (d, J_{C-F} = 4.5 Hz), 130.3, 129.7, 127.7 (d, J_{C-F} = 8.0 Hz), 127.3 (d, J_{C-F} = 15.8 Hz), 126.6, 126.0, 124.0 (d, J_{C-F} = 3.7 Hz), 115.1 (d, J_{C-F} = 21.9 Hz), 32.0 (d, J_{C-F} = 3.3 Hz), 19.5. **^{19}F NMR** (565

MHz, $CDCl_3$) δ -117.60 – -117.64 (m, 1F). **HRMS** (EI) calcd. for $C_{14}H_{13}F^{•+}$ $[M]^{•+}$: 200.0996; Found: 200.0997.

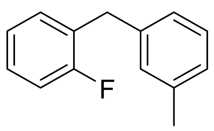

1-fluoro-2-(3-methylbenzyl)benzene (3h)

Colorless liquid, 18.0 mg, 45% yield. **^{1}H NMR** (400 MHz, $CDCl_3$) δ 7.21 – 7.10 (m, 3H), 7.08 – 6.95 (m, 5H), 3.96 (s, 2H), 2.31 (s, 3H). **^{13}C NMR** (101 MHz, $CDCl_3$) δ 160.9 (d, $J_{C\text{-}F}$ = 245.2 Hz), 139.7, 138.1, 131.0 (d, $J_{C\text{-}F}$ = 4.7 Hz), 129.6, 128.4, 128.2 (d, $J_{C\text{-}F}$ = 15.9 Hz), 127.8 (d, $J_{C\text{-}F}$ = 8.0 Hz), 127.0, 125.8, 124.0 (d, $J_{C\text{-}F}$ = 3.6 Hz), 115.3 (d, $J_{C\text{-}F}$ = 22.1 Hz), 34.7 (d, $J_{C\text{-}F}$ = 3.0 Hz), 21.4. **^{19}F NMR** (376 MHz, $CDCl_3$) δ -117.89 – -117.95 (m, 1F). **IR** (KBr): 3400, 2995, 1770, 1245, 672 cm^{-1}. **MS** (EI, m/z%): 200.08 ($M^{•+}$, 77.19), 185.04 (100.00), 165.06 (35.04), 183.04 (28.63), 184.05 (15.77), 91.09 (14.25), 199.09 (11.94), 98.10 (10.95), 201.10 (10.05), 77.06 (9.75), 83.01(9.63). **HRMS** (EI) calcd. for $C_{14}H_{13}F^{•+}$ $[M]^{•+}$: 200.0996; Found: 200.0994.

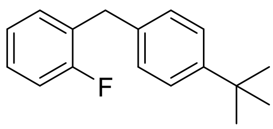

1-(4-(tert-butyl)benzyl)-2-fluorobenzene (3i)

Colorless liquid, 27.6 mg, 57% yield. **^{1}H NMR** (600 MHz, $CDCl_3$) δ 7.32 (d, J = 8.3 Hz, 2H), 7.21 – 7.15 (m, 4H), 7.08 – 7.02 (m, 2H), 3.98 (s, 2H), 1.31 (s, 9H). **^{13}C NMR** (151 MHz, $CDCl_3$) δ 161.0 (d, $J_{C\text{-}F}$ = 245.1 Hz), 149.0, 136.8, 131.1 (d, $J_{C\text{-}F}$ = 4.5 Hz), 128.4, 128.2 (d, $J_{C\text{-}F}$ = 15.8 Hz), 127.8 (d, $J_{C\text{-}F}$ = 7.9 Hz), 125.4, 124.0 (d, $J_{C\text{-}F}$ = 3.3 Hz), 115.3 (d, $J_{C\text{-}F}$ = 22.1 Hz), 34.4, 34.3 (d, $J_{C\text{-}F}$ = 2.9 Hz), 31.4. **^{19}F NMR** (376 MHz, $CDCl_3$) δ -117.92 (m, -117.89- -117.95, 1F). **IR** (KBr): 2963, 1514, 1268, 754 cm^{-1}. **MS** (EI, m/z %): 242.15 ($M^{•+}$, 22.31), 109.07 (100.00), 227.11 (89.92), 228.13 (13.83), 99.54 (10.94), 91.13 (9.89), 83.05 (8.87), 110.09 (7.93), 98.16 (7.87), 89.05 (6.25), 183.07 (6.17), 113.65 (6.10), 92.07 (5.95), 98.76 (5.77), 105.10 (5.73). **HRMS** (EI) calcd. for $C_{17}H_{19}F^{•+}$ $[M]^{•+}$: 242.1465; Found: 242.1463.

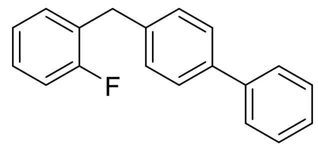

4-(2-fluorobenzyl)-1,1'-biphenyl (**3j**)

Colorless liquid, 31.4 mg, 60% yield. **^{1}H NMR** (400 MHz, $CDCl_3$) δ 7.56 – 7.54 (m, 2H), 7.51 – 7.49 (m, 2H), 7.42 – 7.38 (m, 2H), 7.33 – 7.26 (m, 3H), 7.22 – 7.16 (m, 2H), 7.08 – 7.02 (m, 2H), 4.03 (s, 2H). **^{13}C NMR** (101 MHz, $CDCl_3$) δ 161.0 (d, $J_{C\text{-}F}$ = 245.3 Hz), 140.9, 139.2, 138.9, 131.0 (d, $J_{C\text{-}F}$ = 4.5 Hz), 129.2, 128.7, 128.0 (d, $J_{C\text{-}F}$ = 8.8 Hz), 127.9 (d, $J_{C\text{-}F}$ = 16.2 Hz) 127.2, 127.1, 127.0, 124.1 (d, $J_{C\text{-}F}$ = 3.6 Hz), 115.4 (d, $J_{C\text{-}F}$ = 22.0 Hz), 34.5 (d, $J_{C\text{-}F}$ = 3.0 Hz). **^{19}F NMR** (376 MHz, $CDCl_3$) δ -117.82 (m, 1F). **IR** (KBr): 3454, 2078, 1636, 753 cm^{-1}. **MS** (EI, m/z%): 262.09 ($M^{\bullet+}$, 100), 165.05 (49.82), 261.11 (20.29), 109.08 (19.62), 183.04 (19.51), 167.10 (18.98), 263.14 (18.68), 152.06 (17.36), 207.02 (16.74), 115.05 (11.96), 91.16 (8.60). **HRMS** (EI) calcd. for $C_{19}H_{15}F^{\bullet+}$ $[M]^{\bullet+}$: 262.1152; Found: 262.1152.

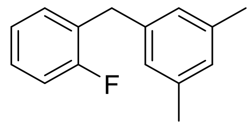

1-(2-fluorobenzyl)-3,5-dimethylbenzene (**3k**)

Colorless liquid, 24.4 mg, 57% yield. **^{1}H NMR** (600 MHz, $CDCl_3$) δ 7.16 (m, 2H), 7.03 (m, 2H), 6.84 – 6.83 (m, 3H), 3.92 (s, 2H), 2.27 (s, 6H). **^{13}C NMR** (151 MHz, $CDCl_3$) δ 161.0 (d, $J_{C\text{-}F}$ = 245.7 Hz), 139.7, 138.0, 131.1 (d, $J_{C\text{-}F}$ = 4.6 Hz), 128.3 (d, $J_{C\text{-}F}$ = 15.9 Hz), 127.9, 127.8 (d, $J_{C\text{-}F}$ = 8.0 Hz), 126.6, 124.0 (d, $J_{C\text{-}F}$ = 3.4 Hz), 115.3 (d, $J_{C\text{-}F}$ = 22.1 Hz), 34.6 (d, $J_{C\text{-}F}$ = 2.4 Hz), 21.3 **^{19}F NMR** (376 MHz, $CDCl_3$) δ -117.77 – -118.04 (m, 1F). **IR** (KBr): 3449, 1640, 754 cm^{-1}. **MS** (EI, m/z%): 214.12 ($M^{\bullet+}$, 68.21), 199.09 (100.00), 183.09 (25.71), 184.08 (25.19), 105.10 (23.18), 179.11 (18.09), 200.10 (17.24), 77.08 (13.41), 106.10 (12.36), 215.13 (11.42), 109.06 (10.77), 91.13 (10.57), 118.12 (9.86), 83.04 (8.85), 98.10 (8.66). **HRMS** (EI) calcd. for $C_{15}H_{15}F^{\bullet+}$ $[M]^{\bullet+}$: 214.1152; Found: 214.1150.

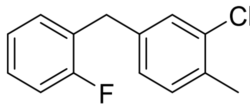

2-chloro-4-(2-fluorobenzyl)-1-methylbenzene (**3l**)

Colorless liquid, 34.2 mg, 73% yield. **^{1}H NMR** (400 MHz, $CDCl_3$) δ 7.24 – 7.15 (m, 2H), 7.14 – 7.11 (m, 2H), 7.06 – 6.98 (m, 3H), 3.92 (s, 2H), 2.31 (s, 3H). **^{13}C NMR** (101 MHz, $CDCl_3$) δ 160.9 (d, J_{C-F} = 245.6 Hz), 139.1, 134.3, 133.8, 130.9 (t, J_{C-F} = 2.2 Hz, 2C), 129.2, 128.1 (d, J_{C-F} = 8.1 Hz), 127.5 (d, J_{C-F} = 15.8 Hz), 127.0, 124.1 (d, J_{C-F} = 3.6 Hz), 115.4 (d, J_{C-F} = 22.0 Hz), 34.1 (d, J_{C-F} = 3.1 Hz), 19.6. **^{19}F NMR** (376 MHz, $CDCl_3$) δ -117.74 – -117.80 (m, 1F). **IR** (KBr): 3452, 2077, 1638, 752 cm^{-1}. **MS** (EI, m/z%): 234.02 ($M^{•+}$, 43.63), 199.07 (100), 183.07 (36.19), 98.10 (15.70), 179.09 (15.33), 236.02 (14.72), 200.10 (14.70) 109.07 (9.66), 77.04 (9.64), 85.07 (7.87). **HRMS** (EI) calcd. for $C_{14}H_{12}ClF^{•+}$ $[M]^{•+}$: 234.0606; Found: 234.0606.

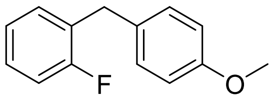

1-fluoro-2-(4-methoxybenzyl)benzene (**3m**)

Colorless liquid, 23.8 mg, 55% yield. **^{1}H NMR** (600 MHz, $CDCl_3$) δ 7.19 – 7.16 (m, 1H), 7.13 (m, 3H), 7.07 – 6.99 (m, 2H), 6.83 (d, J = 8.5 Hz, 2H), 3.94 (s, 2H), 3.78 (s, 3H). **^{13}C NMR** (151 MHz, $CDCl_3$) δ 160.9 (d, J_{C-F} = 245.6 Hz), 158.1, 132.0, 130.9 (d, J_{C-F} = 4.5 Hz), 129.7, 128.5 (d, J_{C-F} = 15.8 Hz), 127.8 (d, J_{C-F} = 8.0 Hz), 124.0 (d, J_{C-F} = 3.5 Hz), 115.3 (d, J_{C-F} = 22.1 Hz), 114.0, 55.3, 33.9 (d, J_{C-F} = 3.0 Hz). **^{19}F NMR** (376 MHz, $CDCl_3$) δ -112.94 – -121.65 (m, 1F). **IR** (KBr): 3444, 2079, 1639, 755 cm^{-1}. **MS** (EI, m/z%): 216.10 ($M^{•+}$, 100.00), 121.08 (43.47), 215.10 (36.65), 185.07 (29.67), 201.08 (20.12), 183.06 (19.94), 152.09 (16.38), 77.08 (15.35), 165.08 (15.11), 109.07 (15.05), 217.09 (14.43), 171.07 (13.90), 91.12 (12.84), 153.09 (12.44), 170.06 (11.22). **HRMS** (EI) calcd. for $C_{14}H_{13}FO^{•+}$ $[M]^{•+}$: 216.0945; Found: 216.0942.

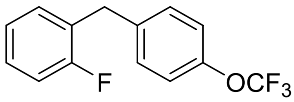

1-fluoro-2-(4-(trifluoromethoxy)benzyl)benzene (**3n**)

Colorless liquid, 35.1 mg, 65% yield. **^{1}H NMR** (600 MHz, $CDCl_3$) δ 7.24 – 7.18 (m, 3H), 7.17 – 7.10 (m, 3H), 7.09 – 7.01 (m, 2H), 3.99 (s, 2H). **^{13}C NMR** (151 MHz,

$CDCl_3$) δ 161.0 (d, $J_{C\text{-}F}$ = 245.6 Hz), 147.7, 138.6, 130.9 (d, $J_{C\text{-}F}$ = 4.4 Hz), 130.0, 128.3 (d, $J_{C\text{-}F}$ = 8.0 Hz), 127.4 (d, $J_{C\text{-}F}$ = 15.8 Hz), 124.2 (d, $J_{C\text{-}F}$ = 3.5 Hz), 121.0, 120.5 (q, $J_{C\text{-}F}$ = 256.7 Hz) 115.5 (d, $J_{C\text{-}F}$ = 22.0 Hz), 34.2 (d, $J_{C\text{-}F}$ = 2.7 Hz). **^{19}F NMR** (376 MHz, $CDCl_3$) δ -57.91 (s, 3F), -117.75 – -117.81 (m, 1F). **IR** (KBr): 3439, 2077, 1636, 754 cm^{-1}. **MS** (EI, m/z%): 270.02 ($M^{\bullet+}$, 55.65), 185.05 (100.00), 183.07 (42.79), 165.06 (31.78), 109.06 (22.37), 186.06 (13.80), 69.01 (13.23), 83.03 (12.04), 269.01 (11.27), 170.05 (11.02), 77.07 (6.10). **HRMS** (EI) calcd. for $C_{14}H_{10}F_4O^{\bullet+}$ $[M]^{\bullet+}$: 270.0662; Found: 270.0663.

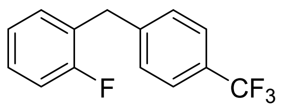

1-fluoro-2-(4-(trifluoromethyl)benzyl)benzene (3o)

Colorless liquid, 32.0 mg, 63% yield. **^{1}H NMR** (400 MHz, $CDCl_3$) δ 7.52 (d, J = 7.6 Hz, 2H), 7.31 (d, J = 7.7 Hz, 2H), 7.23 – 7.18 (m, 1H), 7.16 – 7.12 (m, 1H), 7.08 – 7.02 (m, 2H), 4.03 (s, 2H). **^{13}C NMR** (151 MHz, $CDCl_3$) δ 161.0 (d, $J_{C\text{-}F}$ = 245.7 Hz), 144.1, 131.0 (d, $J_{C\text{-}F}$ = 4.4 Hz), 129.0, 128.5 (d, $J_{C\text{-}F}$ = 8.1 Hz), 127.0 (d, $J_{C\text{-}F}$ = 15.4 Hz), 125.4 (q, $J_{C\text{-}F}$ = 3.4 Hz), 124.4 (q, 271.8 Hz), 124.3 (d, $J_{C\text{-}F}$ = 3.7 Hz), 115.5 (d, $J_{C\text{-}F}$ = 21.9 Hz), 34.8 (d, $J_{C\text{-}F}$ = 3.1 Hz). **^{19}F NMR** (376 MHz, $CDCl_3$) δ -62.34 – -62.35 (m, 3F), -117.59 – -117.61 (m, 1F). **IR** (KBr): 3049, 2935, 1328, 1067,754 cm^{-1}. **MS** (EI, m/z%): 254.06 ($M^{\bullet+}$, 57.13), 185.09 (100.00), 165.09 (39.22), 183.06 (35.95), 109.06 (31.75), 186.09 (14.77), 83.03 (13.50), 184.09 (13.44), 91.56 (10.12), 107.06 (9.21), 255.07 (8.32), 233.05 (8.32), 253.07 (7.75), 235.06 (7.56), 63.07 (6.03). **HRMS** (EI) calcd. for $C_{14}H_{10}F_4^{\bullet+}$ $[M]^{\bullet+}$: 254.0713; Found: 254.0713.

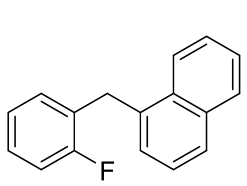

1-(2-fluorobenzyl)naphthalene (3p)

Colorless liquid, 23.6 mg, 50% yield. **^{1}H NMR** (600 MHz, $CDCl_3$) δ 7.98 (d, J = 6.5 Hz, 1H), 7.87 – 7.86 (m, 1H), 7.77 (d, J = 8.2 Hz, 1H), 7.64 – 7.46 (m, 2H), 7.41 (t, J = 7.6 Hz, 1H), 7.27 (d, J = 6.9 Hz, 1H), 7.17 (m, 1H), 7.08 (t, J = 9.1 Hz, 1H), 7.00 – 6.91 (m, 2H), 4.45 (s, 2H). **^{13}C NMR** (151 MHz, $CDCl_3$) δ 160.8 (d, $J_{C\text{-}F}$ = 244.9 Hz),

135.3, 133.9, 132.1, 130.8 (d, J_{C-F} = 4.2 Hz), 128.7, 127.8 (d, J_{C-F} = 8.0 Hz), 127.4 (d, J_{C-F} = 16.2 Hz), 127.3, 127.1, 126.1, 125.6, 125.5, 124.0 (d, J_{C-F} = 3.4 Hz), 123.9, 115.1 (d, J_{C-F} = 22.0 Hz), 31.4 (d, J_{C-F} = 3.1 Hz). **^{19}F NMR** (376 MHz, $CDCl_3$) δ -117.59 – -117.64 (m, 1F). **IR** (KBr): 3447, 2086, 1639, 754 cm^{-1}. **MS** (EI, m/z%): 236.10 ($M^{\bullet+}$, 100.00), 235.10 (48.70), 106.99 (28.00), 215.10 (26.49), 115.10 (25.57), 141.11 (21.52), 220.08 (20.83), 221.09 (20.79), 233.08 (19.69), 237.12 (17.31), 107.99 (12.84), 139.07 (10.54), 216.10 (9.04), 103.50 (8.70), 94.55 (8.69). **HRMS** (EI) calcd. for $C_{17}H_{13}F^{\bullet+}$ $[M]^{\bullet+}$: 236.0996; Found: 236.0994.

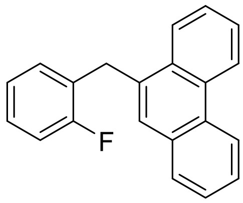

9-(2-fluorobenzyl)phenanthrene (3q)

White solid, m.p.128-129 °C, 25.8 mg, 45% yield. **^{1}H NMR** (600 MHz, $CDCl_3$) δ 8.72 (d, J = 8.3 Hz, 1H), 8.65 (d, J = 8.3 Hz, 1H), 8.01 (d, J = 8.2 Hz, 1H), 7.78 (d, J = 7.9 Hz, 1H), 7.64 – 7.59 (m, 2H), 7.57 – 7.54 (m, 2H), 7.51 (s, 1H), 7.20 – 7.17 (m, 1H), 7.12 – 7.09 (m, 1H), 7.01 – 6.99 (m, 1H), 6.96 – 6.93 (m, 1H), 4.48 (s, 2H). **^{13}C NMR** (151 MHz, $CDCl_3$) δ 160.9 (d, J_{C-F} = 245.7 Hz), 133.5, 131.8, 131.2, 130.8 (d, J_{C-F} = 4.7 Hz, 2C), 130.0, 128.3, 128.0 (d, J_{C-F} = 8.0 Hz), 127.8, 127.1 (d, J_{C-F} = 15.5 Hz), 126.7, 126.7, 126.3 (d, J_{C-F} = 2.8 Hz, 2C), 124.7, 124.1 (d, J_{C-F} = 3.5 Hz), 123.2, 122.5, 115.2 (d, J_{C-F} = 22.1 Hz), 31.9 (d, J_{C-F} = 3.5 Hz). **IR** (KBr): 2372, 1489, 1228, 747 cm^{-1}. **^{19}F NMR** (376 MHz, $CDCl_3$) δ -117.57 – 117.63 (m, 1F). **MS** (EI, m/z %): 286.09 ($M^{\bullet+}$, 100.00), 285.09 (27.26), 287.07 (20.81), 191.09 (18.00), 189.07 (15.11), 283.08 (14.88), 165.06 (14.12), 270.05 (13.96), 265.08 (13.46), 131.57 (13.40), 135.24 (12.46), 133.01 (11.10), 143.15 (10.48), 271.08 (10.35). **HRMS** (EI) calcd. for $C_{21}H_{15}F^{\bullet+}$ $[M]^{\bullet+}$: 286.1152; Found: 286.1151.

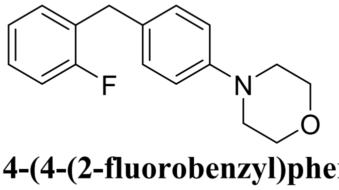

4-(4-(2-fluorobenzyl)phenyl)morpholine (3r)

Colorless liquid, 23.3 mg, 43% yield. **^{1}H NMR** (600 MHz, $CDCl_3$) δ 7.18 – 7.14 (m, 1H), 7.12 – 7.11 (m, 3H), 7.04 – 6.99 (m, 2H), 6.84 (d, J = 12.0Hz, 2H), 3.92 (s, 2H), 3.84 (t, J = 6.0 Hz, 4H), 3.11(t, J = 6.0 Hz, 4H). **^{13}C NMR** (151 MHz, $CDCl_3$) δ 160.9 (d, $J_{C\text{-}F}$ = 245.0 Hz), 149.7, 131.4, 130.9 (d, $J_{C\text{-}F}$ = 4.7 Hz), 129.5, 128.5 (d, $J_{C\text{-}F}$ = 15.9 Hz), 127.7 (d, $J_{C\text{-}F}$ = 8.0 Hz), 124.0 (d, $J_{C\text{-}F}$ = 3.7 Hz), 66.9, 49.6, 33.9 (d, $J_{C\text{-}F}$ = 3.0 Hz). **^{19}F NMR** (376 MHz, $CDCl_3$) δ -118.06 - -118.12 (m, 1F). **IR** (KBr): 3445, 1635, 1515, 756 cm^{-1}. **HRMS** m/z (ESI) calcd. for $C_{17}H_{19}FNO^+$ $[M+H]^+$: 272.1445; found: 272.1446.

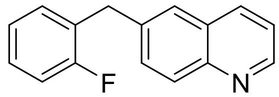

6-(2-fluorobenzyl)quinoline (3s)

Colorless liquid, 34.6 mg, 75% yield. **^{1}H NMR** (600 MHz, $CDCl_3$) δ 8.86 (s, 1H), 8.06 – 8.03 (m, 2H), 7.59 – 7.58 (m, 2H), 7.35 – 7.34 (m, 1H), 7.22 – 7.17 (m, 2H), 7.07 – 7.04 (m, 2H), 4.17 (s, 2H). **^{13}C NMR** (151 MHz, $CDCl_3$) δ 161.0 (d, $J_{C\text{-}F}$ = 245.8 Hz), 149.8, 147.2, 138.3, 135.7, 131.0 (d, $J_{C\text{-}F}$ = 4.4 Hz), 130.9, 129.5, 128.3, 128.2 (d, $J_{C\text{-}F}$ = 8.1 Hz), 127.4 (d, $J_{C\text{-}F}$ = 15.7 Hz), 126.7, 124.2 (d, $J_{C\text{-}F}$ = 3.5 Hz), 121.1, 115.4 (d, $J_{C\text{-}F}$ = 22.0 Hz), 34.8 (d, $J_{C\text{-}F}$ = 2.7 Hz). **^{19}F NMR** (376 MHz, $CDCl_3$) δ -117.66 – -117.73 (m, 1F). **IR** (KBr): 3456, 2065, 1636, 754 cm^{-1}. **HRMS** m/z (ESI) calcd. for $C_{16}H_{13}FN^+$ $[M+H]^+$: 238.1027; found: 238.1028.

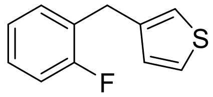

3-(2-fluorobenzyl)thiophene (3t)

Colorless liquid, 22.3 mg, 58% yield. **^{1}H NMR** (600 MHz, $CDCl_3$) δ 7.26 (m, 1H), 7.23 – 7.14 (m, 2H), 7.11 – 7.02 (m, 2H), 6.96 – 6.95 (m, 2H), 4.01 (s, 2H). **^{13}C NMR** (151 MHz, $CDCl_3$) δ 160.9 (d, $J_{C\text{-}F}$ = 245.4 Hz), 140.0, 130.8 (d, $J_{C\text{-}F}$ = 4.5 Hz), 128.3, 128.0 (d, $J_{C\text{-}F}$ = 8.0 Hz), 127.7 (d, $J_{C\text{-}F}$ = 15.7 Hz), 125.6, 124.1 (d, $J_{C\text{-}F}$ = 3.5 Hz), 121.4, 115.3 (d, $J_{C\text{-}F}$ = 21.9 Hz), 29.5 (d, $J_{C\text{-}F}$ = 3.1 Hz). **^{19}F NMR** (376 MHz, $CDCl_3$) δ -118.13 – -118.38 (m, 1F). **IR** (KBr): 3444, 1639, 753 cm^{-1}. **MS** (EI, m/z%): 192.05 ($M^{•+}$, 100.00), 97.04 (85.36), 191.04 (79.12), 96.04 (17.77), 83.04 (16.72), 193.06 (15.46), 159.07 (12.88), 133.07 (12.03), 109.07 (11.41), 147.08 (11.20), 85.08 (10.24),

146.08 (9.82), 171.06 (8.57), 69.03 (8.39), 63.07 (7.99). **HRMS** (EI) calcd. for $C_{11}H_{19}FS^{\bullet+}$ $[M]^{\bullet+}$: 192.0404; Found: 192.0403.

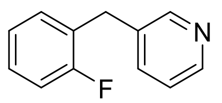

3-(2-fluorobenzyl)pyridine (**3u**)

Colorless liquid, 22.4 mg, 60% yield. **^{1}H NMR** (600 MHz, $CDCl_3$) δ 8.52 (s, 1H), 8.45 (d, J = 4.2 Hz, 1H), 7.50 (d, J = 7.9 Hz, 1H), 7.24 – 7.17 (m, 2H), 7.14 (dd, J = 7.5, 6.3 Hz, 1H), 7.09 – 7.01 (m, 2H), 3.99 (s, 2H). **^{13}C NMR** (151 MHz, $CDCl_3$) δ 160.9 (d, $J_{C\text{-}F}$ = 245.5 Hz), 150.0, 147.7, 136.2, 135.4, 130.8 (d, $J_{C\text{-}F}$ = 4.1 Hz), 128.5 (d, $J_{C\text{-}F}$ = 8.0 Hz), 126.8 (d, $J_{C\text{-}F}$ = 15.8 Hz), 124.3 (d, $J_{C\text{-}F}$ = 3.4 Hz), 123.4, 115.5 (d, $J_{C\text{-}F}$ = 21.9 Hz), 32.2 (d, $J_{C\text{-}F}$ = 2.8 Hz). **^{19}F NMR** (376 MHz, $CDCl_3$) δ -117.59 – 117.65 (m, 1F). **IR** (KBr): 3444, 2077, 1636, 754 cm^{-1}. **HRMS** m/z (ESI) calcd. for $C_{12}H_{11}FN^+$ $[M+H]^+$: 188.0870; found: 188.0871.

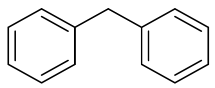

Diphenylmethane (**3v**)

Colorless liquid, 16.8 mg, 60% yield. **^{1}H NMR** (300 MHz, $CDCl_3$) δ 7.30 – 7.17 (m, 4H), 7.2 – 7.1 (m, 6H), 4.0 (s, 2H). **^{13}C NMR** (75 MHz, $CDCl_3$) δ 141.1, 128.9, 128.4, 126.0, 41.9.

^{1}H and ^{13}C NMR data agreed with the literature[12]

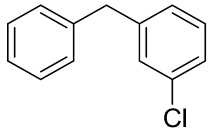

1-benzyl-3-chlorobenzene (**3w**)

Colorless liquid, 25.0 mg, 67% yield. **^{1}H NMR** (600 MHz, $CDCl_3$) δ 7.31 (t, J = 7.6 Hz, 2H), 7.25-7.18 (m, 6H), 7.08 (d, J = 7.2 Hz, 2H), 7.08 (d, J = 7.2 Hz, 1H), 3.96 (s, 2H). **^{13}C NMR** (151 MHz, $CDCl_3$) δ 143.2, 140.2, 134.3, 129.7, 129.0, 128.9, 128.6, 127.09, 126.4, 126.3, 41.6 **IR** (KBr): 3449, 2082, 1636, 681 cm^{-1}. **MS** (EI, m/z%): 202

($M^{\bullet+}$, 38.27), 167 (100), 165 (48.26), 81 (28.11), 166 (22.36), 152 (18.24), 82 (15.39). **HRMS** (EI) calcd. for $C_{13}H_{11}Cl^{\bullet+}$ $[M]^{\bullet+}$: 202.0544; Found: 202.0543.

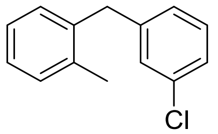

1-(3-chlorobenzyl)-2-methylbenzene (**3x**)

Colorless liquid, 28.5 mg, 66% yield. **^{1}H NMR** (400 MHz, $CDCl_3$) δ 7.19 – 7.13 (m, 5H), 7.11 – 7.06 (m, 2H), 7.01 – 6.97 (m, 1H), 3.95 (s, 2H), 2.22 (s, 3H). **^{13}C NMR** (101 MHz, $CDCl_3$) δ 142.5, 138.0, 136.6, 134.2, 130.4, 130.0, 129.6, 128.8, 126.9, 126.8, 126.2, 126.1, 39.1, 19.6. **MS** (EI, m/z%): 216.03 ($M^{\bullet+}$, 76.82), 181.06 (100.00), 165.06 (82.56), 166.08 (79.61), 89.09 (66.98), 201.02 (43.68), 104.05 (43.56), 76.07 (31.26), 218.04 (25.24), 77.06 (22.90), 91.07 (21.05), 178.07 (17.61), 105.09 (16.58), 63.05 (16.12), 179.06 (15.70). **IR** (KBr): 3454, 2077, 1637, 740 cm^{-1}. **HRMS** (EI) calcd. for $C_{14}H_{13}Cl^{\bullet+}$ $[M]^{\bullet+}$: 216.0700; Found: 216.0700.

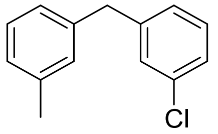

1-chloro-3-(3-methylbenzyl)benzene (**3y**)

Colorless liquid, 26.9 mg, 62% yield. **^{1}H NMR** (400 MHz, $CDCl_3$) δ 7.24 – 7.13 (m, 4H), 7.09 – 7.00 (m, 2H), 7.00 – 6.94 (m, 2H), 3.90 (s, 2H), 2.31 (s, 3H). **^{13}C NMR** (101 MHz, $CDCl_3$) δ 143.3, 140.1, 138.2, 134.2, 129.7, 129.6, 129.0, 128.5, 127.1, 127.1, 126.2, 125.9, 41.5, 21.4. **IR** (KBr): 3444, 2077, 1636, 682 cm^{-1}. **MS** (EI, m/z%): 216.04 ($M^{\bullet+}$, 67.10), 181.08 (100.00), 166.05 (73.62), 165.07 (72.24), 89.14 (60.99), 201.02 (32.00), 76.08 (26.69), 218.02 (21.15), 77.04 (17.63), 179.06 (14.28), 178.07 (14.04), 182.09 (13.82), 63.08 (13.58), 180.09 (12.29), 105.06 (11.28). **HRMS** (EI) calcd. for $C_{14}H_{13}Cl^{\bullet+}$ $[M]^{\bullet+}$: 216.0700; Found: 216.0699.

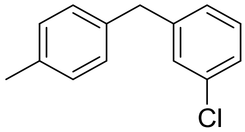

1-chloro-3-(4-methylbenzyl)benzene (**3z**)

Colorless liquid, 28.9 mg, 67% yield. **^{1}H NMR** (400 MHz, $CDCl_3$) δ 7.28 – 7.13 (m, 3H), 7.13 – 7.00 (m, 5H), 3.90 (s, 2H), 2.31 (s, 3H). **^{13}C NMR** (101 MHz, $CDCl_3$) δ 143.4, 137.1, 135.9, 134.2, 129.6, 129.3, 128.9, 128.8, 127.0, 126.2, 41.1, 21.0. **IR** (KBr): 3440, 2078, 1635, 672 cm^{-1}. **MS** (EI, m/z%): 216.05 ($M^{\bullet+}$, 70.88), 181.09 (100.00), 166.06 (79.21), 165.05 (77.31), 89.09 (52.73), 201.02 (39.55), 76.08 (25.91), 105.10 (16.85), 63.04 (14.38), 178.07 (14.37), 203.02 (13.17), 179.08 (13.06), 182.09 (12.92), 180.11 (12.50), 91.08 (11.33). **HRMS** (EI) calcd. for $C_{14}H_{13}Cl^{\bullet+}$ $[M]^{\bullet+}$: 216.0700; Found: 216.0698.

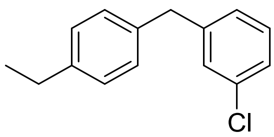

1-chloro-3-(4-ethylbenzyl) benzene (**3aa**)

Colorless liquid, 30.0 mg, 65% yield. **^{1}H NMR** (400 MHz, $CDCl_3$) δ 7.23 – 7.03 (m, 8H), 3.90 (s, 2H), 2.61 (q, *J* = 7.6 Hz, 2H), 1.22 (t, *J* = 7.6 Hz, 3H). **^{13}C NMR** (101 MHz, $CDCl_3$) δ 143.4, 142.3, 137.4, 134.2, 129.6, 129.0, 128.8, 128.1, 127.1, 126.2, 41.2, 28.4, 15.6. **IR** (KBr): 3442, 2078, 1635, 1245, 694 cm^{-1}. **MS** (EI, m/z%): 230.07 ($M^{\bullet+}$, 66.04), 201.02 (100.00), 165.07 (66.69), 166.10 (50.22), 105.09 (47.13), 89.07 (33.88), 203.03 (31.99), 195.11 (20.91), 178.09 (19.36), 232.06 (19.30), 179.07 (17.57), 215.00 (16.35), 167.08 (15.12). **HRMS** (EI) calcd. for $C_{15}H_{15}Cl^{\bullet+}$ $[M]^{\bullet+}$: 230.0857; Found: 230.0855.

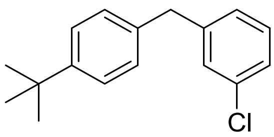

1-(4-(tert-butyl)benzyl)-3-chlorobenzene (**3ab**)

Colorless liquid, 31.5 mg, 61% yield. **^{1}H NMR** (400 MHz, $CDCl_3$) δ 7.34 – 7.28 (m, 2H), 7.24 – 7.14 (m, 3H), 7.12 – 7.05 (m, 3H), 3.91 (s, 2H), 1.30 (s, 9H). **^{13}C NMR** (101 MHz, $CDCl_3$) δ 149.2, 143.3, 137.1, 134.2, 129.6, 129.0, 128.5, 127.1, 126.2, 125.5, 41.1, 34.4, 31.4. **IR** (KBr): 3447, 2077, 1636, 683 cm^{-1}. **MS** (EI, m/z%): 258.10 ($M^{\bullet+}$, 27.79), 243.05 (100.00), 125.04 (74.80), 89.07 (45.44), 245.07 (35.17), 90.08 (33.20), 127.05 (23.64), 244.09 (19.97), 165.09 (17.80), 91.03 (15.99), 115.06 (9.41),

260.11 (9.27), 105.07 (8.86), 76.07 (8.84), 77.05 (6.67). **HRMS** (EI) calcd. for $C_{17}H_{19}Cl^{•+}$ $[M]^{•+}$: 258.1170; Found: 258.1171.

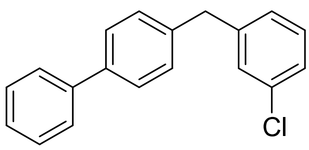

4-(3-chlorobenzyl)-1,1'-biphenyl (3ac)

White solid, m.p. 45-46 °C, 40.1 mg, 72% yield. **^{1}H NMR** (400 MHz, $CDCl_3$) δ 7.62 – 7.49 (m, 4H), 7.45 – 7.41 (m, 2H), 7.36 – 7.72 (m, 1H), 7.28 – 7.17 (m, 5H), 7.14 – 7.08 (m, 1H), 4.00 (s, 2H). **^{13}C NMR** (101 MHz, $CDCl_3$) δ 143.0, 140.9, 139.4, 139.3, 134.3, 129.7, 129.3, 129.0, 128.7, 127.3, 127.2, 127.1, 127.0, 126.4, 41.2. **IR** (KBr): 3438, 2077, 1636, 672 cm^{-1}. **MS** (EI, m/z%): 278.03 ($M^{•+}$, 85.72), 165.04 (100.00), 243.10 (50.01), 280.02 (30.74), 167.08 (28.30), 166.07 (28.16), 91.09 (24.35), 119.72 (23.48), 115.07 (23.03), 241.06 (20.66), 152.07 (20.12), 206.99 (19.45), 279.03 (18.41), 239.05 (16.28), 242.10 (16.64), 89.09 (15.22). **HRMS** (EI) calcd. for $C_{19}H_{15}Cl^{•+}$ $[M]^{•+}$: 278.0857; Found: 278.0854.

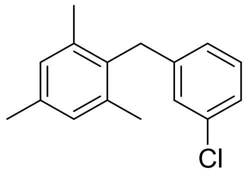

2-(3-chlorobenzyl)-1,3,5-trimethylbenzene (3ad)

Colorless liquid, 32.3 mg, 66% yield. **^{1}H NMR** (400 MHz, $CDCl_3$) δ 7.17 – 7.13 (m, 2H), 6.99 (s, 1H), 6.89 – 6.87 (m,3H), 3.99 (s, 2H), 2.29 (s, 3H), 2.19 (s, 6H). **^{13}C NMR** (101 MHz, $CDCl_3$) δ 142.3, 136.9, 136.0, 134.2, 132.8, 129.5, 129.0, 127.9, 126.0, 125.9, 34.4, 20.9, 20.1. **IR** (KBr): 3438, 1635, 677 cm^{-1}. **MS** (EI, m/z%): 244.08 ($M^{•+}$, 90.05), 229.08 (100.00), 194.08 (54.65), 133.15 (47.48), 179.08 (45.42), 119.09 (42.65), 231.08 (36.44), 178.10 (36.09), 91.12 (33.04), 132.12 (31.49), 89.08 (30.51), 193.09 (30.49), 246.07 (26.97), 115.09 (25.27), 77.08 (22.18). **HRMS** (EI) calcd. for $C_{16}H_{17}Cl^{•+}$ $[M]^{•+}$: 244.1013; Found: 244.1013.

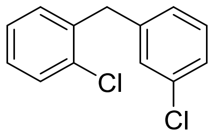

1-chloro-2-(3-chlorobenzyl)benzene (**3ae**)

Colorless liquid, 34.7 mg, 73.5% yield. **^{1}H NMR** (600 MHz, $CDCl_3$) δ 7.40 – 7.34 (m, 1H), 7.25 – 7.11 (m, 6H), 7.06 (d, J = 7.0 Hz, 1H), 4.06 (s, 2H). **^{13}C NMR** (151 MHz, $CDCl_3$) δ 141.6, 137.8, 134.3, 134.2, 131.0, 129.7 (2C), 128.9, 127.96, 127.1, 126.9, 126.5, 38.8. **IR** (KBr): 3451, 2077, 1638, 751 cm^{-1}. **MS** (EI, m/z%): 235.97 ($M^{\bullet+}$, 53.53), 165.05 (100.00), 165.06 (86.31), 82.03 (81.08), 201.00(77.92), 203.02 (26.40), 89.06 (23.68), 237.96 (23.22), 82.77 (18.62), 81.12 (17.83), 63.04 (16.78), 164.07 (15.56), 163.04 (14.97), 124.99 (13.18), 75.06 (12.30). **HRMS** (EI) calcd. for $C_{13}H_{10}Cl_2^{\bullet+}$ $[M]^{\bullet+}$: 236.0154; Found: 236.0153.

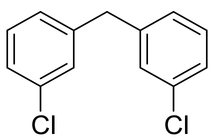

Bis(3-chlorophenyl)methane (**3af**)

Colorless liquid, 31.2mg, 66% yield. **^{1}H NMR** (600 MHz, $CDCl_3$) δ 7.24 – 7.17 (m, 4H), 7.16 – 7.14 (m, 2H), 7.06 – 7.03 (m, 2H), 3.90 (s, 2H). **^{13}C NMR** (101 MHz, $CDCl_3$) δ 142.2, 134.4, 129.8, 129.0 127.1, 126.6, 41.1. **IR** (KBr): 3449, 2082, 1636, 681 cm^{-1}. **MS** (EI, m/z%): 235.96 ($M^{\bullet+}$, 39.35), 165.06 (100.00), 166.07 (84.41), 201.01 (82.38), 82.04 (72.47), 237.96 (24.97), 89.05 (24.15), 81.18 (19.77), 82.77 (17.17), 63.03 (15.98), 164.08 (13.15), 75.04 (13.13), 125.01 (12.82), 202.03 (12.43), 163.05 (12.26). **HRMS** (EI) calcd. for $C_{13}H_{10}Cl_2^{\bullet+}$ $[M]^{\bullet+}$: 236.0154; Found: 236.0152.

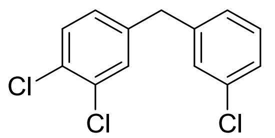

1,2-dichloro-4-(3-chlorobenzyl)benzene (**3ag**)

Colorless liquid, 30.2 mg, 56% yield. **^{1}H NMR** (600 MHz, $CDCl_3$) δ 7.35 (dd, J = 8.2, 1.4 Hz, 1H), 7.25 – 7.20 (m, 3H), 7.14 (s, 1H), 7.03 (d, J = 7.0 Hz, 1H), 6.99 (d, J = 8.2 Hz, 1H), 3.89 (s, 2H). **^{13}C NMR** (151 MHz, $CDCl_3$) δ 141.7, 140.4, 134.6, 132.6, 130.8, 130.6, 130.5, 129.9, 129.0, 128.3, 127.0, 126.9, 40.6. **IR** (KBr): 3434, 1635, 663 cm^{-1}.

MS (EI, m/z%): 269.96 ($M^{\bullet+}$, 30.82), 165.08 (100.00), 234.98 (80.34), 236.98 (48.01), 199.03 (41.90), 81.75 (37.84), 99.10 (34.31), 271.95 (30.58), 200.04 (30.09), 81.10 (26.98), 99.70 (23.21), 163.07 (23.19), 89.06 (20.39), 201.04 (17.63). **HRMS** (EI) calcd. for $C_{13}H_{19}Cl_3^{\bullet+}$ $[M]^{\bullet+}$: 269.9764; Found: 269.9763.

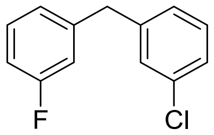

1-chloro-3-(3-fluorobenzyl)benzene (3ah)

Colorless liquid, 28.2 mg, 64% yield. **^{1}H NMR** (400 MHz, $CDCl_3$) δ 7.33 – 7.17 (m, 4H), 7.09 (d, J = 6.7 Hz, 1H), 7.03 – 6.84 (m, 3H), 3.97 (s, 2H). **^{13}C NMR** (101 MHz, $CDCl_3$) δ 163.0 (d, J_{C-F} = 246.0 Hz), 142.7 (d, J_{C-F} = 7.1 Hz), 142.3, 134.4, 130.0 (d, J_{C-F} = 8.3 Hz), 129.8, 129.0, 127.1, 126.6, 124.5 (d, J_{C-F} = 2.8 Hz), 115.8 (d, J_{C-F} = 21.3 Hz), 113.3 (d, J_{C-F} = 21.1 Hz), 41.2 (d, J_{C-F} = 1.7 Hz). **^{19}F NMR** (376 MHz, $CDCl_3$) δ -105.62 – -116.99 (m). **IR** (KBr): 3441, 2078, 1635, 1247, 682 cm^{-1}. **MS** (EI, m/z%): 220.00 ($M^{\bullet+}$, 53.53), 185.07 (100.00), 165.06 (48.18), 183.04 (43.76), 184.06 (20.07), 222.02 (16.54), 82.04 (13.37), 91.76 (13.29), 186.09 (12.60), 109.03 (9.39), 83.02 (9.28). **HRMS** (EI) calcd. for $C_{13}H_{10}ClF^{\bullet+}$ $[M]^{\bullet+}$: 220.0450; Found: 220.0449.

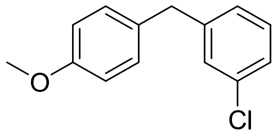

1-chloro-3-(4-methoxybenzyl)benzene (3ai)

Colorless liquid, 26.0 mg, 56% yield. **^{1}H NMR** (400 MHz, $CDCl_3$) δ 7.23 – 7.13 (m, 3H), 7.12 – 7.02 (m, 3H), 6.88 – 6.79 (m, 2H), 3.88 (s, 2H), 3.78 (s, 3H). **^{13}C NMR** (101 MHz, $CDCl_3$) δ 158.1, 143.6, 134.2, 132.3, 129.9, 129.6, 128.9, 126.9, 126.2, 114.0, 55.2, 40.7. **IR** (KBr): 3443, 2077, 1636, 1510, 1245, 681 cm^{-1}. **HRMS** m/z (ESI) calcd. for $C_{14}H_{14}ClO^+$ $[M+H]^+$: 233.0728; found: 233.0726.

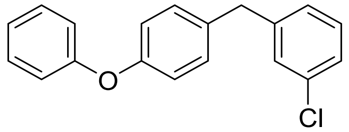

1-chloro-3-(4-phenoxybenzyl)benzene (3aj)

Colorless liquid, 35.9 mg, 61% yield. **^{1}H NMR** (400 MHz, $CDCl_3$) δ 7.32 (dd, *J* = 8.5, 7.5 Hz, 2H), 7.26 – 7.15 (m, 3H), 7.15 – 7.03 (m, 4H), 7.03 – 6.97 (m, 2H), 6.94 (d, *J* = 8.6 Hz, 2H), 3.92 (s, 2H). **^{13}C NMR** (101 MHz, $CDCl_3$) δ 157.3, 155.7, 143.2, 135.1, 134.3, 130.1, 129.7 (2C), 129.0, 127.0, 126.3, 123.1, 119.0, 118.7, 40.8. **IR** (KBr): 3439, 2077, 1636, 1238, 678 cm^{-1}. **MS** (EI, m/z%): 294.03 ($M^{\bullet+}$, 59.29), 165.06 (100.00), 166.08 (86.22), 77.05 (53.92), 201.06 (51.87), 51.06 (31.44), 153.09 (23.99), 152.07 (23.38), 296.03 (19.95), 259.10 (18.98), 183.05 (17.36), 89.08 (17.22), 107.08 (10.26). **HRMS** (EI) calcd. for $C_{19}H_{15}ClO^{\bullet+}$ $[M]^{\bullet+}$: 294.0806; Found: 294.0806.

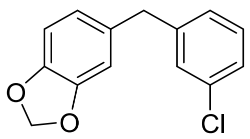

5-(3-chlorobenzyl)benzo[d][1,3]dioxole (3ak)

Colorless liquid, 24.6 mg, 50% yield. **^{1}H NMR** (400 MHz, $CDCl_3$) δ 7.29 – 7.12 (m, 3H), 7.05 (d, *J* = 7.1 Hz, 1H), 6.74 (d, *J* = 8.4 Hz, 1H), 6.64 (d, *J* = 7.2 Hz, 2H), 5.92 (s, 2H), 3.85 (s, 2H). **^{13}C NMR** (101 MHz, $CDCl_3$) δ 147.8, 146.1, 143.3, 134.2, 134.0, 129.7, 128.8, 126.9, 126.3, 121.8, 109.3, 108.3, 100.9, 41.2. **IR** (KBr): 3444, 2077, 1636, 682 cm^{-1}. **HRMS** m/z (ESI) calcd. for $C_{14}H_{12}ClO_2^+$ $[M+H]^+$: 247.0502; found: 247.0529.

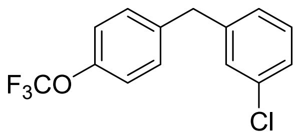

1-chloro-3-(4-(trifluoromethoxy)benzyl)benzene (3al)

Colorless liquid, 42.9 mg, 75% yield. **^{1}H NMR** (400 MHz, $CDCl_3$) δ 7.25 – 7.11 (m, 7H), 7.05 (dt, *J* = 7.0, 1.7 Hz, 1H), 3.94 (s, 2H). **^{13}C NM**R (101 MHz, $CDCl_3$) δ 147.8, 142.4, 138.9, 134.4, 130.1, 129.8, 129.0, 127.1, 126.6, 121.1, 120.16 ($J_{C\text{-}F}$ = 257.6) 40.8. **^{19}F NMR** (376 MHz, $CDCl_3$) δ -57.90 (s, 3F). **IR** (KBr): 3442, 1634, 1254, 681 cm^{-1}. **MS** (EI, m/z%): 285.98 ($M^{\bullet+}$, 47.47), 165.07 (100.00), 251.04 (83.94), 166.08 (37.22), 152.05 (26.07), 201.03 (20.27), 153.07 (17.78), 69.06 (17.53), 287.99 (16.00), 252.06 (13.68), 89.05 (12.62), 63.06 (10.67), 175.02 (9.34). **HRMS** (EI) calcd. for $C_{14}H_{10}ClF_3O^{\bullet+}$ $[M]^{\bullet+}$: 286.0367; Found: 286.0363.

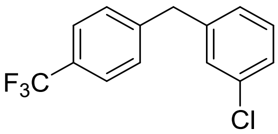

1-chloro-3-(4-(trifluoromethyl)benzyl)benzene (3am)

Colorless liquid, 28.1 mg, 52% yield. **^{1}H NMR** (600 MHz, $CDCl_3$) δ 7.54 (d, J = 7.7 Hz, 2H), 7.27 (d, J = 7.7 Hz, 2H), 7.24 – 7.19 (m, 2H), 7.16 (s, 1H), 7.04 (d, J = 6.7 Hz, 1H), 3.99 (s, 2H). **^{13}C NMR** (151 MHz, $CDCl_3$) δ 144.3, 142.0, 134.5, 129.9, 129.2 (2C), 129.0, 127.1, 126.7, 126.2 (q, J_{C-F} = 271.8 Hz) 125.6 (q, J_{C-F} = 3.5 Hz), 41.3. **^{19}F NMR** (376 MHz, $CDCl_3$) δ -62.40 (s, 3F). **IR** (KBr): 3436, 2077, 1635, 681 cm^{-1}. **MS** (EI, m/z%): 270.00 ($M^{•+}$, 51.03), 235.03 (100.00), 165.06 (77.86), 166.09 (62.64), 215.04 (25.49), 201.04 (22.20), 271.99 (17.04), 89.06 (15.59), 107.07 (14.42), 236.06 (14.07), 233.04 (11.61), 75.04 (11.35), 63.06 (11.24), 81.44 (9.40), 125.02 (9.35). **HRMS** (EI) calcd. for $C_{14}H_{10}ClF_3^{•+}$ $[[M]^{•+}$: 270.0418; Found: 270.0414.

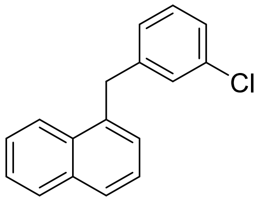

1-(3-chlorobenzyl)naphthalene (3an)

Yelow oil, 25.2 mg, 50% yield. **^{1}H NMR** (600 MHz, $CDCl_3$) δ 7.91 (d, J = 7.7 Hz, 1H), 7.85 (d, J = 6.8 Hz, 1H), 7.77 (d, J = 8.0 Hz, 1H), 7.50 – 7.38 (m, 3H), 7.28 (d, J = 6.6 Hz, 1H), 7.20 – 7.13 (m, 3H), 7.05 (d, J = 5.5 Hz, 1H), 4.39 (s, 2H). **^{13}C NMR** (151 MHz, $CDCl_3$) δ 142.8, 135.7, 134.3, 134.0, 132.0, 129.7, 128.8 (2C), 127.5 (2C), 126.7, 126.32, 126.1, 125.7, 125.5, 124.1, 38.7. **IR** (KBr): 3452, 2088, 1639, 773 cm^{-1}.

MS (EI, m/z%): 252.05 ($M^{•+}$, 86.96), 217.10 (100.00), 215.10 (71.77), 107.75 (46.57), 106.60 (43.66), 202.08 (43.44), 115.07 (40.56), 216.11 (40.04), 94.59 (36.46), 141.09 (33,89), 107.24 (30.54), 108.27(25.67), 254.05 (23.95), 218.13 (17.89), 253.08 (16.93), 213.07 (15.20). **HRMS** (EI) calcd. for $C_{17}H_{13}Cl^{•+}$ $[M]^{•+}$: 252.0700; Found: 252.0698.

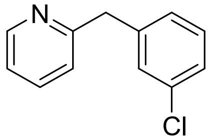

2-(3-chlorobenzyl)pyridine (3ao)

Colorless liquid, 24.8 mg, 61% yield. **^{1}H NMR** (600 MHz, $CDCl_3$) δ 8.55 (d, *J* = 4.0 Hz, 1H), 7.59 (t, *J* = 7.5 Hz, 1H), 7.26 – 7.25 (m, 1H), 7.23 – 7.18 (m, 2H), 7.16 – 7.09 (m, 3H), 4.12 (s, 2H). **^{13}C NMR** (151 MHz, $CDCl_3$) δ 160.1, 149.5, 141.5, 136.6, 134.3, 129.8, 129.2, 127.3, 126.6, 123.1, 121.5, 44.3. **IR** (KBr): 3447, 2078, 1635, 748 cm^{-1}. **HRMS** m/z (ESI) calcd. for $C_{12}H_{11}ClN^+$ $[M+H]^+$: 204.0575; found: 204.0577.

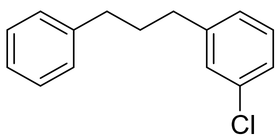

1-chloro-3-(3-phenylpropyl)benzene (3ap)

Colorless liquid, 20.0 mg, 39% yield. **^{1}H NMR** (300 MHz, $CDCl_3$) δ 7.31-7.24 (m ,2H), 7.19-7.17 (m, 6H), 7.06-7.04 (m, 1H), 2.67-2.59 (m, 4H), 2.01 – 1.85 (m, 2H). **^{13}C NMR** (75 MHz, $CDCl_3$) δ 144.3, 141.9, 134.0, 129.5, 128.5, 128.4, 128.3, 126.6, 125.9, 125.8, 35.3, 35.0, 32.7. **IR** (KBr): 2934, 1597, 1097, 697 cm^{-1}. **MS** (EI, m/z%): 230.08 ($M^{\bullet+}$, 21.32), 91.10 (100.00), 92.10 (60.72), 126.04 (38.58), 105.10 (24.50), 77.06 (20.73), 103.08 (16.49), 65.05 (15.25), 128.03 (9.93), 79.07 (8.75), 89.04 (8.14), 232.06 (7.53), 139.06 (7.52), 125.00 (6.44), 51.03 (6.18). **HRMS** (EI) calcd. for $C_{15}H_{15}Cl^{\bullet+}$ $[M]^{\bullet+}$: 230.0857; Found: 230.0857.

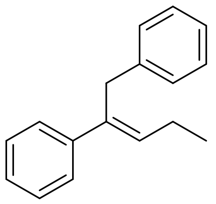

(Z/E)-pent-2-ene-1,2-diyldibenzene (6)

Colorless liquid, 4.9 mg, 11% yield. **^{1}H NMR** (600 MHz, $CDCl_3$) δ 7.35 – 7.32 (m, 1H), 7.25 – 7.22 (m, 4H), 7.19 – 7.13 (m, 4H), 5.97 (t, *J* = 7.2 Hz, 1H), 3.88 (s, 2H), 2.29 – 2.24 (m, 2H), 1.08 (t, *J* = 7.5 Hz, 3H). **^{13}C NMR** (151 MHz, $CDCl_3$) δ 143.04, 139.96, 136.84, 132.73, 128.33, 128.17, 128.14, 126.53, 126.26, 125.78, 35.75, 22.29, 14.17. **IR** (KBr): 3026, 2964, 2918, 1600, 1493, 1451, 752, 722, 696 cm^{-1}. **HRMS** (EI) calcd. for $C_{17}H_{18}^{\bullet+}$ $[M]^{\bullet+}$: 222.1403; Found: 222.1402.

9. References

[1] A. D. Becke, *J. Chem. Phys.,* **1993**, *98*, 5648-5652.

[2] C. Lee, W. Yang, R. G. Parr, *Phys. Rev. B Condens. Matter,* **1988**, *37*, 785-789.

[3] S. Grimme, S. Ehrlich, L. Goerigk, *J Comput. Chem.,* **2011**, *32*, 1456-1465.

[4] D. Andrae, U. Huermann, M. Dolg, H. Stoll, H. Preu, *Theor. Chim. Acta,* **1990**, *77*, 123-141.

[5] A. V. Marenich, C. J. Cramer, D. G. Truhlar, *J. Phys. Chem. B,* **2009**, *113*, 6378-6396.

[6] Y. Zhao, D. G. Truhlar, *Theo. Chem. Acc.,* **2007**, *120*, 215-241.

[7] S. Grimme, *J. Comput. Chem.,* **2004**, *25*, 1463-1473.

[8] W. Humphrey, A. Dalke, K. Schulten, *J. Mol. Graph.,* **1996**, *14*, 33-38.

[9] T. Lu, F. Chen, *J. Comput. Chem.,* **2012**, *33*, 580-592.

[10] CYLview, 1.0b Université de Sherbrooke, **2009** (http://www.cylview.org).

[11] X. Mo, J. Yakiwchuk, J. Dansereau, J. A. McCubbin, D. G. Hall, *J. Am. Chem. Soc.,* **2015**, *137*, 9694-9703.

[12] Y.-Y. Sun, J. Yi, X. Lu, Z.-Q. Zhang, B. Xiao, Y. Fu, *Chem. Commun.,* **2014**, *50*, 11060-11062.

10. Copies of ^{1}H NMR, ^{19}F NMR and ^{13}C NMR spectra

^{1}H, ^{19}F and ^{13}C NMR spectra of compound **3a**

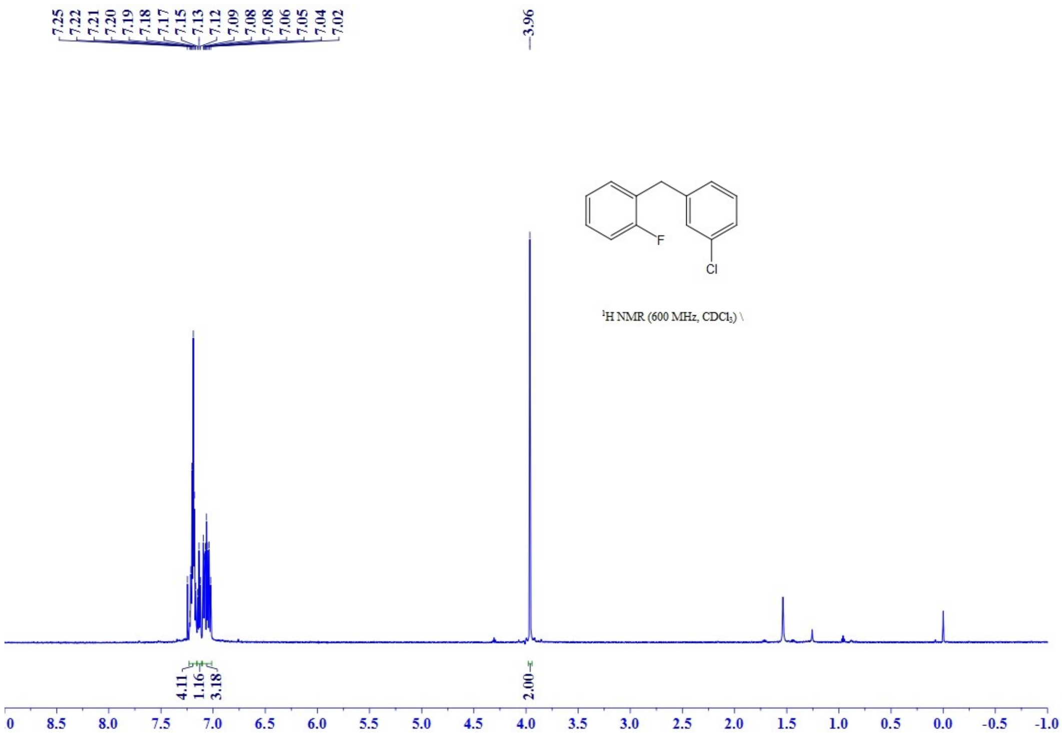

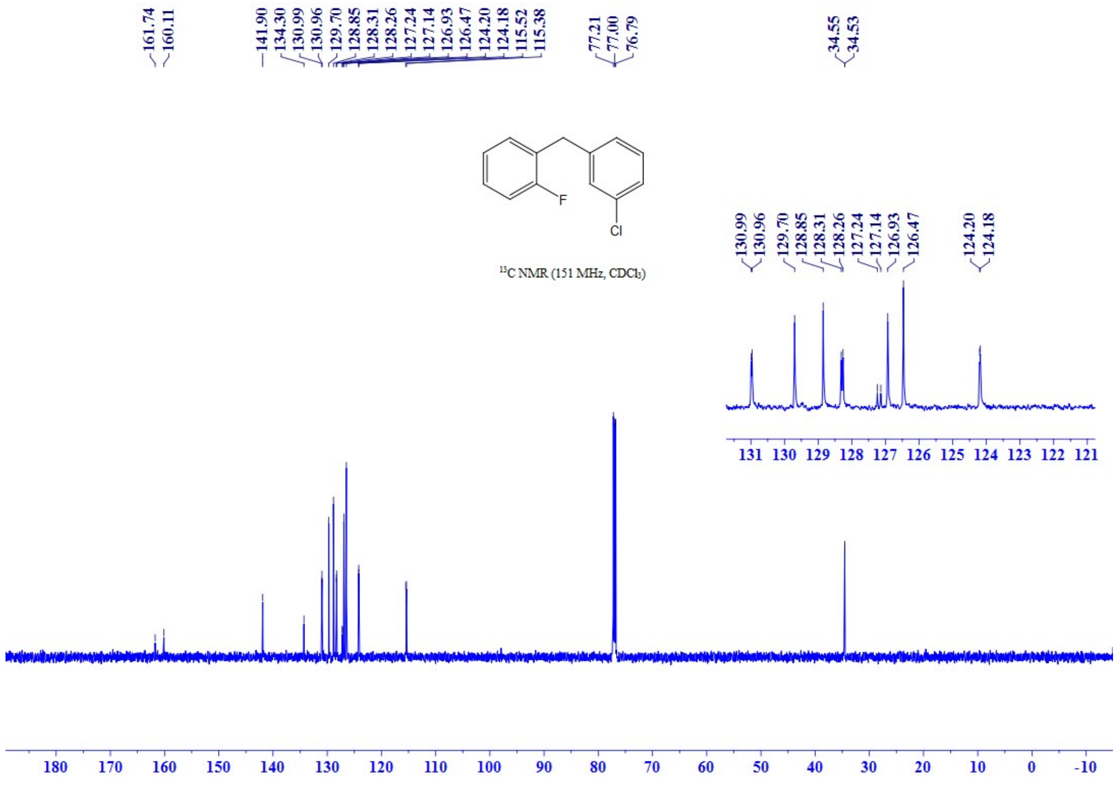

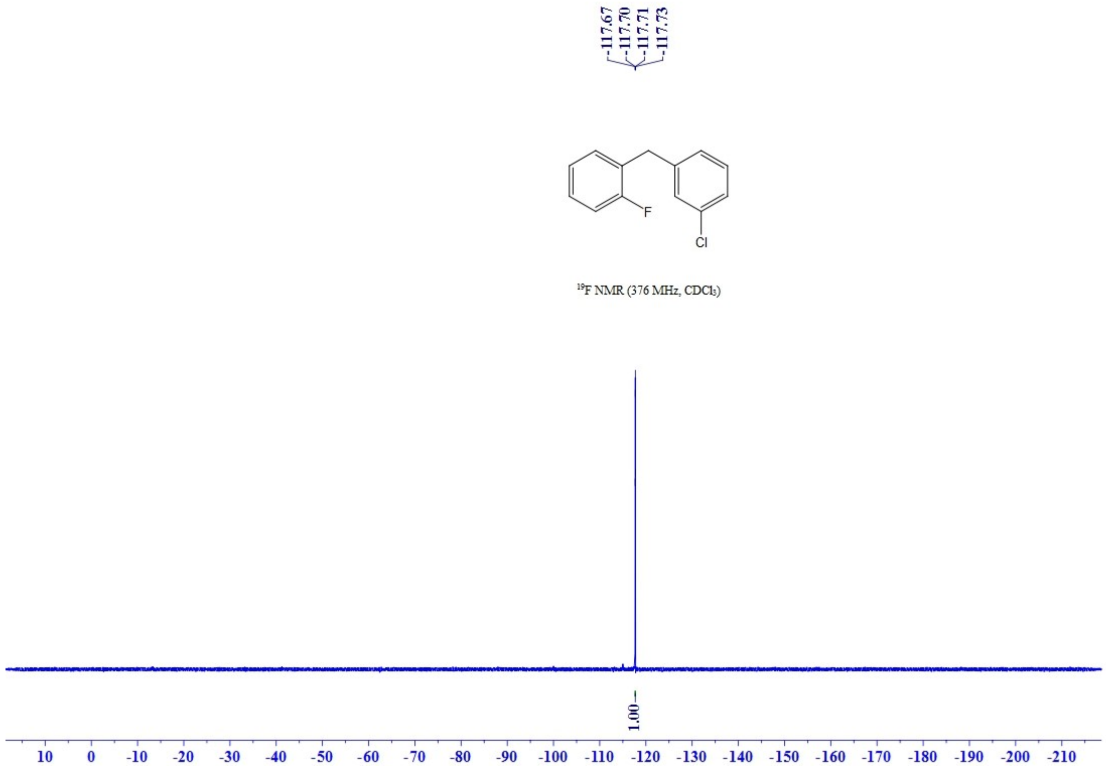

^{1}H, ^{19}F and ^{13}C NMR spectra of compound **3b**

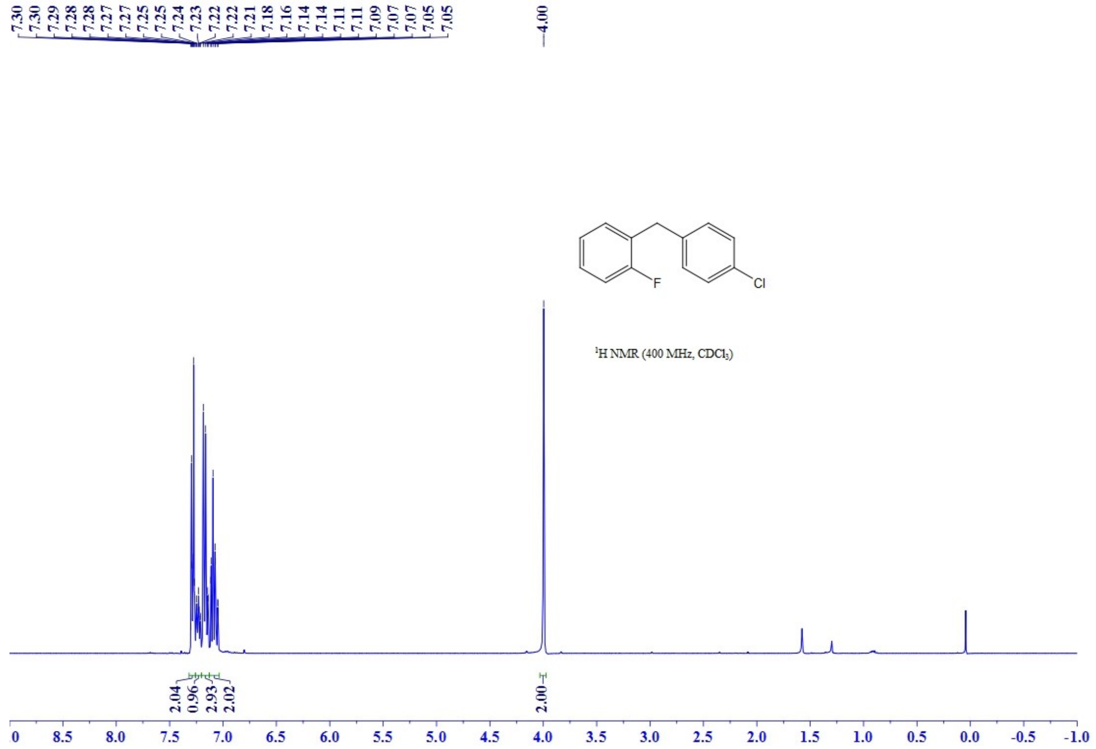

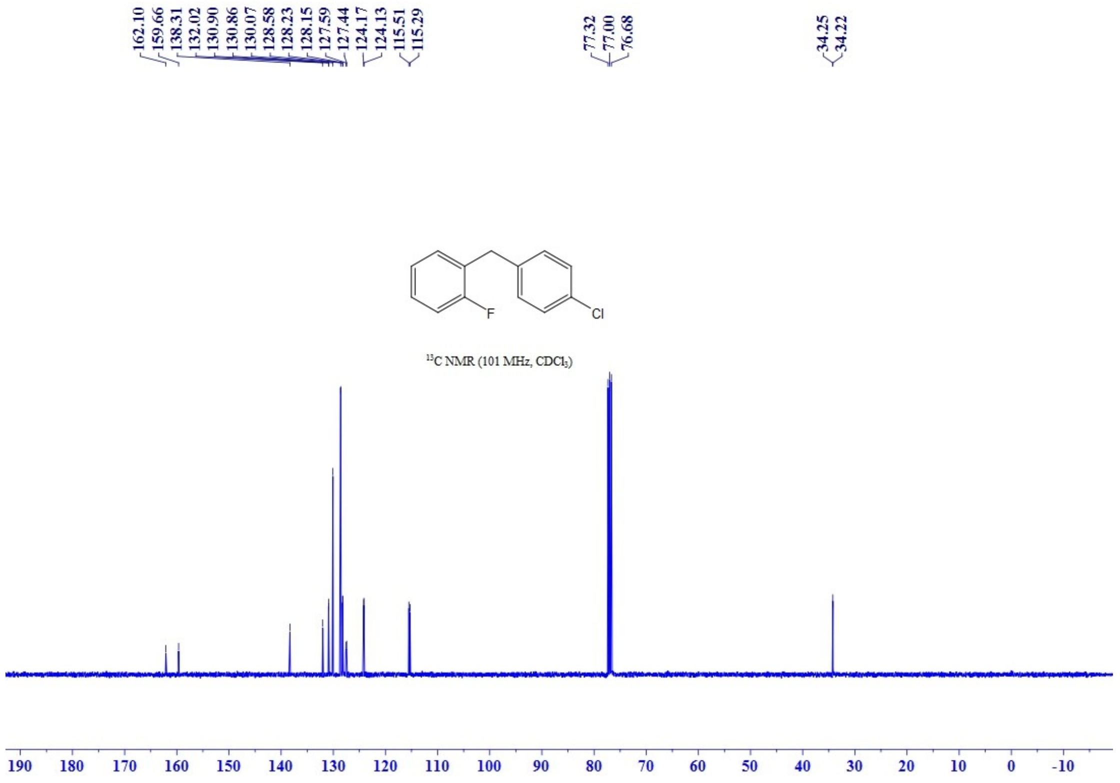
162.10
159.66
138.31
132.02
130.90
130.86
130.07
128.58
128.23
128.15
127.59
127.44
124.17
124.13
115.51
115.29
77.32
77.00
76.68
34.25
34.22
F
Cl
13C NMR (101 MHz, CDCl3)
190 180 170 160 150 140 130 120 110 100 90 80 70 60 50 40 30 20 10 0 -10

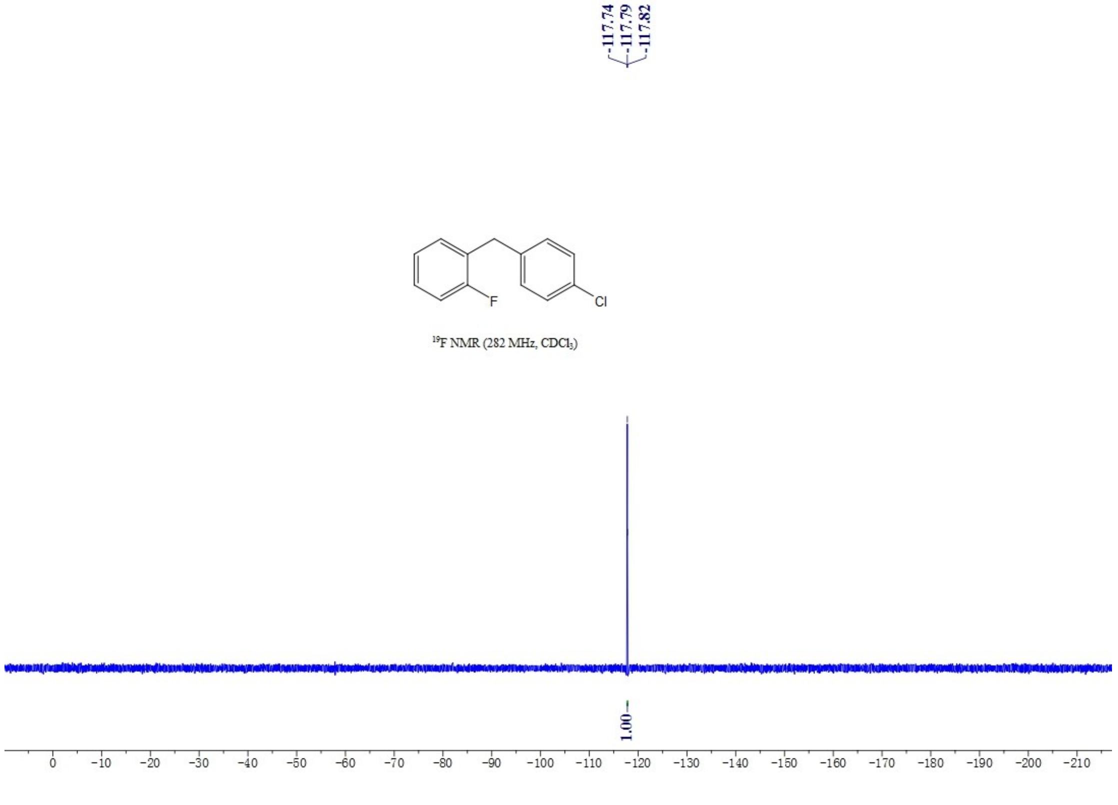
-117.74
-117.79
-117.82
F
Cl
19F NMR (282 MHz, CDCl3)
1.00
0 -10 -20 -30 -40 -50 -60 -70 -80 -90 -100 -110 -120 -130 -140 -150 -160 -170 -180 -190 -200 -210

^{1}H, ^{19}F and ^{13}C NMR spectra of compound **3c**

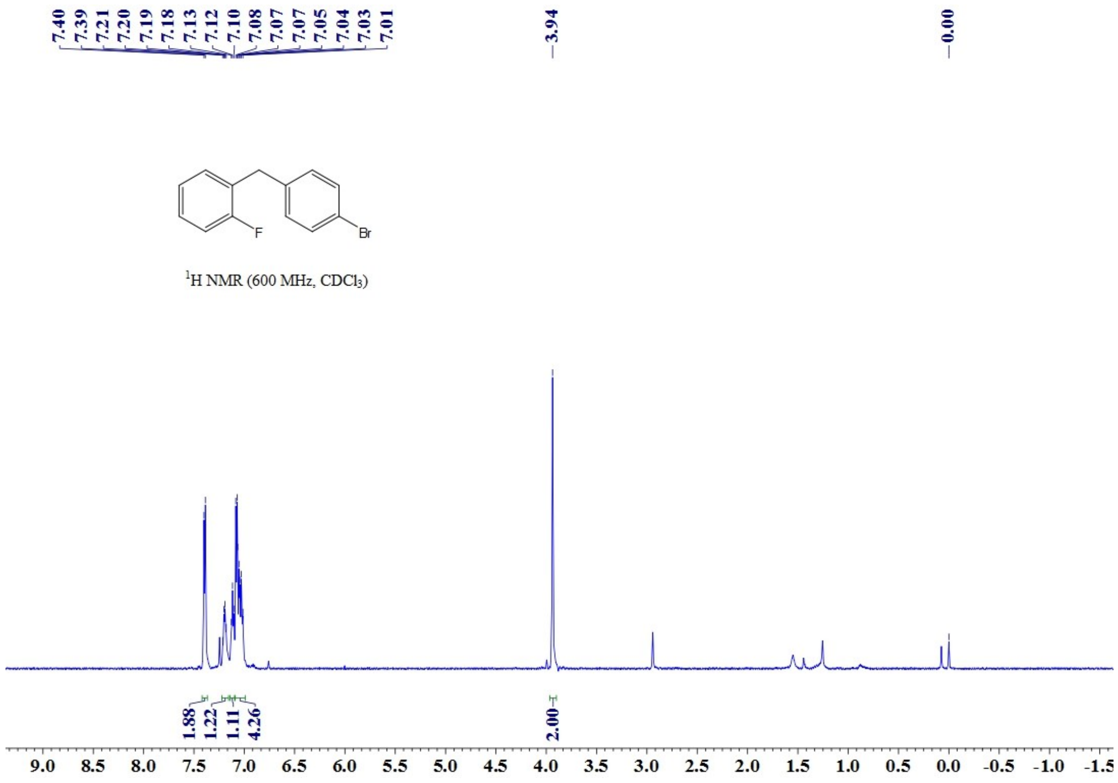

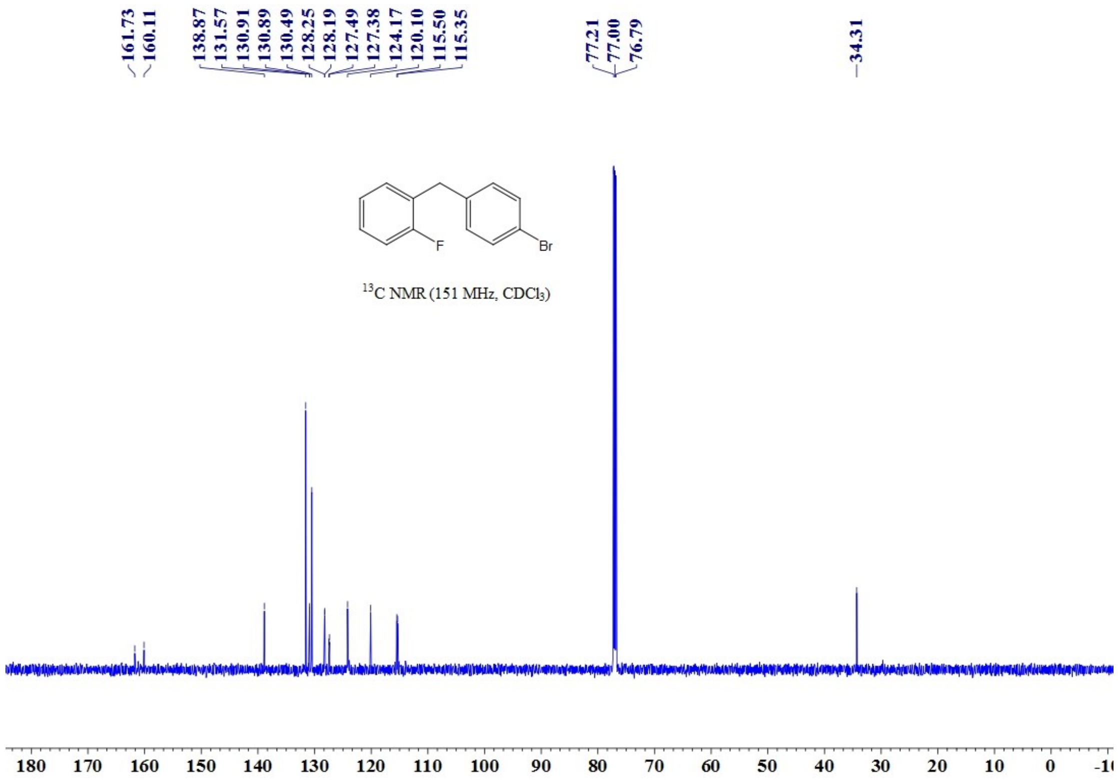

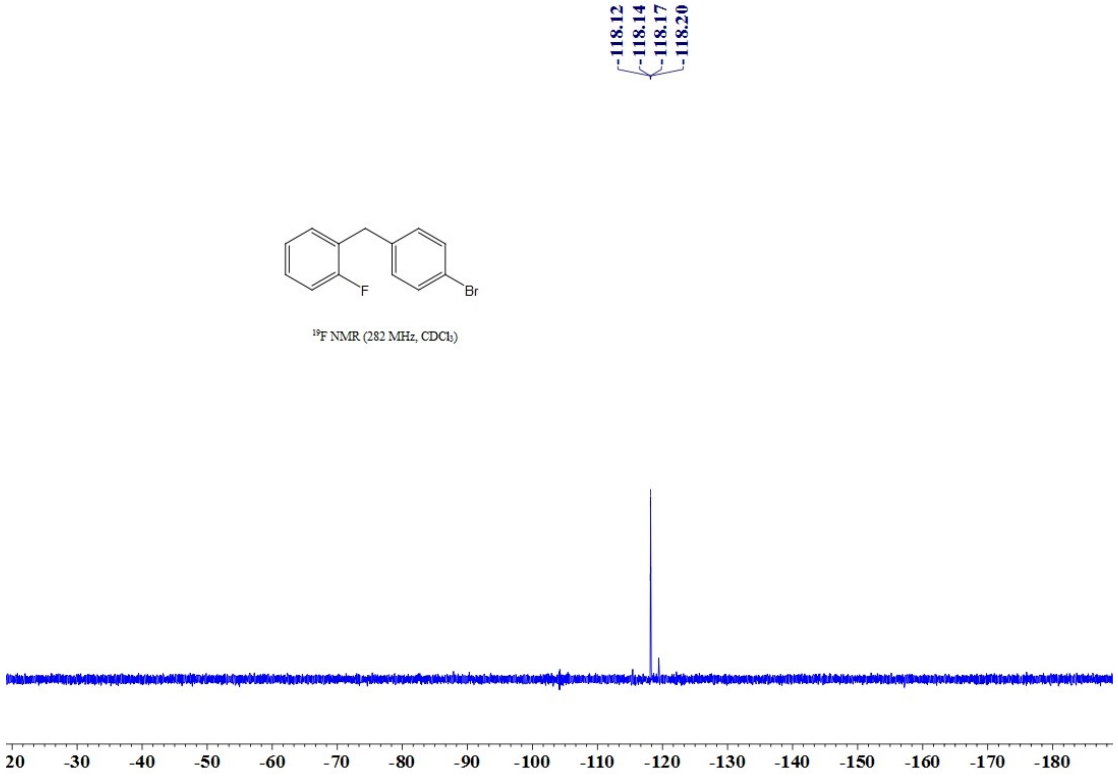

^{1}H, ^{19}F and ^{13}C NMR spectra of compound **3d**

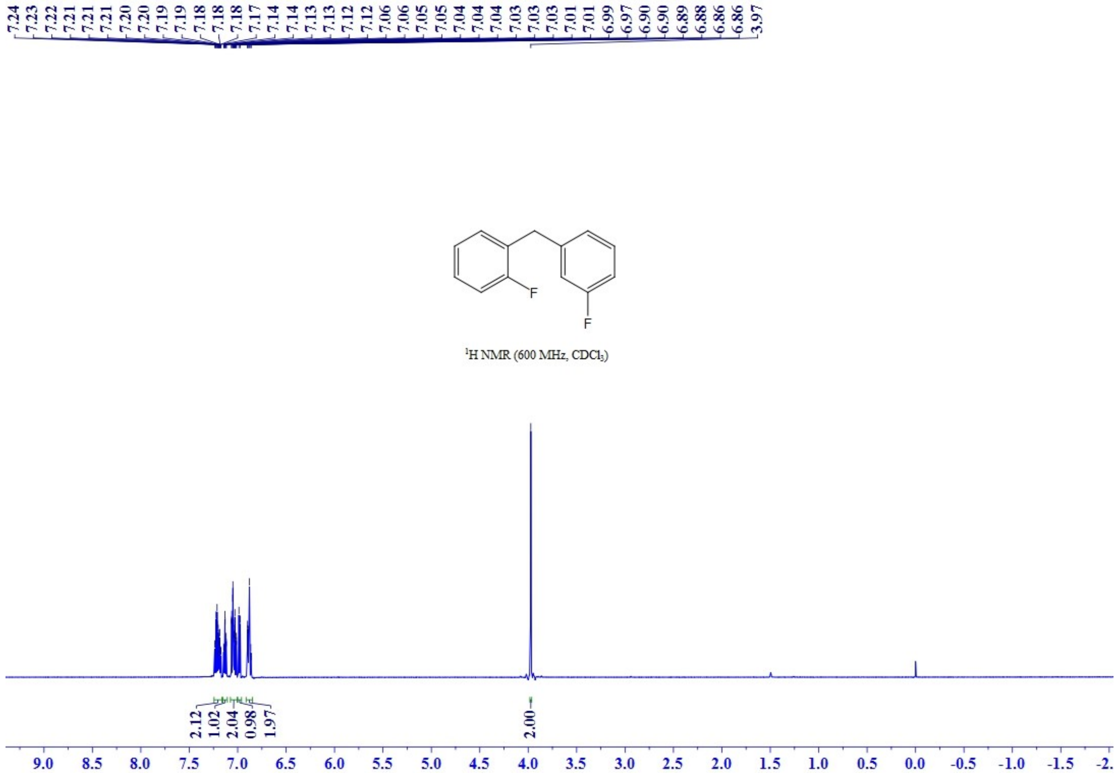

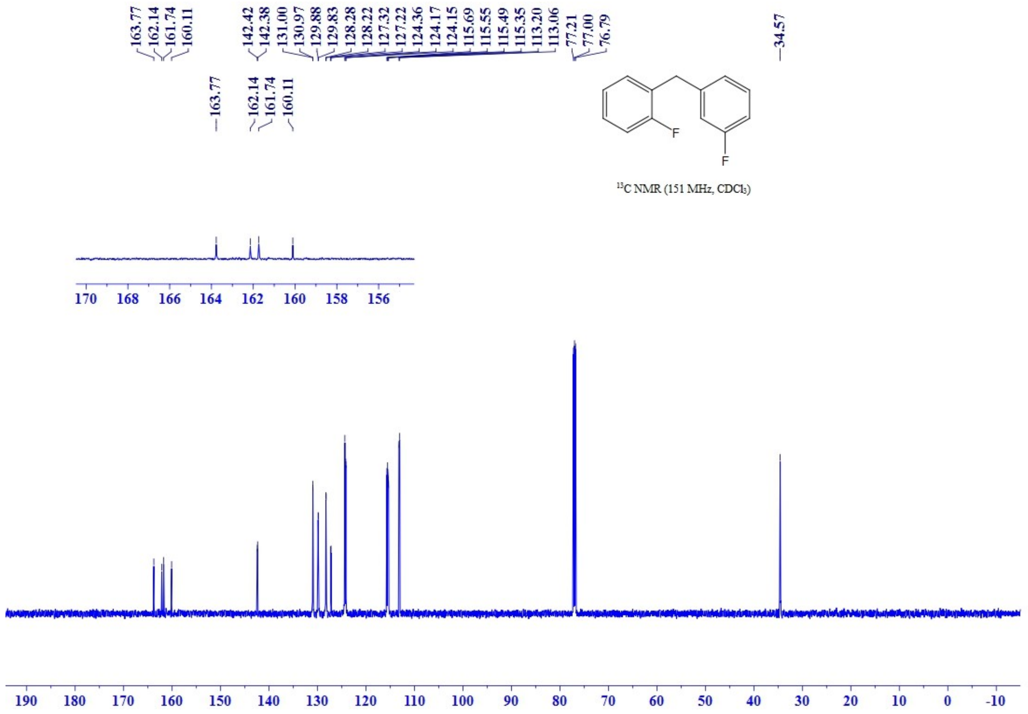

^{13}C NMR (151 MHz, $CDCl_3$)

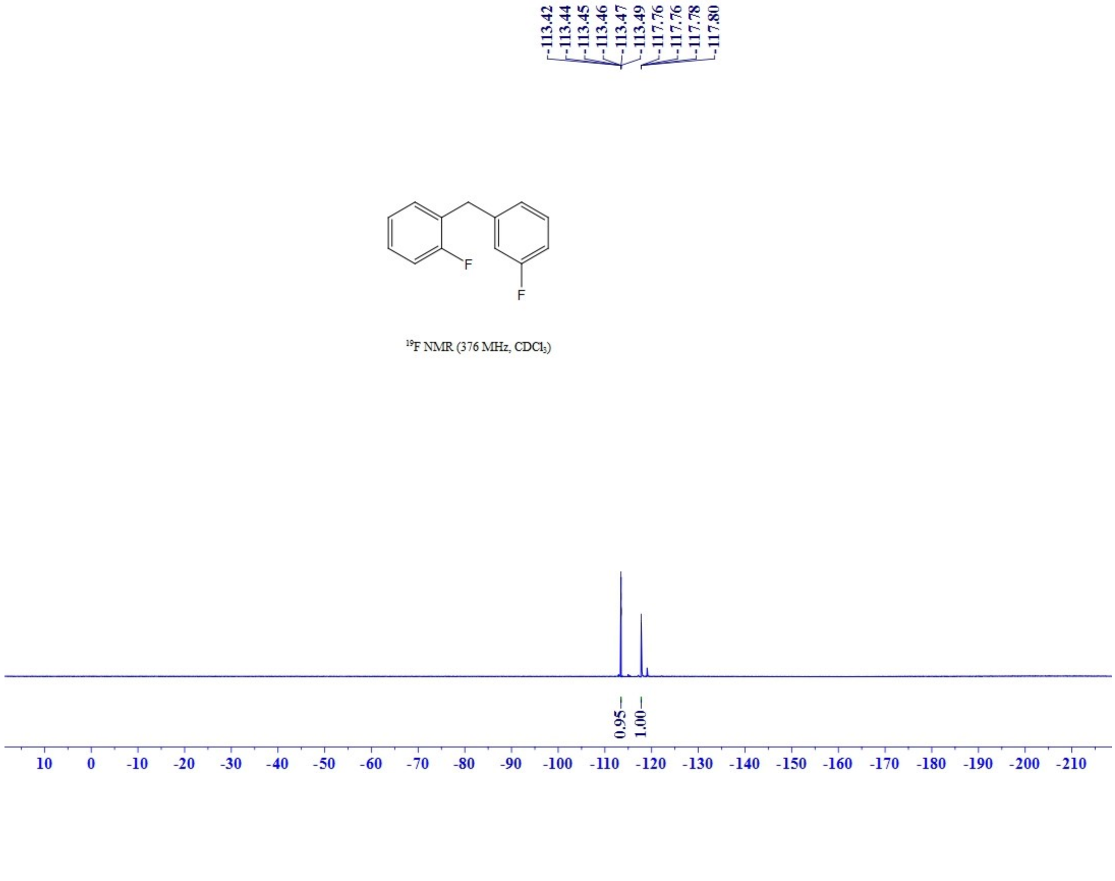

^{19}F NMR (376 MHz, $CDCl_3$)

^{1}H, ^{19}F and ^{13}C NMR spectra of compound **3e**

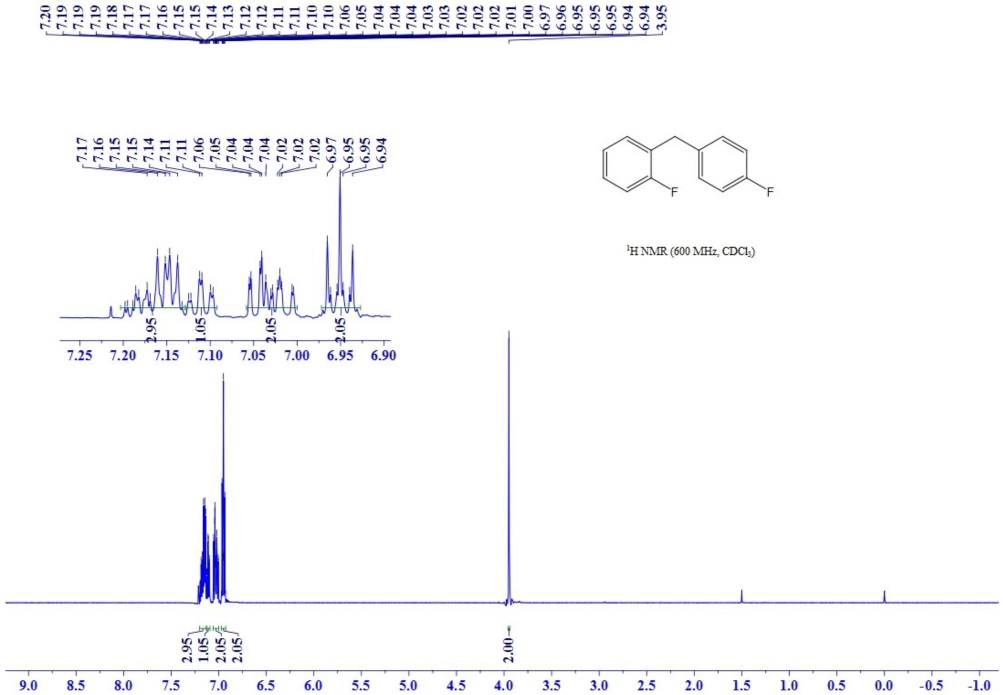

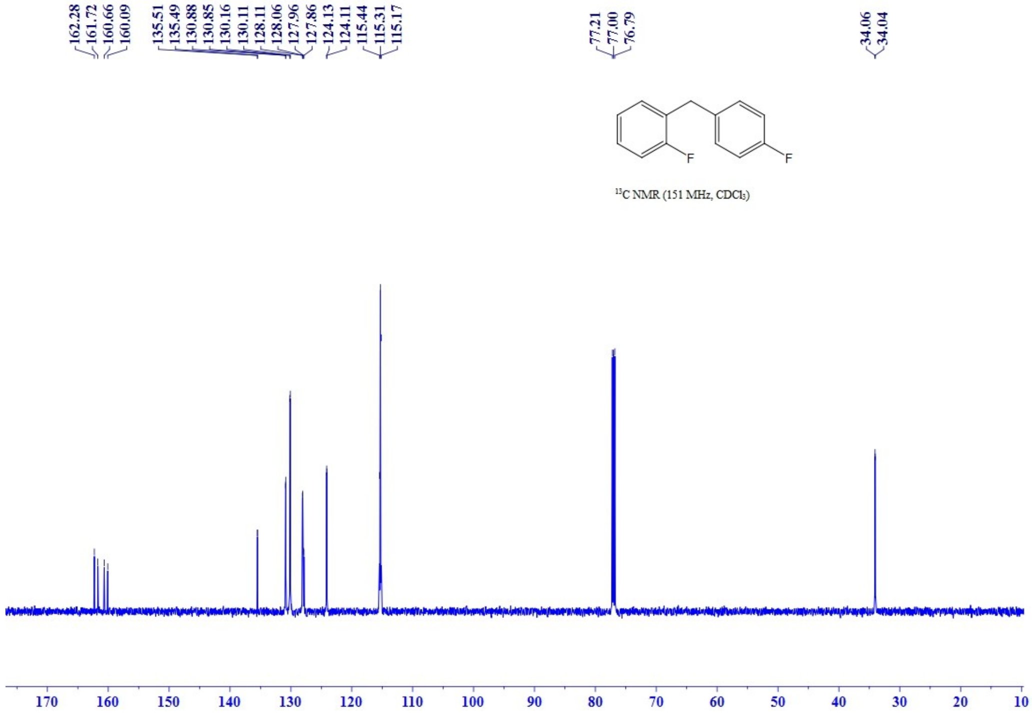

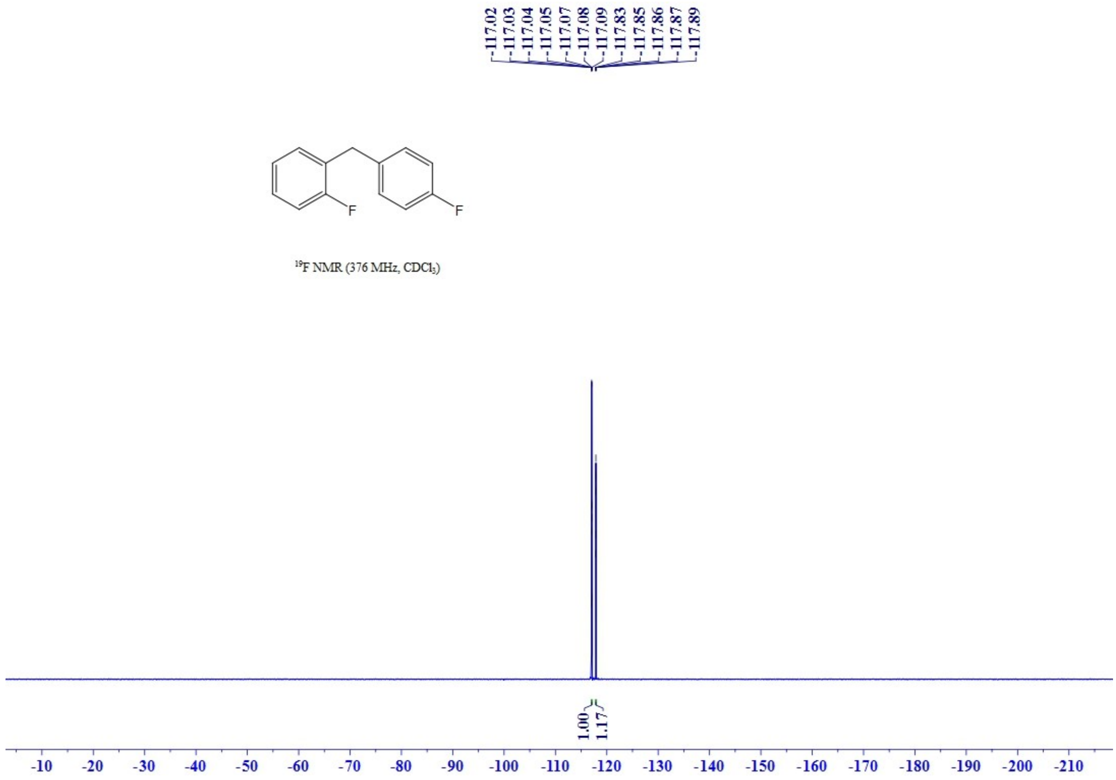

^{1}H, ^{19}F and ^{13}C NMR spectra of compound **3f**

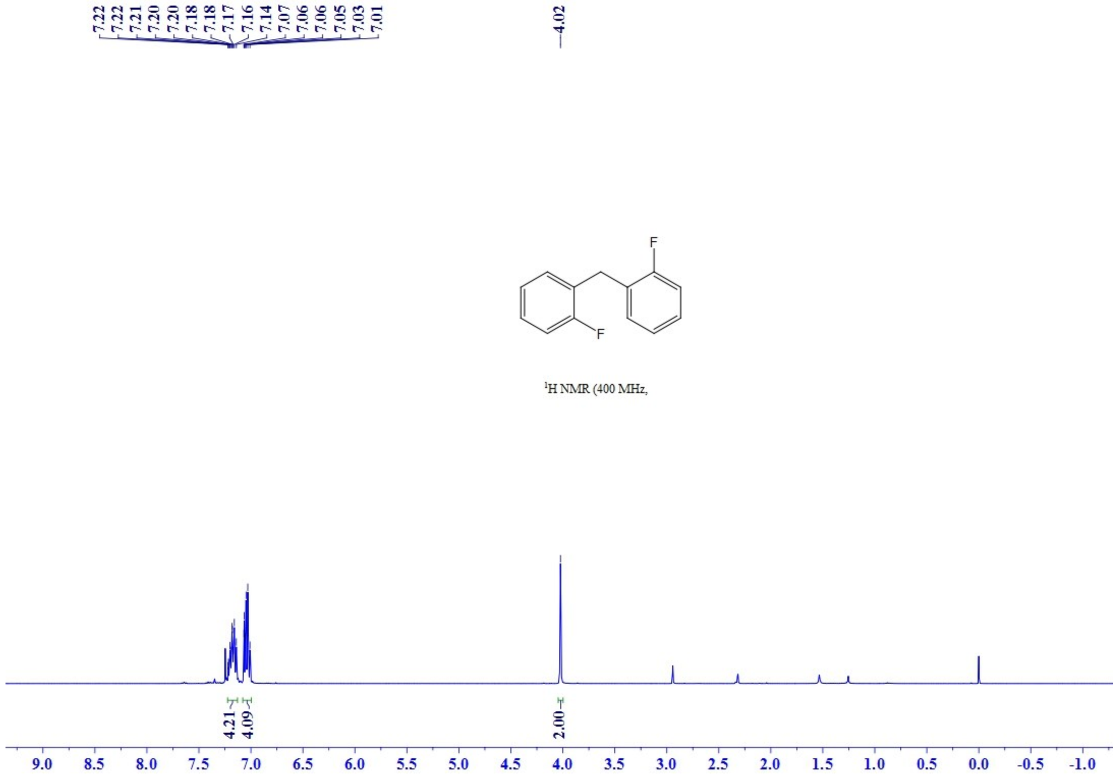

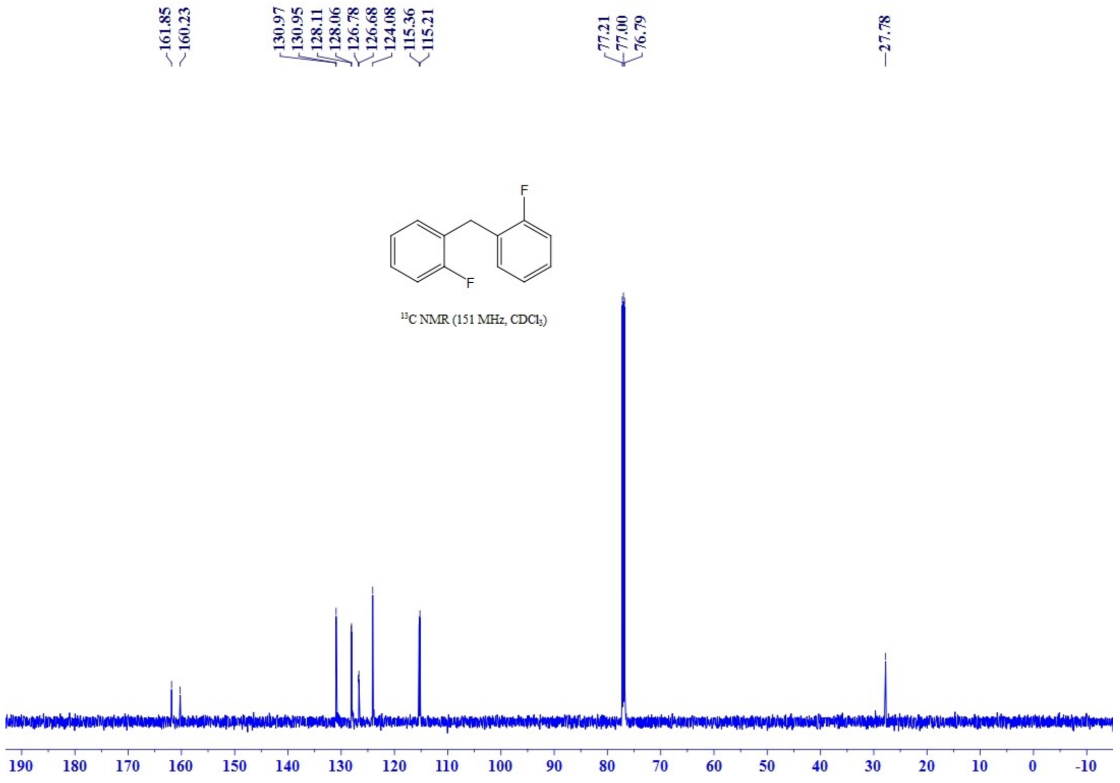
161.85
160.23
130.97
130.95
128.11
128.06
126.78
126.68
124.08
115.36
115.21
77.21
77.00
76.79
27.78
F
F
13C NMR (151 MHz, CDCl3)
190 180 170 160 150 140 130 120 110 100 90 80 70 60 50 40 30 20 10 0 -10

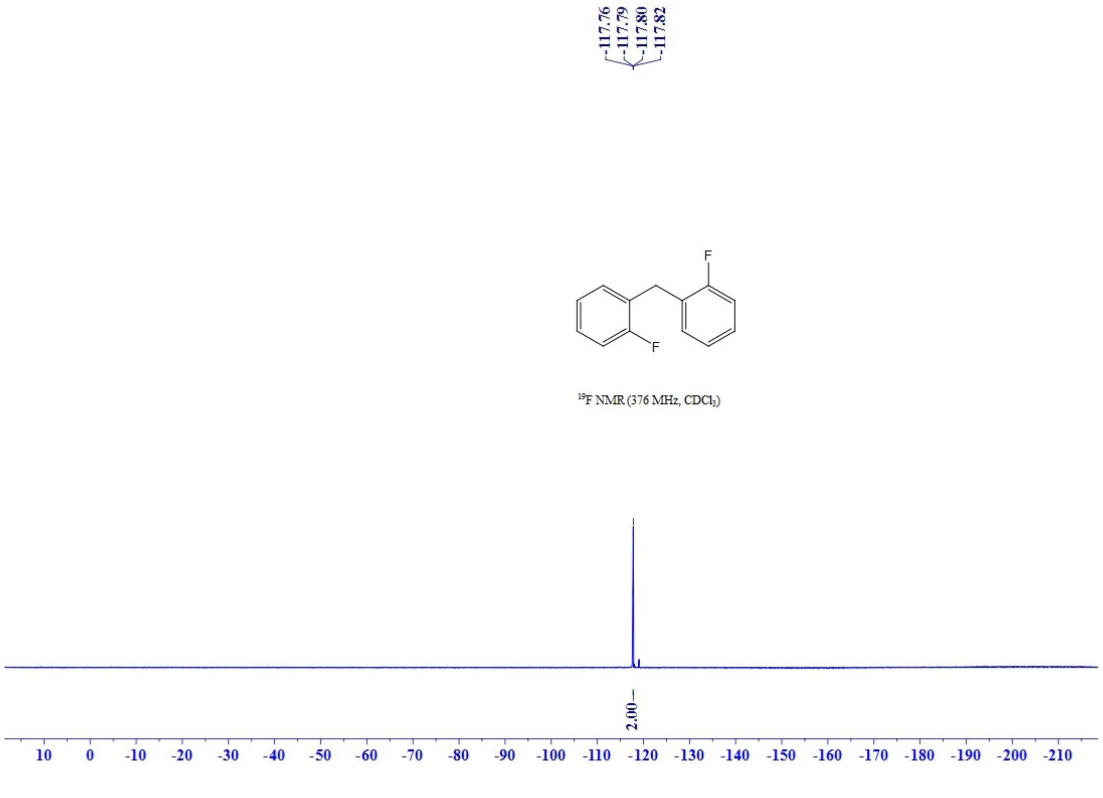
-117.76
-117.79
-117.80
-117.82
F
F
19F NMR (376 MHz, CDCl3)
2.00
10 0 -10 -20 -30 -40 -50 -60 -70 -80 -90 -100 -110 -120 -130 -140 -150 -160 -170 -180 -190 -200 -210

^{1}H, ^{19}F and ^{13}C NMR spectra of compound **3g**

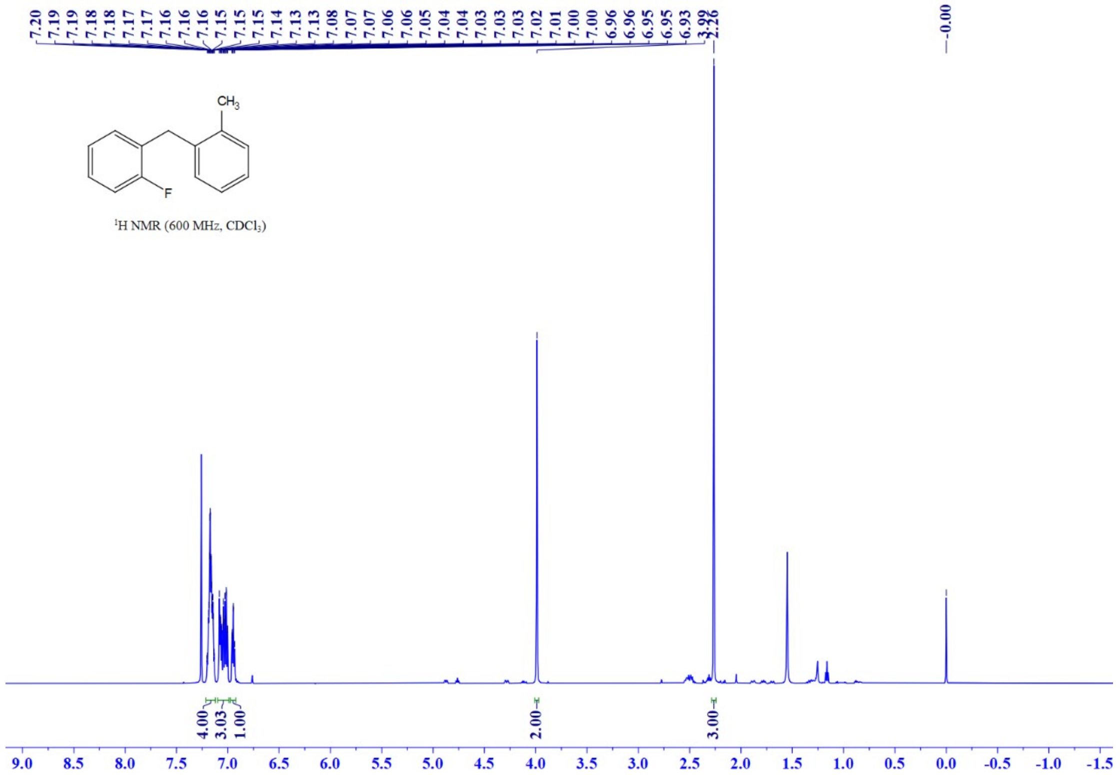

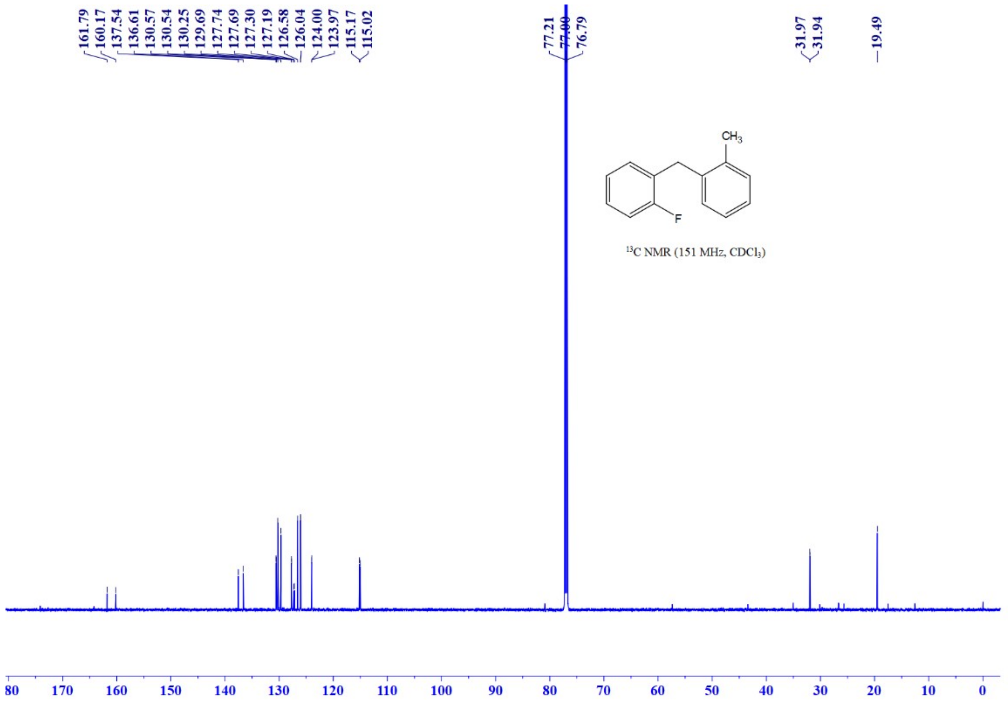

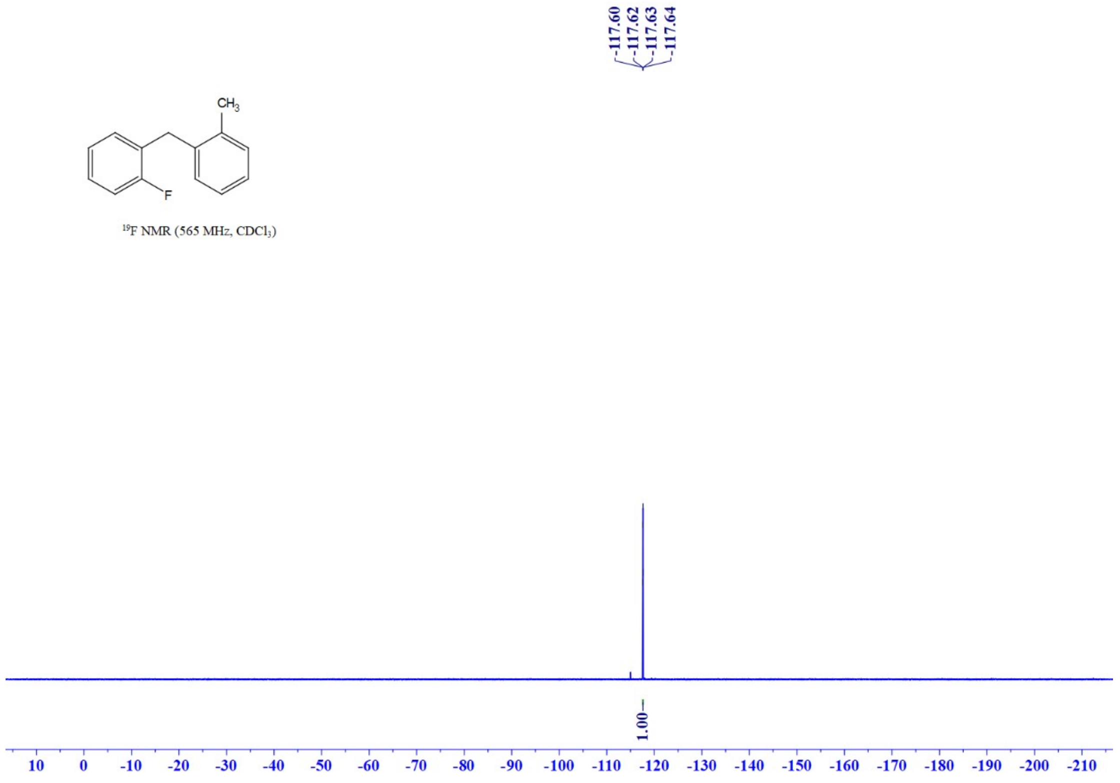

^{1}H, ^{19}F and ^{13}C NMR spectra of compound **3h**

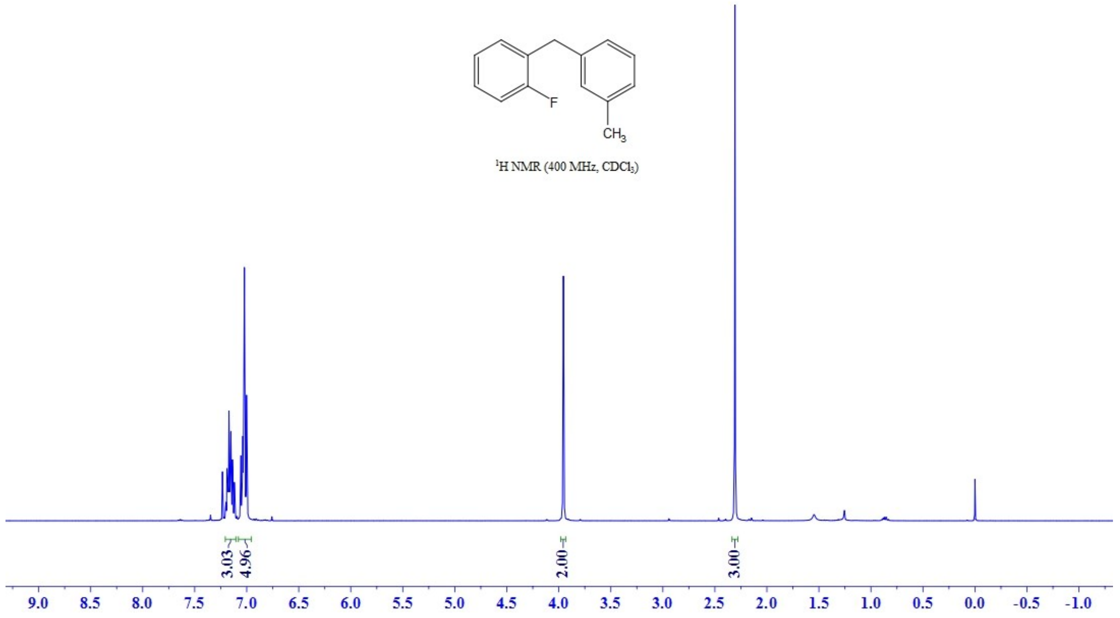

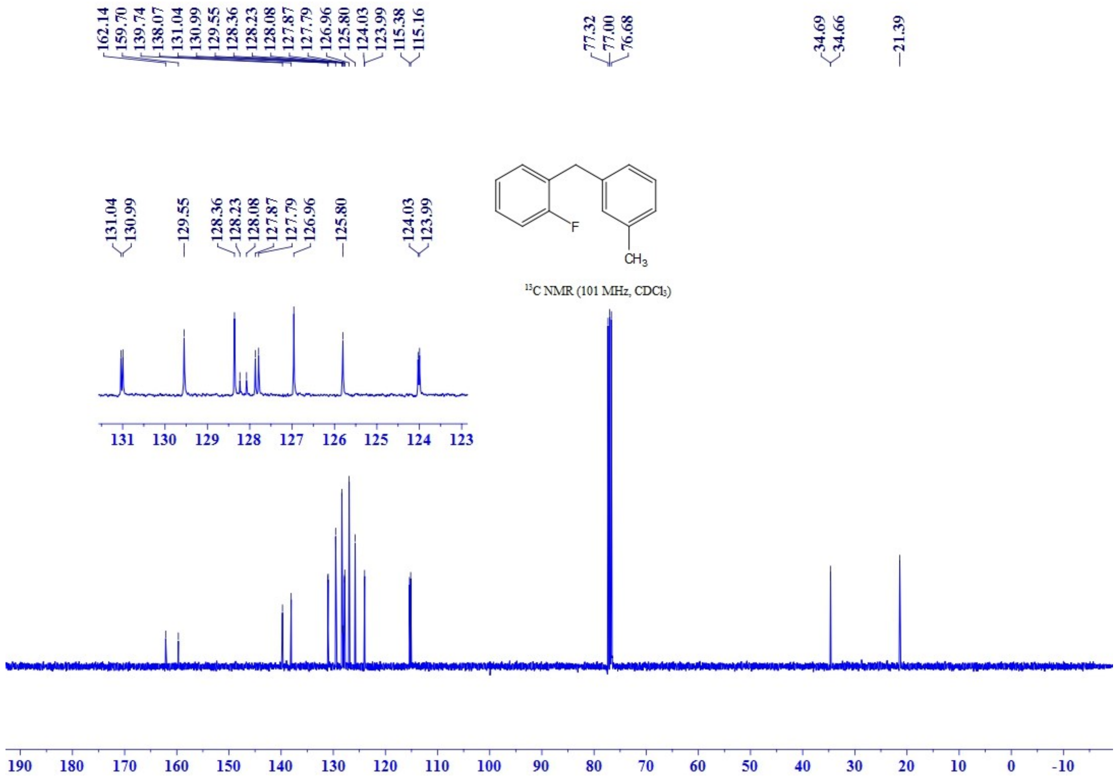

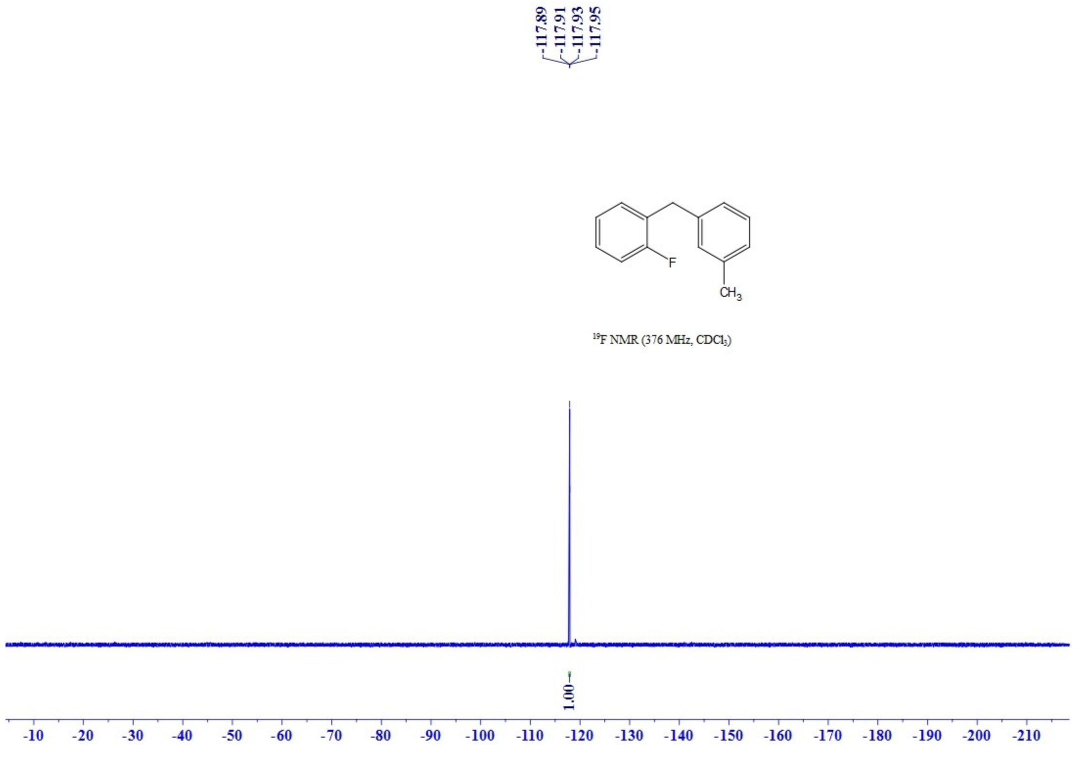

^{1}H, ^{19}F and ^{13}C NMR spectra of compound **3i**

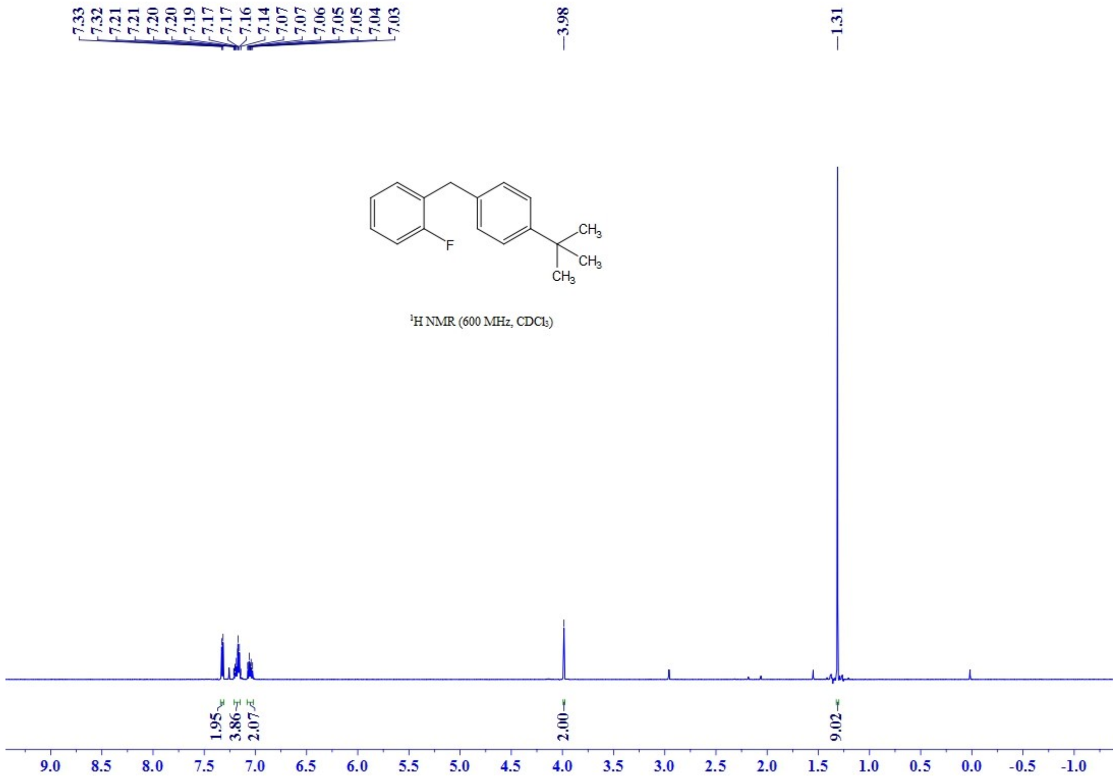
1H NMR (600 MHz, CDCl3)
CH3
F

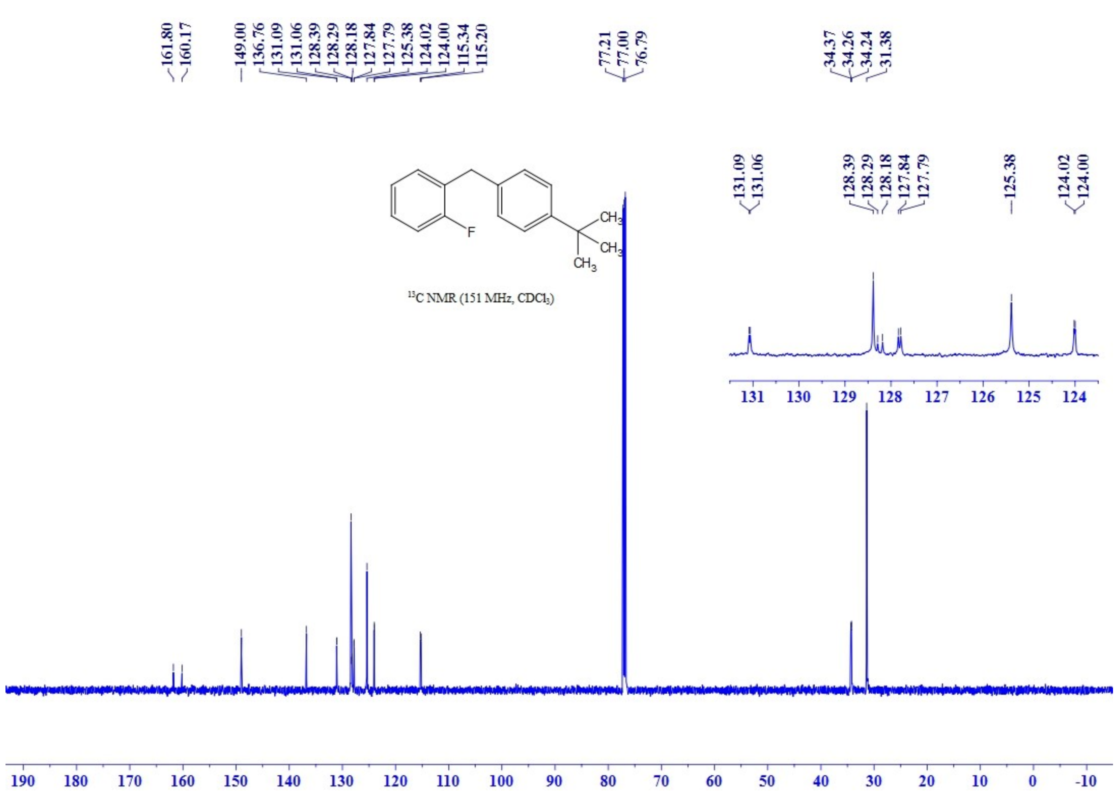
13C NMR (151 MHz, CDCl3)
CH3
F

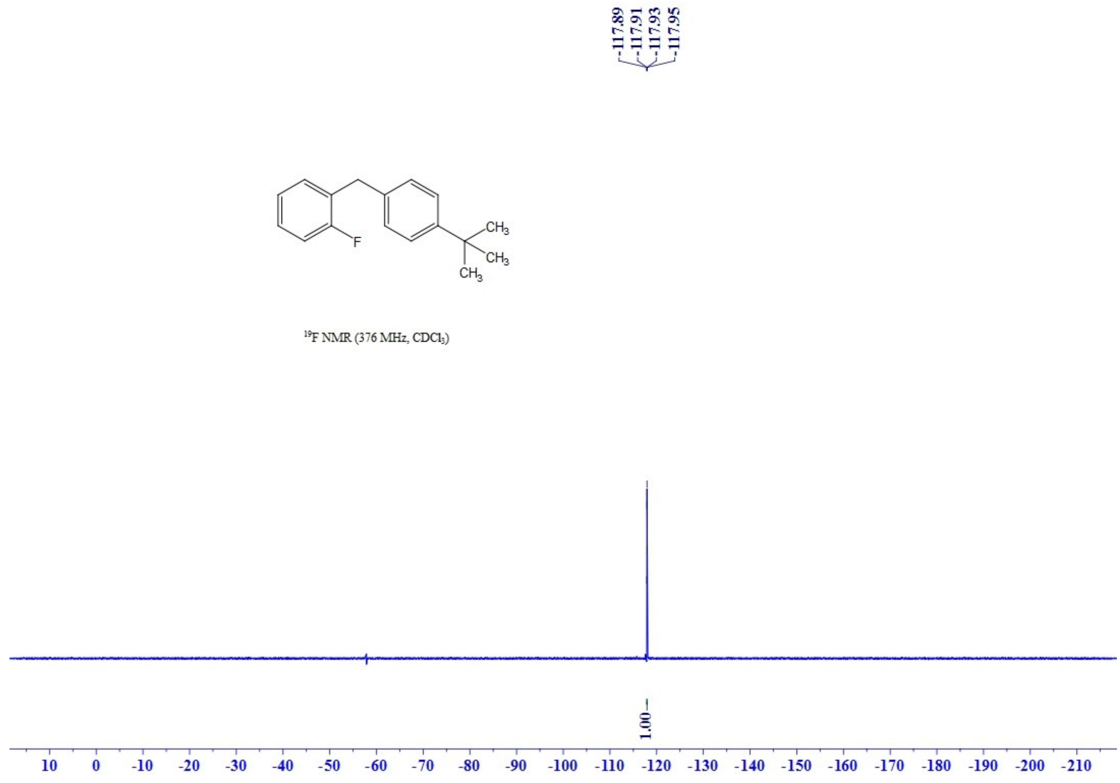

^{1}H, ^{19}F and ^{13}C NMR spectra of compound **3j**

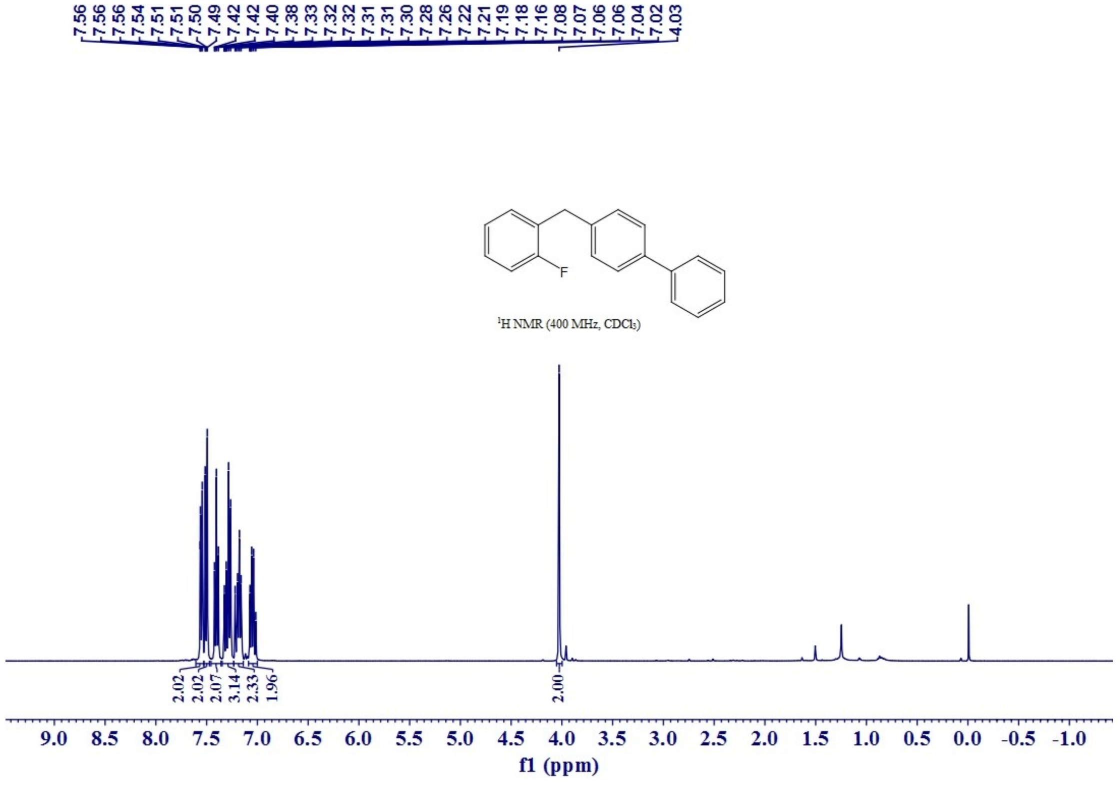

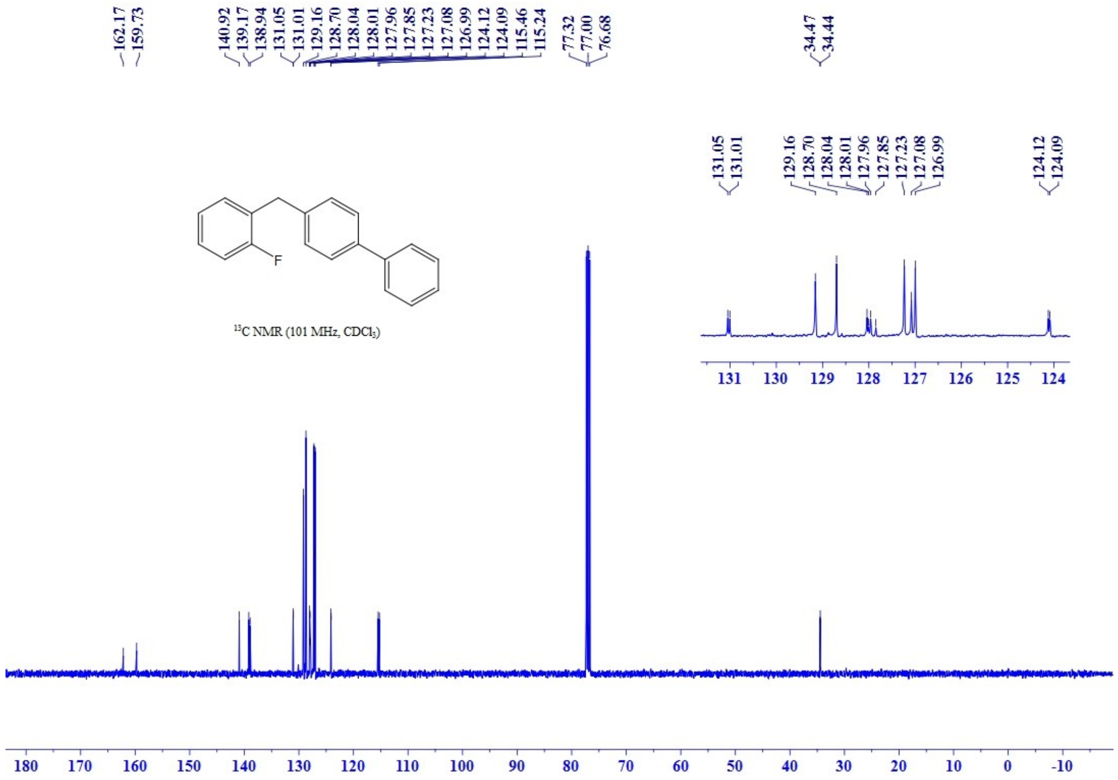

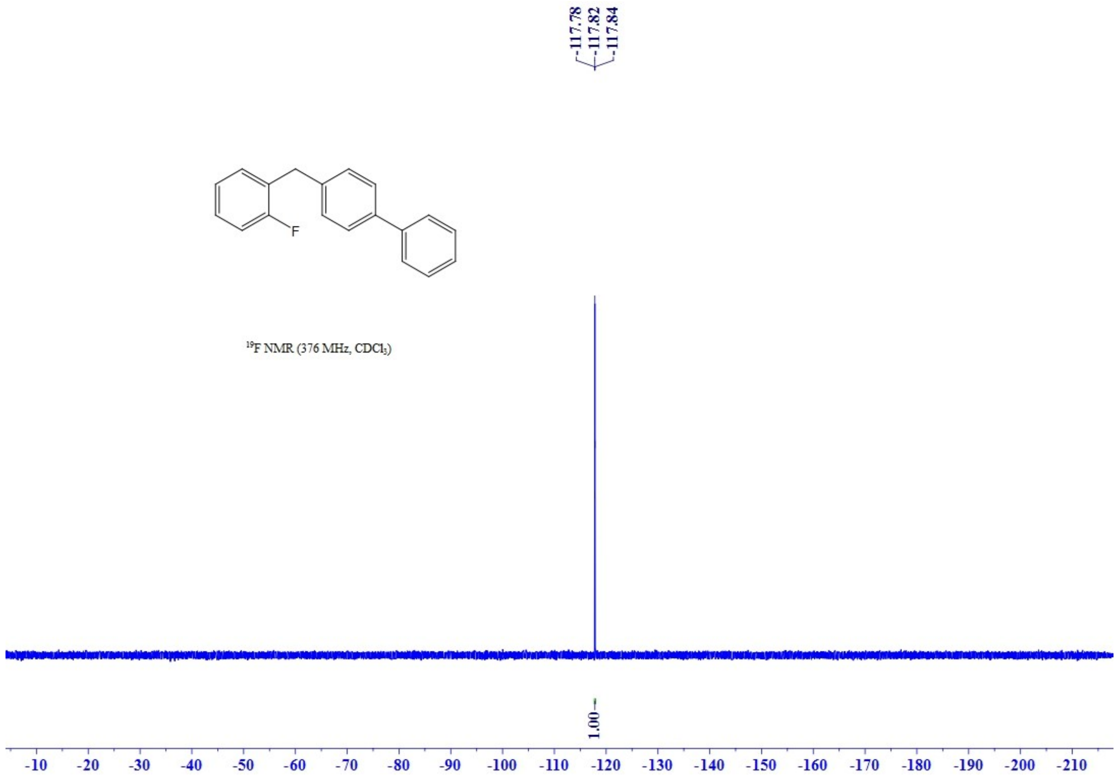

^{1}H, ^{19}F and ^{13}C NMR spectra of compound **3k**

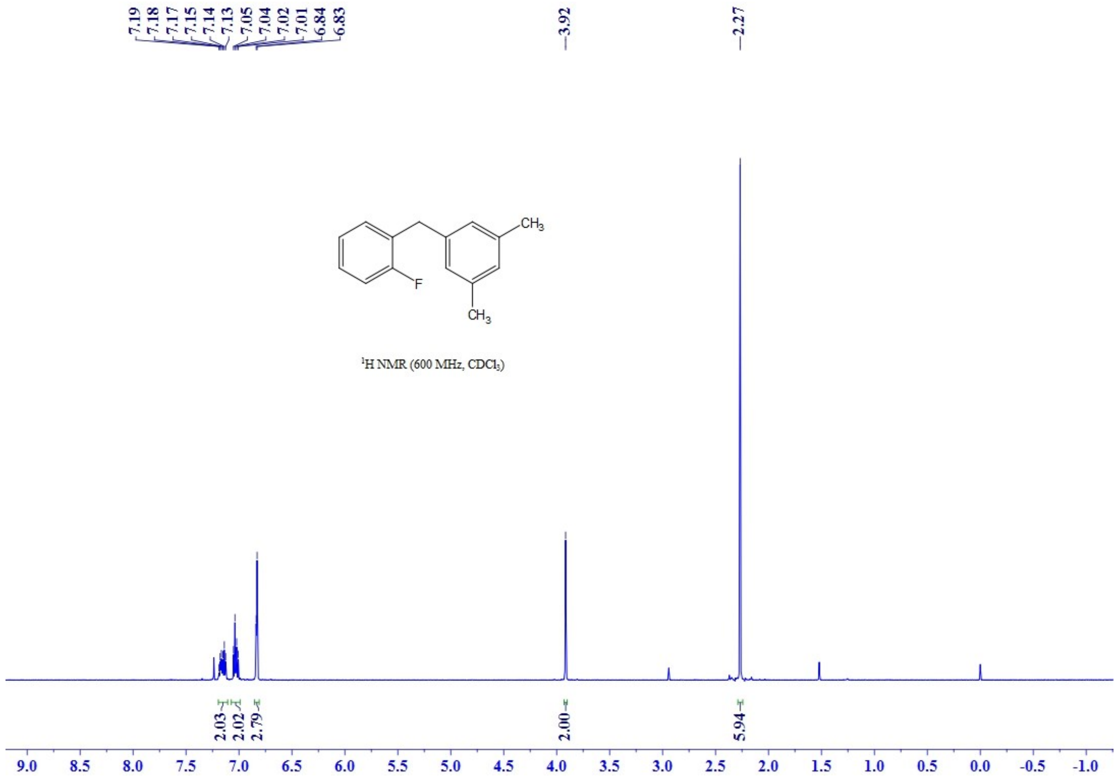

7.19
7.18
7.17
7.15
7.14
7.13
7.05
7.04
7.02
7.01
6.84
6.83
3.92
2.27
CH3
F
CH3
1H NMR (600 MHz, CDCl3)
2.03
2.02
2.79
2.00
5.94

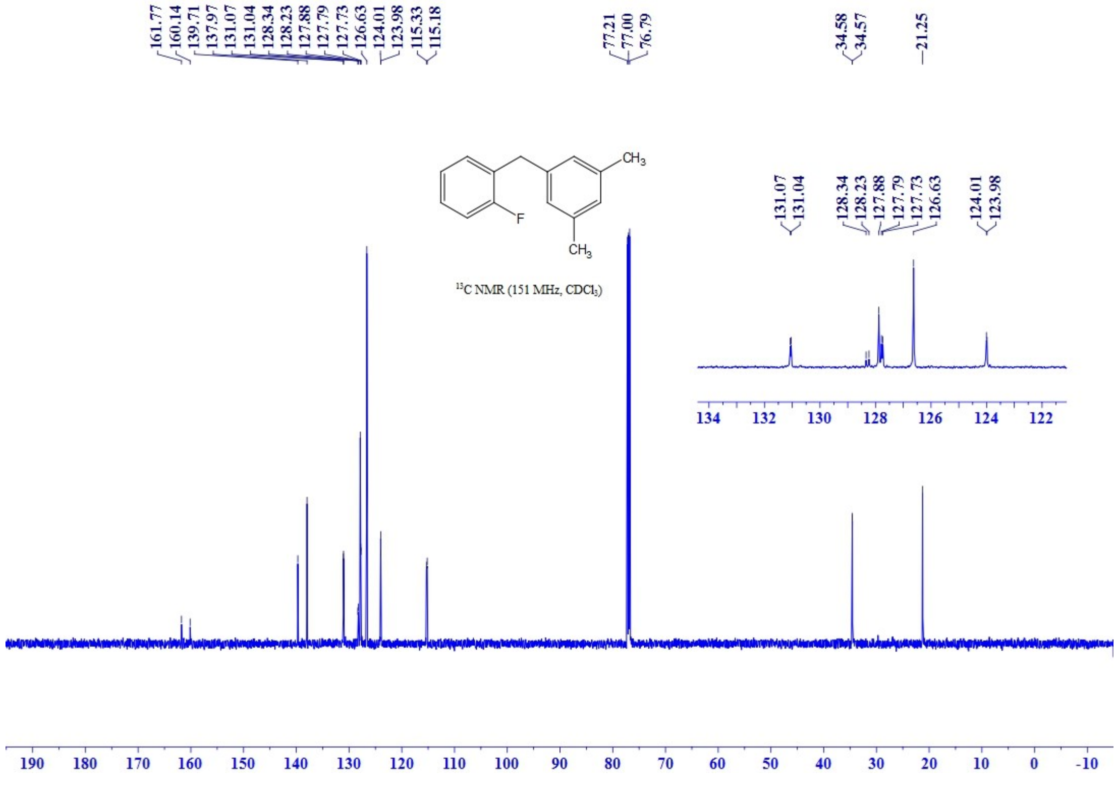

161.77
160.14
139.71
137.97
131.07
131.04
128.34
128.23
127.88
127.79
127.73
126.63
124.01
123.98
115.33
115.18
77.21
77.00
76.79
34.58
34.57
21.25
CH3
F
CH3
13C NMR (151 MHz, CDCl3)

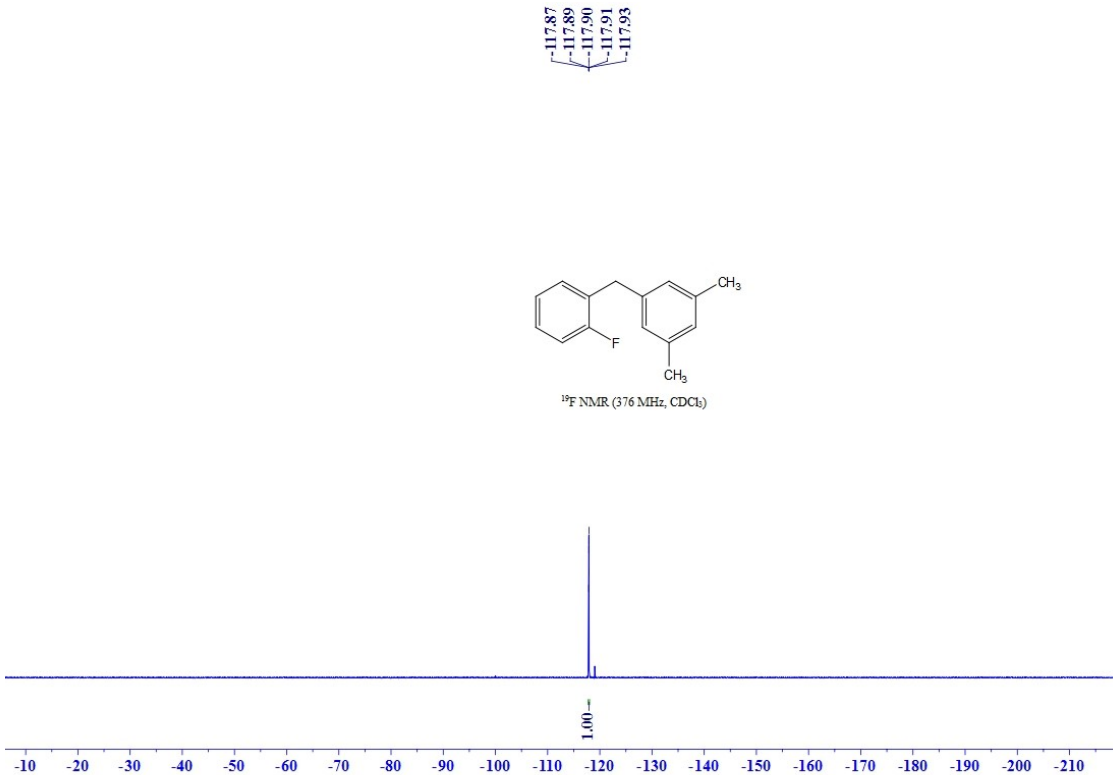

^{1}H, ^{19}F and ^{13}C NMR spectra of compound **3l**

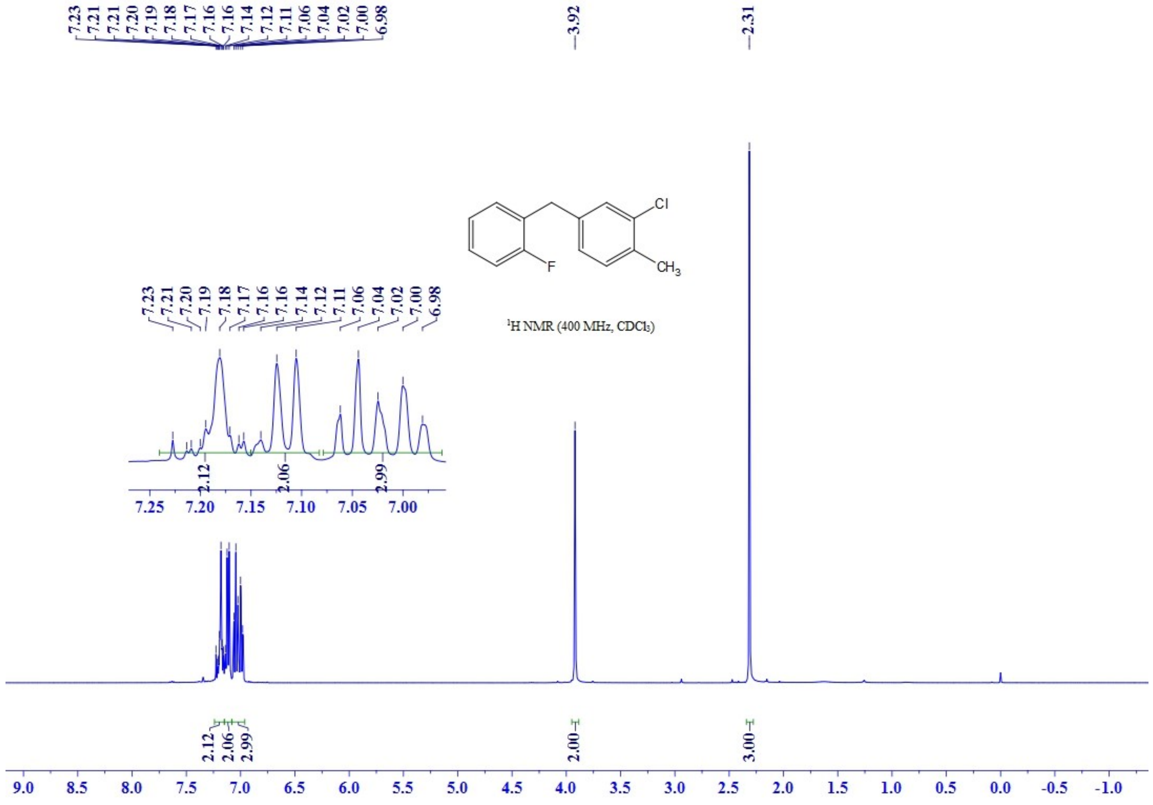

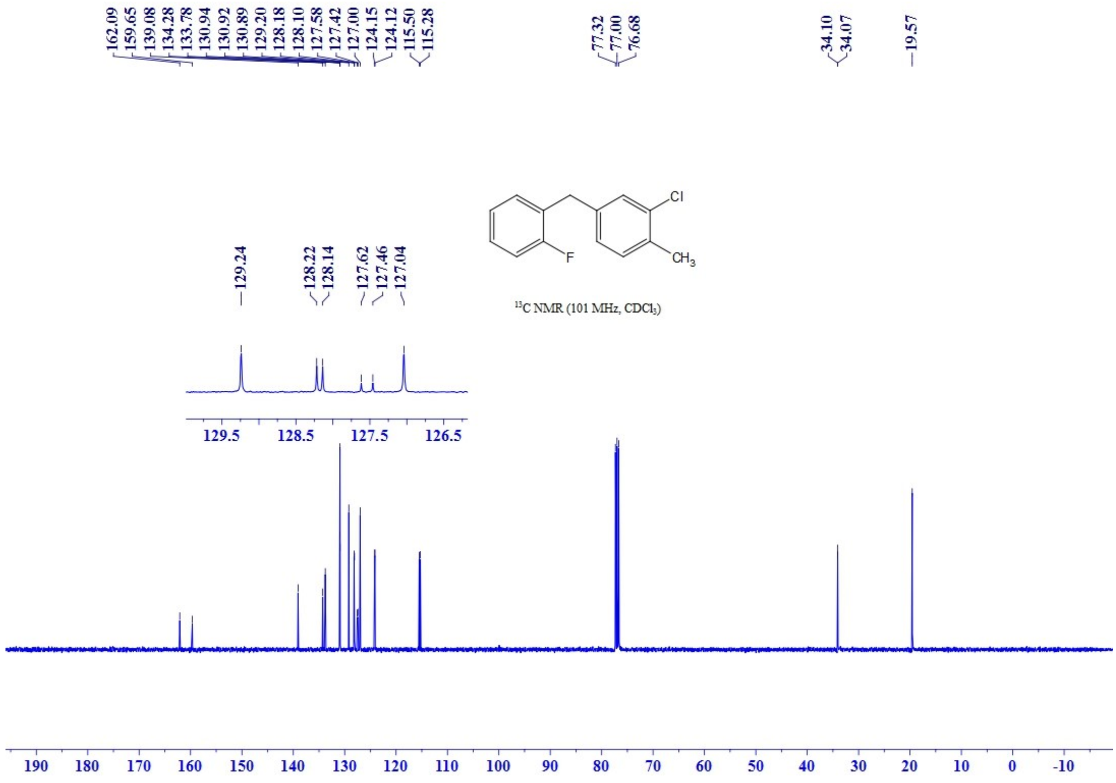

^{13}C NMR (101 MHz, $CDCl_3$)

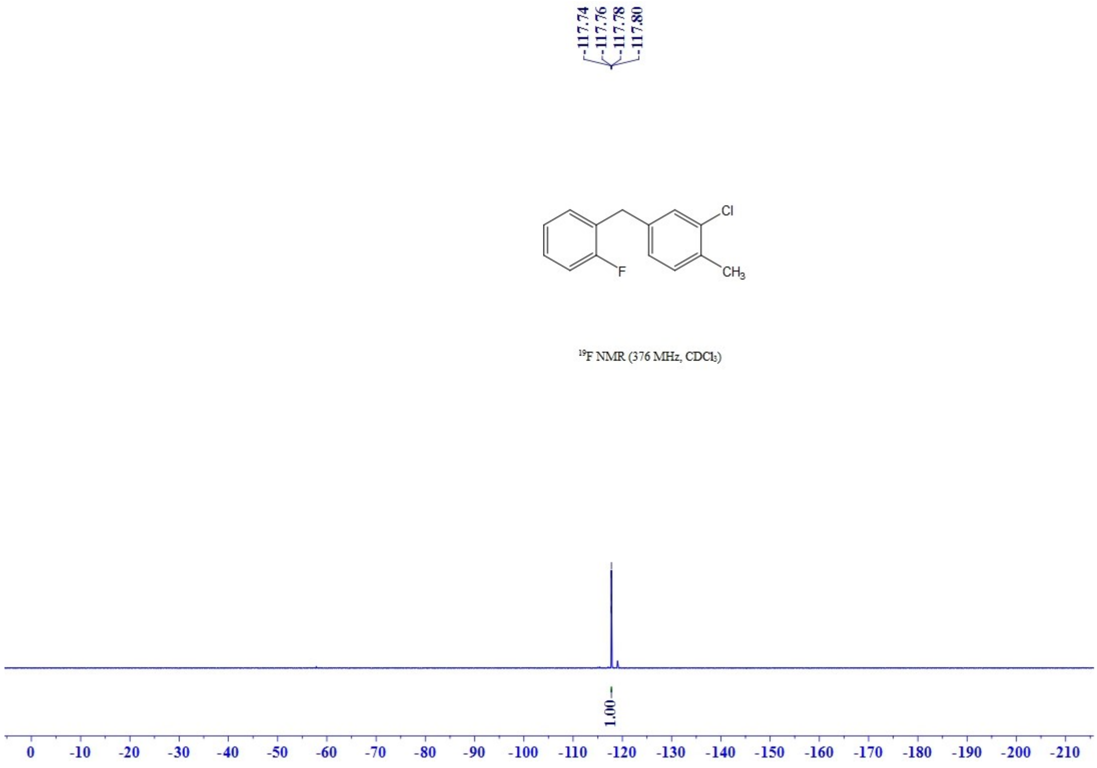

^{19}F NMR (376 MHz, $CDCl_3$)

^{1}H, ^{19}F and ^{13}C NMR spectra of compound **3m**

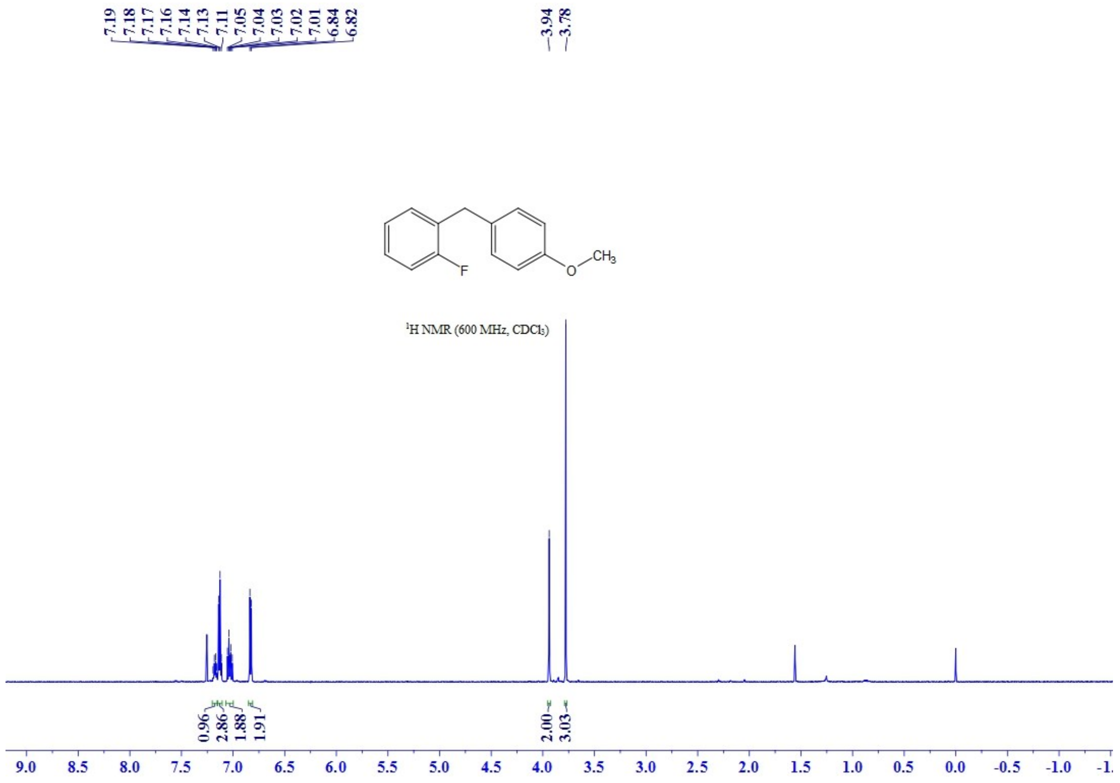

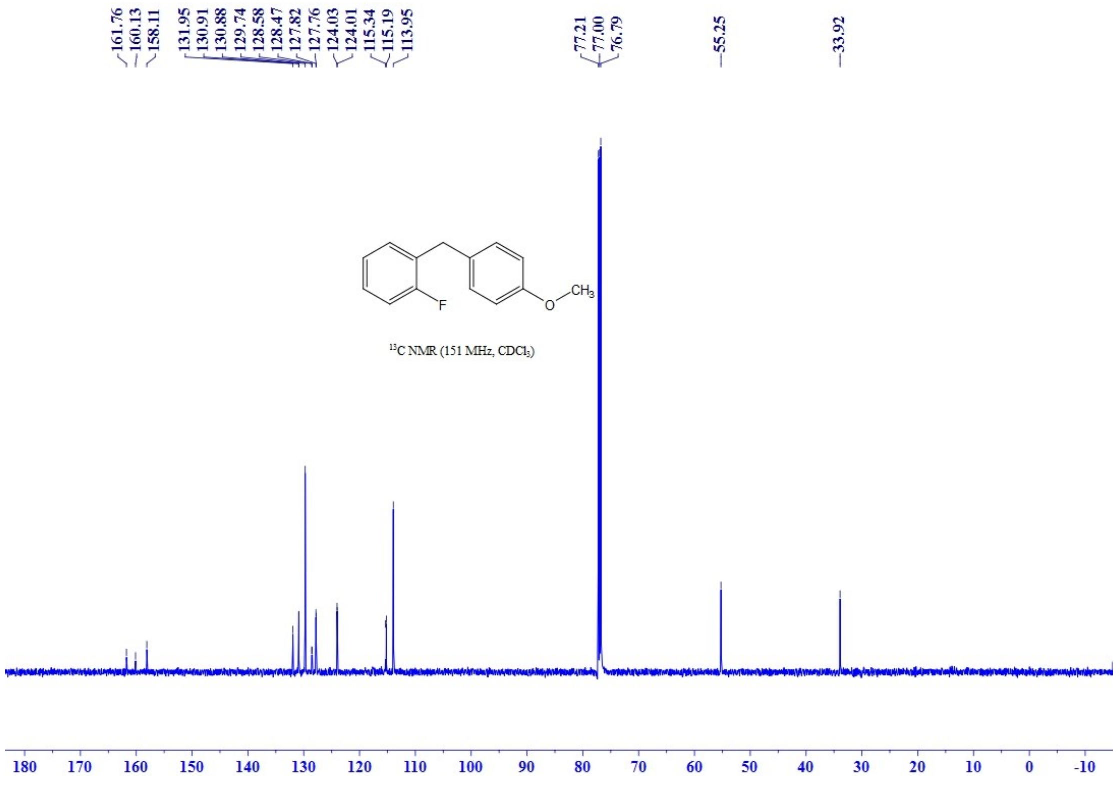

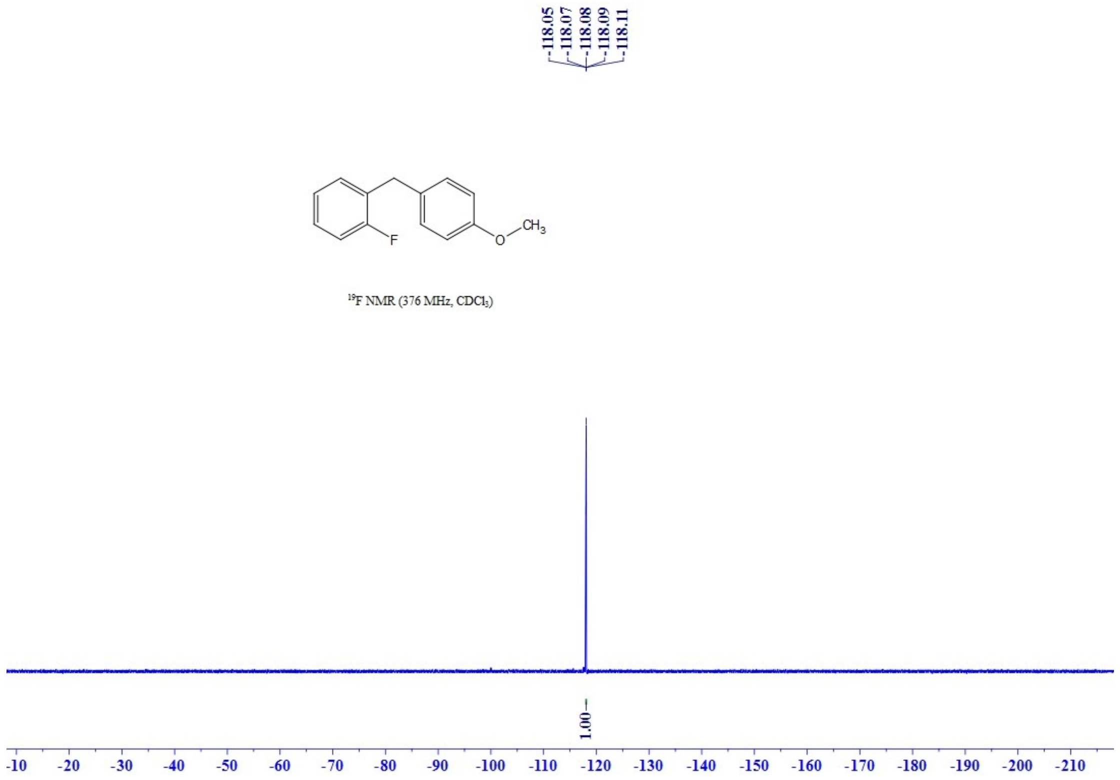

^{1}H, ^{19}F and ^{13}C NMR spectra of compound **3n**

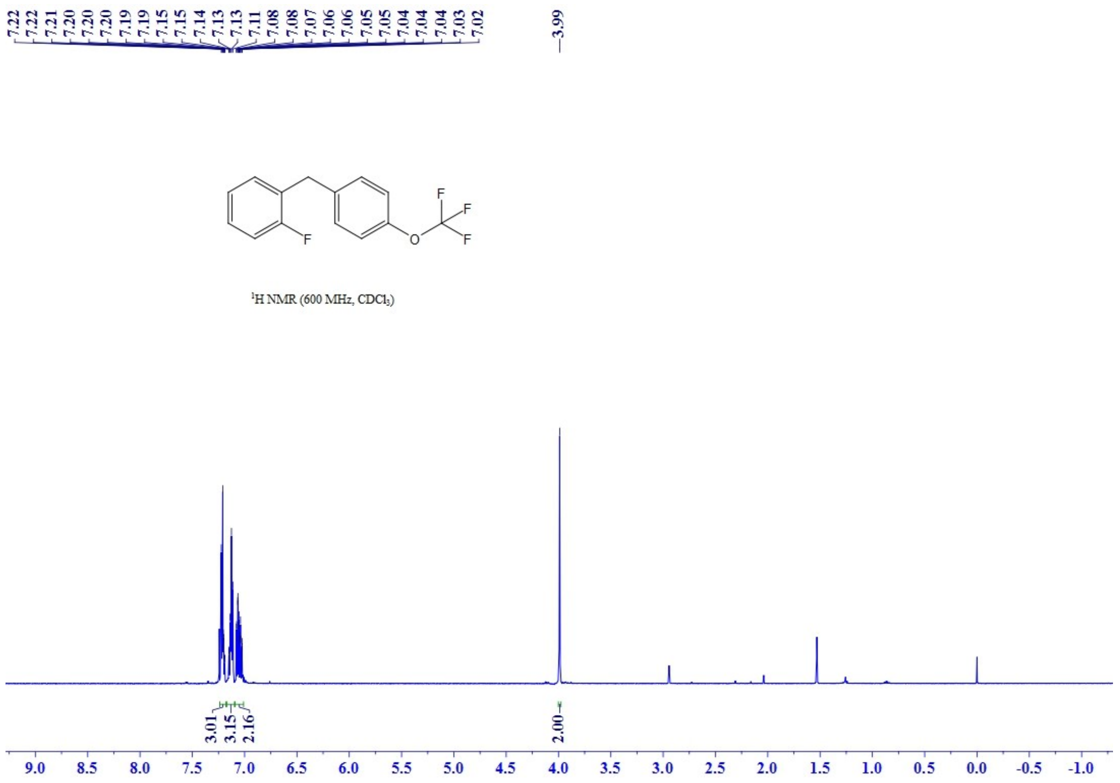

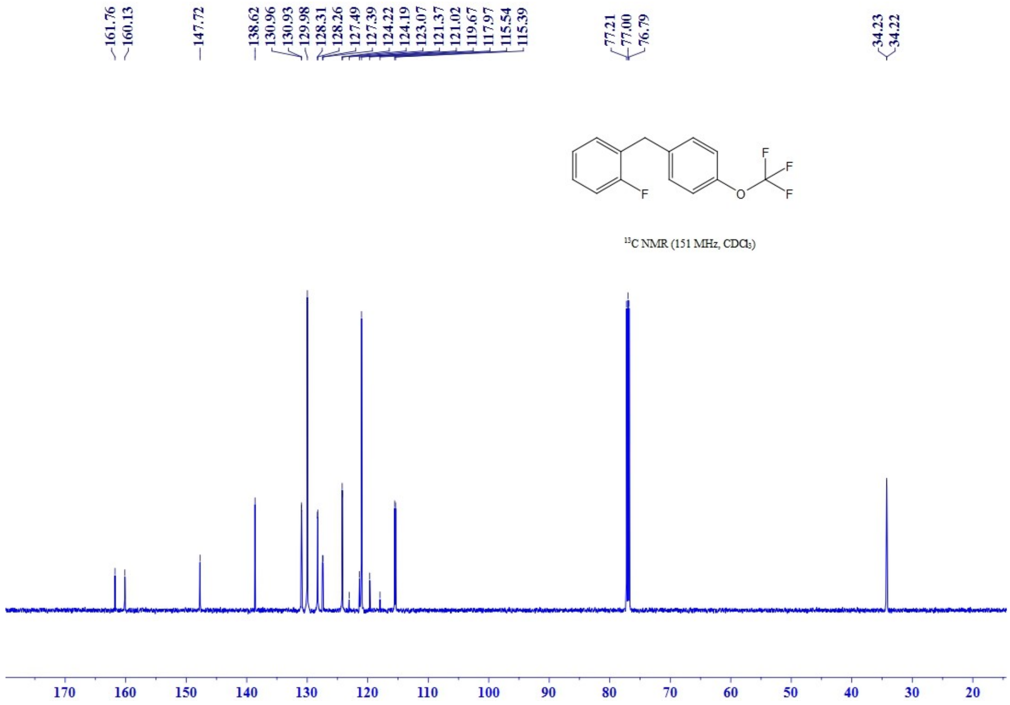

161.76
160.13
147.72
138.62
130.96
130.93
129.98
128.31
128.26
127.49
127.39
124.22
124.19
123.07
121.37
121.02
119.67
117.97
115.54
115.39
77.21
77.00
76.79
34.23
34.22
F
F
F
O
F
^{13}C NMR (151 MHz, $CDCl_3$)
170 160 150 140 130 120 110 100 90 80 70 60 50 40 30 20

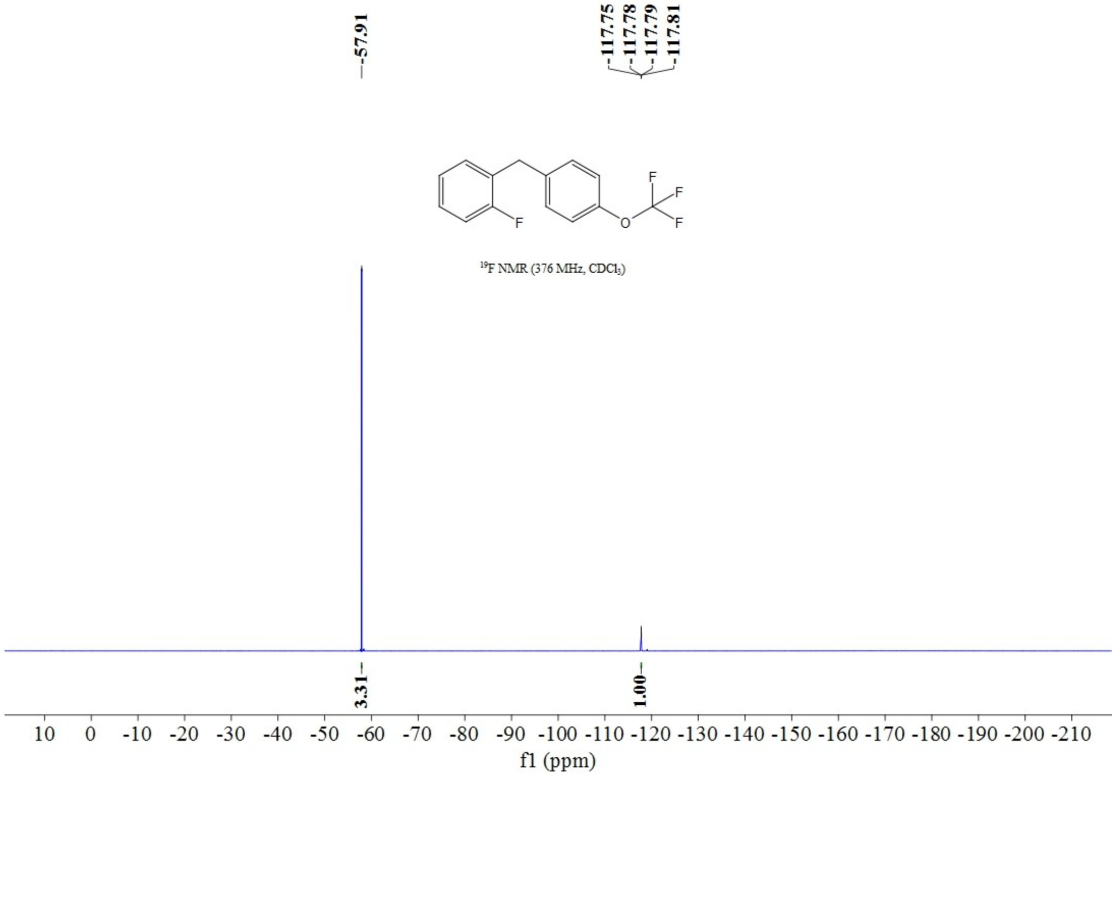

-57.91
-117.75
-117.78
-117.79
-117.81
F
F
F
O
F
^{19}F NMR (376 MHz, $CDCl_3$)
3.31
1.00
10 0 -10 -20 -30 -40 -50 -60 -70 -80 -90 -100 -110 -120 -130 -140 -150 -160 -170 -180 -190 -200 -210
f1 (ppm)

^{1}H, ^{19}F and ^{13}C NMR spectra of compound **3o**

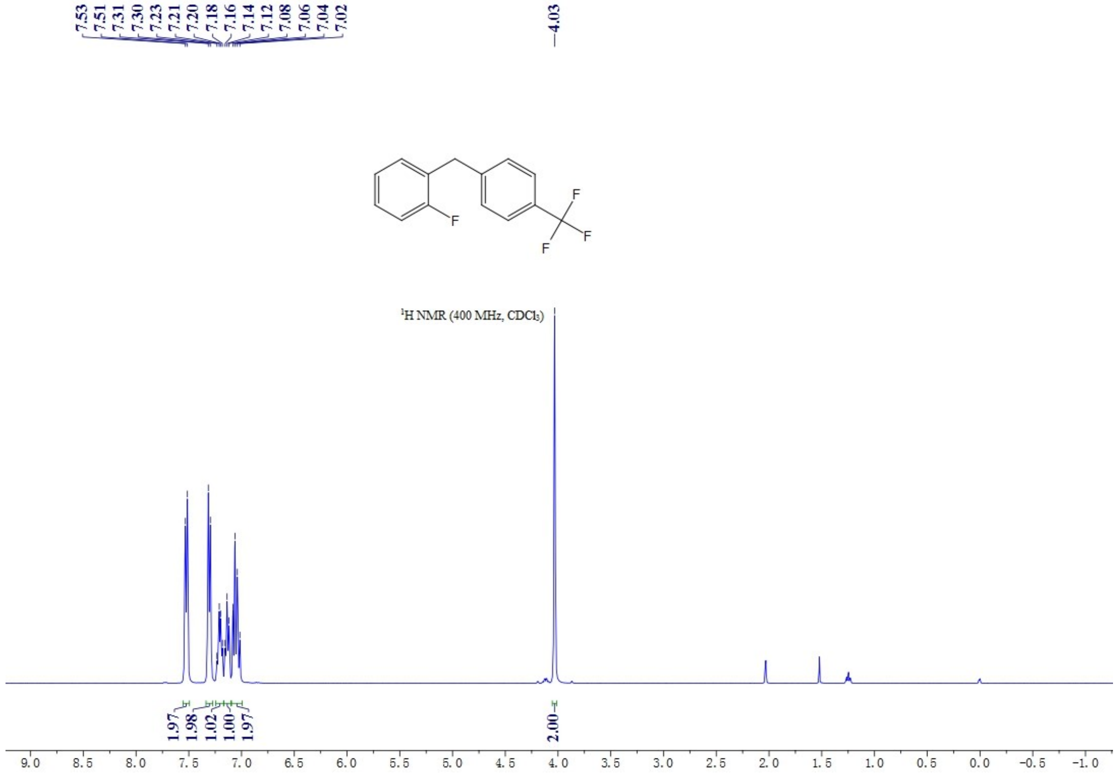

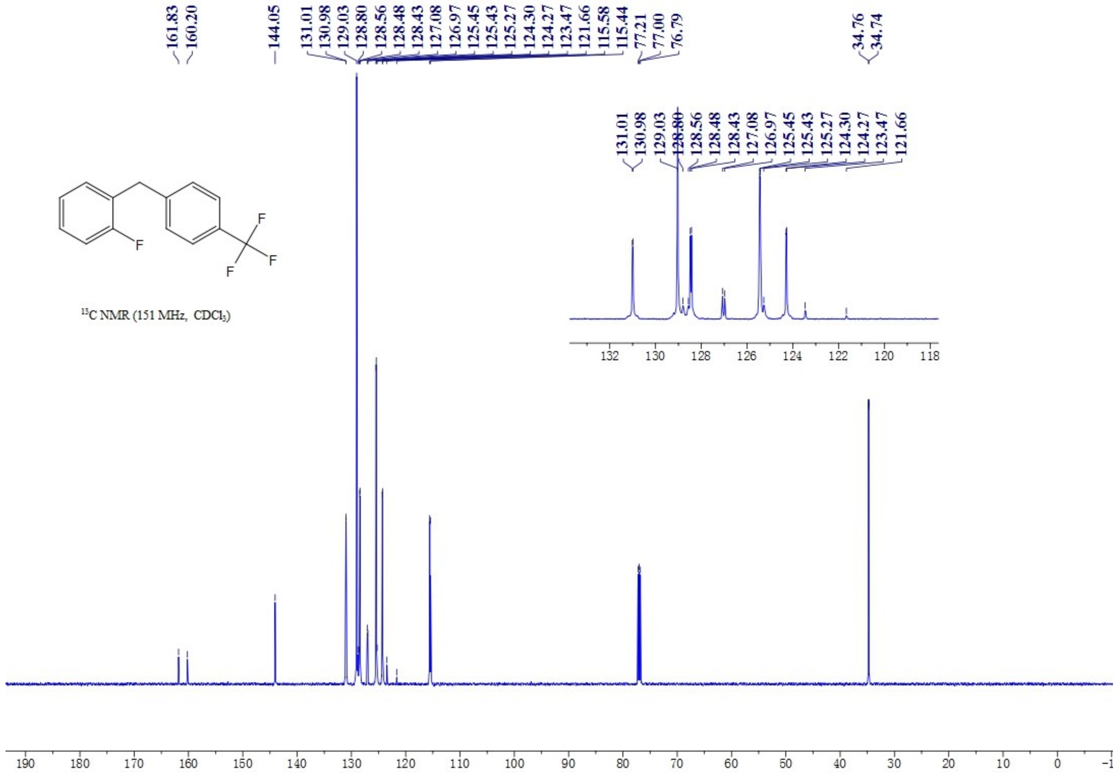

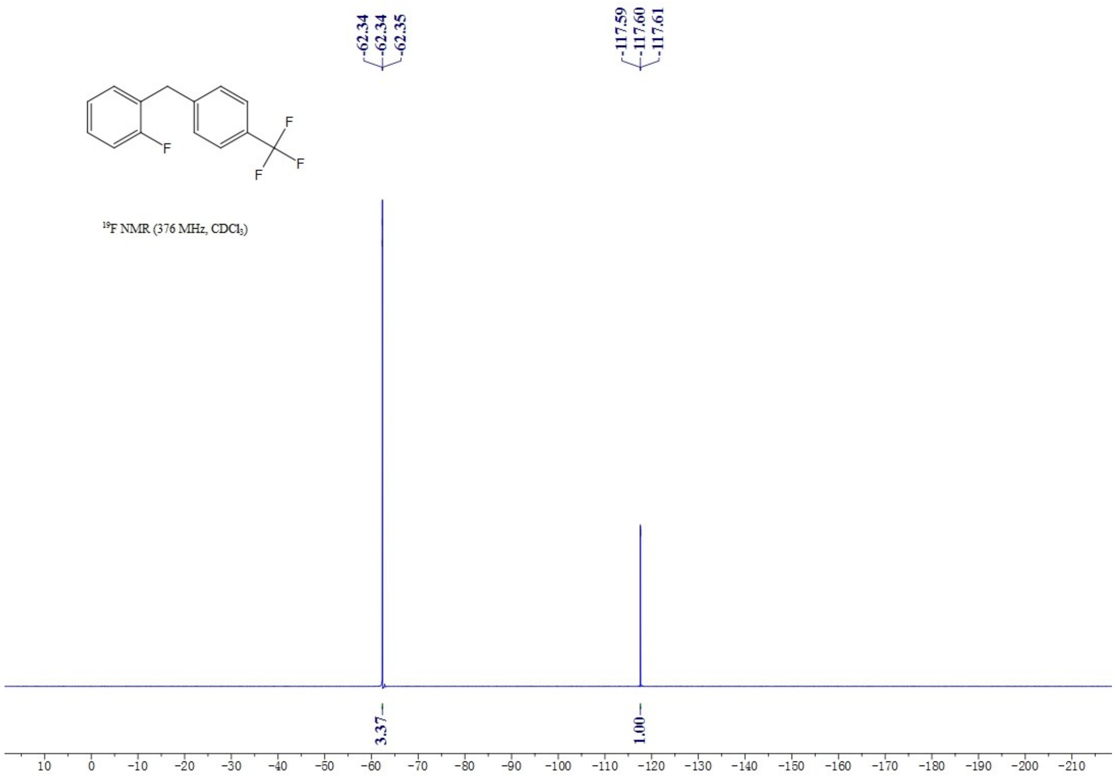

^{1}H, ^{19}F and ^{13}C NMR spectra of compound **3p**

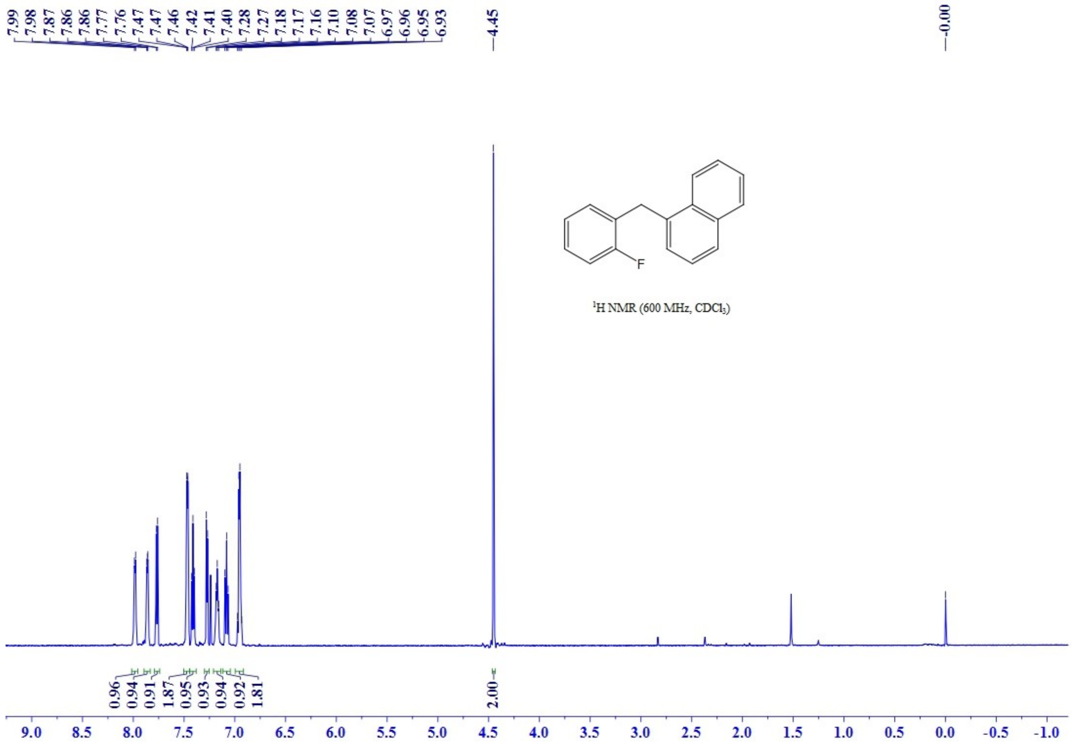

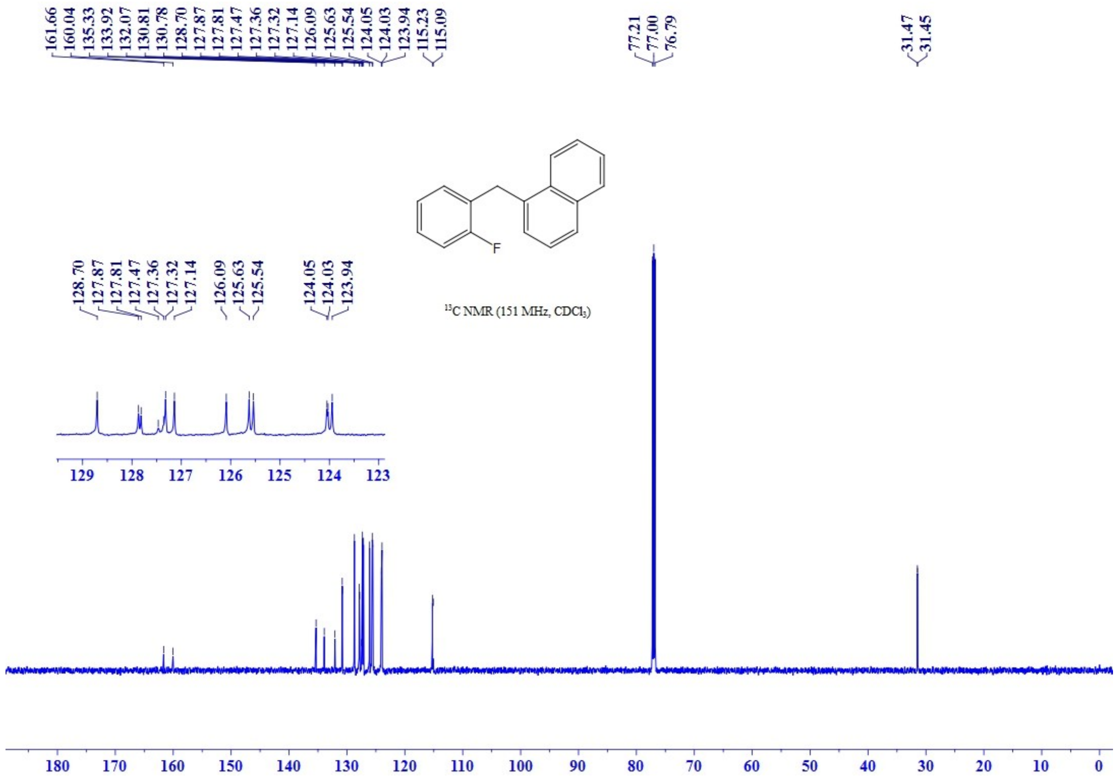
161.66
160.04
135.33
133.92
132.07
130.81
130.78
128.70
127.87
127.81
127.47
127.36
127.32
127.14
126.09
125.63
125.54
124.05
124.03
123.94
115.23
115.09
77.21
77.00
76.79
31.47
31.45
F
13C NMR (151 MHz, CDCl3)
128.70
127.87
127.81
127.47
127.36
127.32
127.14
126.09
125.63
125.54
124.05
124.03
123.94
129 128 127 126 125 124 123
180 170 160 150 140 130 120 110 100 90 80 70 60 50 40 30 20 10 0

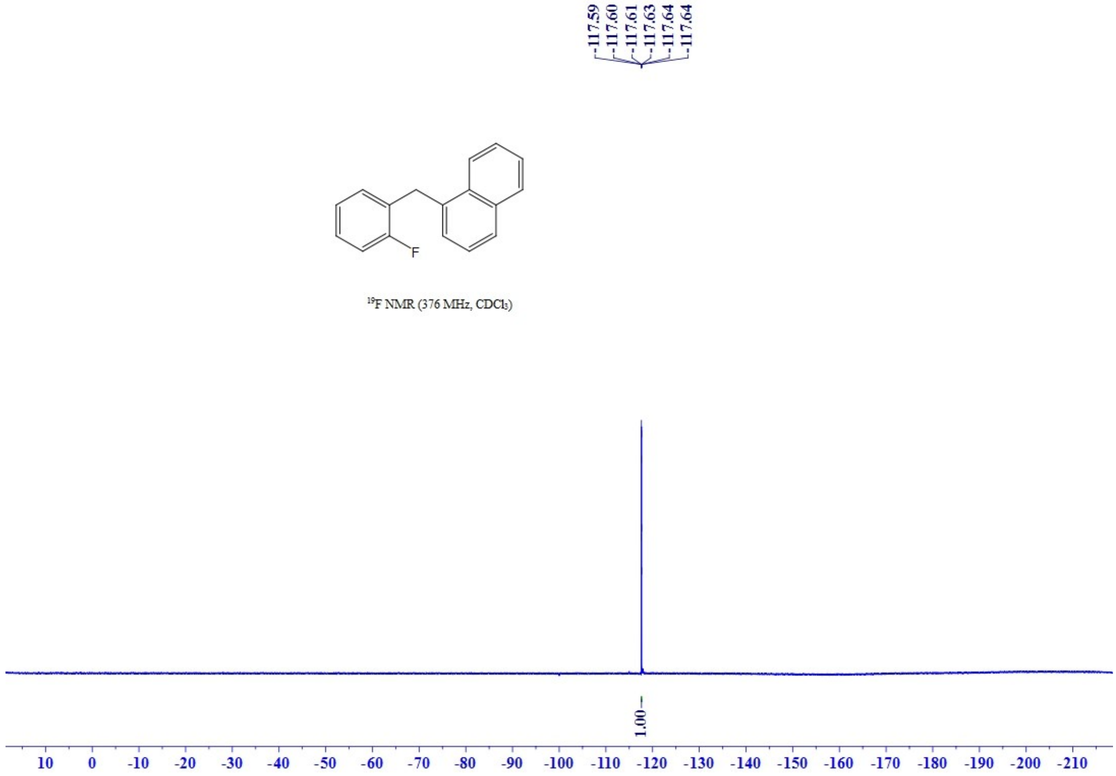
-117.59
-117.60
-117.61
-117.63
-117.64
-117.64
F
19F NMR (376 MHz, CDCl3)
1.00
10 0 -10 -20 -30 -40 -50 -60 -70 -80 -90 -100 -110 -120 -130 -140 -150 -160 -170 -180 -190 -200 -210

^{1}H, ^{19}F and ^{13}C NMR spectra of compound **3q**

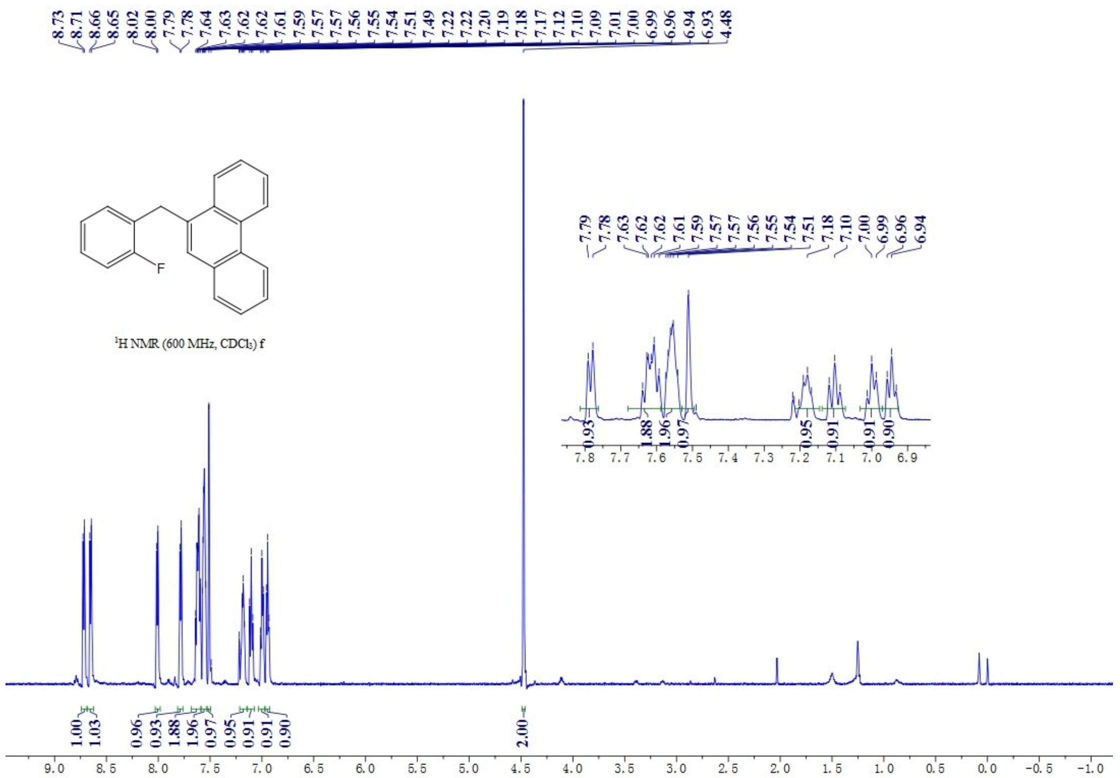

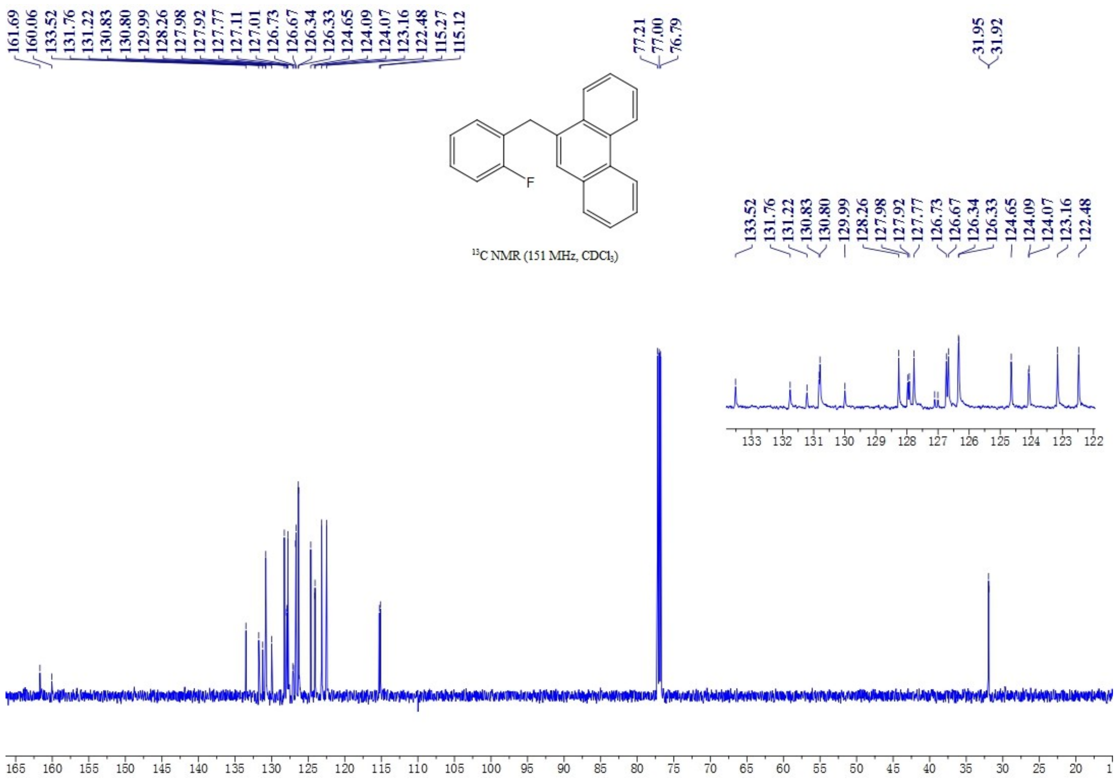

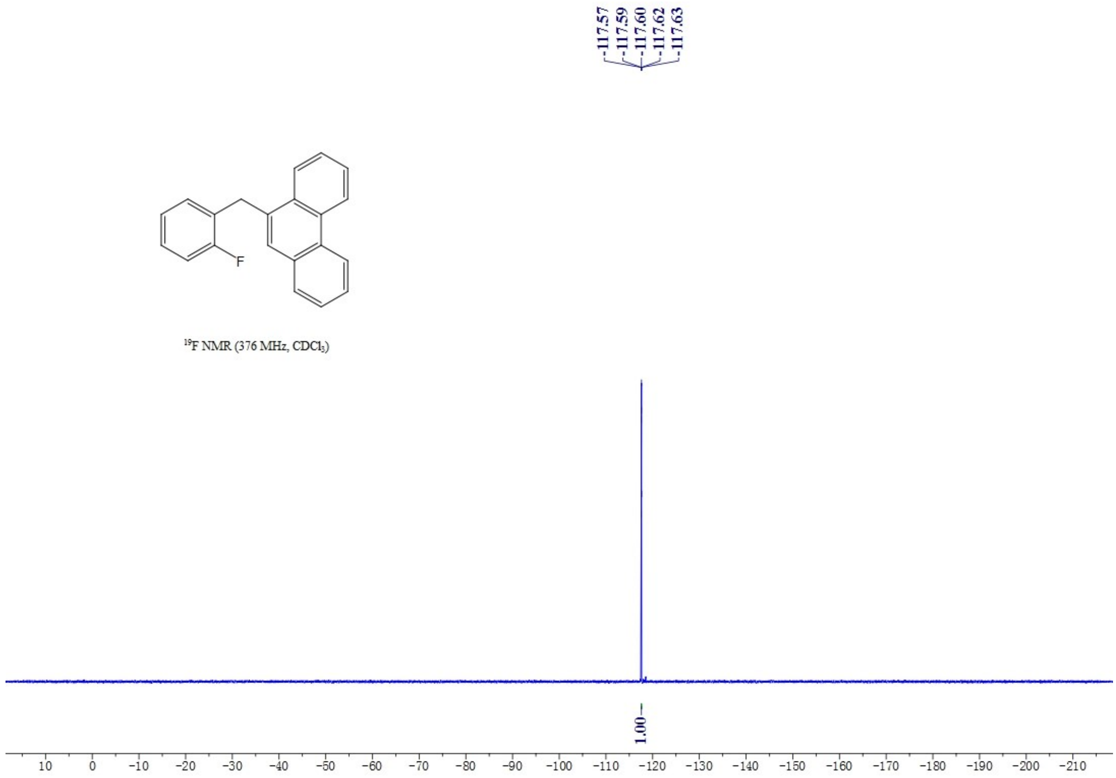

^{1}H, ^{19}F and ^{13}C NMR spectra of compound **3r**

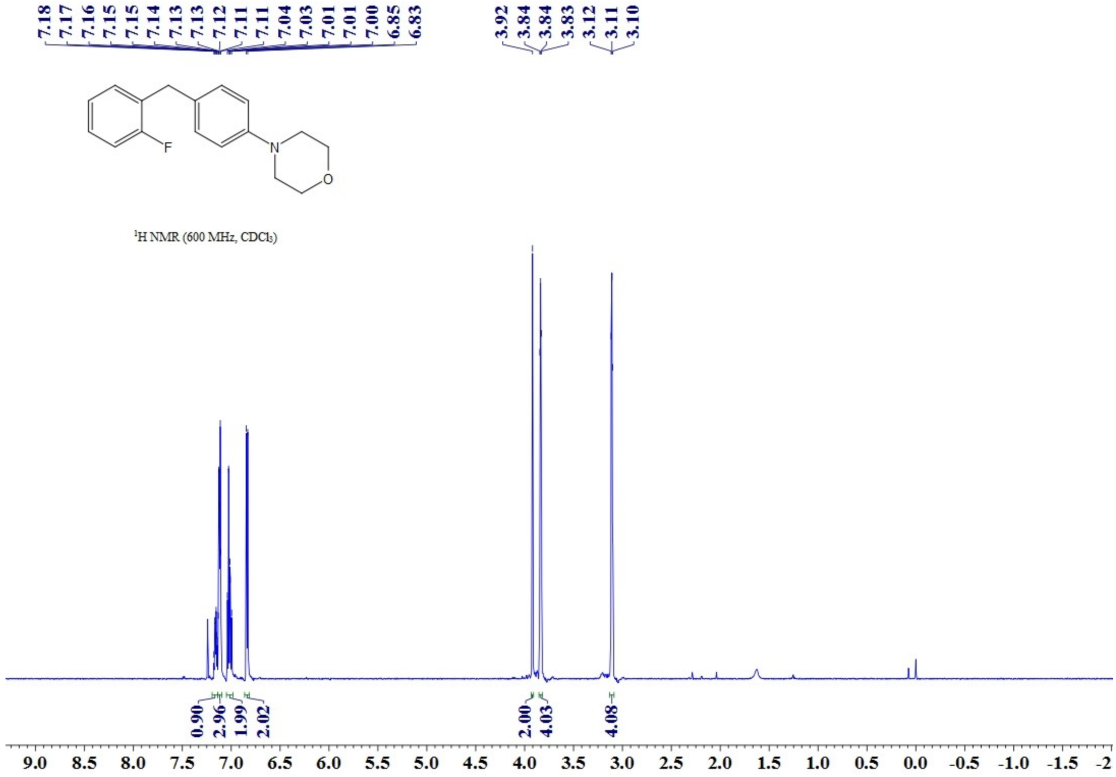

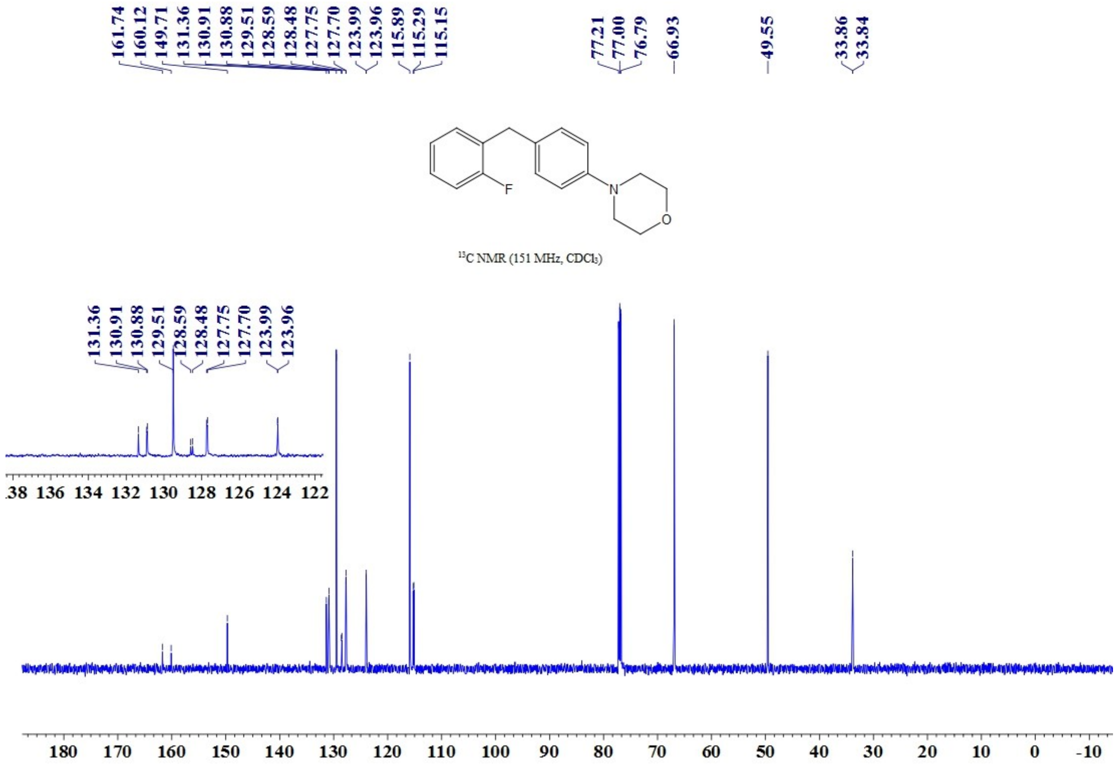

^{13}C NMR (151 MHz, $CDCl_3$)

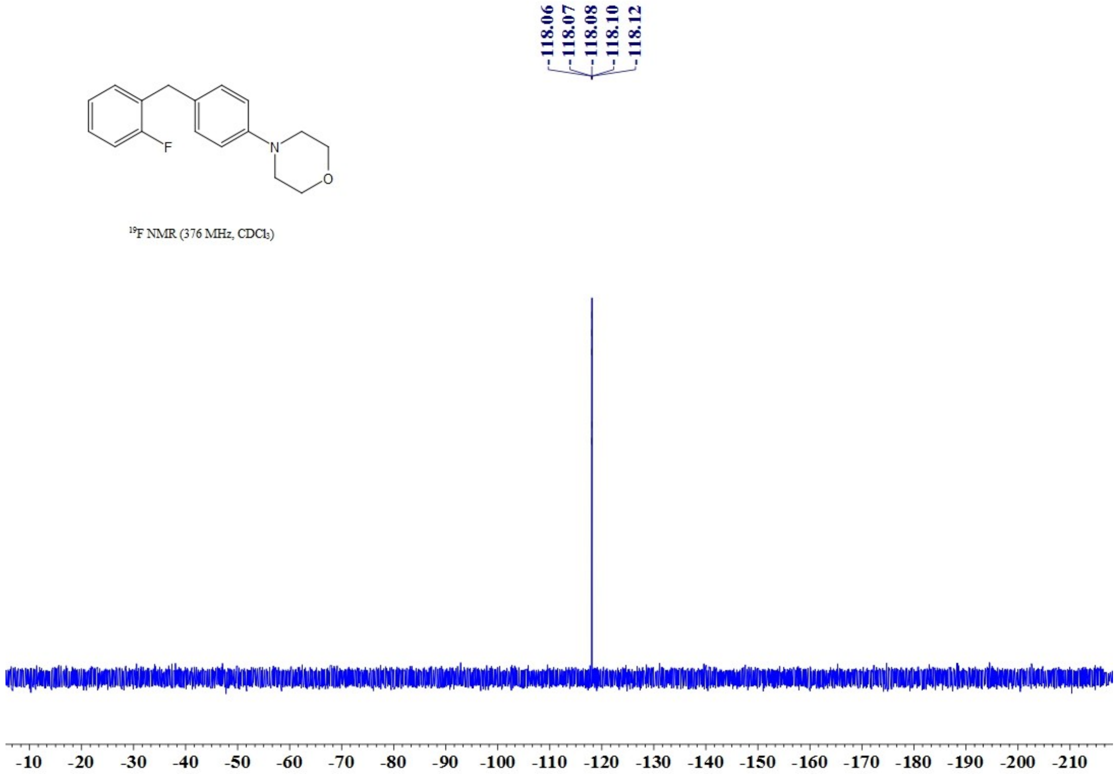

^{19}F NMR (376 MHz, $CDCl_3$)

^{1}H, ^{19}F and ^{13}C NMR spectra of compound **3s**

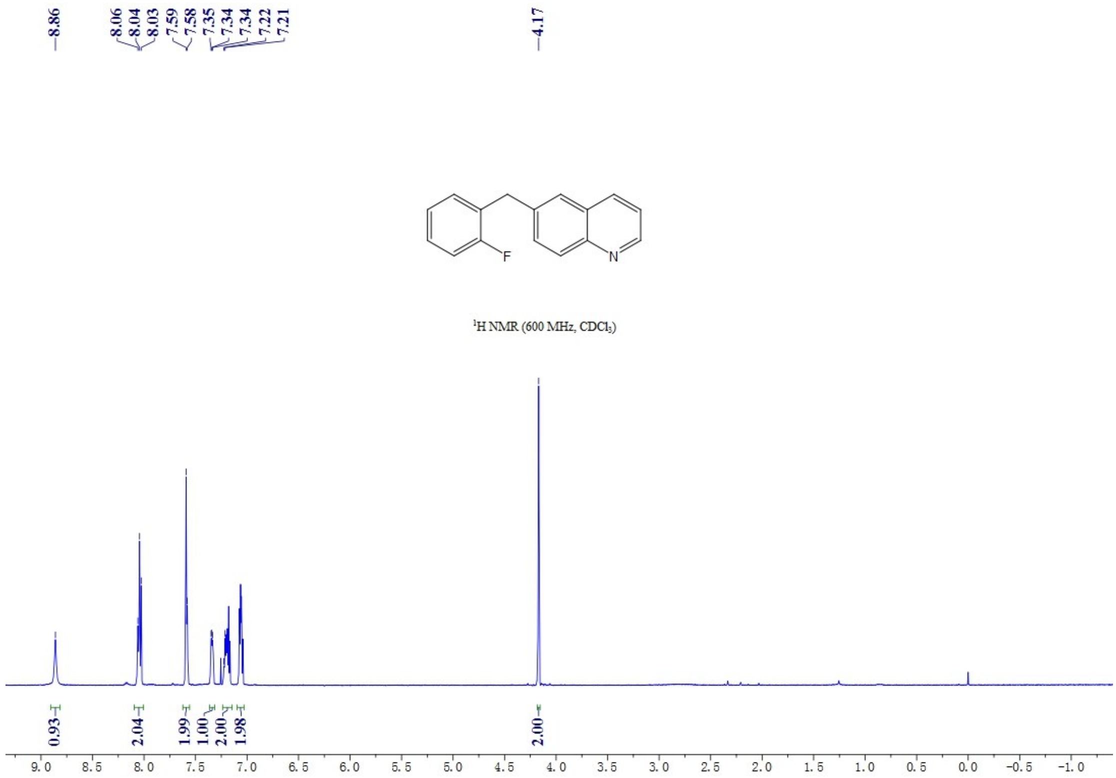

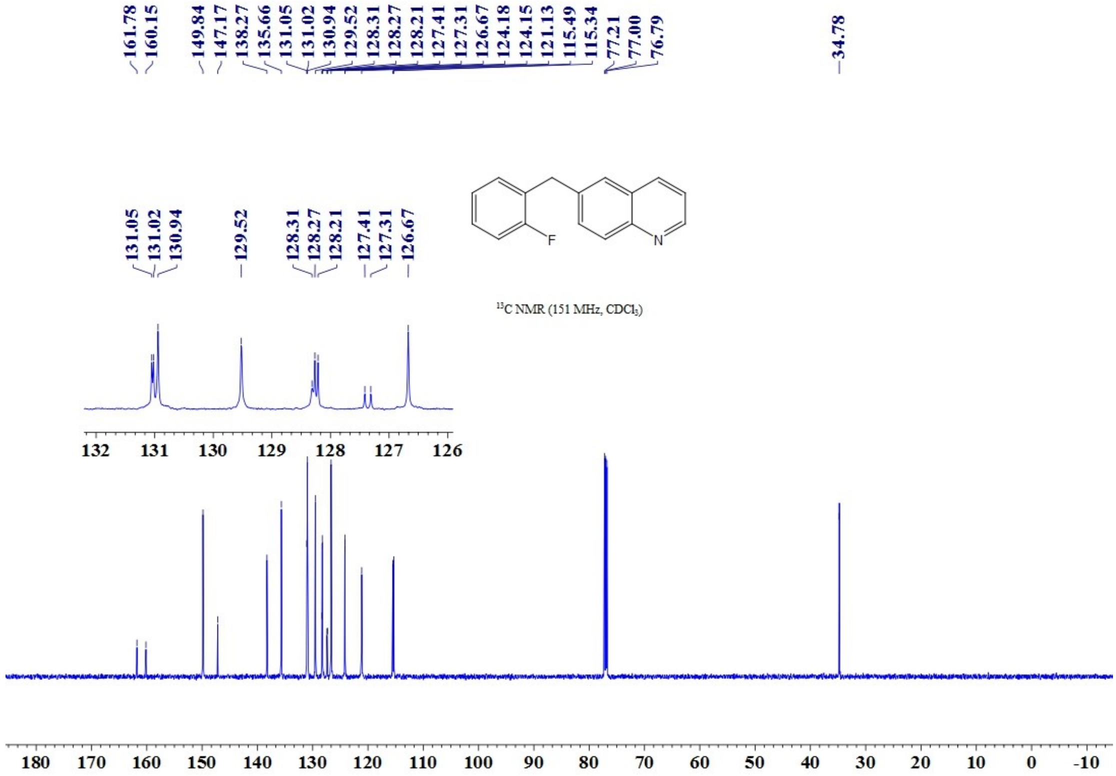

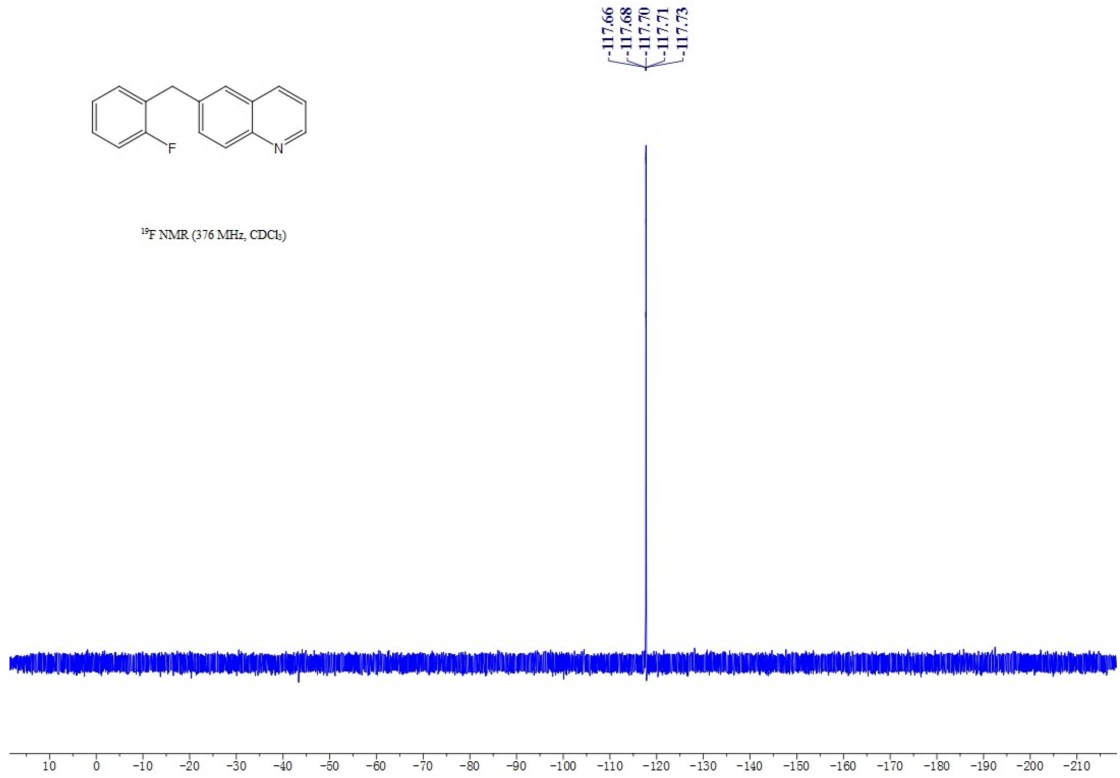

^{1}H, ^{19}F and ^{13}C NMR spectra of compound **3t**

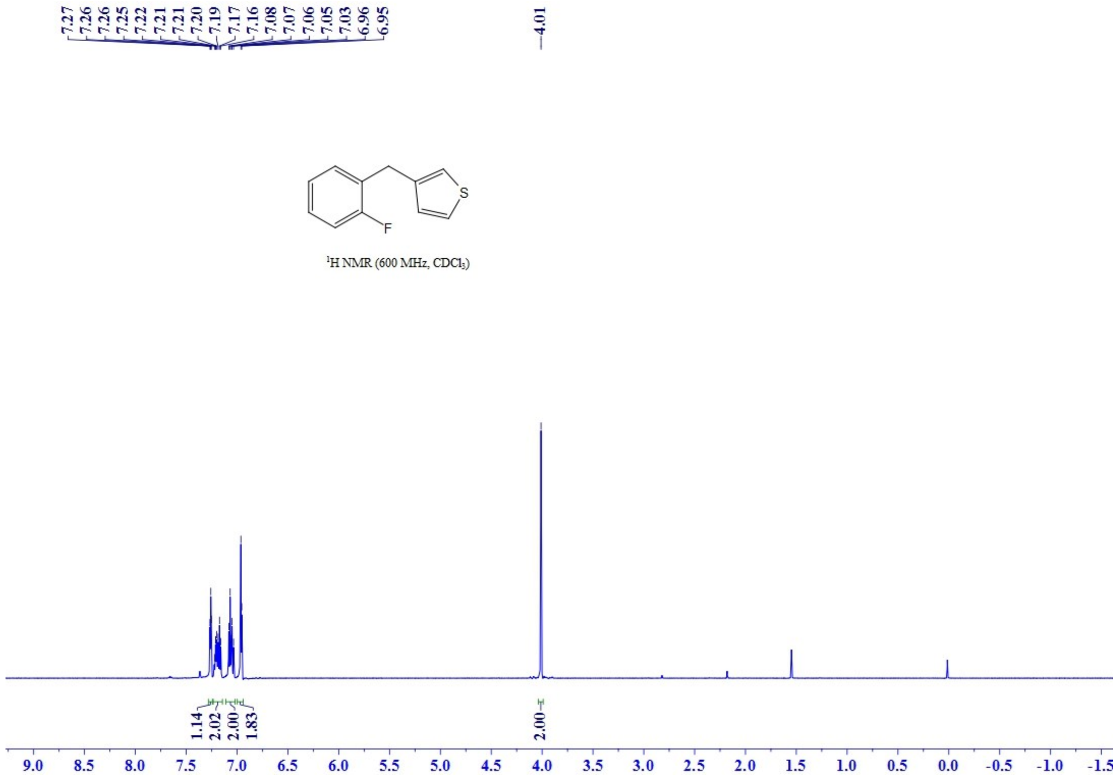

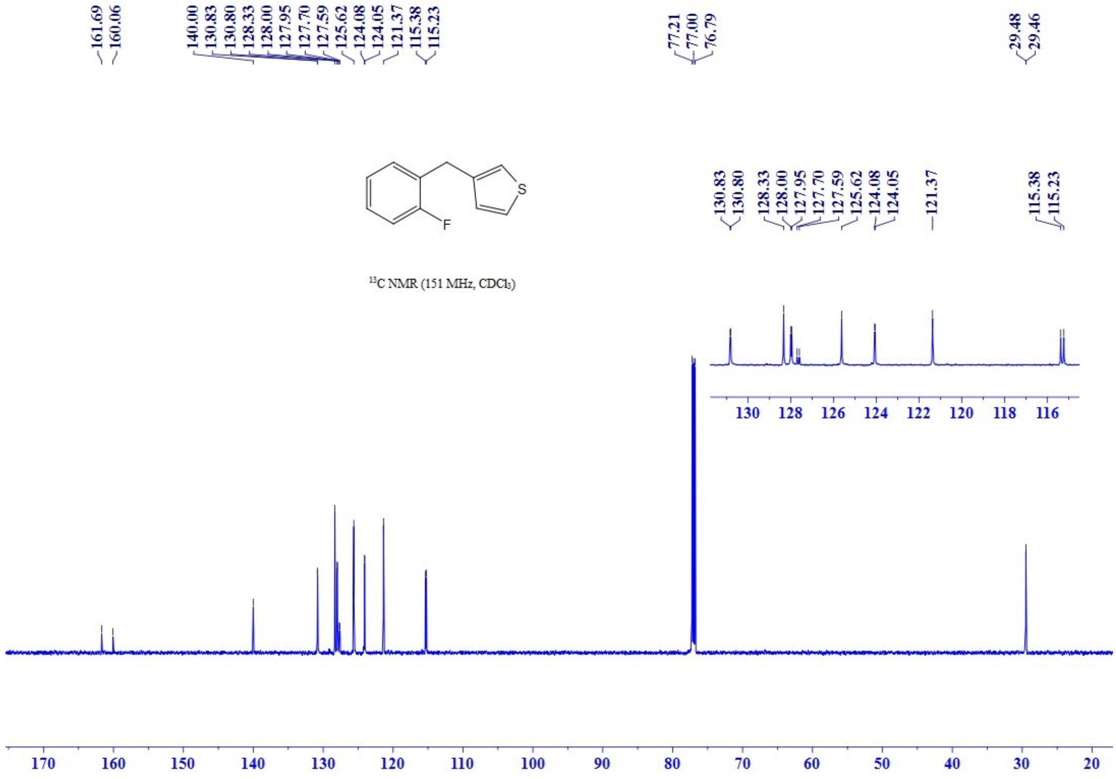
161.69
160.06
140.00
130.83
130.80
128.33
128.00
127.95
127.70
127.59
125.62
124.08
124.05
121.37
115.38
115.23
77.21
77.00
76.79
29.48
29.46
S
F
13C NMR (151 MHz, CDCl3)
130 128 126 124 122 120 118 116
170 160 150 140 130 120 110 100 90 80 70 60 50 40 30 20

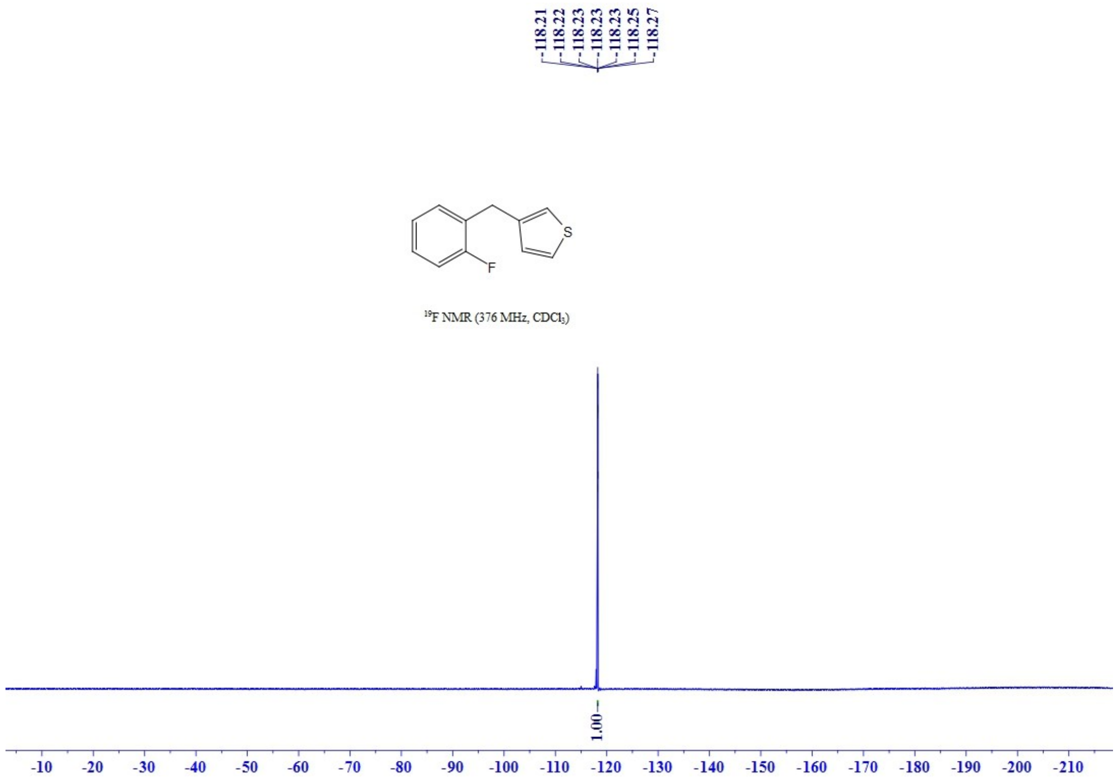
-118.21
-118.22
-118.23
-118.23
-118.23
-118.25
-118.27
S
F
19F NMR (376 MHz, CDCl3)
1.00
-10 -20 -30 -40 -50 -60 -70 -80 -90 -100 -110 -120 -130 -140 -150 -160 -170 -180 -190 -200 -210

^{1}H, ^{19}F and ^{13}C NMR spectra of compound **3u**

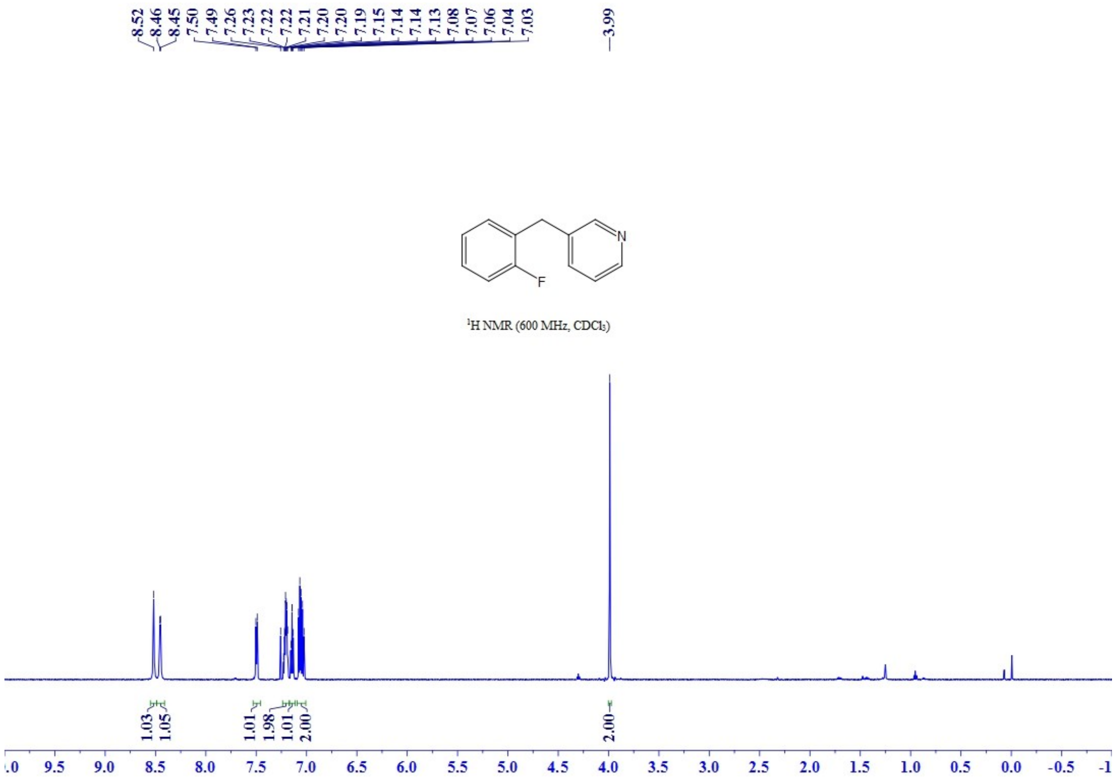

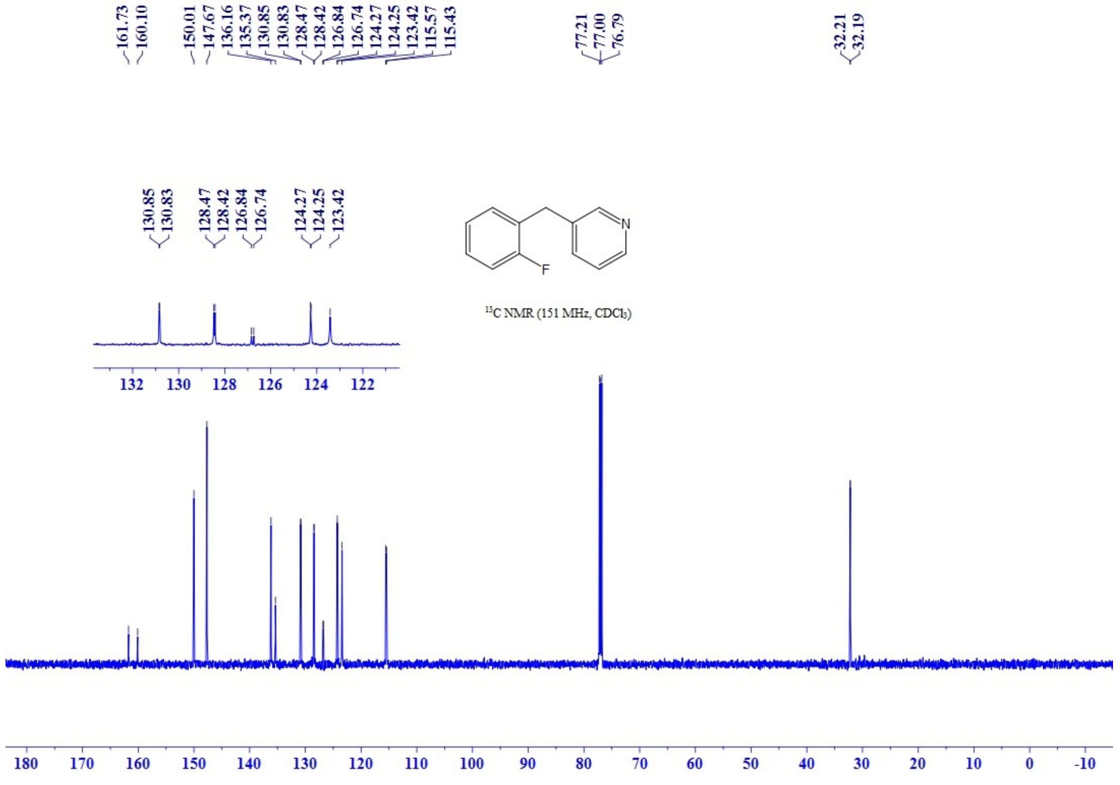

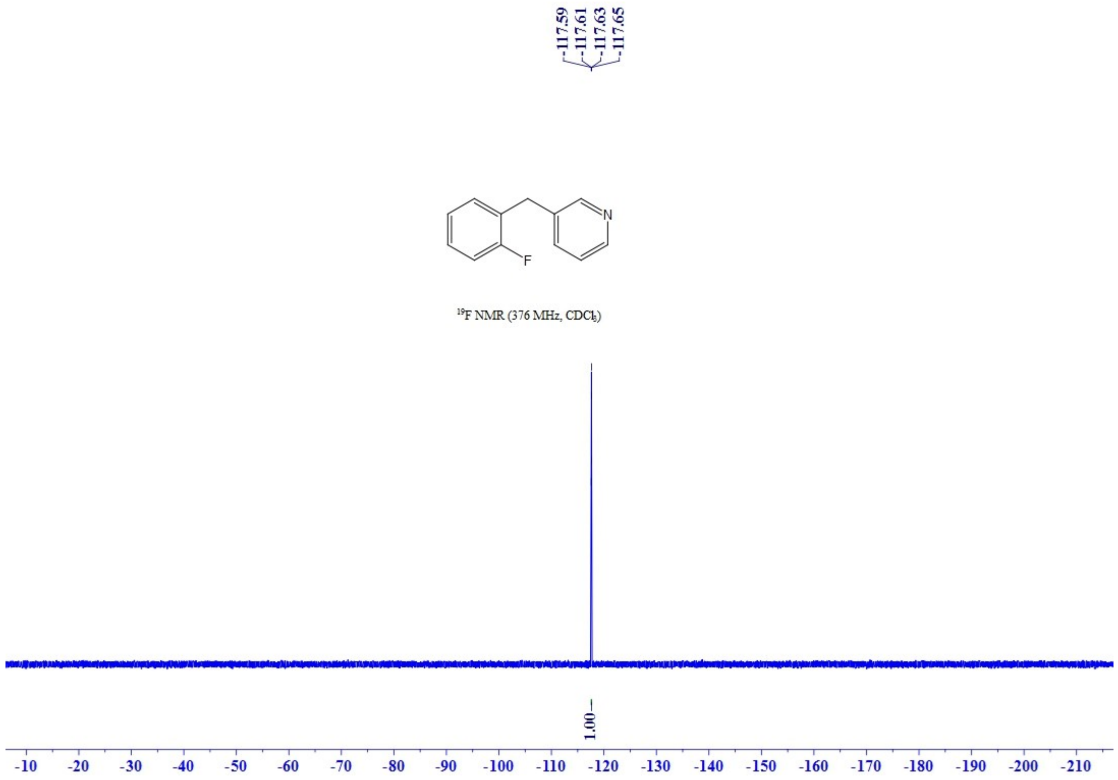

^{1}H and ^{13}C NMR spectra of compound **3v**

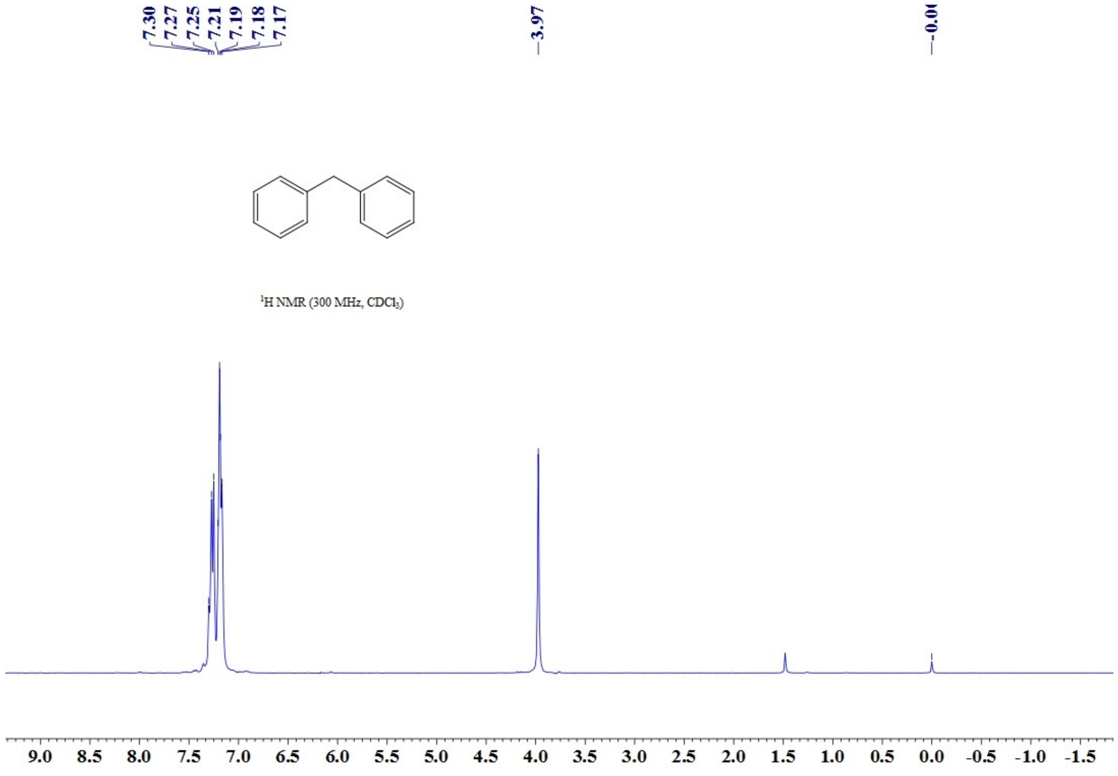

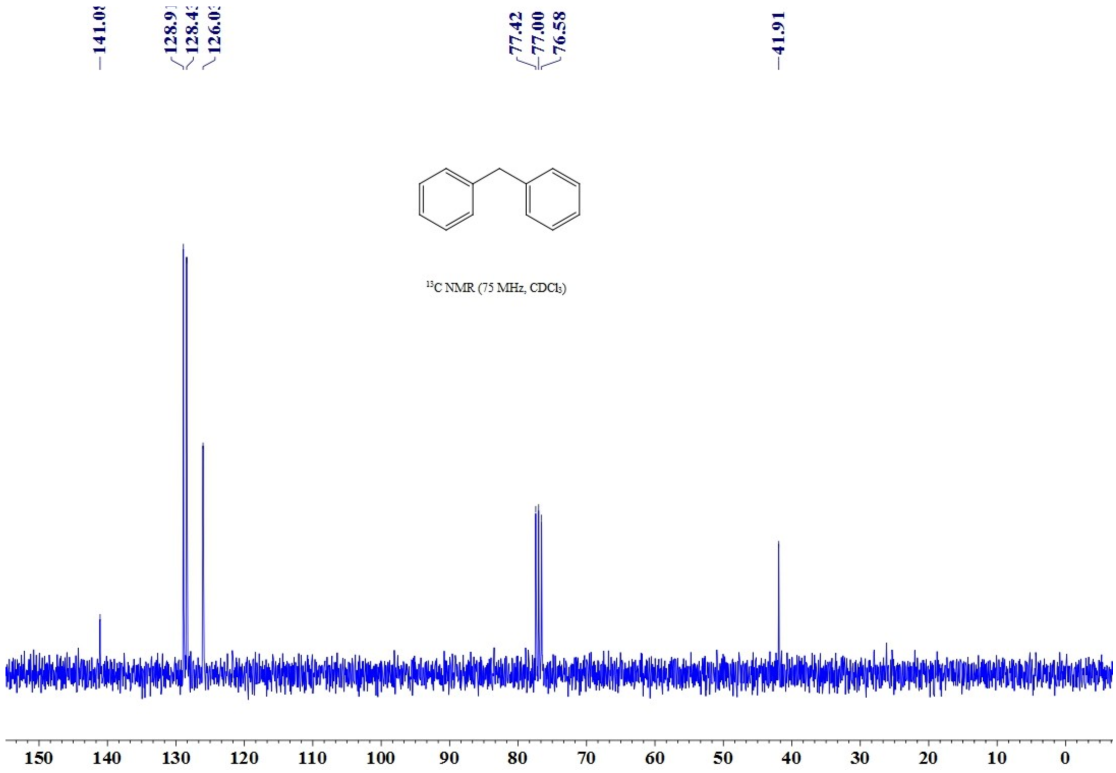

^{1}H and ^{13}C NMR spectra of compound **3w**

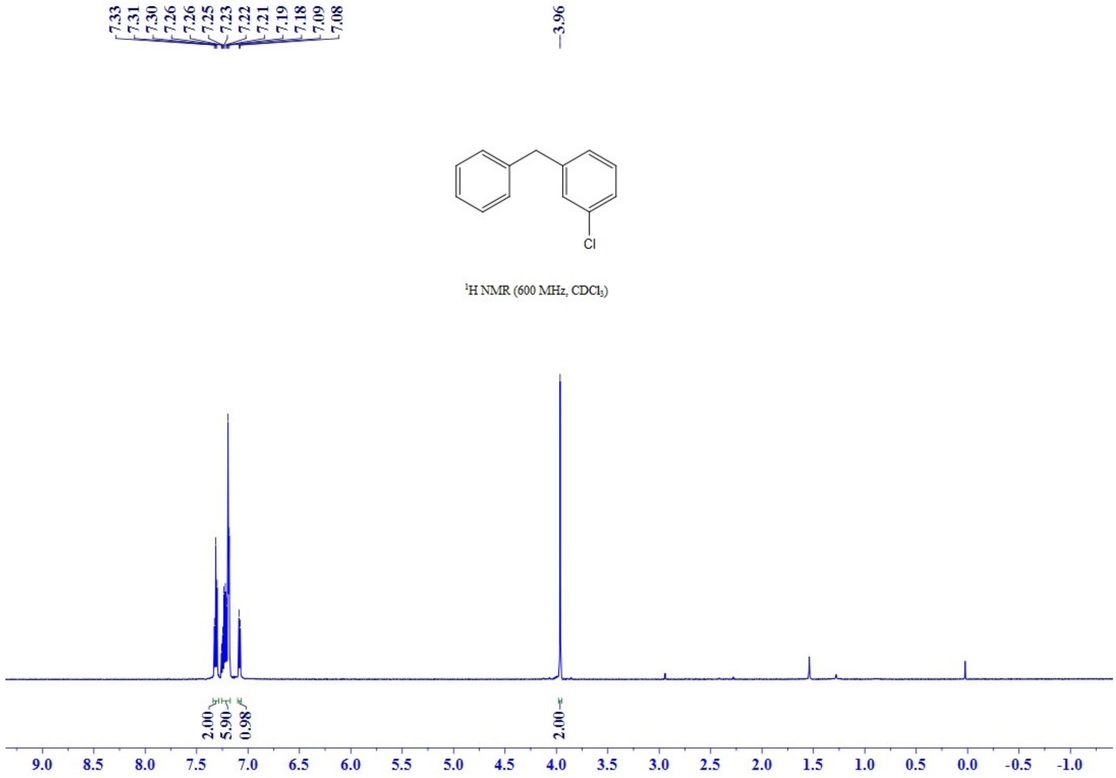

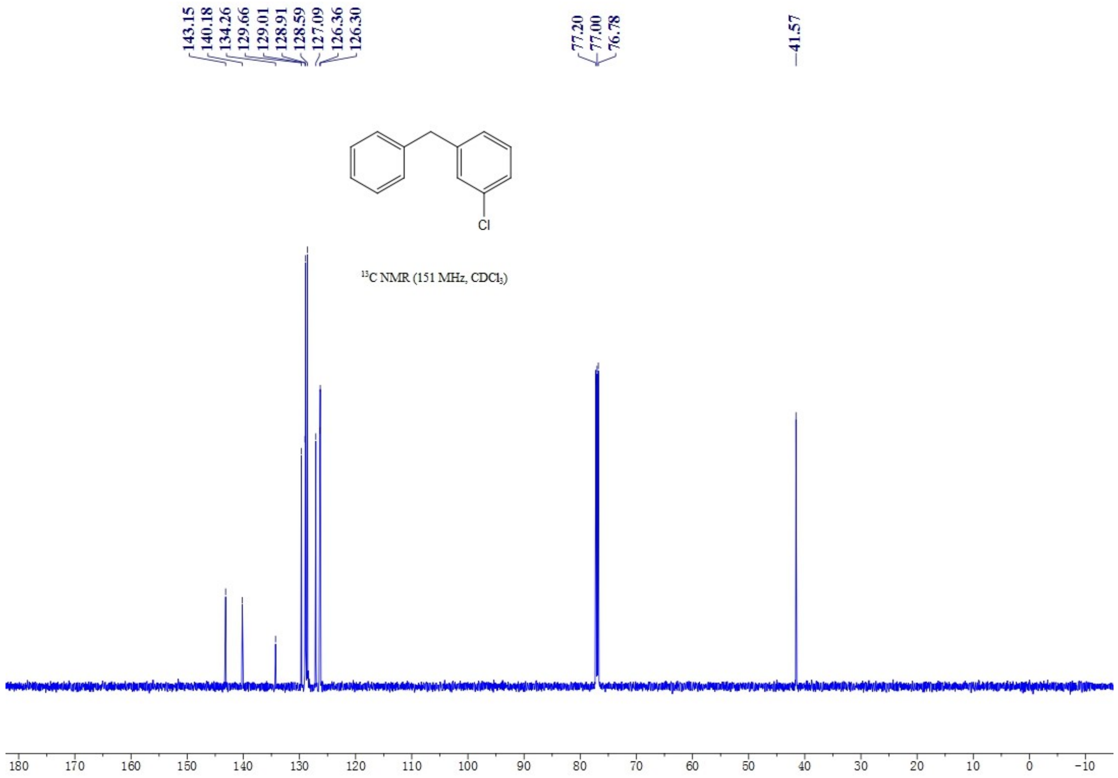

^1H and ^{13}C NMR spectra of compound **3x**

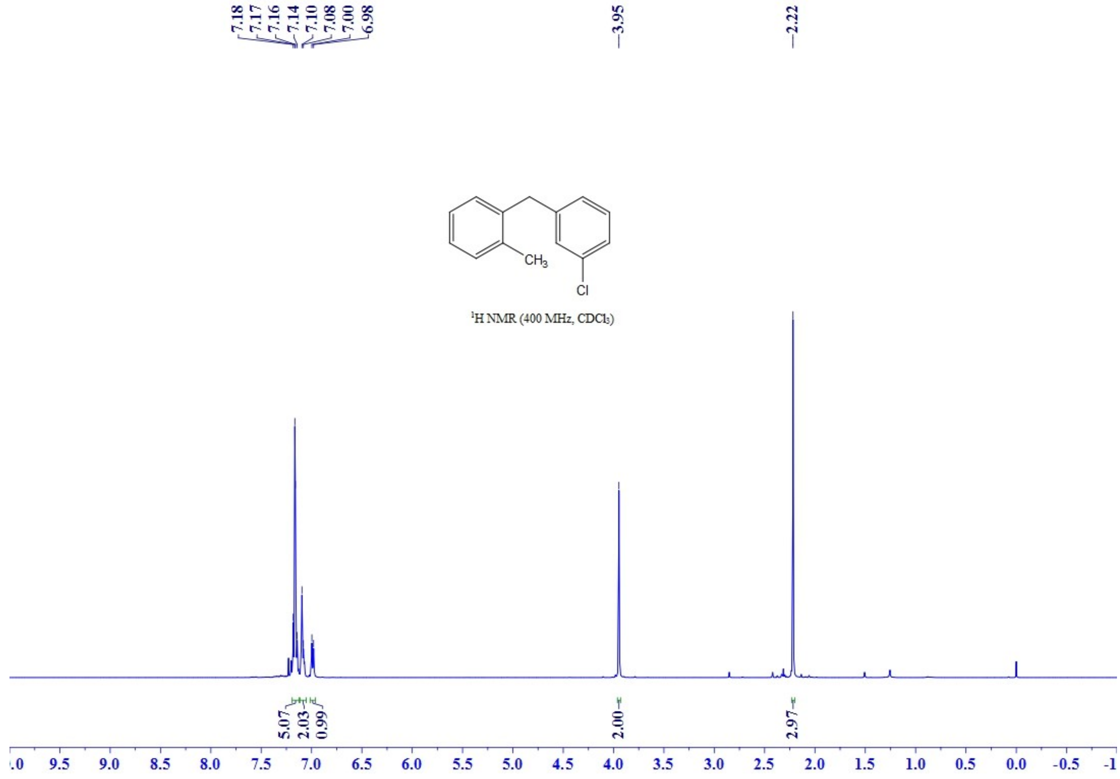

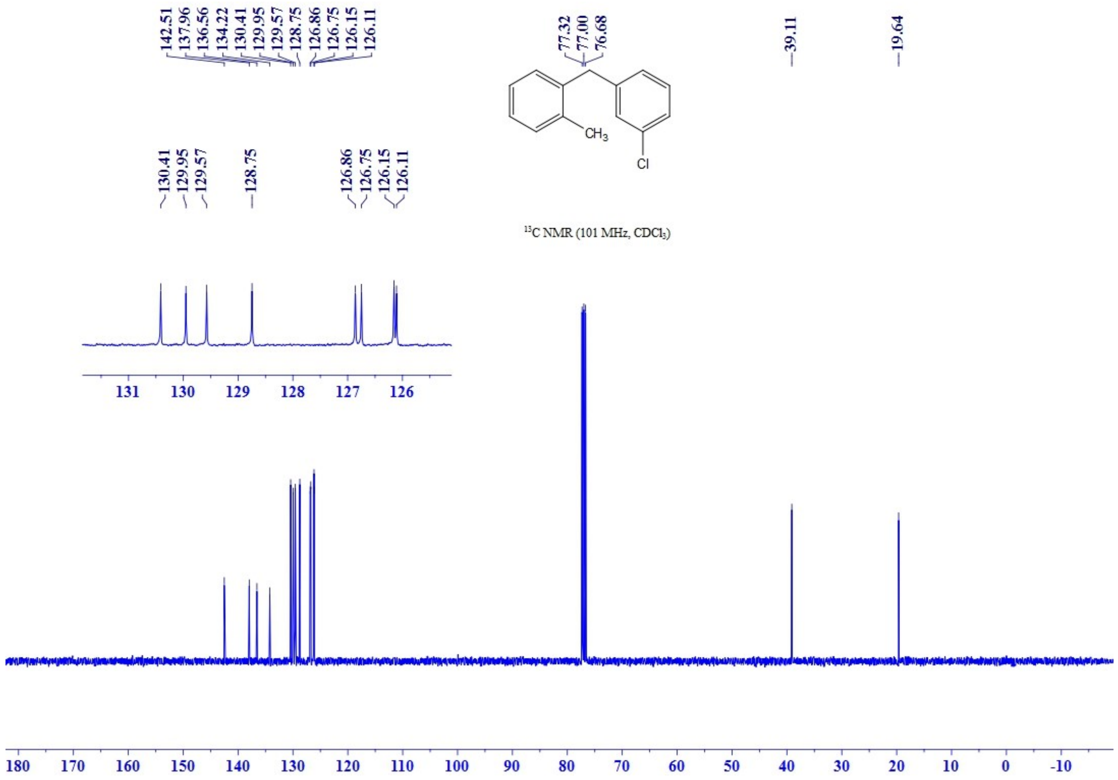

^{1}H and ^{13}C NMR spectra of compound **3y**

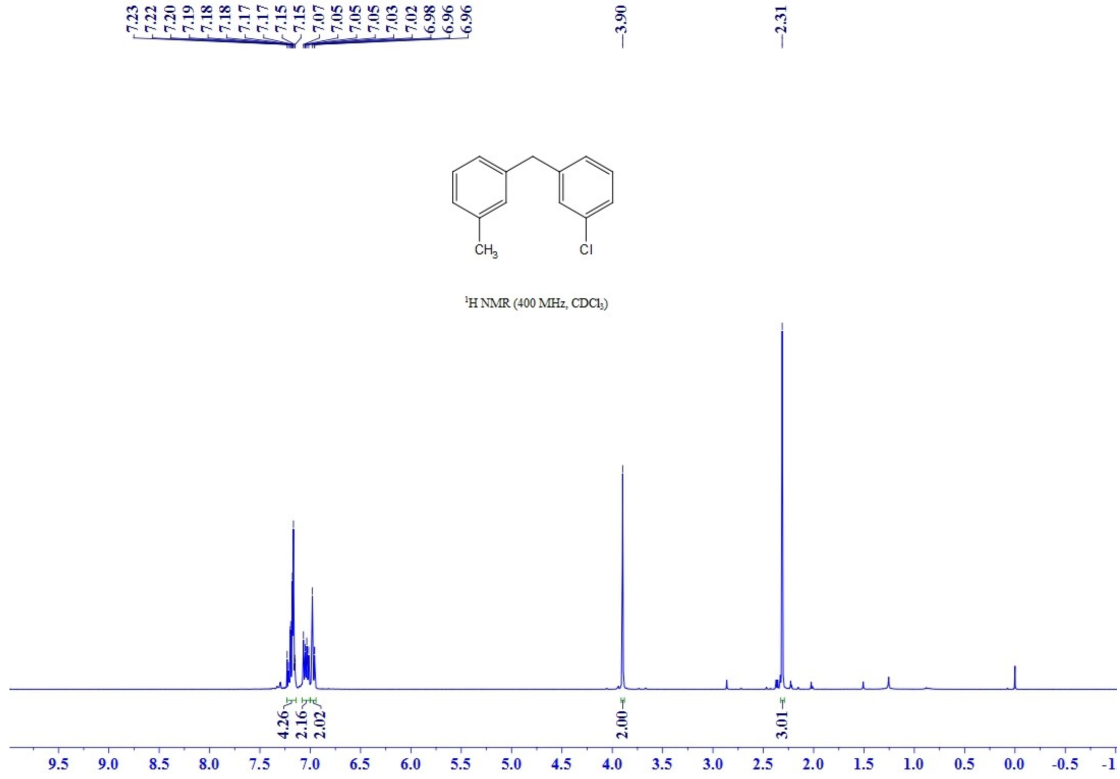

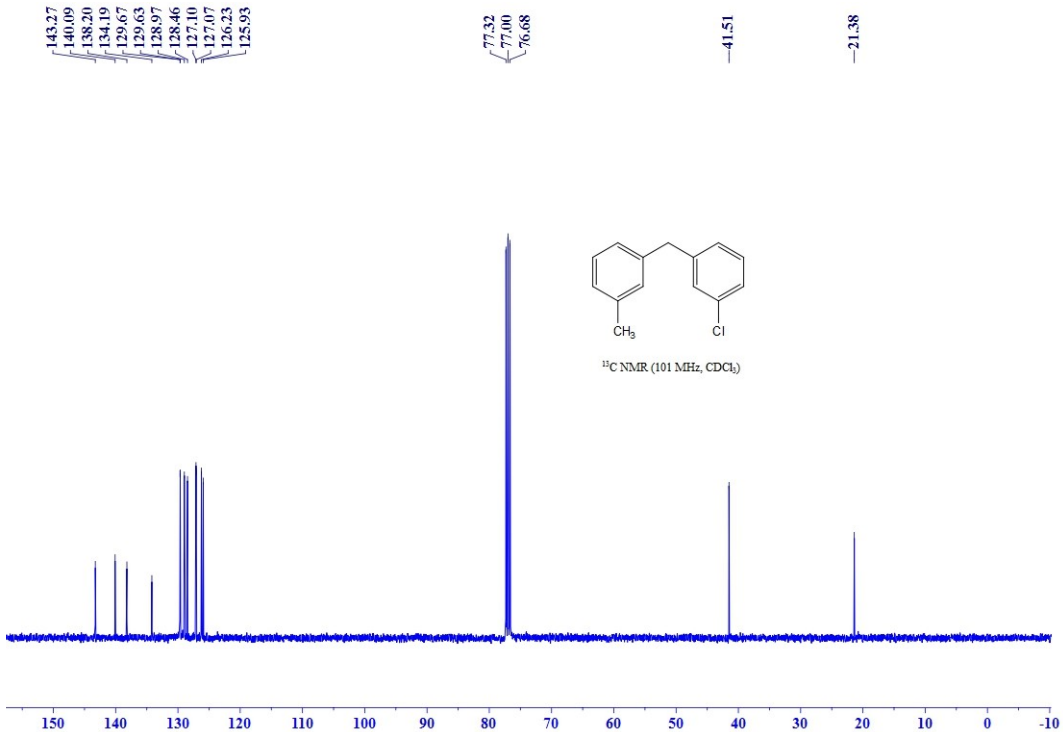

^{1}H and ^{13}C NMR spectra of compound **3z**

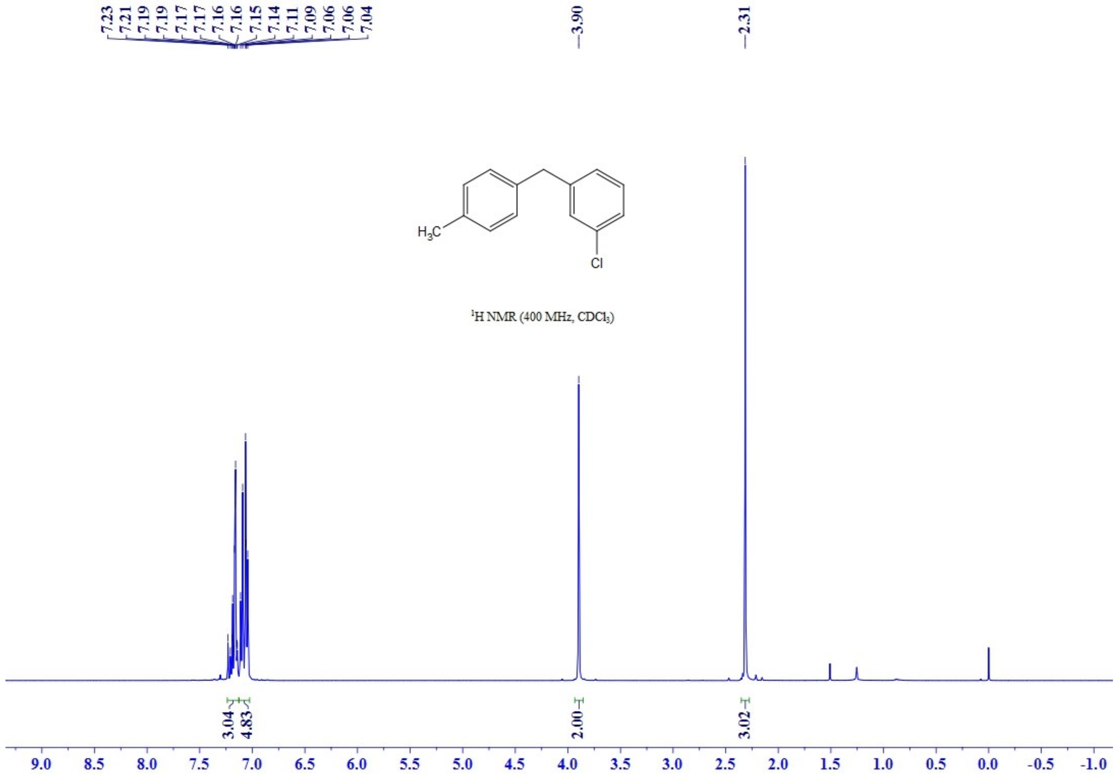

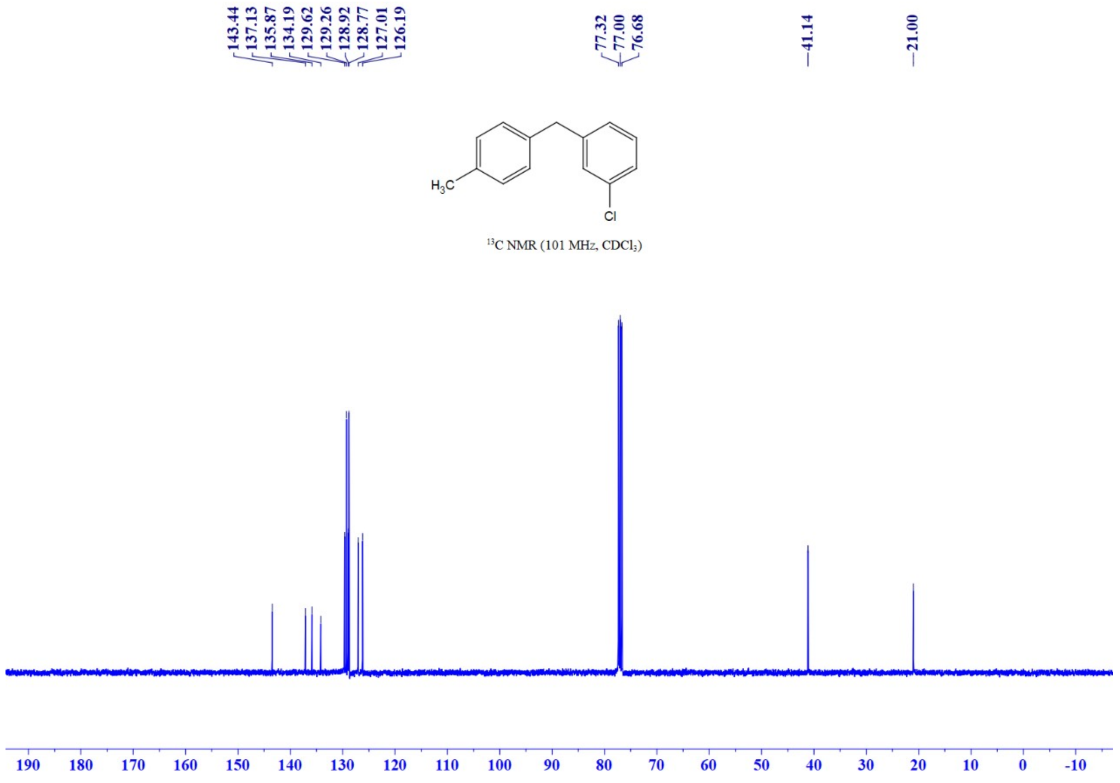

^{1}H and ^{13}C NMR spectra of compound **3aa**

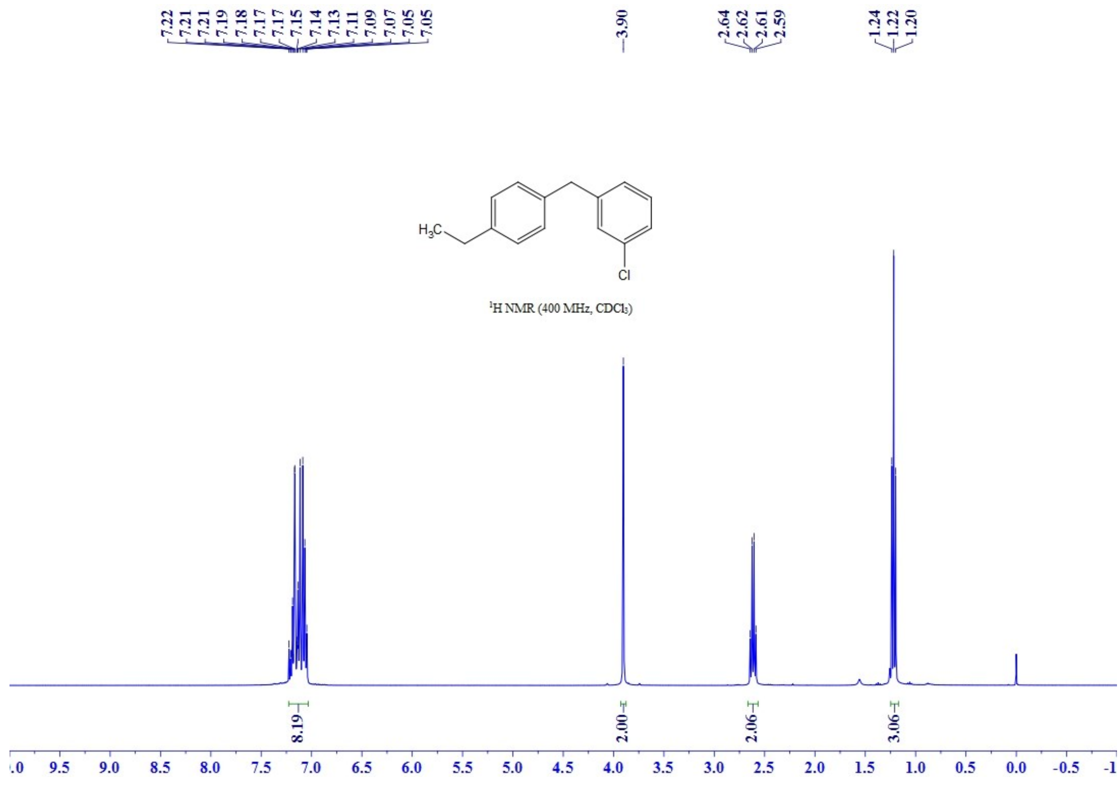

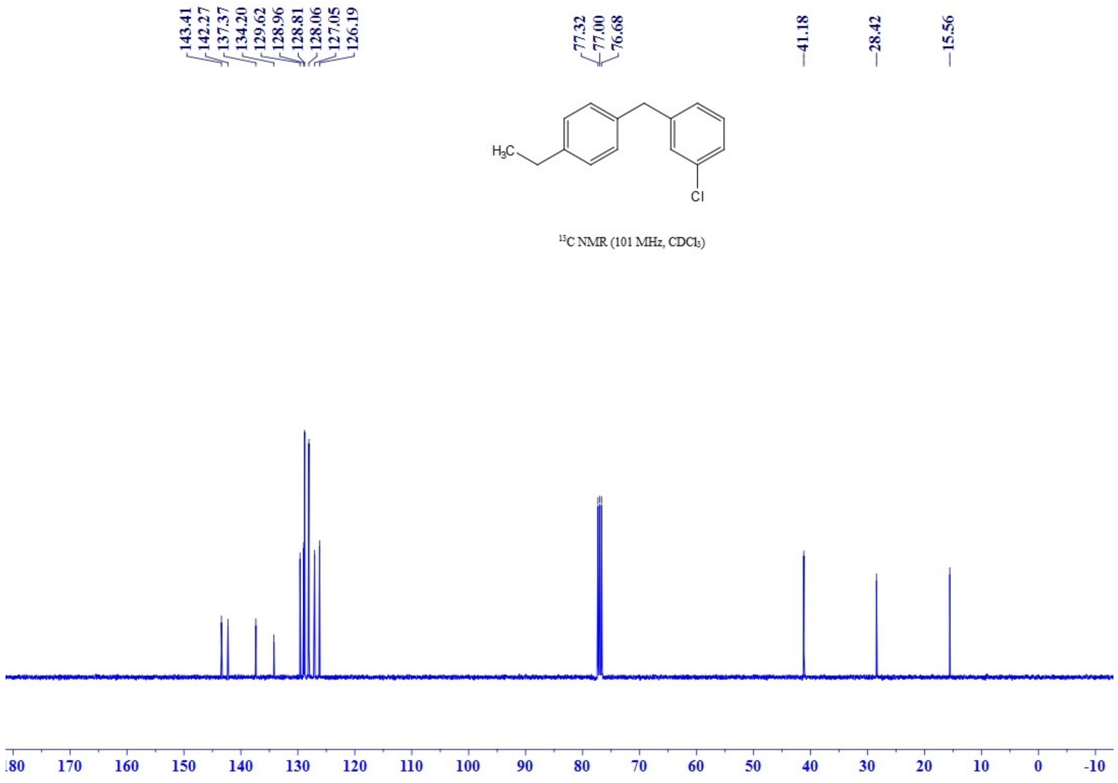

^{1}H and ^{13}C NMR spectra of compound **3ab**

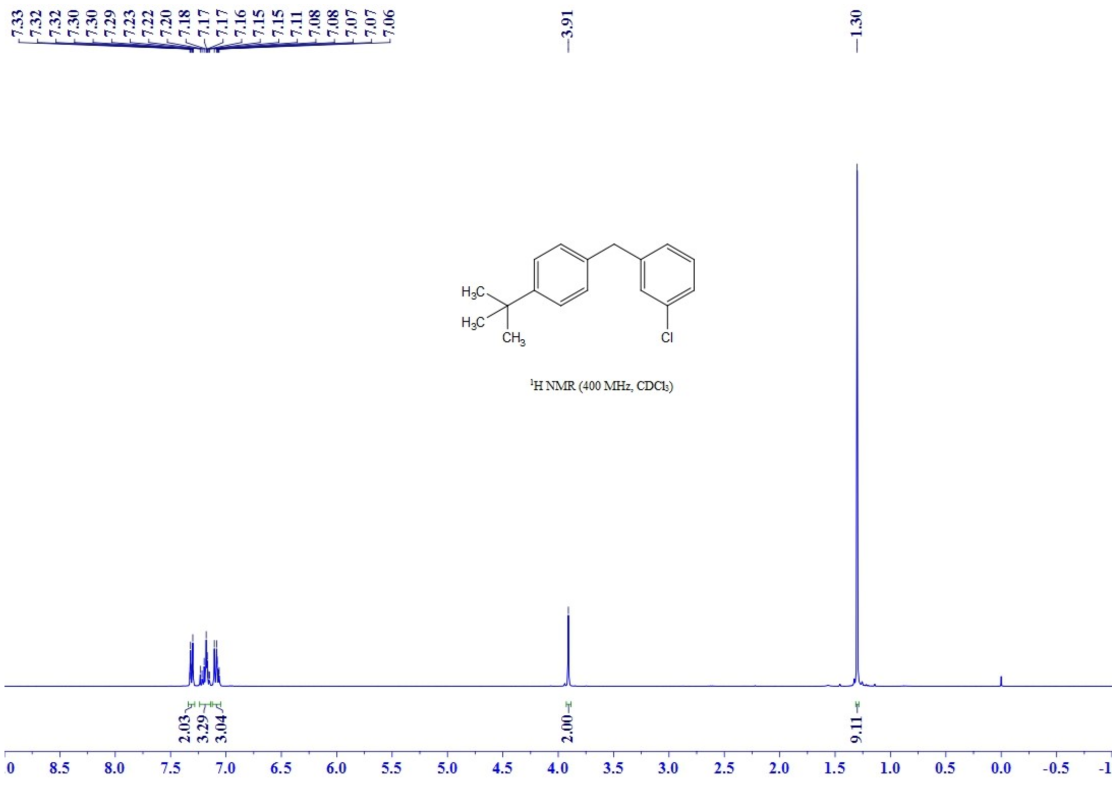

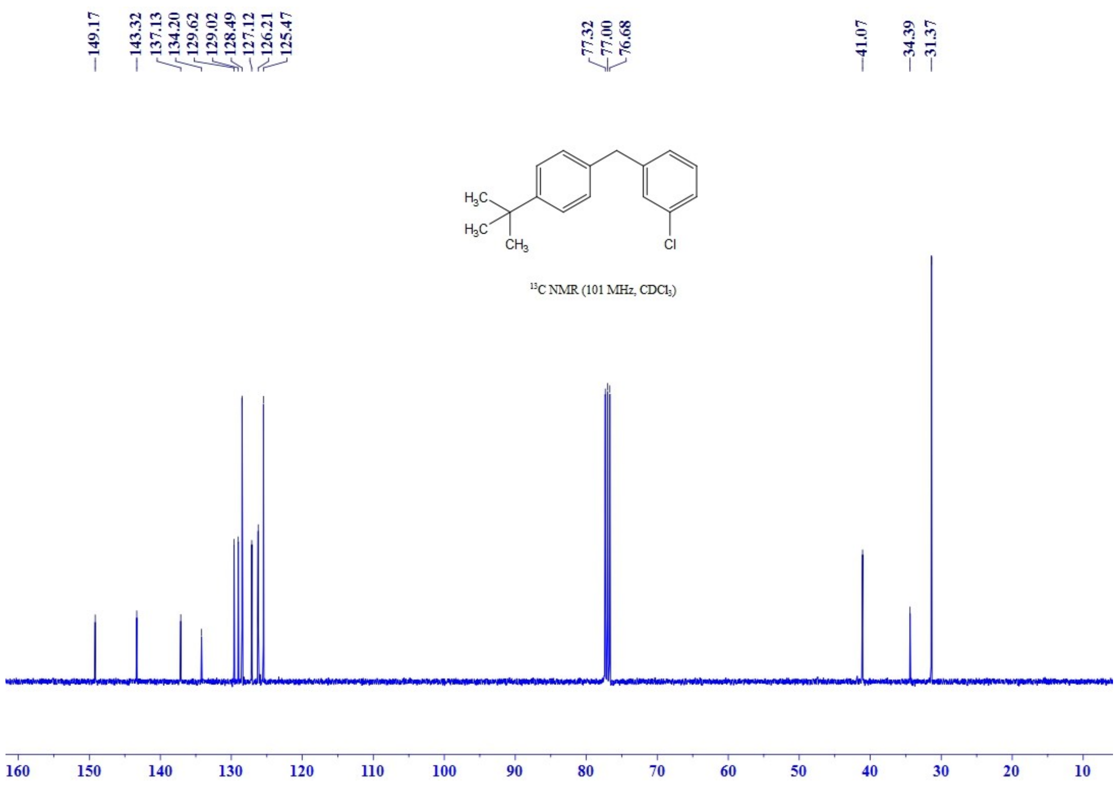

^{1}H and ^{13}C NMR spectra of compound **3ac**

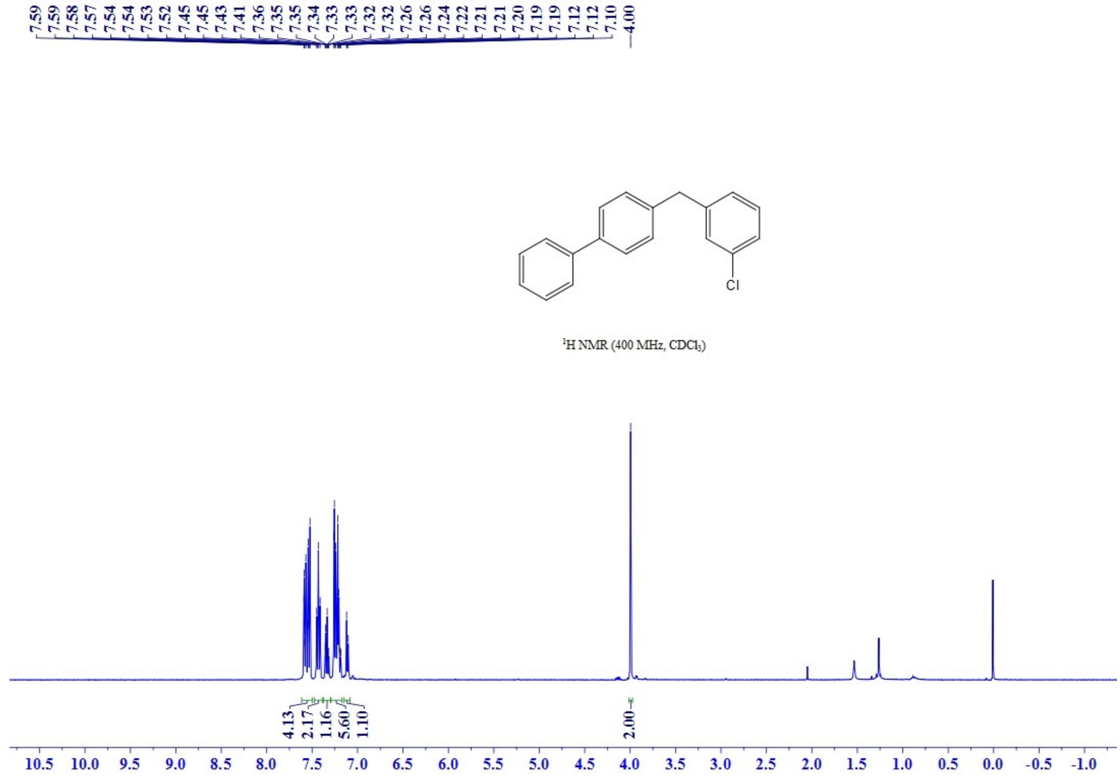

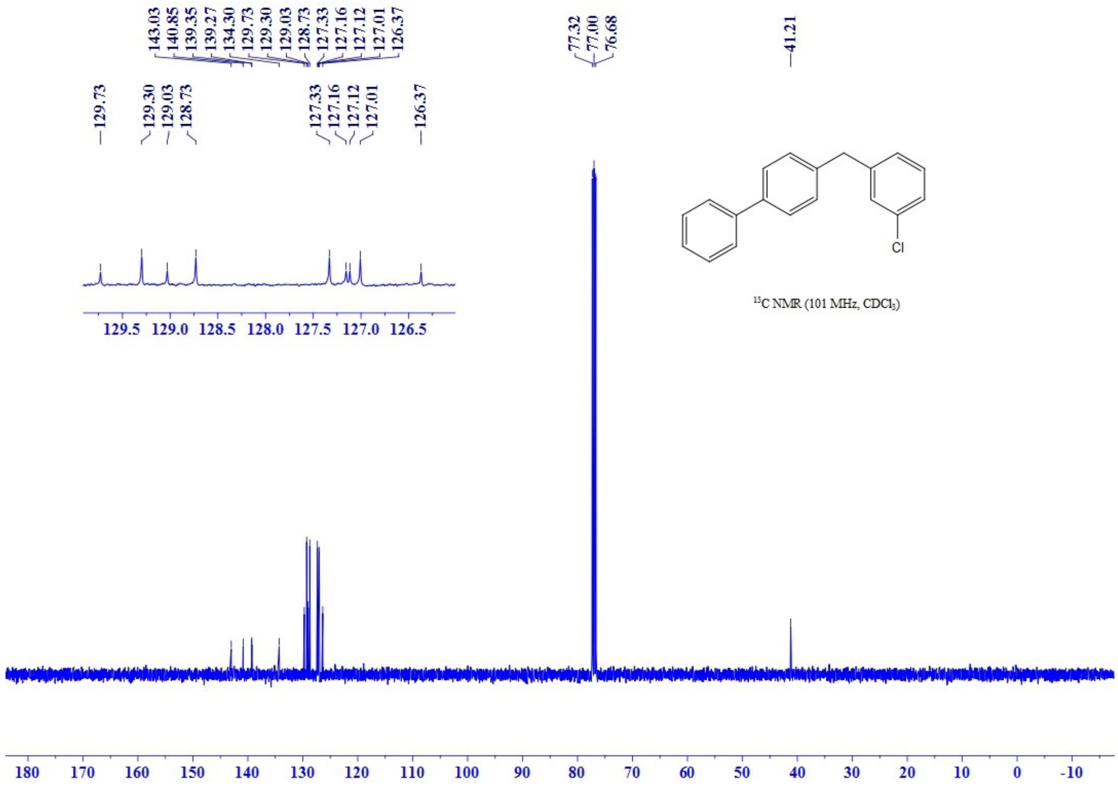

^{1}H and ^{13}C NMR spectra of compound **3ad**

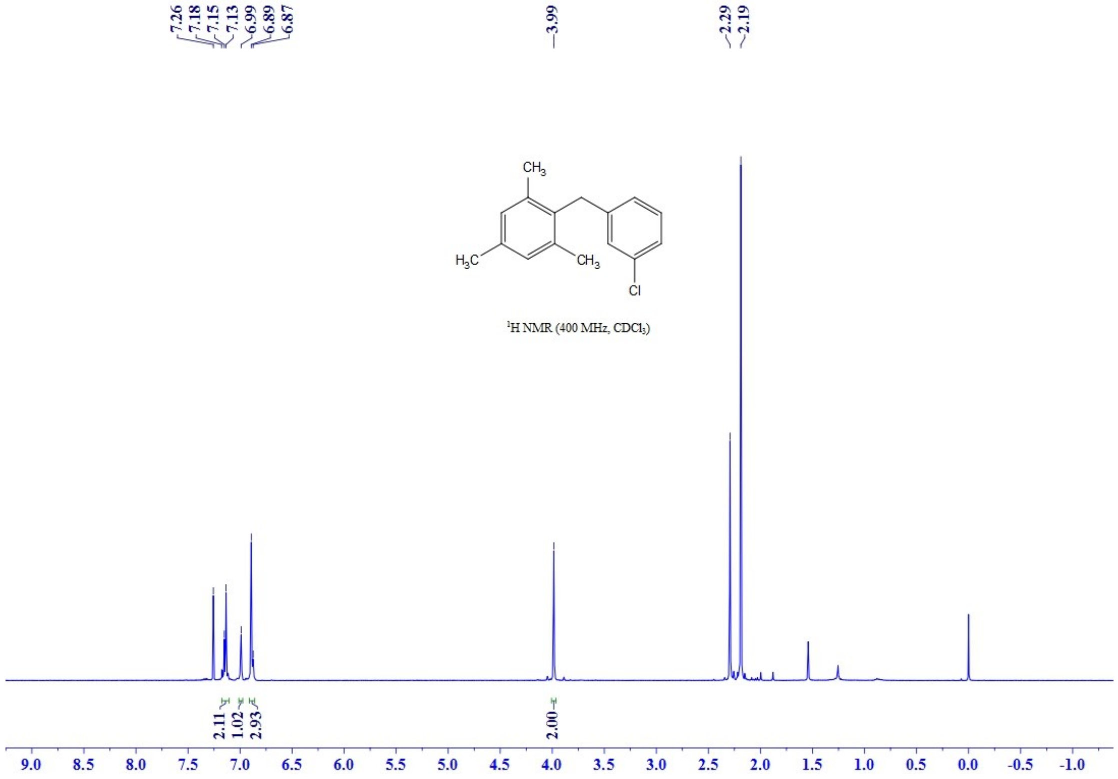

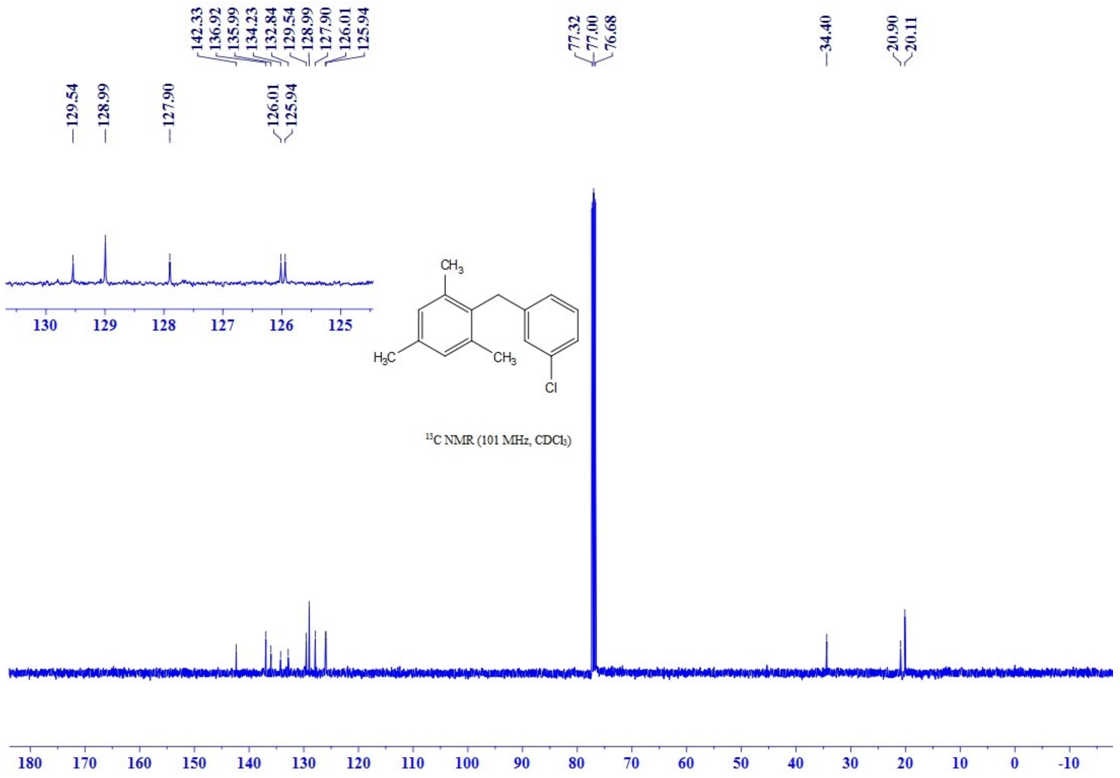

^{1}H and ^{13}C NMR spectra of compound **3ae**

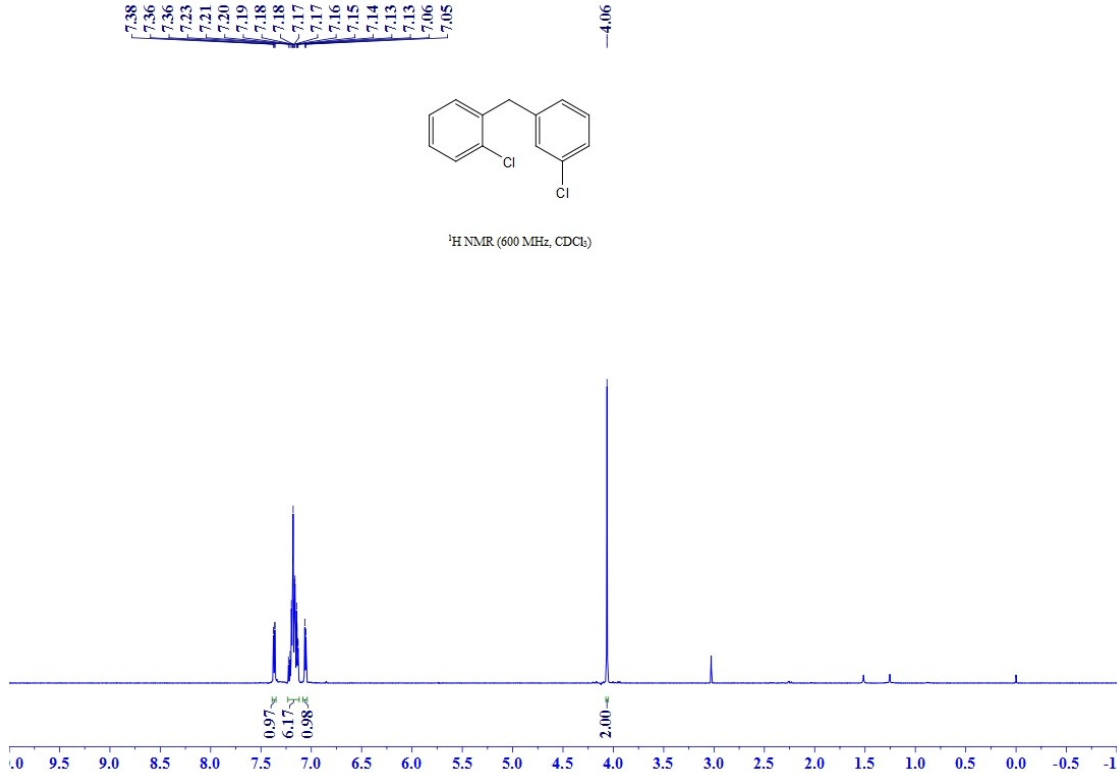

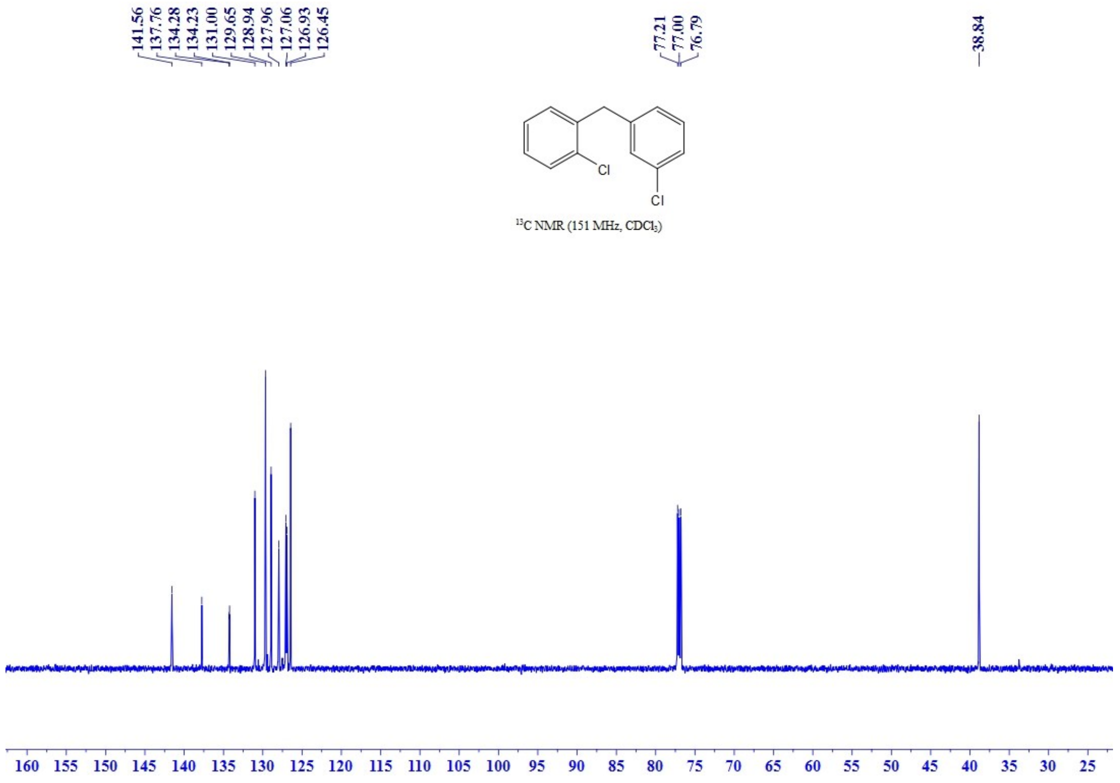

^{1}H and ^{13}C NMR spectra of compound **3af**

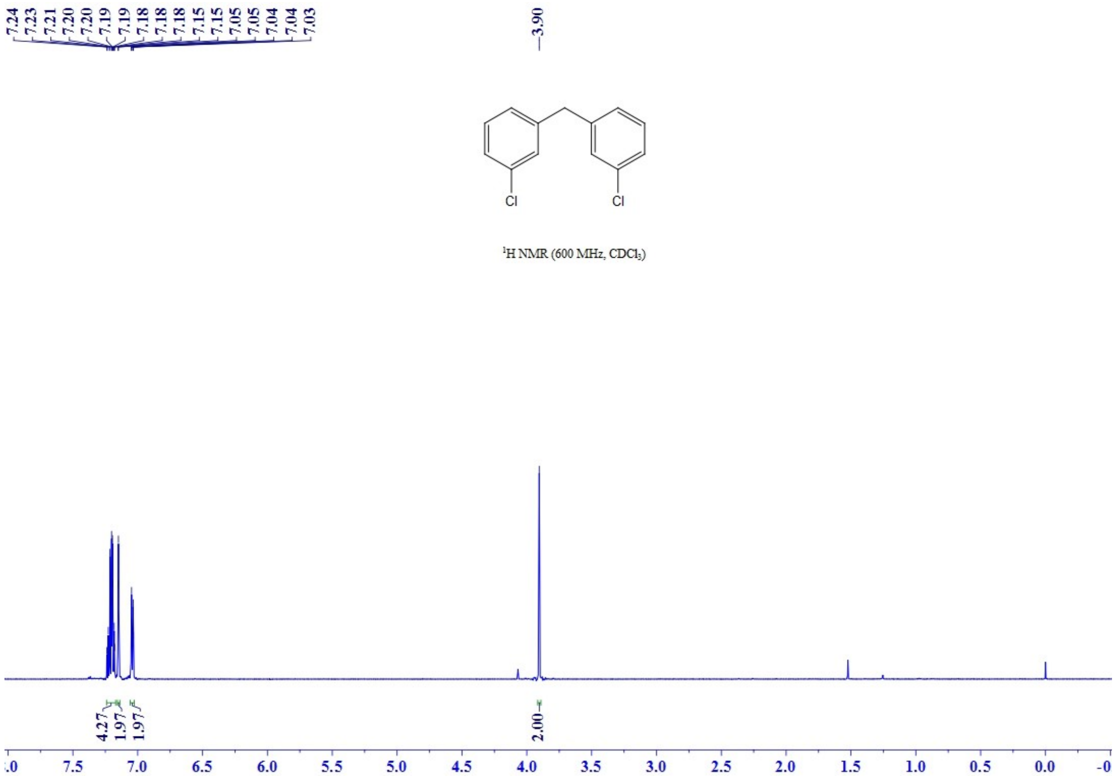

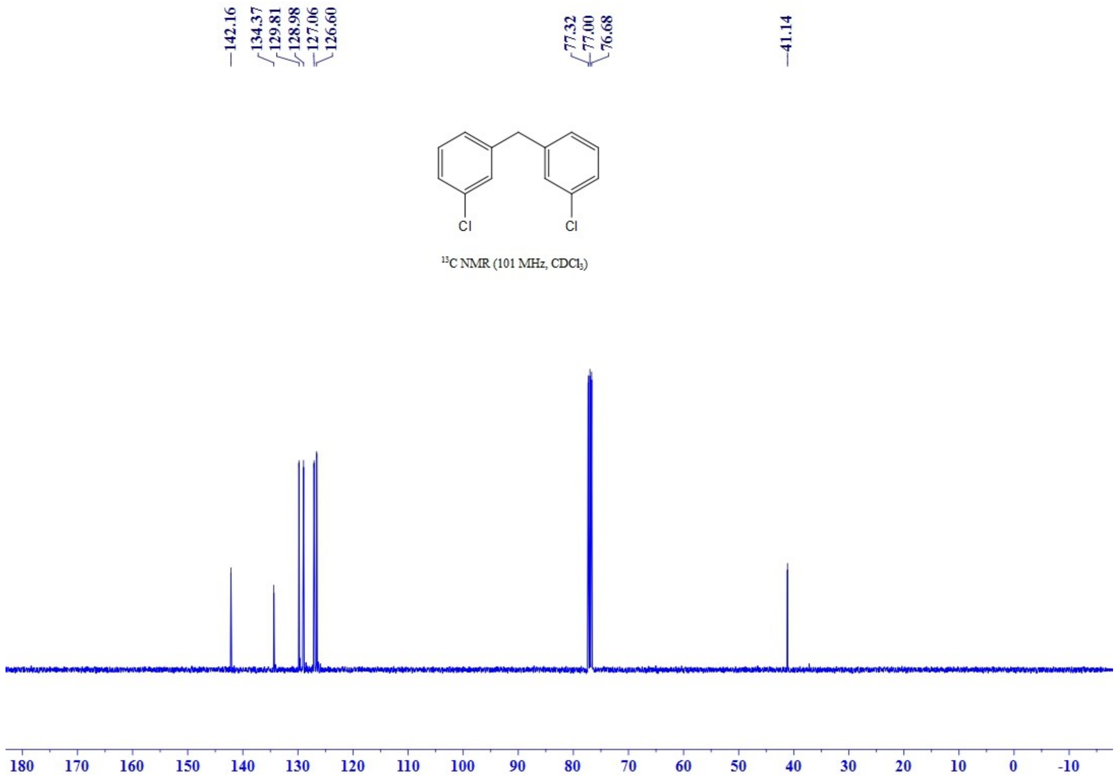

^{1}H and ^{13}C NMR spectra of compound **3ag**

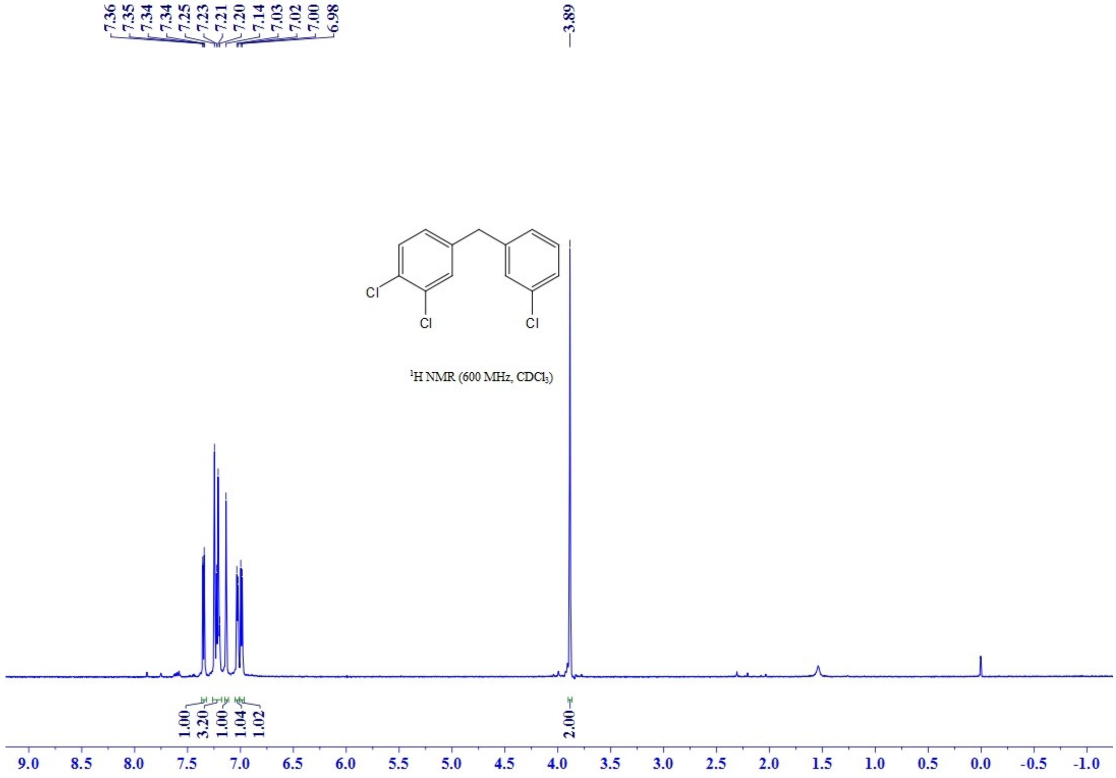

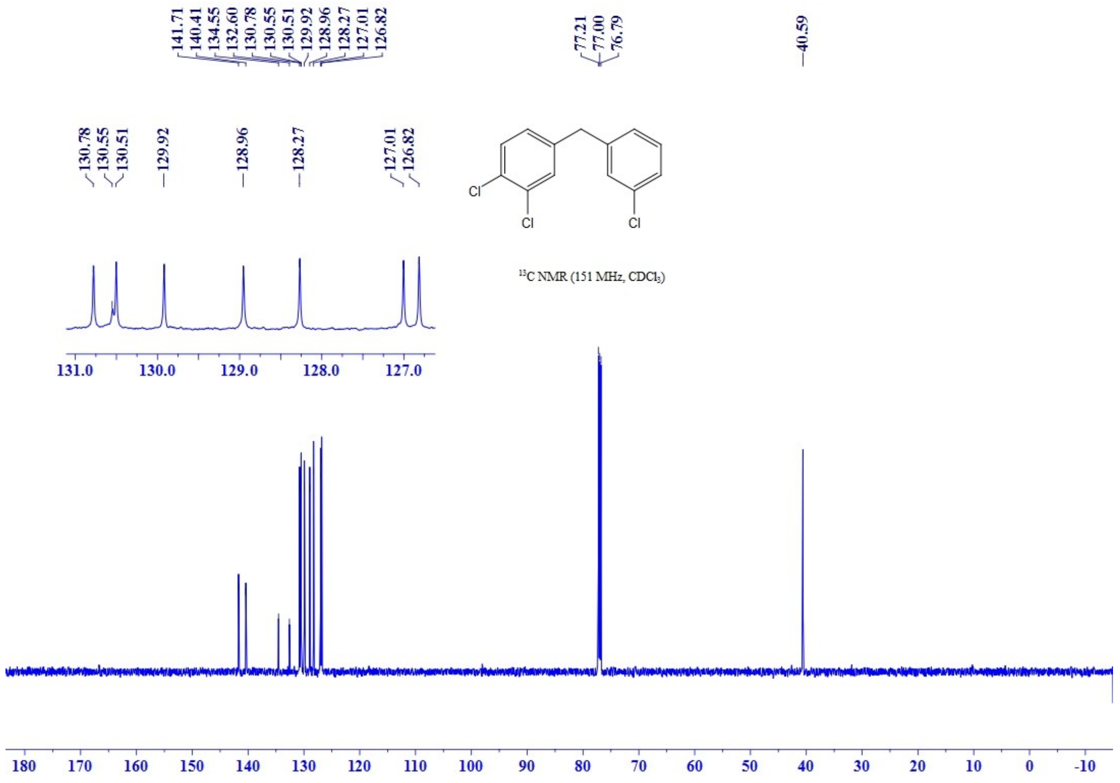

^{1}H, ^{19}F and ^{13}C NMR spectra of compound **3ah**

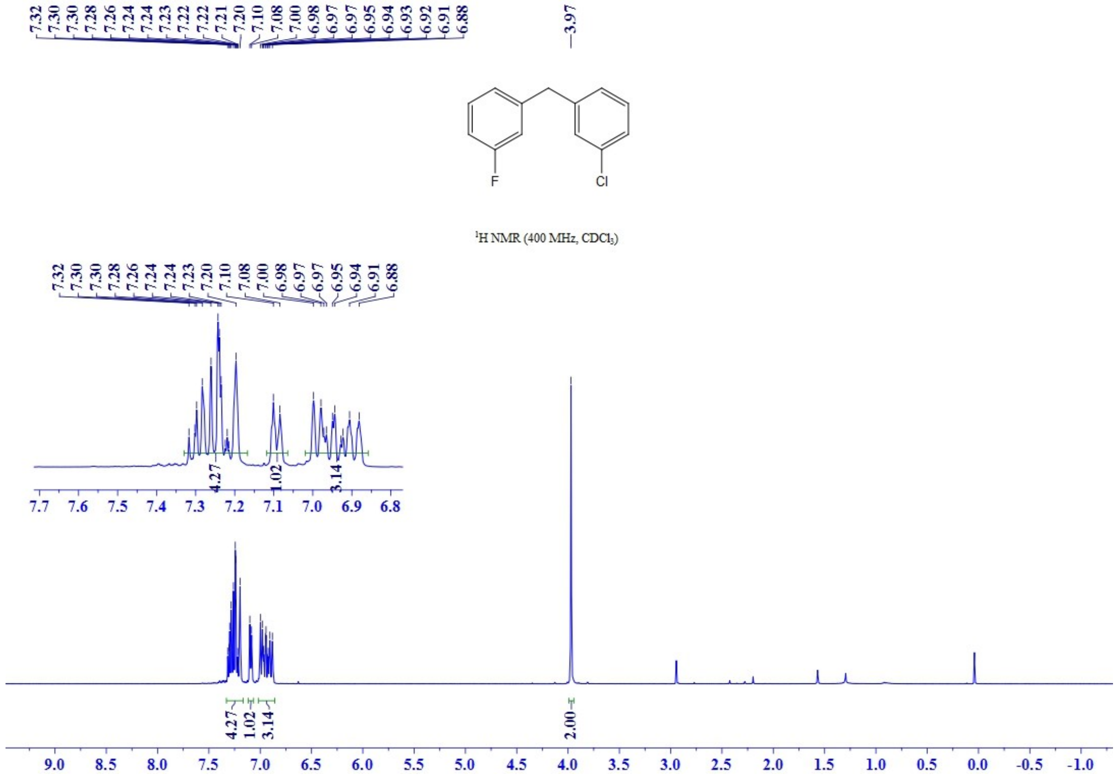

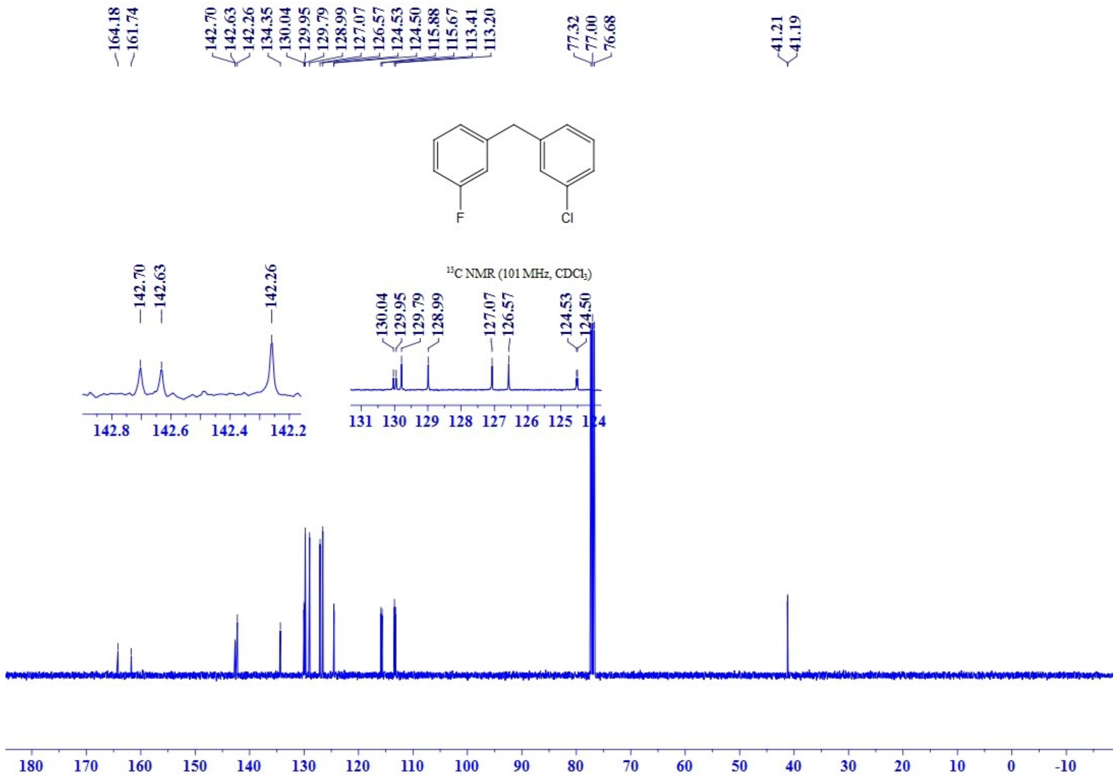
164.18
161.74
142.70
142.63
142.26
134.35
130.04
129.95
129.79
128.99
127.07
126.57
124.53
124.50
115.88
115.67
113.41
113.20
77.32
77.00
76.68
41.21
41.19
F
Cl
13C NMR (101 MHz, CDCl3)
142.8
142.6
142.4
142.2
131 130 129 128 127 126 125 124
180 170 160 150 140 130 120 110 100 90 80 70 60 50 40 30 20 10 0 -10

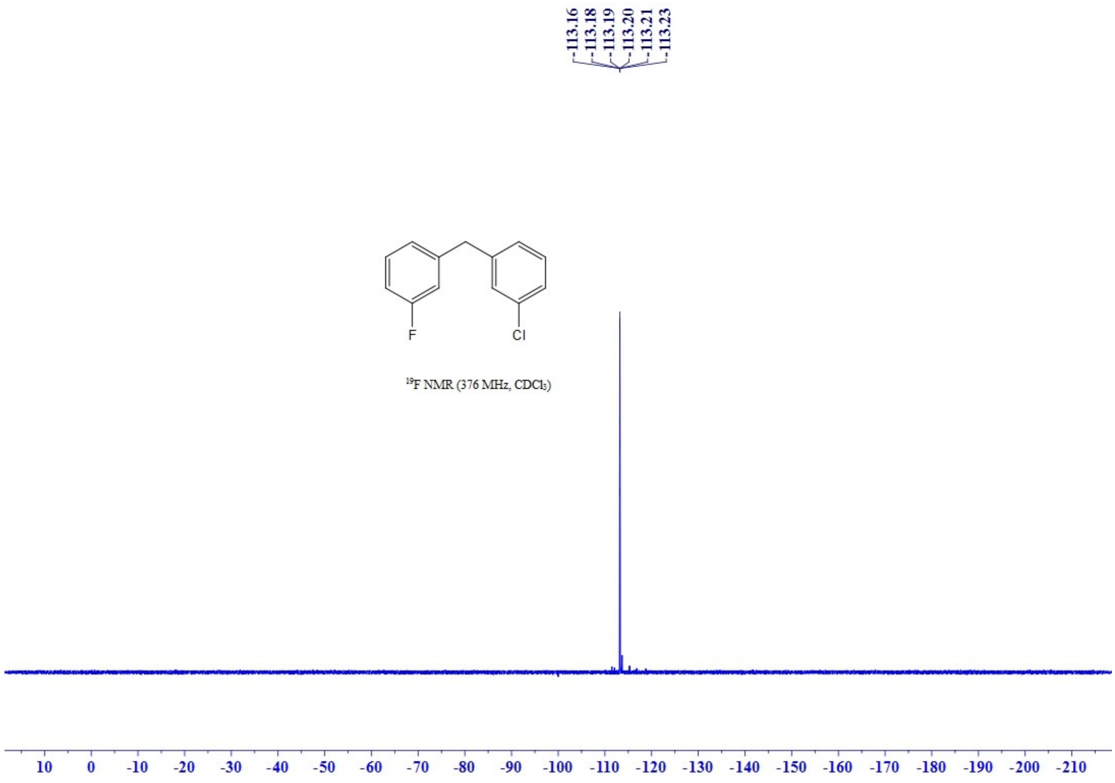
-113.16
-113.18
-113.19
-113.20
-113.21
-113.23
F
Cl
19F NMR (376 MHz, CDCl3)
10 0 -10 -20 -30 -40 -50 -60 -70 -80 -90 -100 -110 -120 -130 -140 -150 -160 -170 -180 -190 -200 -210

^{1}H and ^{13}C NMR spectra of compound **3ai**

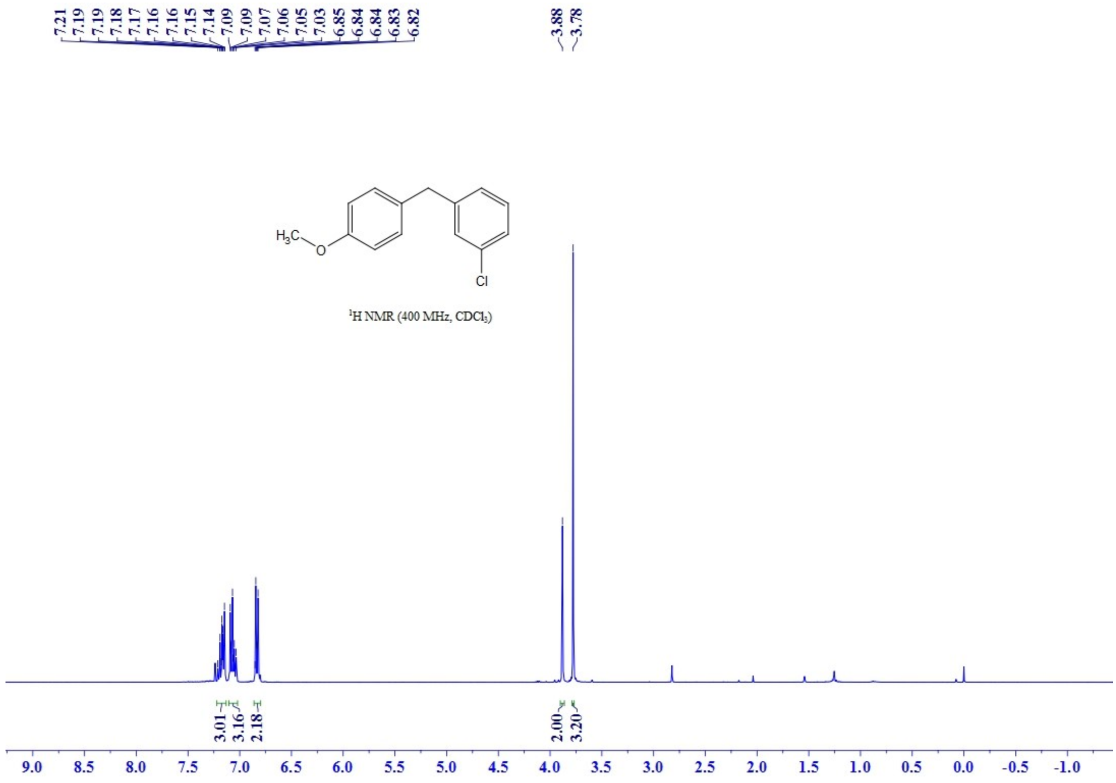

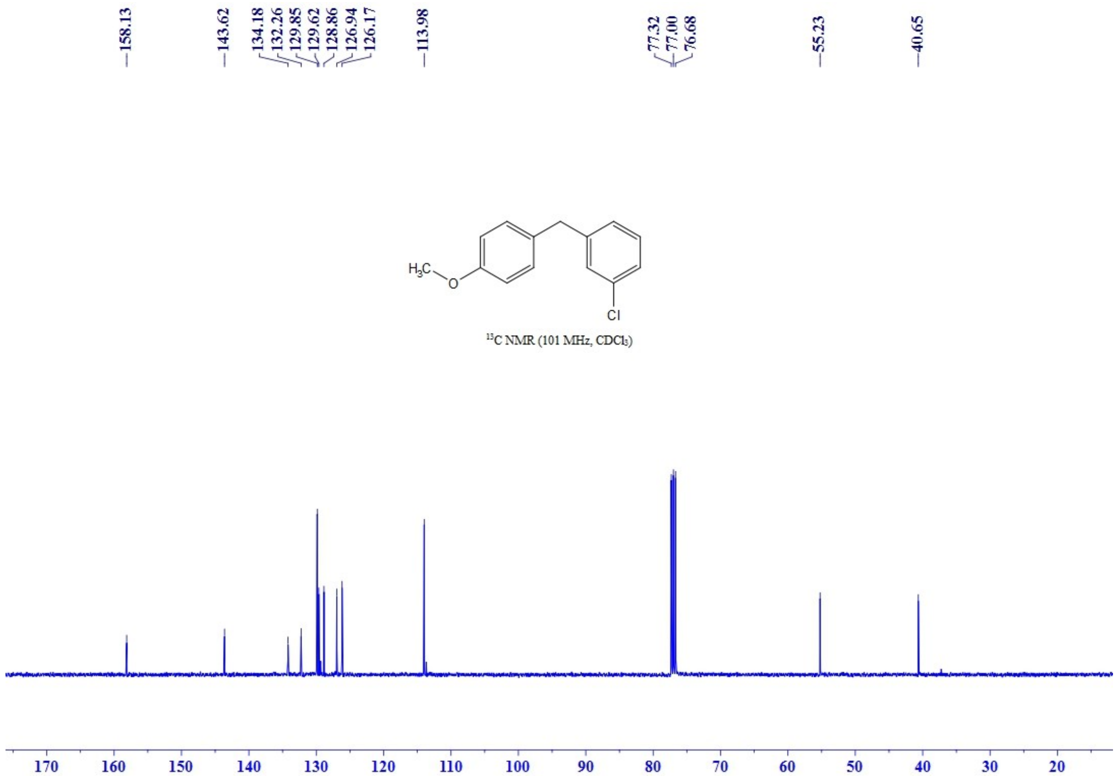

^{1}H and ^{13}C NMR spectra of compound **3aj**

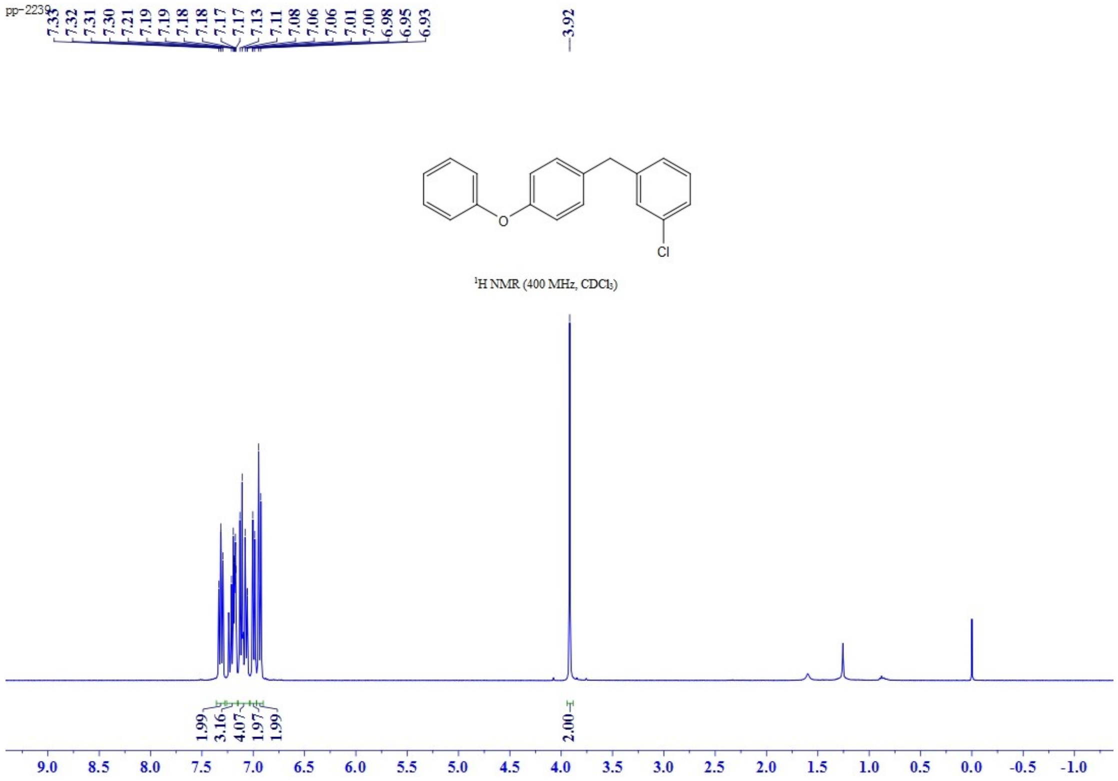

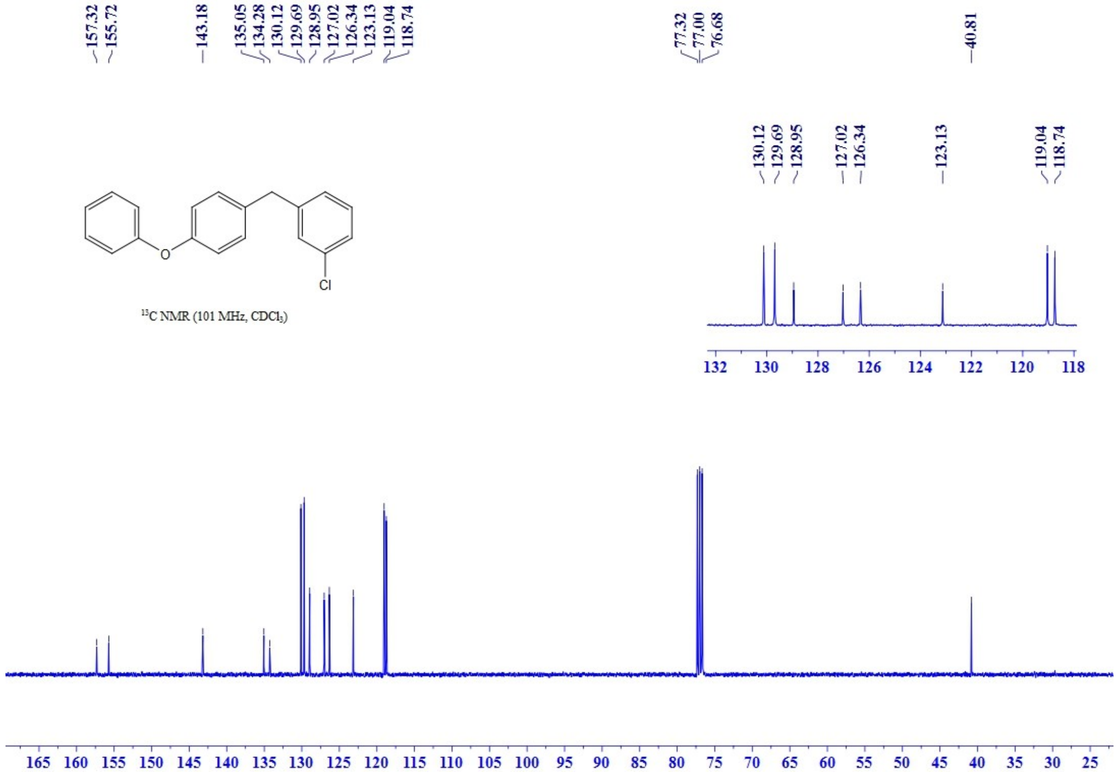

^{1}H and ^{13}C NMR spectra of compound **3ak**

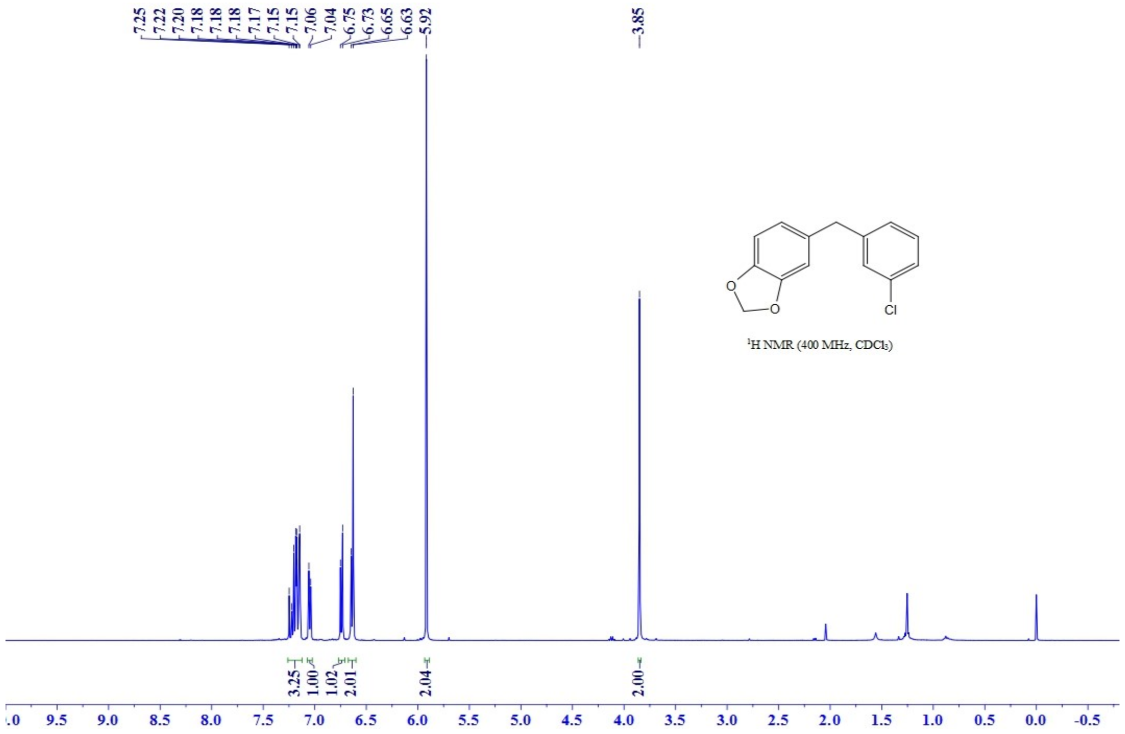

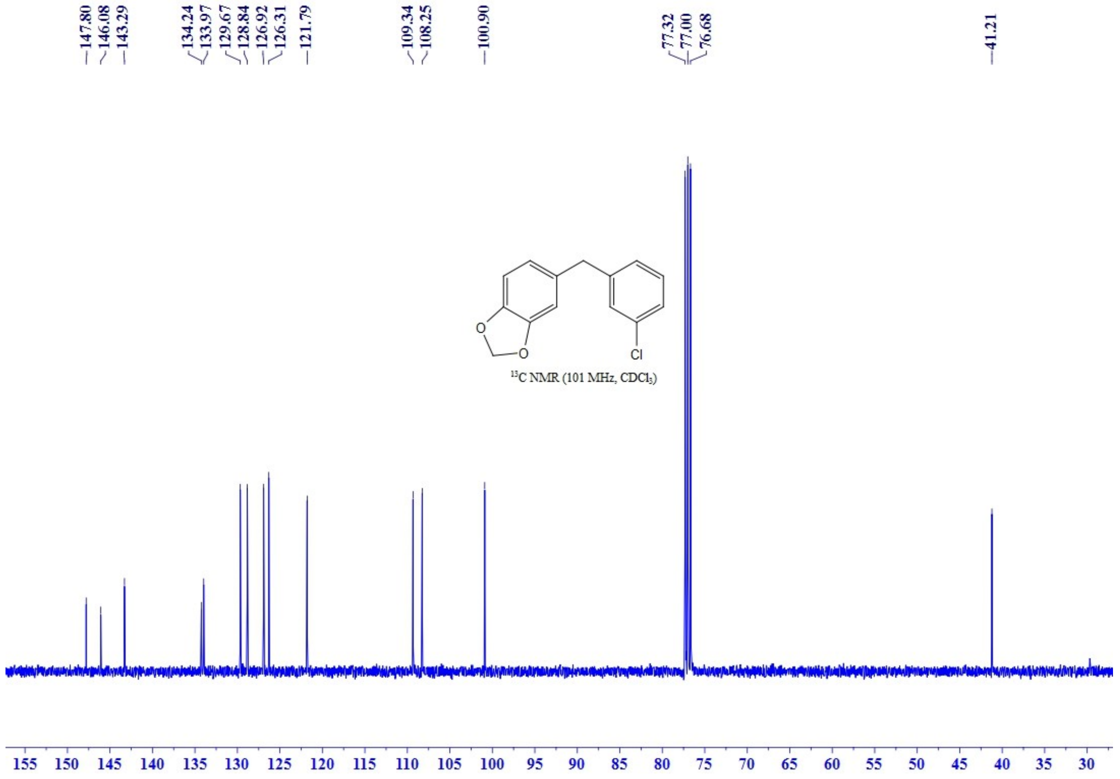

^{1}H, ^{19}F and ^{13}C NMR spectra of compound **3al**

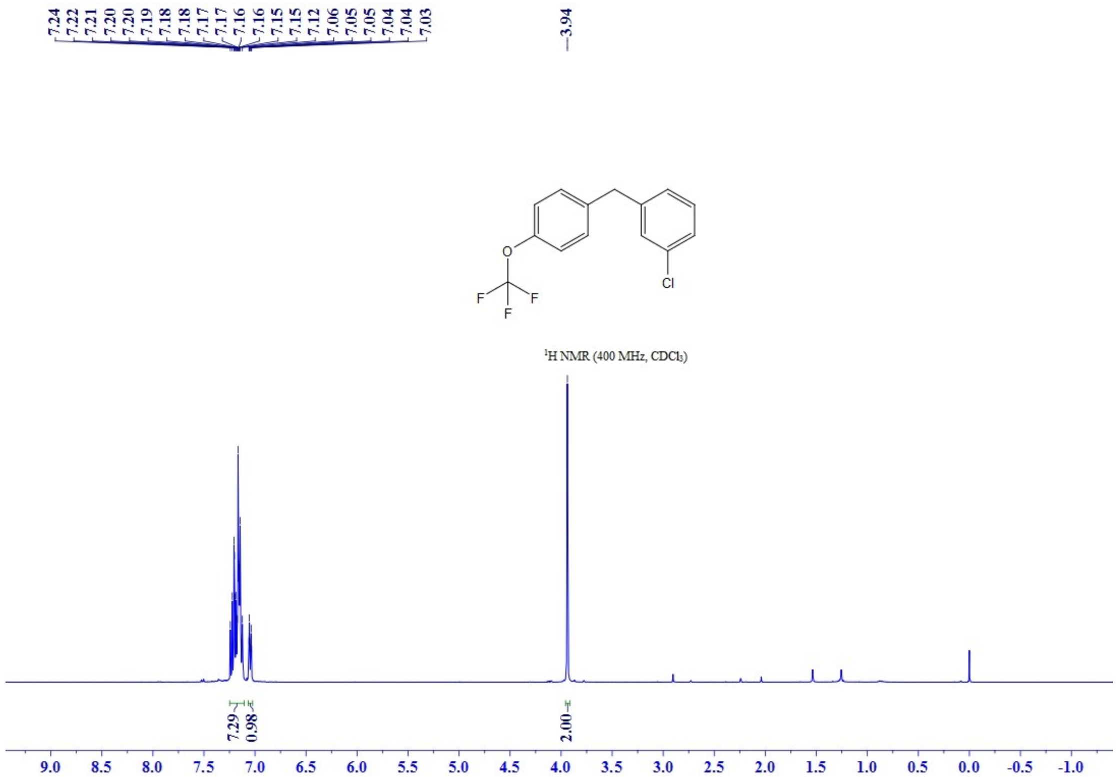

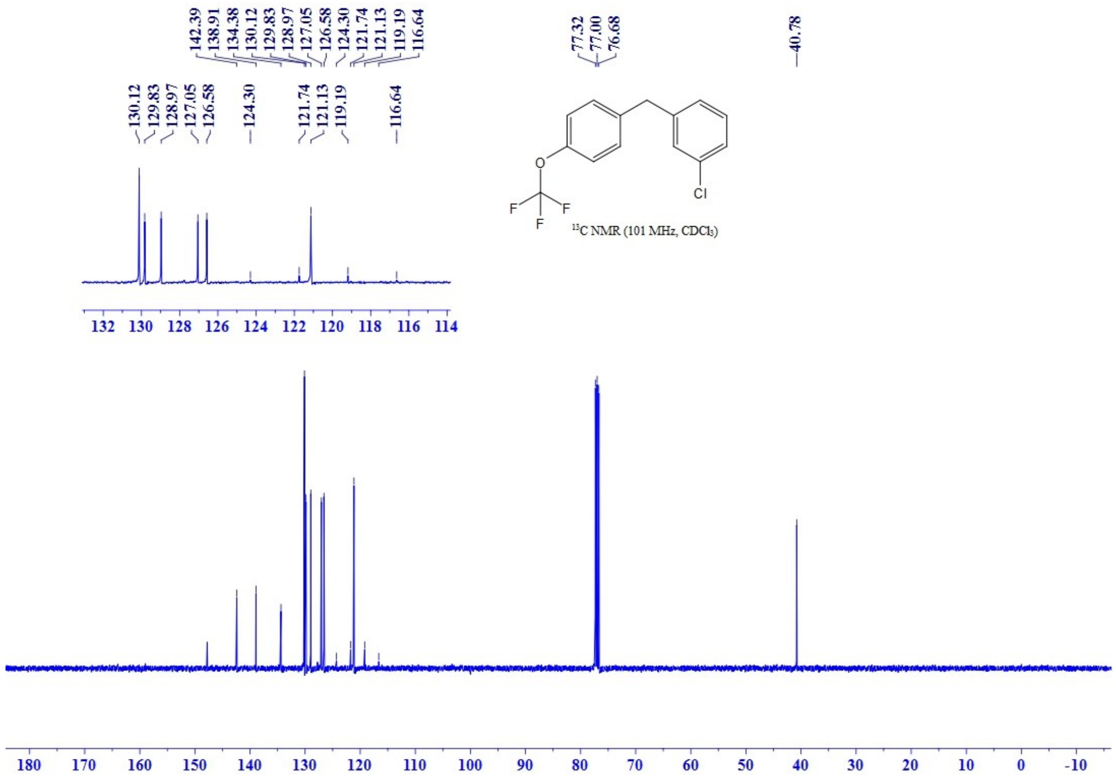

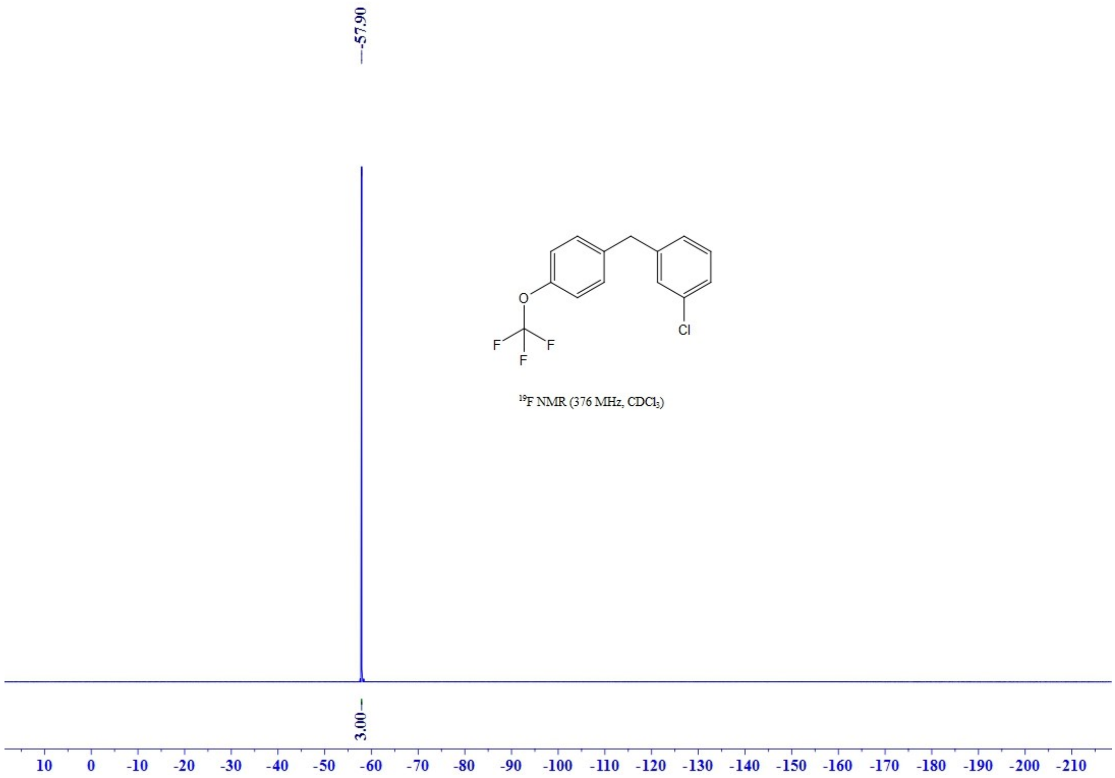

^{1}H, ^{19}F and ^{13}C NMR spectra of compound **3am**

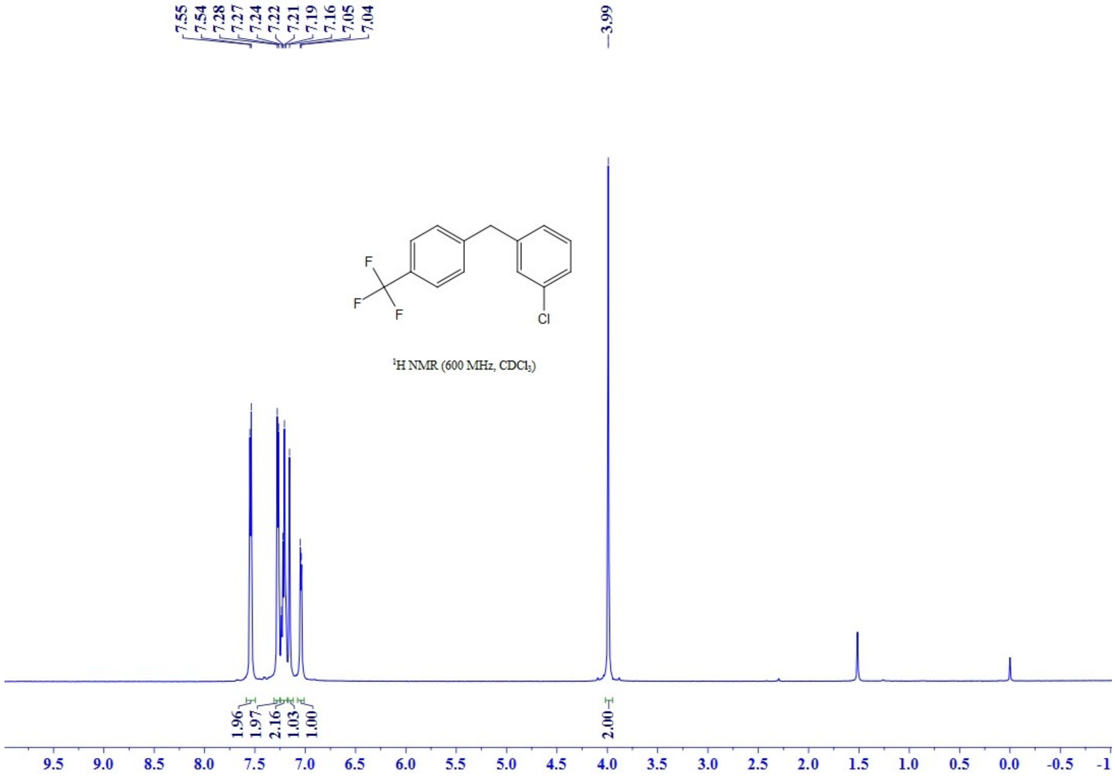

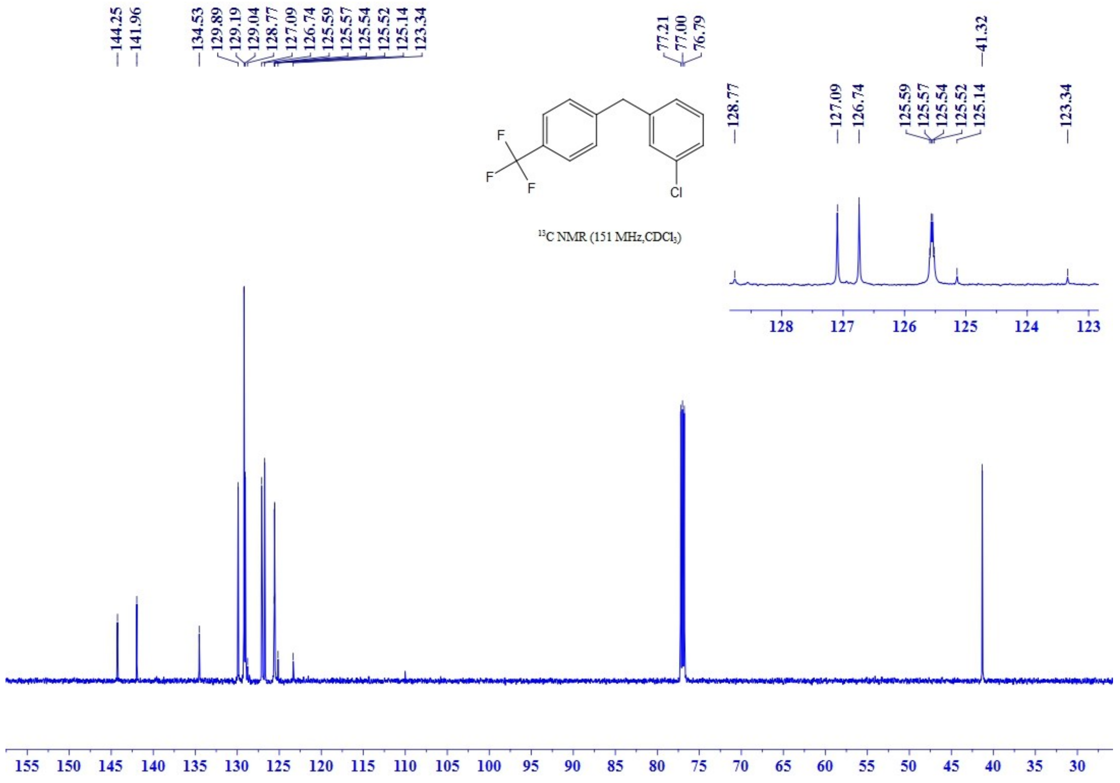
144.25
141.96
134.53
129.89
129.19
129.04
128.77
127.09
126.74
125.59
125.57
125.54
125.52
125.14
123.34
77.21
77.00
76.79
41.32
F
F
F
Cl
13C NMR (151 MHz,CDCl3)
128.77
127.09
126.74
125.59
125.57
125.54
125.52
125.14
123.34
128 127 126 125 124 123
155 150 145 140 135 130 125 120 115 110 105 100 95 90 85 80 75 70 65 60 55 50 45 40 35 30

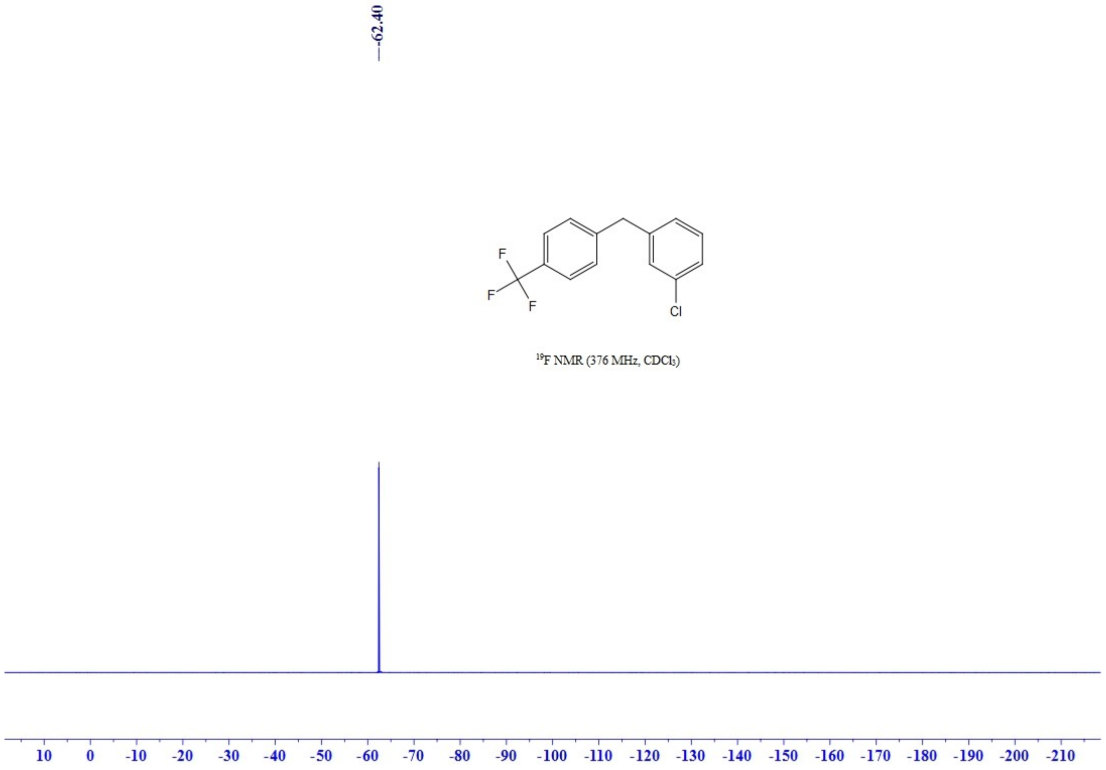
-62.40
F
F
F
Cl
19F NMR (376 MHz, CDCl3)
10 0 -10 -20 -30 -40 -50 -60 -70 -80 -90 -100 -110 -120 -130 -140 -150 -160 -170 -180 -190 -200 -210

^{1}H and ^{13}C NMR spectra of compound **3an**

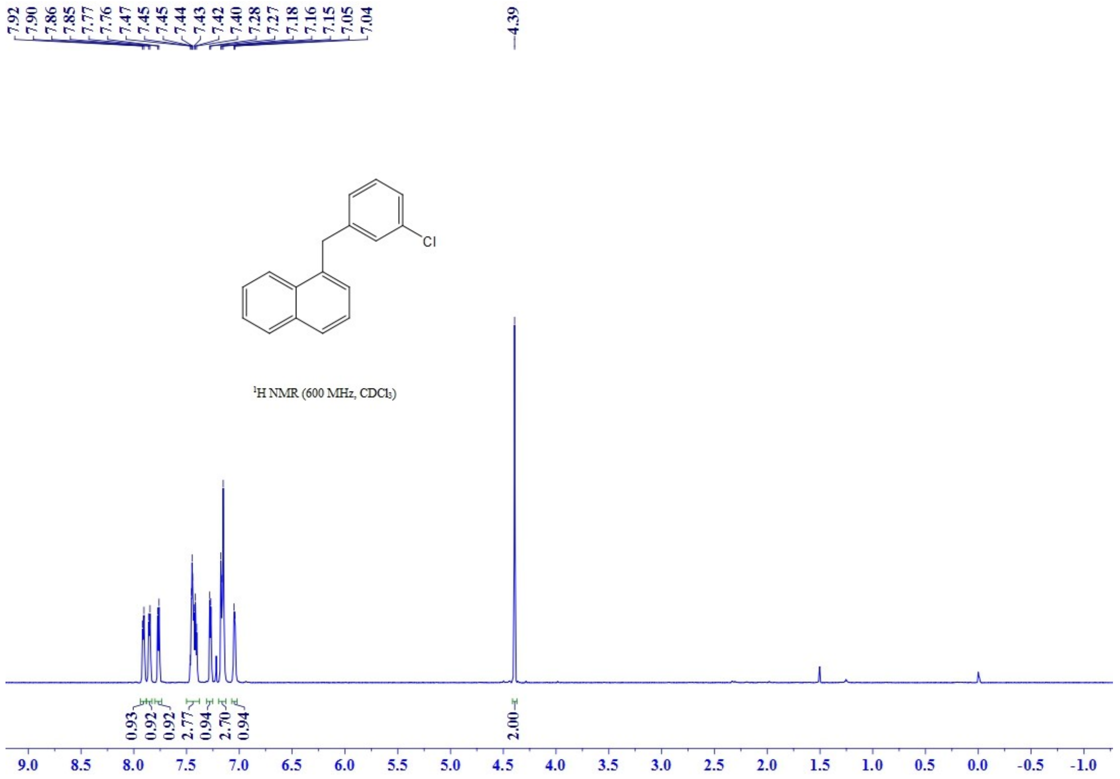

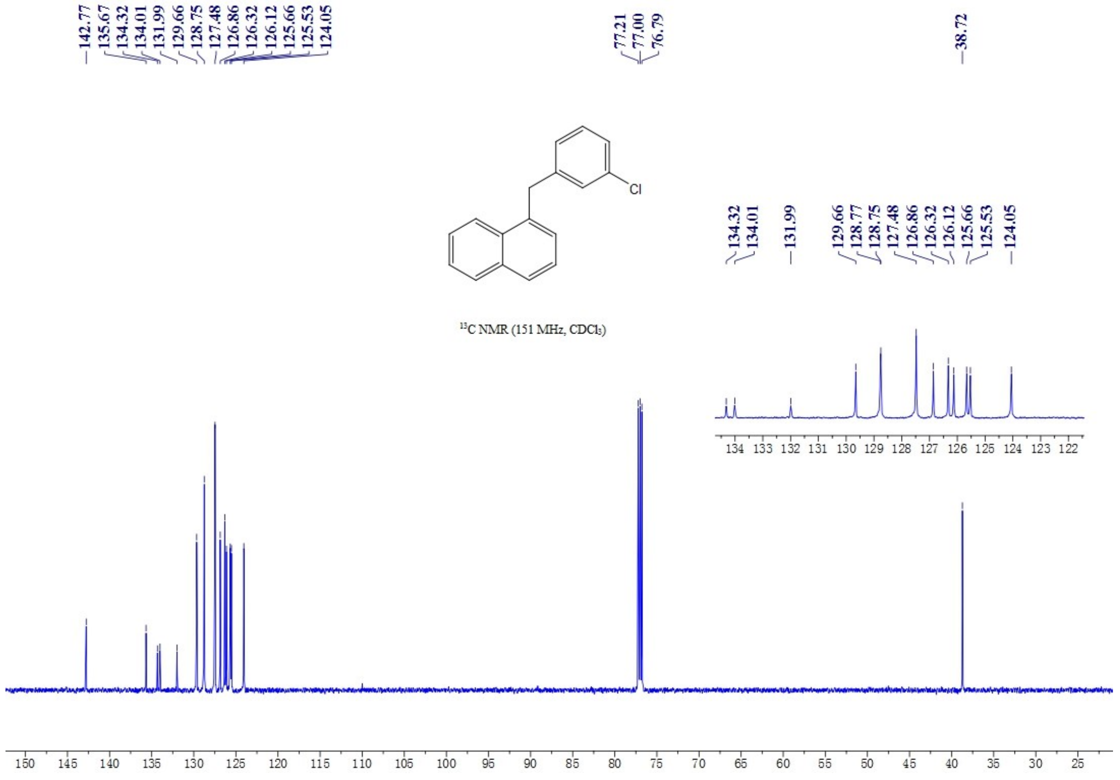

^{1}H and ^{13}C NMR spectra of compound **3ao**

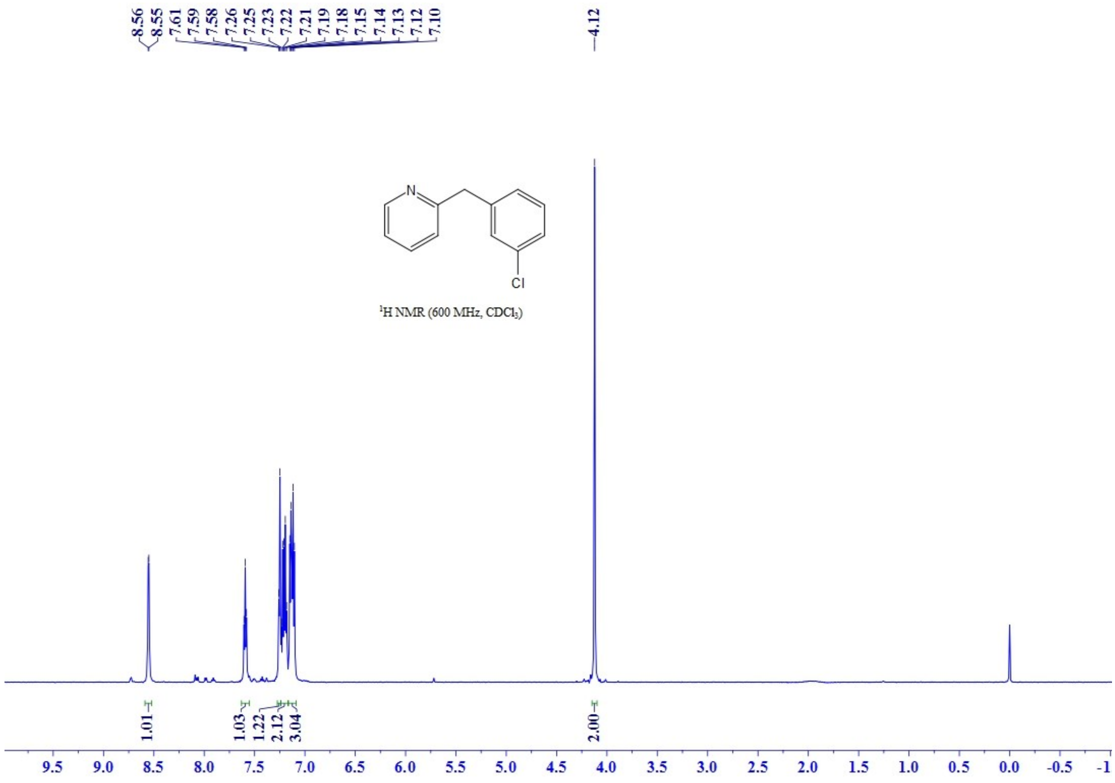

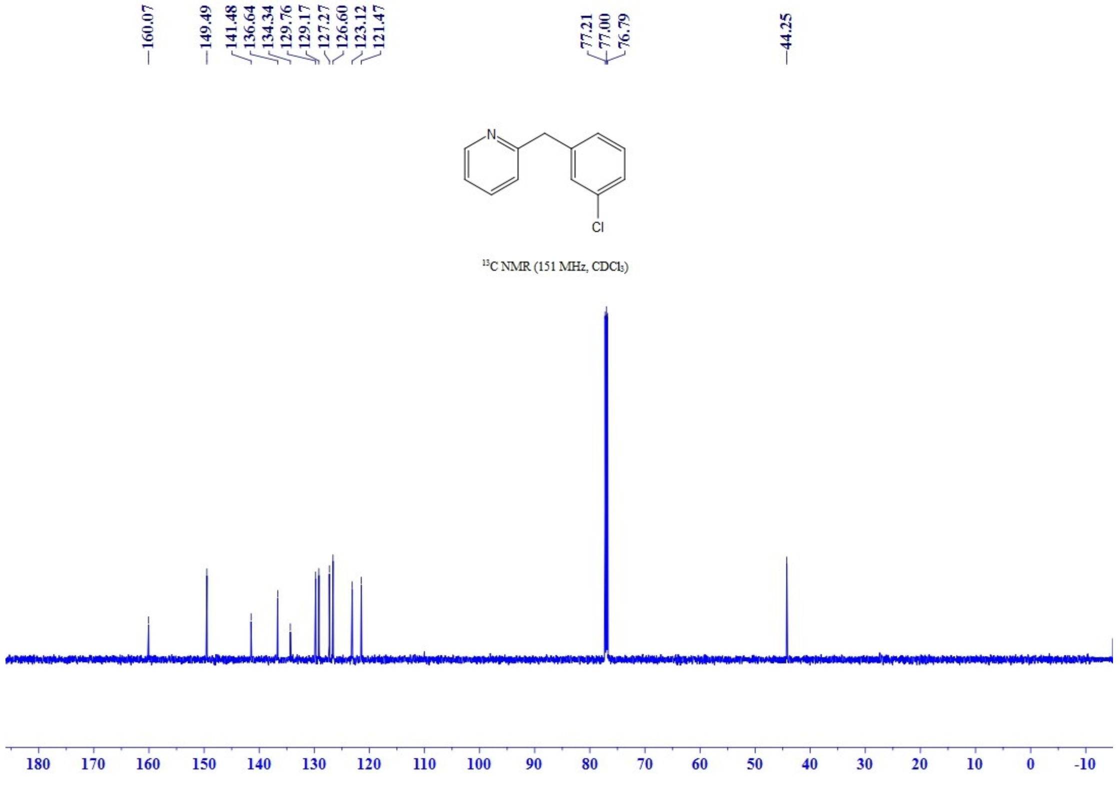

^1H and ^{13}C NMR spectra of compound **3ap**

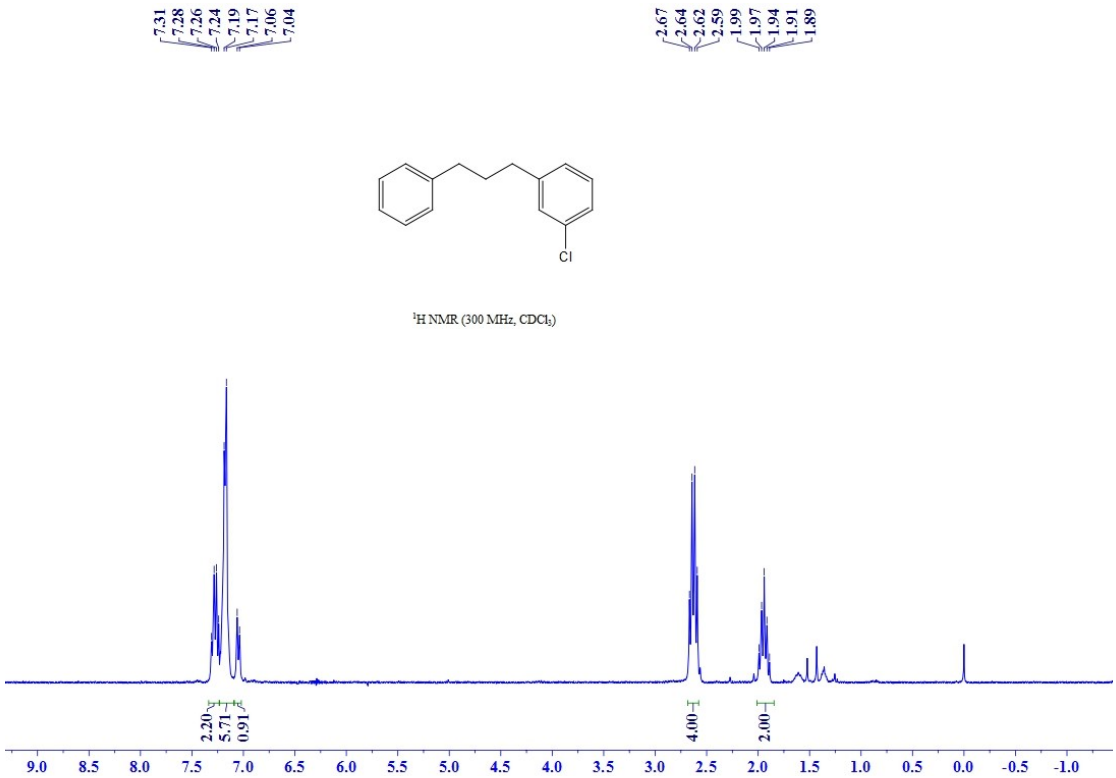

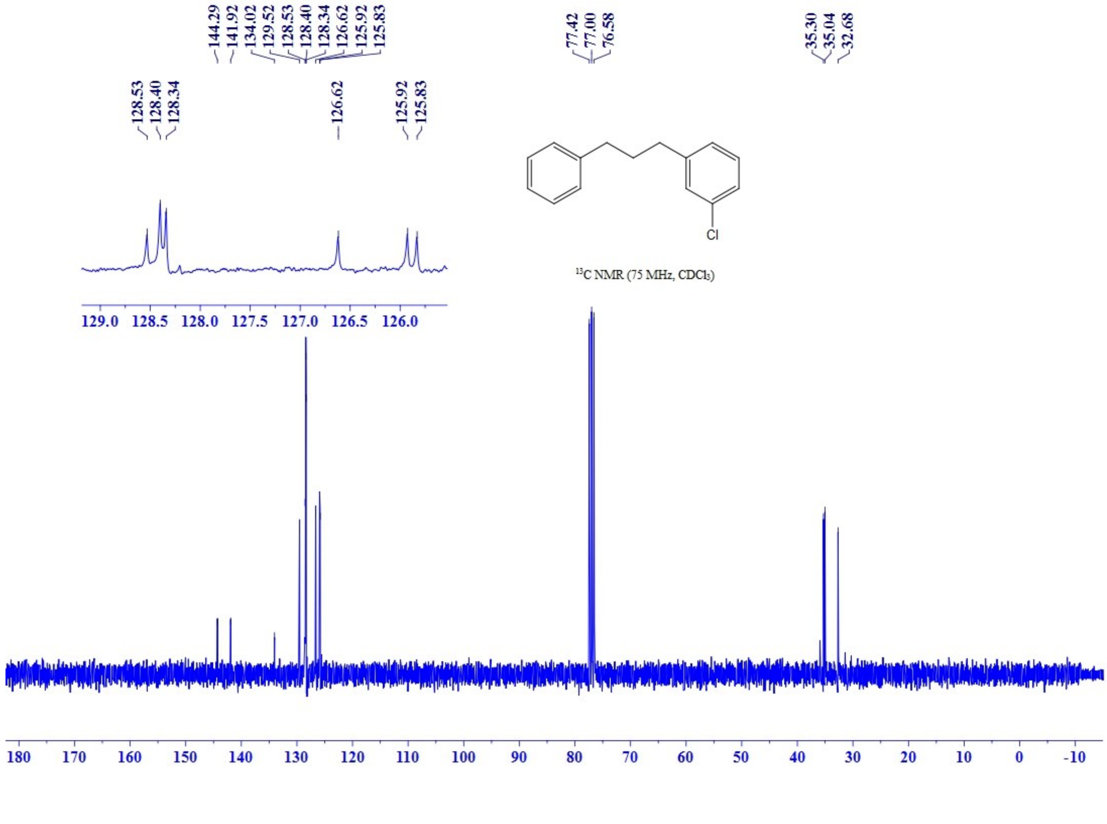

^{1}H and ^{13}C NMR spectra of compound **6**

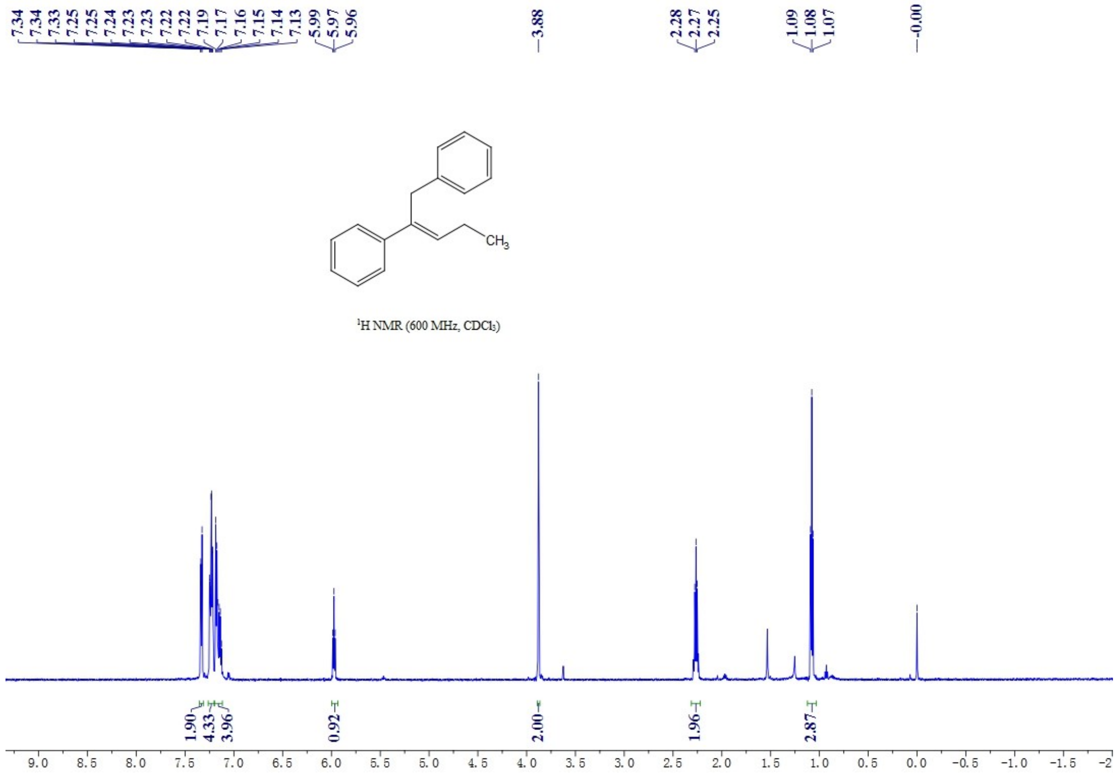

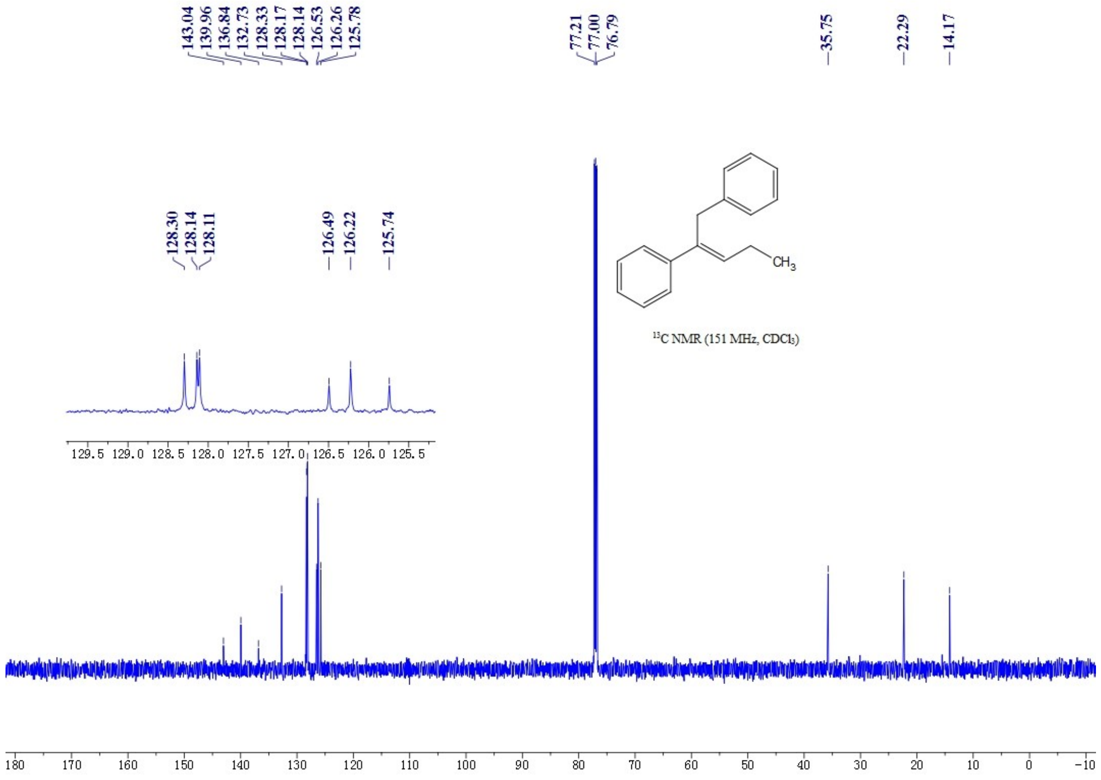

Supplement: SC-013-D2SC01909D-s001 [file SC-013-D2SC01909D-s001.pdf]
